# Cohort profile: creation of the SAIL MELD-B e-cohort (SMC) and SAIL MELD-B children and young adult e-cohort (SMYC) to investigate the lived experience of the ‘burdensomeness’ of multimorbidity

**DOI:** 10.1136/bmjopen-2024-087946

**Published:** 2025-01-07

**Authors:** Roberta Chiovoloni, Jakub J Dylag, Nisreen A Alwan, Ann Berrington, Michael Boniface, Nic Fair, Emilia Holland, Rebecca Hoyle, Mozhdeh Shiranirad, Sebastian Stannard, Zlatko Zlatev, Rhiannon K Owen, Simon Fraser, Ashley Akbari

**Affiliations:** 1 Population Data Science, Faculty of Medicine, Swansea University Medical School, Swansea, UK; 2 School of Electronics and Computer Science, University of Southampton, Southampton, UK; 3 School of Primary Care, Population Sciences and Medical Education, Faculty of Medicine, Southampton General Hospital, Southampton, UK; 4 NIHR Southampton Biomedical Research Centre, University Hospital Southampton NHS Foundation Trust, Southampton, UK; 5 Department of Social Statistics and Demography, University of Southampton, Southampton, UK; 6 School of Mathematical Sciences, University of Southampton, Southampton, UK

**Keywords:** Chronic Disease, Electronic Health Records, STATISTICS & RESEARCH METHODS, Multimorbidity

## Abstract

**Abstract:**

**Purpose:**

We have established the SAIL MELD-B electronic cohort (e-cohort SMC) and the SAIL MELD-B children and Young adults e-cohort (SMYC) as a part of the Multidisciplinary Ecosystem to study Lifecourse Determinants and Prevention of Early-onset Burdensome Multimorbidity (MELD-B) project. Each cohort has been created to investigate and develop a deeper understanding of the lived experience of the ‘burdensomeness’ of multimorbidity by identifying new clusters of burdensomeness concepts, exploring early life risk factors of multimorbidity and modelling hypothetical prevention scenarios.

**Participants:**

The SMC and SMYC are longitudinal e-cohorts created from routinely collected individual-level population-scale anonymised data sources available within the Secure Anonymised Information Linkage (SAIL) Databank. They include individuals with available records from linked health and demographic data sources in SAIL at any time between 1 January 2000 and 31 December 2022. The SMYC e-cohort is a subset of the SMC, including only individuals born on or after the cohort start date.

**Findings to date:**

The SMC and SMYC cohorts include 5 180 602 (50.3% female and 49.7% male) and 896 155 (48.7% female and 51.3% male) individuals, respectively. Considering both primary and secondary care health data, the five most common long-term conditions for individuals in SMC are ‘Depression’, affecting 21.6% of the cohort, ‘Anxiety’ (21.1%), ‘Asthma’ (17.5%), ‘Hypertension’ (16.2%) and ‘Atopic Eczema’ (14.1%) and the five most common conditions for individuals in SMYC are ‘Atopic Eczema’ (21.2%), ‘Asthma’ (11.6%), ‘Anxiety’ (6.0%), ‘Deafness’ (4.6%) and ‘Depression’ (4.3%).

**Future plans:**

The SMC and SMYC e-cohorts have been developed using a reproducible, maintainable concept curation pipeline, which allows for the cohorts to be updated dynamically over time and manages for the request and processing of further approved long-term conditions and burdensomeness concepts extraction. Best practices from the MELD-B project can be utilised across other projects, accessing similar data with population-scale data sources and trusted research environments.

**STRENGTHS AND LIMITATIONS OF THIS STUDY**

- Secure Anonymised Information Linkage (SAIL) Multimorbidity Cohort and SAIL Multimorbidity and Young adult Cohort are representative of the Welsh population.
- Anonymised cohorts serve as an effective strategy for overcoming consent-related barriers, enabling seamless data aggregation and analysis.
- The creation of a reproducible concept curation pipeline to manage and process data extraction for the e-cohorts enables efficient delivery of data sets in support of multiple research questions and outcomes.
- Routine data do not capture data on important aspects such as quality of life and it can be subject to missing data or errors.
- Lack of coverage of burdensomeness indicators in routine data.

## Introduction

 The prevalence of Multiple Long-Term Condition Multimorbidity (MLTC-M), commonly defined as the co-occurrence of two or more chronic conditions in an individual, has increased in many regions of the world as a result of many factors, including changes in lifestyles, the ageing population and increasing diagnosis of long-term conditions (LTCs).^1^

In the UK, it is estimated that more than half of the population aged 65 and above suffers from two or more LTCs, and it is predicted that by 2035, two-thirds of people aged over 65 will experience MLTC-M.^2^

MLTC-M is often a burden for patients, their carers and their health service providers. It is associated with reduced quality of life,^3^ fragmented and costly care,^4 5^ pharmacy,^6^,^8^ physiological distress, extended hospital stays,^9 10^ increased mortality^11^ and it substantially contributes to healthcare inefficiency and cost in both primary and secondary care settings.^12^,^15^

However, to date, different aspects of MLTC-M are not well understood.^16^ For example, most MLTC-M studies have focused only on a selected subset of the population, specifically older individuals in high-income countries,^17^ a small number of conditions^18^ and the analysis of clustering of conditions in repeated cross-sectional studies.^19^,^24^

There is limited research examining the association between MLTC-M, socioeconomic status and longitudinal trends,^25^,^27^ and limited evidence regarding other social and behavioural determinants that could be fundamental in the emergence and evolution of less common MLTC-M patterns.^28^ Additionally, few studies investigate how the timing and nature of exposure to risk factors influence the accrual of LTCs,^2729^,^33^ and little research focuses on how to prevent MLTC-M development.^34^

The Multidisciplinary Ecosystem to study Lifecourse Determinants and Prevention of Early-onset Burdensome Multimorbidity (MELD-B) collaboration aims to address some of these key gaps in the evidence in MLTC-M research by developing a deeper understanding of the lived experience of ‘burdensomeness’ of multimorbidity, identifying new clusters of burdensome MLTC-M and their key early-life risk factors, mapping trajectories across the lifecourse towards burdensome clusters in those under 65 and modelling prevention scenarios to inform policy.^35^ These will be achieved through the analysis of birth cohorts and routinely collected electronic health record (EHR) data sources, using a combination of Artificial-Intelligence. Questions are built around several key areas of inquiry: clustering individuals based on burdensomeness concepts, analysing the determinants of these burdensomeness clusters, examining the sequence of acquisition of burdensomeness features identifying early determinants of health outcomes, analysing the sequence of sentinel conditions (the first LTC acquired in the lifecourse) and subsequent accrual of burden. As well as the LTCs required for MLTC-M research, these burdensomeness concepts include indicators of the ‘work’ associated with living with MLTC-M such as symptoms, emotions, indicators of financial stress and observable and measurable information relevant to health or healthcare, such as medical diagnoses, blood tests, appointments, hospital admissions and number of medications. For a more detailed description of the MELD-B objectives and structure.^35^,^37^

To support the MELD-B project, we have created the SAIL MELD-B e-cohort (SMC) and the SAIL MELD-B children and Young adult e-cohort (SMYC), longitudinal population-based e-cohorts based in Wales. The e-cohorts are representative of the wider population in terms of sex, age and socioeconomic deprivation.

The e-cohorts are developed to support multiple research questions within the MELD-B work packages and collaboration. They will be used as maintainable research ready data assets enabling the MELD-B collaboration to perform clustering, sequencing and statistical analyses to identify the critical time-points for public health intervention.^38^ This will both allow the evaluation of the burden of MLTC-M on individuals and also provide insights into the wider determinants of MLTC-M, the temporal dynamics of disease and burden progression, and potential effects of intervention and prevention.

The inclusion of newborns and young individuals in the e-cohort will allow us to better understand how social, biological and environmental factors in early life contribute to the risk of developing MLTC-M, as there is substantial evidence indicating the critical role of early life in determining health during childhood and adulthood.^39^,^43^

## Cohort description

The SMC is a longitudinal e-cohort defined using routinely collected anonymised linked demographic, administrative and EHR data sources available within the Secure Anonymised Information Linkage (SAIL) Databank.^44^ The SMYC is a subset of the SMC, including only individuals born after the study start date with demographic data available before 18 years of age, and with consistent maternal records.

The MELD-B coinvestigators derived burdensomeness concepts from a qualitative evidence synthesis with extensive patient and public involvement. Extracting health service interactions and records from routine data can provide measurable observations for the derived concepts for individuals in SMC and SMYC. This will offer fundamental insights into measuring and conceptualising burdensome MLTC-M.

All codes and scripts used in this study are available for others to access here: https://github.com/SwanseaUniversityDataScience/1377-MELD_B-CohortCuration.

### Sail Databank and data sources

The SAIL Databank (www.saildatabank.com) contains anonymised, encrypted, routinely collected individual-level population-scale linkable data sources for all Welsh residents using any National Health Service (NHS) UK-wide services and any individuals residing outside of Wales using NHS Wales services. To ensure anonymity and confidentiality, each individual is assigned a unique identifier (Anonymised Linking Field, ALF), used to link together different data sources at the individual level. The ALF is generated through a double encryption process: Digital Health and Care Wales (DHCW) uses NHS number or a combination of unique demographic information (such as sex, name and date of birth) to generate a unique identifier, which is then further encrypted within the SAIL Databank. This process ensures that no single organisation can decrypt the records, making SAIL a TRE for record-linkage studies.^45^,^47^ Note that while SAIL is referred to as an anonymised TRE, the Information Commissioner’s Office (ICO) might also describe it as pseudo-anonymised.^48^

To build the SMC and SMYC, we linked demographic and mortality data sources: the Welsh Demographic Service Data set (WDSD), the Annual District Death Extract (ADDE) from the Office for National Statistics (ONS) mortality register, the Annual District Birth Extract (ADBE) from the ONS birth register, the National Community Child Health database (NCCH) and the Maternal Indicators DataSet (MIDS), see online supplemental table S1.

The baseline demographic characteristics of the e-cohorts include: ALF, Sex (male or female), Week of Birth (WOB), Date of Death (DOD) where applicable, Ethnic group,^49^ Lower-layer Super Output Area, 2011 version (LSOA 2011) and Welsh Index of Multiple Deprivation, 2019 version (WIMD 2019). The last two provide insights on the socioeconomic status of the individuals at an area level: LSOAs are small areas containing around 1500 individuals used to link individual records to the WIMD 2019 to derive deprivation status.

Ethnic groups have been classified using two different classifications, the ONS and the New and Emerging Respiratory Virus Threats Advisory Group (NER) classifications, which have five and nine ethnicity categories, respectively.

The health data sources available to the MELD-B project include, the Welsh Longitudinal General Practice (WLGP) data, the Patient Episode Database for Wales (PEDW), the Emergency Department Dataset (EDDS), the Outpatient Database for Wales (OPDW) and the Welsh Results Reports Service (WRRS), the National Community Child Health Database (NCCH) and the Maternity indicators data set (MIDS), see online supplemental table S1.^50^

Currently, WLGP contains primary care data for 86% of the Welsh population registered with a General Practice (GP) and 80% of GP practices covering all local authorities in Wales.^51^ In Wales, primary care GP data are recorded using Read V2 codes, while data for secondary care episodes, such as hospital admissions, are recorded using the International Classification of Disease V.10 (ICD-10) and the Office of Population Censuses and Surveys codes V.4 (OPCS-4). Emergency department data have its own coding system.^52^

Data are available from different data sources at different times, and their quality improves over time. Given the requirements of the study and the completeness of the data sources, 1 January 2000 was chosen as the start date of the study and 1 January 1990 as the start date of data collection. Note where data sources start after these dates, their coverage begins from the respective data source’s start date.^50^

### Cohorts design

The SMC is a longitudinal population e-cohort including all people residing in Wales and registered with a Welsh GP between 1 January 2000 (identified as *cohort start* date) and 31 December 2022 (note that this is the cohort end date at the point of publication; however, follow-up data will be included once available in the SAIL Databank; identified as *cohort end date*), and it provides a generalisable population sample to the population of Wales with respect to sex, age and socioeconomic deprivation.^53^

The primary data source used to build the SMC is the WDSD.

**Figure 1:**
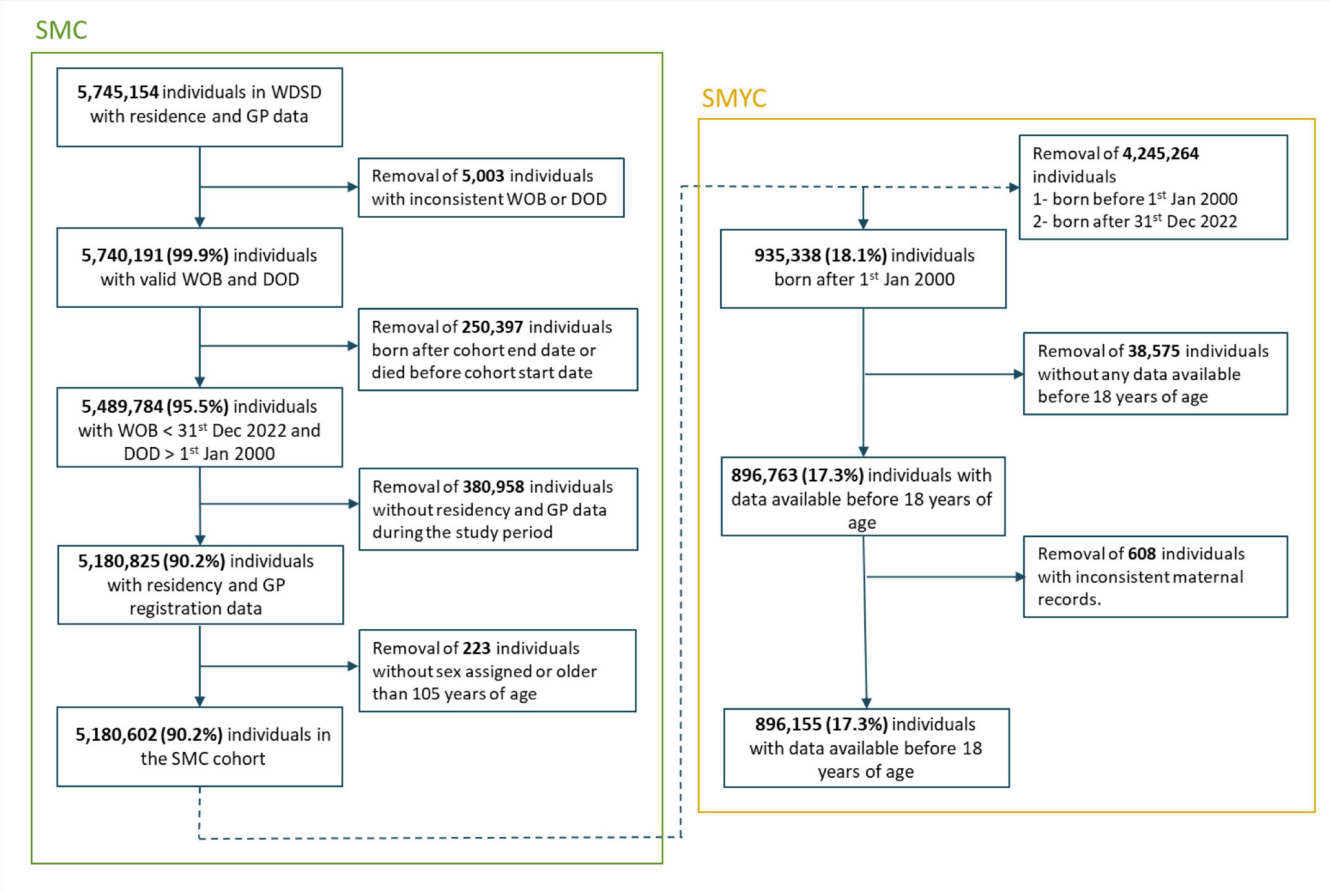
SAIL MELD-B consort diagram based on inclusion criteria. DOD, Date of Death; GP, general practise; SMC, SAIL MELD-B e-cohort; SMYC, SAIL MELD-B Young cohort; WDSD, Welsh Demographic Service Data set; WOB, Week of Birth.

As a longitudinal e-cohort, the number of individuals will change throughout the study as they can leave or join the e-cohort at any time during the study period. SMC entries include all residents in Wales who meet *all* of the following conditions, see figure 1:

- They have a consistent date of birth and/or DOD (we removed individuals with different date of birth/death in different data sources).
- They are alive between the 1 January 2000 and the 31 December 2022.
- They have residency and GP registration data available over the same period of time.
- They have a recorded sex at birth (male/female) in either ADBE or WDSD.
- They are less than 105 years of age on 1 January 2000.

The SMYC cohort is a subset of the SMC cohort, including only the following:

- Individuals born between 1 January 2000 and 31 December 2021 (this allows for at least 1 year of follow-up after entering the cohort).
- Individuals with both demographic and healthcare data available *before* they turn 18 years of age.
- Individuals with *consistent* maternal records (an individual has a *consistent* maternal record if they can be linked to *at most* one mother, see ‘Maternal records study to identify SMYC’ section).

The cohort entry date is defined as the date an individual enters the cohort and is identified by the first date the individual is registered in WDSD.

Cohort censorship was defined by the earliest of:

- Death.
- Migration outside of Wales or break in their residency data.
- End of follow-up on 31 December 2022.

Note that once an individual meets one of the censorship criteria, they are not allowed to re-enter the e-cohort, see online supplemental appendix SA for further details.

To identify all relevant health events recorded in the routinely collected EHR data, we linked individuals in both cohorts to the available data sources. While primary care data are available for all cohort participants, as it is a prerequisite for cohort membership, secondary care and pathology data might not be available for everyone if they have not utilised these services during the study period. The upset plots in online supplemental figure S1 and S2 quantitatively represent individuals within the SMC and SMYC and their interactions with various healthcare settings throughout their cohort membership.

#### Maternal records study to identify SMYC

A necessary condition for being part of the SMYC is to have a consistent maternal record (MAT_ALF) within the National Community Child Health Database (NCCH) and the Maternity indicators data sources (MIDS).

An individual has a consistent maternal record if he or she can be linked to *at most* one mother, that is, if:

1. The individual has *no* MAT_ALF in either NCCH or MIDS.
2. The individual has *one* MAT_ALF either in NCCH or MIDS.
3. The individual has *two* or *more* MAT_ALF in both NCCH and MIDS, *and* they match each other.

When none of the above conditions apply, then the individual is excluded from the SMYC.

Note that if a person has an available maternal record but the mother is *not* included in SMC, this individual is included in SMYC however we considered this individual as *not* having a maternal record (n=1248, 0.1%). Thus, all the MAT_ALF linked to the SMYC form a subset of the SMC.

After selecting individuals from SMC who meet the SMYC conditions stated above (896 763), we extracted the maternal record(s) available in multiple data classes within NCCH and MIDS (NCCH has two data classes which include maternal record: NCCH_CHILD_BIRTHS and NCCH_CHILD_TRUST): 809 616 (90.3%) individuals have at least 1 maternal record, with 223 310 (24.9%) of them having records in both NCCH and MIDS; 608 (<0.001%) among these are linked to more than one MAT_ALF, that is, have inconsistent maternal records. These individuals are excluded from the SMYC cohort.

### Concept curation pipeline

MELD-B has defined a set of burdensomeness concepts, which can be identified and characterised in routinely collected EHR data (if these concepts are not available in routine data, proxy or derived phenotypes will be derived) to better understand how living with MLTC-M affects people’s lives and to apply this knowledge to inform data curation and extraction.

**Figure 2:**
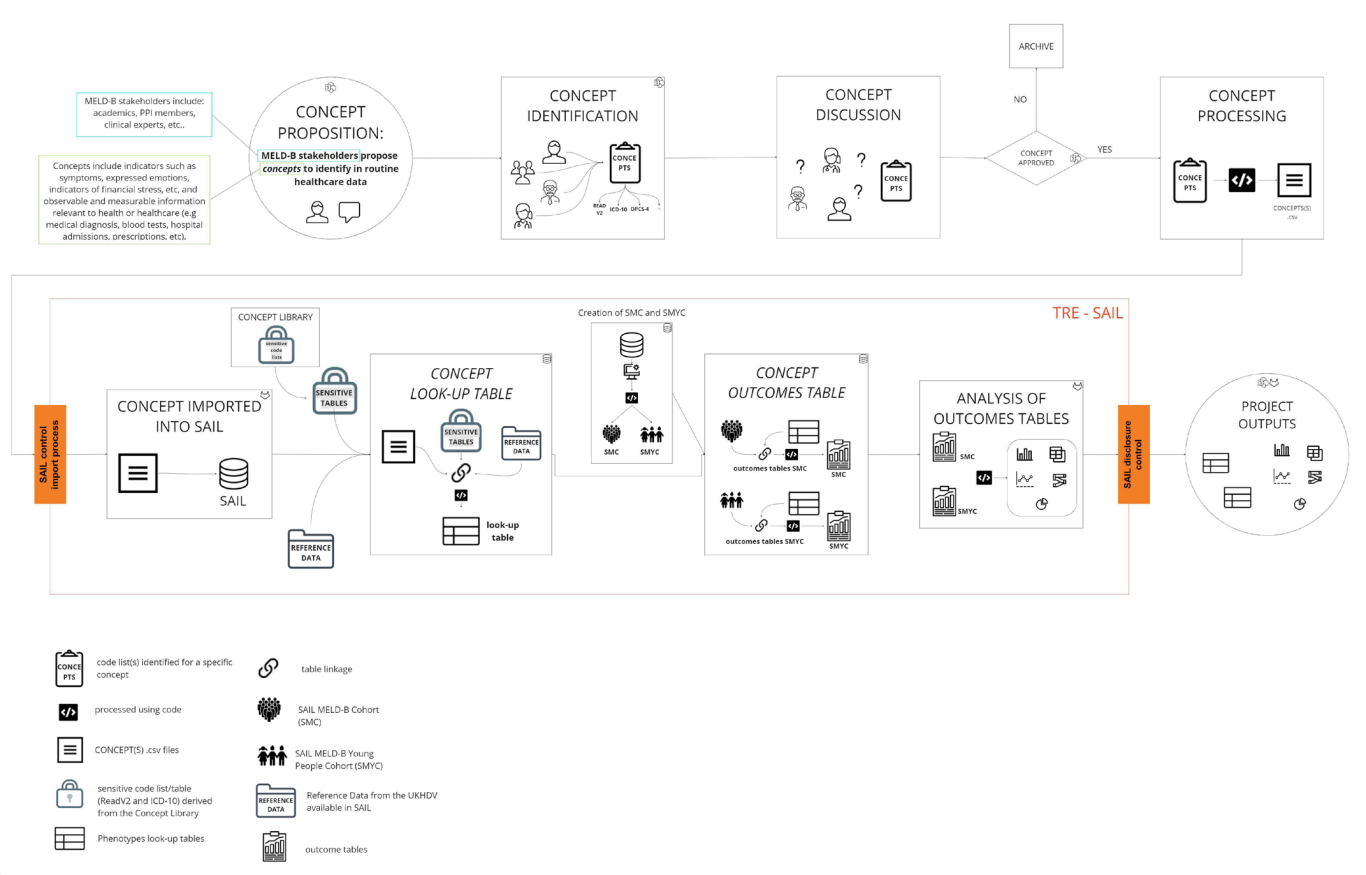
Concept curation pipeline to extract relevant data for SMC and SMYC. SMC, SAIL MELD-B e-cohort; SMYC, SAIL MELD-B children and Young adult e-cohort.

We implemented a reproducible concept curation pipeline to define, approve, process and import the identified burdensomeness concepts inside SAIL.This facilitates the extraction of relevant data associated with the various concepts identified by the MELD-B clinical group from the available linked data sources in SAIL. The current list of burdensomeness concepts developed up to this point is discussed in ‘MELD-B initial set of MLTC-M concepts’ and ‘SMC and SMYC concept curation pipeline’ sections. The pipeline and its outcomes are documented and managed outside of SAIL. It is accessible to all team members to ensure transparency of the process and facilitate collaboration. In figure 2 we provide a visualisation of the pipeline.

The first step of the pipeline is the proposition of each concept: clinicians, with a deep understanding of the phenomena of burden and their representation in routine data, can propose a concept that they believe embodies the idea of burden.

For the proposed concept to be considered and approved, the proposal must be accompanied by a published or open-source code list to review and agree on or a list of requirements that defines the concept, which can be used to derive a code list. A code list is a collection of classification codes associated with a specific concept of interest. Each code list includes only codes associated with a specific classification (eg, SNOMED, Read V2, ICD-10 and OPCS-4); therefore, it is possible to identify one or more code lists for each concept.

All proposed concepts and their associated code lists are then reviewed and discussed by the clinical group and, if approved, included in the study. If the concept proposed is not deemed relevant, it is archived. The approved concepts and their associated code lists are first processed to ensure their formatting, structure and content are available in a machine-readable output file, and then processed into a standardised format.^54^ Subsequently, the output code list can be utilised with routinely collected data sources and imported into the SAIL Databank (a controlled process is in place for importing files into SAIL to ensure they comply with SAIL policies and processes. Any files brought into SAIL must be within the scope of the project and approved by the IGRP since they could, directly or indirectly, impact the privacy protection of any data held within the TRE).

As part of the import and implementation process of the pipeline, all concepts and their associated code lists are cross-referenced with the SAIL sensitive code lists. These are codes that relate to certain protected treatment or diagnoses (the current set of sensitive codes covers: miscarriage, HIV/AIDS, pregnancy termination and sexually transmitted diseases. The sensitive code lists are processed as part of the pipeline) and whose use is restricted by NHS Wales. The current lists of all known sensitive ICD-10 and ReadV2 codes, which are based on a combination of sensitive codes provided by DHCW, a published list of sensitive codes for England and any other code flagged as sensitive by the SAIL team, are downloaded from the Concept Library^55^ and processed as part of the pipeline. Any sensitive codes are excluded from the concepts, so they will not be used or included for data extraction or used in project outputs.

Linking the imported concepts’ code lists with the sensitive code tables and the relevant tables available in SAIL (these tables are derived by the UK Health dimensions Database, which groups reference data for coding information),we create *look-up* tables for each concept and each concept coding classification, see online supplemental material 2 for an example. Linking the look-up tables to SMC and SMYC and the relevant data sources available in SAIL, we extract the relevant records for all the individuals in the cohort in *outcome tables*. Each of these tables contains *all* available records for a specific concept and each applicable concept-specific coding classification for *all* the individuals in SMC and SMYC. The outcome tables can then be used for analysis.

The pipeline allows us to rerun concepts efficiently and reproducibly, facilitating the data extraction and the data linkage with SMC and SMYC. In particular, this involves creating newer versions of look-up and outcomes tables with version control when existing concepts are updated, new concepts are provided and approved, and new and updated versions of the routinely collected data are available. Given the broad applicability of this pipeline, this approach can be shared with other projects, both in SAIL and in other TREs, as a transferable and reproducible method to be implemented.

#### MELD-B initial set of MLTC-M concepts

The MELD-B clinical domain expert group proposed an initial set of LTCs based on the list agreed on by the NIHR AIM Research Consortia,^56^ existing literature and project requirements,^57^,^60^ which will be used for clustering and sequencing analysis. The initial set of concepts proposed by the group consists of 83 LTCs, and the full list can be found in online supplemental table 2 (note that, as some concepts are derived from more sources, they appear only one in the final list).

The identification of burden concepts for inclusion in the study is an ongoing process. The clinical team can continuously propose additional concepts which, once reviewed, are processed through the concept pipeline. However, the identification of some burden concepts may be constrained by the limitations in capturing lived experience observations within the available EHR data. Every time the burden concepts’ list changes, a new concepts version is released in reference.^61^ The version of the concepts used as the basis for the descriptive analysis in this paper, see ‘Findings to date’ section, is V.2.2.4.^61^

The ReadV2 and ICD-10 code lists associated with the initial 83 concepts proposed have been extracted from reference^60^ and the supplementary file provided by the authors in reference^59^ (each condition derived from reference^58^ has been mapped to a concept (or more than one) available in reference^60^ by the clinical group). The MELD-B clinical group reviewed and approved all 83 ReadV2 and 70 (84.3%) of the ICD-10 code lists identified for the 83 concepts. The total number of medical codes identified and approved is 7503: 5987 (81.7%) ReadV2 and 1516 (18.3%) ICD-10 codes; 109 ReadV2 and 16 ICD-10 codes are flagged as sensitive and are therefore not included in the outcome tables created in SAIL or any of the descriptive analyses performed, see ‘Findings to date’ section.

The look-up tables created in the first stage of the MELD-B project are available in online supplemental material 2.

## Findings to date

### Sociodemographic characteristics

The SMC and SMYC e-cohorts have been designed to provide a generalisable population sample to be used to answer different research questions. From the 5 475 154 individuals available longitudinally within the WDSD, 5 180 602 individuals met the SMC inclusion criteria described in ‘Cohorts design’ section, and 896 155 individuals met the inclusion criteria to be included in the SMYC, see figure 1.

The follow-up period is defined as the time an individual spends in the e-cohort, with a minimum follow-up of 1 day and a maximum of 23 years. The number of individuals with full coverage is 1 731 280 (33.4%) for SMC and 47 500 (5.3%) (we considered that there is a delay between an individual’s WOB and his GP and its registration. The average delay is 23 days, but here we considered a delay of 30 days to be more inclusive) for SMYC, see table 1.

**Table 1:** SMC and SMYC baseline demographic information

Baseline demographics	SMC	SMYC
Cohort size	**5 180 602** (**100%**)	**896 155** (**100%**)
Male (%)	2 575 867 (49.7%)	459 644 (51.3%)
Female (%)	2 604 735 (50.3%)	436 511 (48.7%)
Cohort size at cohort start	2 990 123 (57.8%)	N/A
Mean age in years at cohort start	39.7 years	N/A
Cohort exit reason		
Death	747 927 (14.4%)	1614 (0.2%)
Loss to follow-up	1 420 930 (27.4%)	143 836 (16.1%)
End of follow-up	3 011 745 (58.1%)	750 705 (83.7%)
Ethnic group (NER code)		
1. White	3 654 965 (70.6%)	715 839 (79.9%)
2. Mixed	55 150 (1.1%)	27 434 (3.1%)
3. Indian	37 543 (0.7%)	8525 (1.0%)
4. Pakistani	20 404 (0.4%)	6022 (0.7%)
5. Bangladeshi	107 146 (2.1%)	28 849 (3.2%)
6. Chinese	29 316 (0.6%)	2549 (0.3%)
7. Black Caribbean	7803 (0.2%)	914 (0.1%)
8. Black African	28 372 (0.5%)	8932 (1.0%)
9. Other	99 677 (1.9%)	26 252 (2.9%)
10. Unknown ethnicity	1 140 226 (22%)	70 839 (7.8%)
Follow-up period		
≤1 year	354 157 (6.8%)	72 301 (8.1%)
1–3 years	670 798 (12.9%)	115 148 (12.8%)
3–5 years	387 791 (7.5%)	90 087 (10.1%)
5–10 years	663 472 (12.8%)	200 183 (22.3%)
10–15 years	567 058 (10.9%)	183 958 (20.5%)
15–20 years	532 310 (10.3%)	168 030 (18.7%)
≥20 years	2 005 016 (38.7%)	66 448 (7.4%)

The percentage refers to the total number of individuals in each cohort. Note that WIMDs are not available for SMYC as they are computed on 1 January 2000, and SMYC only includes individuals born on or after this date.

N/Anot applicableNERNew and Emerging RespiratorySMCSAIL MELD-B e-cohortSMYCSAIL MELD-B children and Young adult e-cohortWIMDWelsh Index of Multiple Deprivation

Ethnic group records are available for 78% of the individuals in SMC and 92.2% of individuals in SMYC. In both cohorts, the predominant ethnicities are ‘White’ followed by ‘Bangladeshi’ and ‘Mixed’

In table 1, we summarise the demographic information for SMC and SMYC.

**Figure 3:**
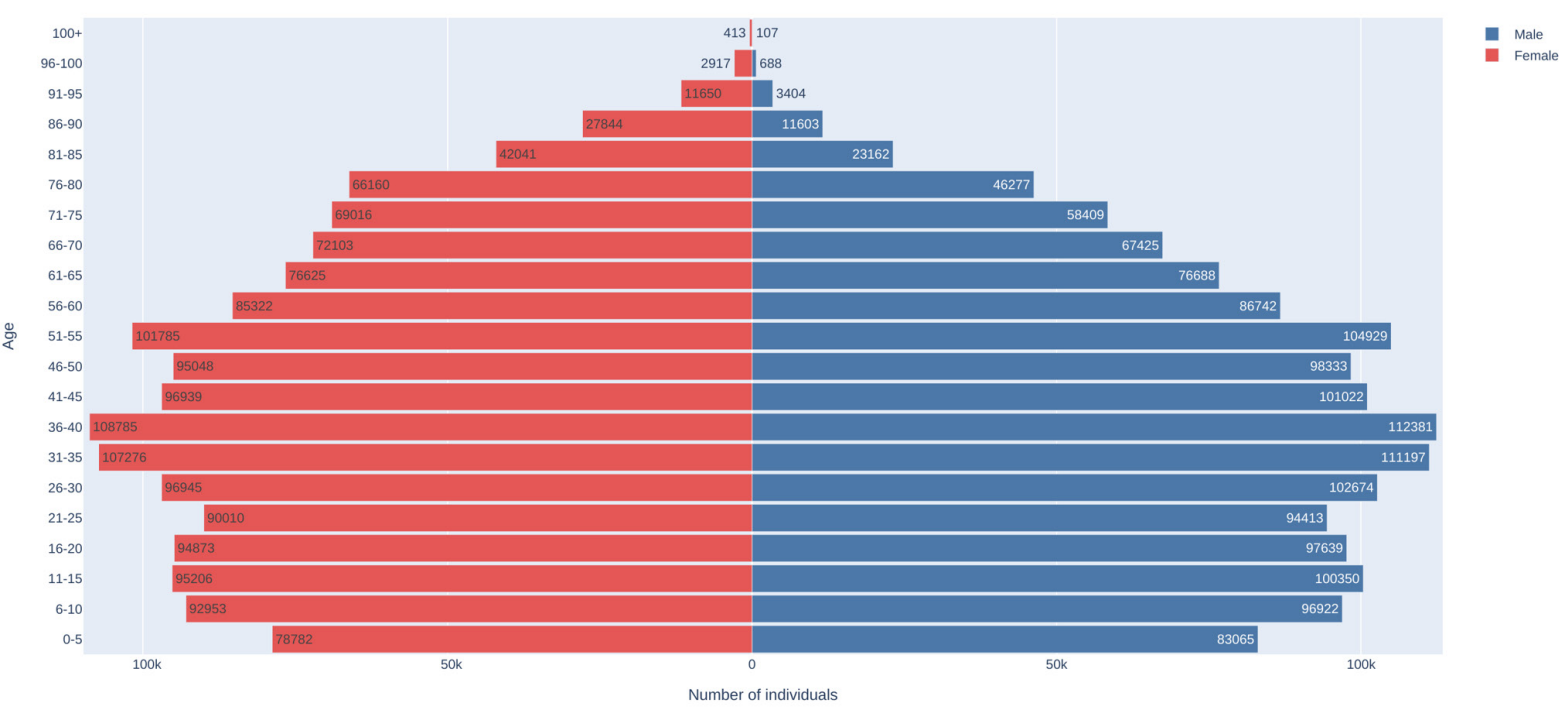
Pyramid plot of SMC at cohort start date. SMC, SAIL MELD-B e-cohort.

The distribution of SMC at cohort inception by age groups and sex is visualised in figure 3.

To provide a quantitative representation of individuals within the cohorts and their interactions with various healthcare settings throughout the cohort membership, we produced the Upset plots in online supplemental figure S1 and S2. Almost 37% of the individuals in the SMC have records in all the routinely collected EHR data sources available to the MELD-B project. In total, 66.4% and 67.9% of individuals used inpatient and outpatient services, respectively, while only 48% of SMC used emergency department services.

The SMYC Upset plot includes children-specific data sources in addition to the routinely collected EHR data sources. Almost every individual (98%) has at least one record in the NCCH data source, see also ‘Maternal records study to identify SMYC’ section 2.2.1, and 59.9%, 64.5% and 58.7% of individuals can be linked to inpatient secondary care, outpatient secondary care and emergency data sources, respectively.

#### Cohorts evolution over the cohort period

To better understand how SMC and SMYC evolved over the study period, we collected demographic information on 1 January of every year during the cohort study period (eg, total number of individuals, sex ratio, number of individuals leaving and/or joining the e-cohort, etc), see online supplemental table S3 and S4 for more details (for the SMYC, we collect information starting on 1 January 2001).

The total number of individuals in the SMC increases until 2010, when the e-cohort includes 3 089 310 individuals. It decreases after this year and reaches its new minimum in 2022 (3 013 498). The female/male ratio steadily reduces from 2000 to 2017, reaching a minimum value of 0.995, and then increases again from 2016 to 2022. In absolute terms, women outnumber men from 2000 to 2011. The number of individuals in SMYC increases over the cohort period, reaching its maximum in 2022 with 720 500 people. Differently from SMC, the ratio of female/male is always less than one, see online supplemental figure S3.

**Figure 4:**
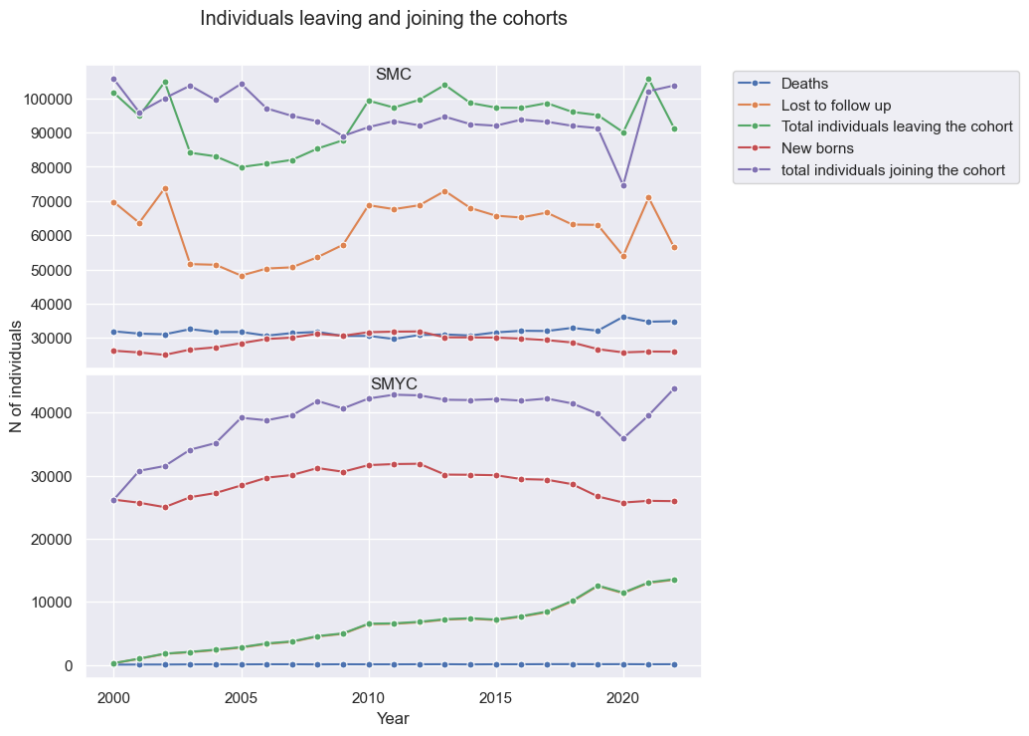
Individuals leaving and joining SMC and SMYC each year. Note that in the SMYC plot, the ‘Lost to follow-up’ line almost coincides with the ‘total individuals leaving the cohort’ line. SMC, SAIL MELD-B e-cohort; SMYC, SAIL MELD-B children and Young adult e-cohort.

Between 2002 and 2009, the number of individuals joining SMC is larger than the number of people leaving it, see figure 4. This trend is reversed from 2010 to 2022. The year with the biggest gap between cohort joiners and cohort leavers is 2005 (104 258 vs 79 923). The year with the smallest number of joiners is 2020 (74 609). This dip is likely attributed to the impact of the COVID-19 pandemic and the resulting decline in the university student population.

In SMYC, the number of individuals joining the cohort is always larger than the number of individuals leaving it, see figure 4. The year with the smallest gap between cohort joiners and cohort leavers is 2020 (35 890 joiners vs 11 440 leavers), where it is possible to see a clear decrease in the number of young individuals registering as Welsh residents compared with the previous years. However, in 2021 and 2022, this number increases again, returning to pre-2020 values.

For SMC, the average number of deaths every year during the e-cohort study accounts for 30%–40% of people leaving the cohort each year. The newborn accounts for 24%–34% of people joining SMC. For SMYC, the average number of deaths per year during is 73 (1%–2% of total leavers). Between 2005 and 2020, newborns account for 70%–75% of individuals joining the cohort, while in 2021 and 2022, they account for 65% and 59%, respectively.

**Figure 5:**
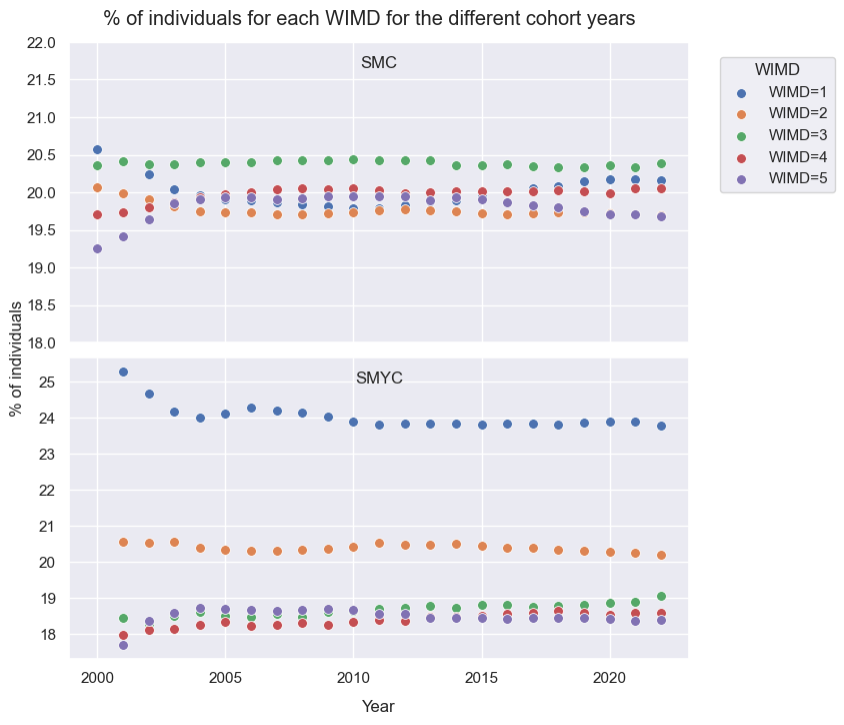
Percentages of individuals residing in LSOA with WIMD=1,2,3,4 or 5 on 1 January of every year during the cohort study period. LSOA, Lower-layer Super Output Area; SMC, SAIL MELD-B e-cohort; SMYC, SAIL MELD-B children and Young adult e-cohort; WIMD, Welsh Index of Multiple Deprivation.

Within SMC, there is a noticeable decline in the percentage of people residing in areas with the lowest WIMD quintile between 2000 and 2005, as a growing number of individuals relocate to less deprived LSOAs (WIMD=4, WIMD=5). However, post 2014, there is a noticeable uptick in the population residing in more deprived areas (WIMD=1) and a consequential decrease of those living in less deprived areas (WIMD=5 and WIMD=3), see figure 5.

The majority of individuals in SMYC, between 24% and 26%, reside in an area with the lowest WIMD quintile (WIMD=1) and approximately 20% of individuals reside in an area with WIMD=2. These percentages remain consistent during the cohort period.

### SMC and SMYC concept curation pipeline

**Figure 6:**
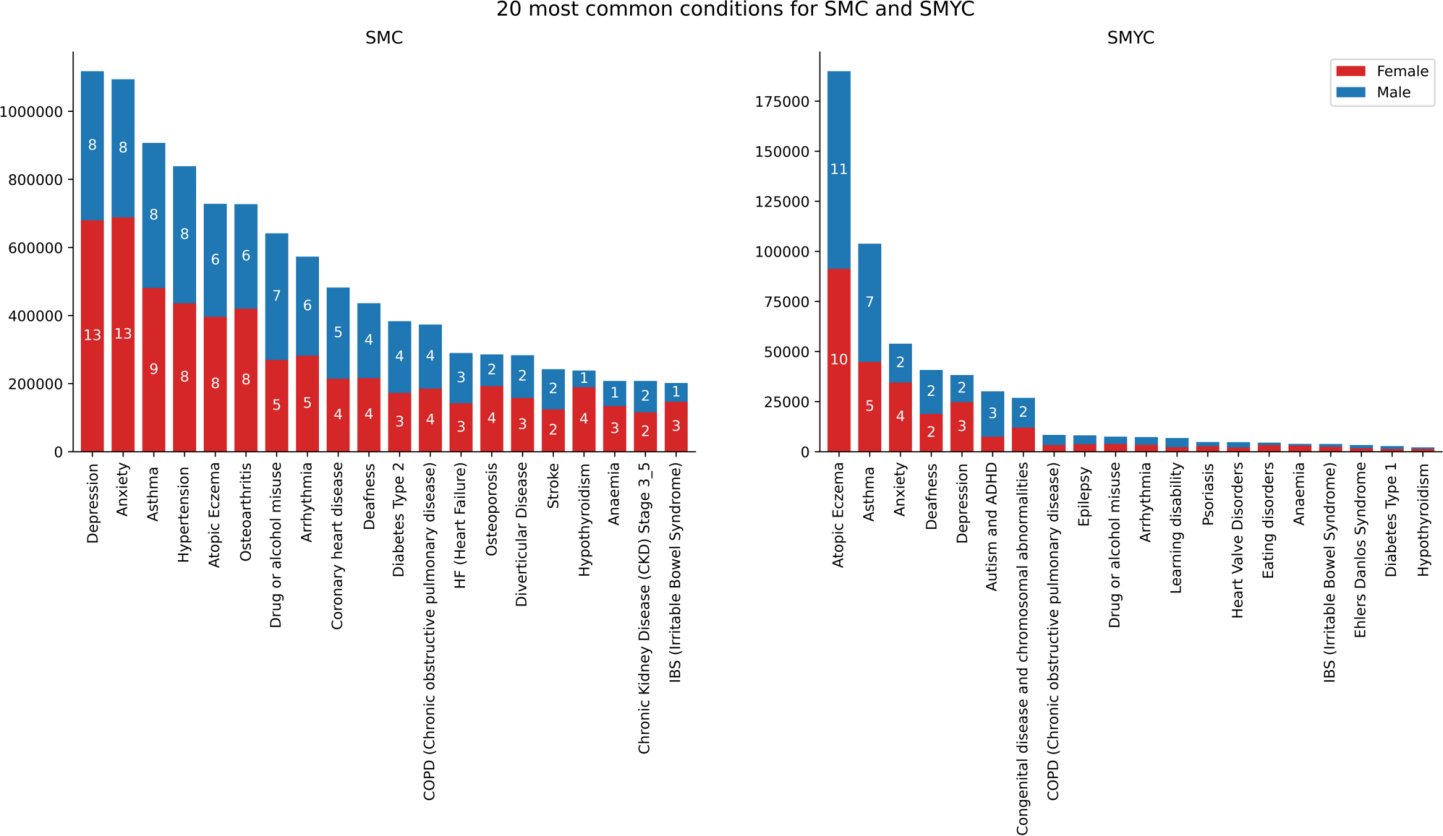
20 most common concepts for SMC and SMYC. The number on the bars represents the % of individuals for each sex with records of each concept compared with the complete SMC. SMC, SAIL MELD-B e-cohort; SMYC, SAIL MELD-B children and Young adult e-cohort.

From the outcomes tables, created through the concept curation pipeline by linking SMC and SMYC to the look-up tables and the relevant data source inside SAIL, it is possible to extract descriptive analysis for all the concepts identified. Considering both primary and secondary care data (WLGP and PEDW), in figure 6, we present the 20 most common condition concepts identified in SMC and SMYC. The five most common conditions for individuals in SMC are depression, anxiety asthma, hypertension and atopic eczema. Notably, females are subject to a substantially higher incidence of anxiety and depression compared with males, with prevalence rates of 26.4% vs 16.6% and 26.1% vs 17.0%.

The five most common conditions for individuals in SMYC are atopic eczema, asthma, anxiety, deafness and depression. Similarly, within the young cohort, females demonstrate a higher prevalence of anxiety (7.5%) and depression (5.4%) compared with males (4.4% and 3.1%). However, for males, the prevalence of autism and ADHD is notably higher compared with females, with rates of 5.2% and 1.6%, respectively.

Looking at the most common 20 concepts for SMC and their age onset, it is clear that certain concepts have distinct patterns of onset across different age groups. Atopic eczema and asthma show a significant proportion of initial diagnoses occurring between the ages of 1 and 10, which account for 35.3% and 23.3% of the total diagnoses, respectively. Depression, anxiety and IBS typically begin to be diagnosed during the teenage years, peaking between the ages of 20 and 30. The first records of concepts, such as hypertension, diabetes type 2, coronary heart disease, osteoporosis etc, are more frequently expected in older individuals, and are typically recorded in individuals after the age of 50, see online supplemental figure S4.

A similar analysis for the most common 20 concepts in the SMYC indicates that atopic eczema, deafness, chronic obstructive pulmonary disease, epilepsy, congenital disease and chromosomal abnormalities, arrhythmia and heart valve disorders peak between the ages of 0 and 4. Autism and ADHD and learning disabilities are mostly recorded for children in primary school age (age 4–11), while anxiety, depression and IBS have a higher incidence rate in teenagers, from the age of 16 years onwards, see online supplemental figure S5.

For a more complete and detailed analysis for each concept included in the study, see online supplemental table S5.

### Main strengths and limitations

The main strengths of the two prospective longitudinal e-cohorts we built is the nationwide coverage of the individuals, making these e-cohorts representative of the comprehensive Welsh population over 22 years of coverage. Having the possibility to link these individuals to demographic, primary and secondary healthcare data facilitates and supports a wide range of approaches to address research questions and deliverables for the MELD-B project and future research. Moreover, the utilisation of anonymised e-cohorts serves as an effective strategy for overcoming consent-related barriers, enabling seamless data aggregation and analysis.

In this paper, we also defined a reproducible concept curation pipeline to manage and process data extraction for the e-cohorts. This pipeline ensures that whenever there is a new data release, updates to the cohorts or modifications to the concepts or their code lists, the relevant tables containing data for the cohorts can be immediately updated, provenance of changes tracked and new data set versions published. This enables support for multiple research questions and outcomes across the range of data analysis in SAIL. Moreover, the adaptability of this pipeline makes it a reusable tool for data preparation or initial data analysis in other research projects.

While similar cohorts have been developed for multimorbidity research,^62^ our e-cohorts are unique in their approach. Most existing studies create bespoke cohorts that follow specific inclusion criteria, and they rarely share the codes or methodologies used to define these cohorts. In contrast, we provide comprehensive documentation, along with all the code and methods used to establish the cohort creation and analysis, which enhances transparency and reproducibility.

The use of routinely collected EHR data in cohort studies is limited as it presents a number of challenges: (1) routinely collected EHR data are primarily collected for clinical and administrative purposes rather than supporting research, therefore they might lack observations relate to lived experiences, (2) there is often incomplete and/or inaccurate data, which may not be harmonised and standardised across data sources. However, the MELD-B project recognises the importance of using large-scale EHR data sources, such as the Secure Anonymised Information Linkage (SAIL) Databank and the Clinical Practice Research Datalink (CPRD). These data sources offer large sample sizes, long follow-up periods and include a wide range of study variables and generalisable populations.

The use of EHR data in cohort studies is limited by missing data and errors in routine records. In addition, EHRs often lack observations related to lived experiences, which are important considerations in multimorbidity studies.

### Collaboration

The data used to create SMC and SMYC are available in the SAIL Databank at Swansea University, Swansea, UK, but as restrictions apply, they are not publicly available. All proposals to use SAIL data are subject to review by an independent Information Governance Review Panel (IGRP). All the codes and scripts used to implement the concept curation pipeline are available in reference.^54^

The MELD-B consortium welcomes input from external investigators regarding research proposals or opportunities for collaboration.

## supplementary material

10.1136/bmjopen-2024-087946 online supplemental file 1

10.1136/bmjopen-2024-087946 online supplemental file 2

## Data Availability

Data may be obtained from a third party and are not publicly available.

## References

[1] Fortin M, Haggerty J, Almirall J (2014). Lifestyle factors and multimorbidity: a cross sectional study. BMC Public Health.

[2] Kingston A, Robinson L, Booth H (2018). Projections of multi-morbidity in the older population in England to 2035: estimates from the Population Ageing and Care Simulation (PACSim) model. Age Ageing.

[3] Lawson KD, Mercer SW, Wyke S (2013). Double trouble: the impact of multimorbidity and deprivation on preference-weighted health related quality of life a cross sectional analysis of the Scottish Health Survey. Int J Equity Health.

[4] Berntsen G, Høyem A, Lettrem I (2018). A person-centered integrated care quality framework, based on a qualitative study of patients’ evaluation of care in light of chronic care ideals. BMC Health Serv Res.

[5] Salisbury C, Johnson L, Purdy S (2011). Epidemiology and impact of multimorbidity in primary care: a retrospective cohort study. Br J Gen Pract.

[6] Feng X, Tan X, Riley B (2018). Polypharmacy and Multimorbidity Among Medicaid Enrollees: A Multistate Analysis. Popul Health Manag.

[7] Khezrian M, McNeil CJ, Murray AD (2020). An overview of prevalence, determinants and health outcomes of polypharmacy. Ther Adv Drug Saf.

[8] Cassell A, Edwards D, Harshfield A (2018). The epidemiology of multimorbidity in primary care: a retrospective cohort study. Br J Gen Pract.

[9] Koller D, Schön G, Schäfer I (2014). Multimorbidity and long-term care dependency - A five-year follow-up. BMC Geriatr.

[10] Aubert CE, Schnipper JL, Fankhauser N (2020). Association of patterns of multimorbidity with length of stay: A multinational observational study. Medicine (Baltimore).

[11] Eto F, Samuel M, Henkin R (2023). Ethnic differences in early onset multimorbidity and associations with health service use, long-term prescribing, years of life lost, and mortality: A cross-sectional study using clustering in the UK Clinical Practice Research Datalink. PLoS Med.

[12] Lehnert T, Heider D, Leicht H (2011). Review: health care utilization and costs of elderly persons with multiple chronic conditions. Med Care Res Rev.

[13] Glynn LG, Valderas JM, Healy P (2011). The prevalence of multimorbidity in primary care and its effect on health care utilization and cost. Fam Pract.

[14] Palladino R, Tayu Lee J, Ashworth M (2016). Associations between multimorbidity, healthcare utilisation and health status: evidence from 16 European countries. Age Ageing.

[15] Bähler C, Huber CA, Brüngger B (2015). Multimorbidity, health care utilization and costs in an elderly community-dwelling population: a claims data based observational study. BMC Health Serv Res.

[16] MacMahon S (2018). Multiple long-term conditions (multimorbidity): a priority for global health research.

[17] Xu X, Mishra GD, Jones M (2017). Mapping the global research landscape and knowledge gaps on multimorbidity: a bibliometric study. J Glob Health.

[18] Ho I-S, Azcoaga-Lorenzo A, Akbari A (2021). Examining variation in the measurement of multimorbidity in research: a systematic review of 566 studies. Lancet Public Health.

[19] Prazeres F, Santiago L (2015). Prevalence of multimorbidity in the adult population attending primary care in Portugal: a cross-sectional study. BMJ Open.

[20] Foguet-Boreu Q, Violán C, Rodriguez-Blanco T (2015). Multimorbidity Patterns in Elderly Primary Health Care Patients in a South Mediterranean European Region: A Cluster Analysis. PLoS One.

[21] Prados-Torres A, Calderón-Larrañaga A, Hancco-Saavedra J (2014). Multimorbidity patterns: a systematic review. J Clin Epidemiol.

[22] Garin N, Koyanagi A, Chatterji S (2016). Global Multimorbidity Patterns: A Cross-Sectional, Population-Based, Multi-Country Study. *GERONA*.

[23] Goodman RA, Ling SM, Briss PA (2016). Multimorbidity Patterns in the United States: Implications for Research and Clinical Practice. *GERONA*.

[24] Kirchberger I, Meisinger C, Heier M (2012). Patterns of multimorbidity in the aged population. Results from the KORA-Age study. PLoS One.

[25] Lyons J, Akbari A, Abrams KR (2023). Trajectories in chronic disease accrual and mortality across the lifespan in Wales, UK (2005-2019), by area deprivation profile: linked electronic health records cohort study on 965,905 individuals. *Lancet Reg Health Eur*.

[26] France EF, Wyke S, Gunn JM (2012). Multimorbidity in primary care: a systematic review of prospective cohort studies. Br J Gen Pract.

[27] Ashworth M, Durbaba S, Whitney D (2019). Journey to multimorbidity: longitudinal analysis exploring cardiovascular risk factors and sociodemographic determinants in an urban setting. BMJ Open.

[28] López-Bueno R, Feng Z, Ortega-Martín E (2023). Social determinants of multimorbidity patterns: A systematic review.

[29] Xu X, Mishra GD, Dobson AJ (2018). Progression of diabetes, heart disease, and stroke multimorbidity in middle-aged women: A 20-year cohort study. PLoS Med.

[30] Ruel G, Lévesque J-F, Stocks N (2014). Understanding the Evolution of Multimorbidity: Evidences from the North West Adelaide Health Longitudinal Study (NWAHS). PLoS ONE.

[31] Stannard S, Holland E, Crozier SR (2022). Early-onset burdensome multimorbidity: an exploratory analysis of sentinel conditions, condition accrual sequence and duration of three long-term conditions using the 1970 British Cohort Study. BMJ Open.

[32] Cezard G, McHale CT, Sullivan F (2021). Studying trajectories of multimorbidity: a systematic scoping review of longitudinal approaches and evidence. BMJ Open.

[33] Owen R, Lyons J, Akbari A (2022). Temporal sequencing in multimorbidity using population-scale linked data for 1.7 million individuals with 20-year follow-up. In Review.

[34] Head A, Fleming K, Kypridemos C (2021). Multimorbidity: the case for prevention. J Epidemiol Community Health.

[35] Fraser SD, Stannard S, Holland E (2023). Multidisciplinary ecosystem to study lifecourse determinants and prevention of early-onset burdensome multimorbidity (MELD-B) - protocol for a research collaboration. J Multimorb Comorb.

[36] Stannard S, Berrington A, Fraser SDS (2024). Mapping domains of early-life determinants of future multimorbidity across three uk longitudinal cohort studies. Pub Glob Health.

[37] Stannard S, Berrington A, Paranjothy S (2023). A conceptual framework for characterising lifecourse determinants of multiple long-term condition multimorbidity. *J Multimorb Comorb*.

[38] Owen RK, Lyons J, Akbari A (2023). Effect on life expectancy of temporal sequence in a multimorbidity cluster of psychosis, diabetes, and congestive heart failure among 1·7 million individuals in Wales with 20-year follow-up: a retrospective cohort study using linked data. Lancet Public Health.

[39] Gluckman PD, Buklijas T, Hanson MA (2016). The Developmental Origins of Health and Disease (DOHaD) Concept: Past, Present, and Future. The Epigenome and Dev Origins of Health and Disease.

[40] Humphreys J, Jameson K, Cooper C (2018). Early-life predictors of future multi-morbidity: results from the Hertfordshire Cohort. Age Ageing.

[41] Gondek D, Bann D, Brown M (2021). Prevalence and early-life determinants of mid-life multimorbidity: evidence from the 1970 British birth cohort. BMC Public Health.

[42] Wilding S, Ziauddeen N, Smith D (2020). Are environmental area characteristics at birth associated with overweight and obesity in school-aged children? Findings from the SLOPE (Studying Lifecourse Obesity PrEdictors) population-based cohort in the south of England. BMC Med.

[43] Fleming TP, Watkins AJ, Velazquez MA (2018). Origins of lifetime health around the time of conception: causes and consequences. The Lancet.

[44] Home - sail databank. https://saildatabank.com/.

[45] Lyons RA, Jones KH, John G (2009). The SAIL databank: linking multiple health and social care datasets. BMC Med Inform Decis Mak.

[46] Ford DV, Jones KH, Verplancke J-P (2009). The SAIL Databank: building a national architecture for e-health research and evaluation. BMC Health Serv Res.

[47] Jones KH, Ford DV, Jones C (2014). A case study of the Secure Anonymous Information Linkage (SAIL) Gateway: a privacy-protecting remote access system for health-related research and evaluation. J Biomed Inform.

[48] ICO Information Commissioner’s Office (2022).

[49] Akbari A, Torabi F, Bedston S (2022). Developing a research ready population-scale linked data ethnicity-spine in wales. Pub Glob Health.

[50] Health data research innovation gateway. https://web.www.healthdatagateway.org/search?search=&datasetSort=latest&tab=Datasets.

[51] WLGP coverage reports - analytical services public - swansea university medical school confluence site. https://docs.hiru.swan.ac.uk/display/SATP/WLGP+coverage+reports.

[52] Abbasizanjani H, Torabi F, Bedston S (2023). Harmonising electronic health records for reproducible research: challenges, solutions and recommendations from a UK-wide COVID-19 research collaboration. BMC Med Inform Decis Mak.

[53] Welsh longitudinal general practice dataset (wlgp) - welsh primary care. https://web.www.healthdatagateway.org/dataset/33fc3ffd-aa4c-4a16-a32f-0c900aaea3d2.

[54] Dylag J, Chiovoloni R, Akbari A (2024). A tool for automating the curation of medical concepts derived from coding lists. https://git.soton.ac.uk/meld/meldb/concepts-processing.

[55] Concept library. https://conceptlibrary.saildatabank.com/.

[56] Dambha-Miller H, Farmer A, Nirantharakumar K (2023). Artificial Intelligence for Multiple Long-term conditions (AIM): A consensus statement from the NIHR AIM consortia.

[57] Barnett K, Mercer SW, Norbury M (2012). Epidemiology of multimorbidity and implications for health care, research, and medical education: a cross-sectional study. The Lancet.

[58] Ho ISS, Azcoaga-Lorenzo A, Akbari A (2022). Measuring multimorbidity in research: Delphi consensus study. *bmjmed*.

[59] Hanlon P, Jani BD, Nicholl B (2022). Associations between multimorbidity and adverse health outcomes in UK Biobank and the SAIL Databank: A comparison of longitudinal cohort studies. PLoS Med.

[60] GitHub - thinkinggroup/phenotypes. https://github.com/THINKINGGroup/phenotypes.

[61] (2024). MELD-b concepts release. https://git.soton.ac.uk/meldb/concepts/-/tree/v2.2.4.

[62] Lyons J, Akbari A, Agrawal U (2021). Protocol for the development of the Wales Multimorbidity e-Cohort (WMC): data sources and methods to construct a population-based research platform to investigate multimorbidity. BMJ Open.

