# Supplementary material for: Cohort profile: creation of the SAIL MELD-B e-cohort (SMC) and SAIL MELD-B children and young adult e-cohort (SMYC) to investigate the lived experience of the ‘burdensomeness’ of multimorbidity
Source: BMJ Open. 2025 Jan 7;15(1):e087946. doi: 10.1136/bmjopen-2024-087946 (PMC11792564; doi:10.1136/bmjopen-2024-087946)
Supplement: online supplemental file 1 [file bmjopen-15-1-s001.docx]

**Cohort profile for the creation of the SAIL MELD-B e-cohort (SMC) and SAIL MELD-B children and Young adult e-cohort (SMYC): Supplementary material**

**Contents**

**Supplementary Table 1:** Page 2-3

**Supplementary Appendix A:** Page 4

**Supplementary Figure 1**: Page 5

**Supplementary Figure 2**: Page 5

**Supplementary Figure 3**: Page 6

**Supplementary Figure 4**: Page 7

**Supplementary Figure 5**: Page 8

**Supplementary Table 2:** Page 9-12

**Supplementary Table 3**: Page 13-14

**Supplementary Table 4**: Page 15-16

**Supplementary Table 5**: Page 17-20

**Supplementary Table 1:** Data sources available to the MELD_B project**.**

| **Data source full name** | **Data source description** |
| --- | --- |
| Annual District Birth Extract (ADBE) | Register of all births relating to Welsh residents, including those born out of Wales and subsequently registered in Wales. |
| Annual District Death Extract (ADDE) | Register of all deaths relating to Welsh residents, including those that died out of Wales. |
| Census 2011 – Welsh Records (CENW) | Data collected during the UK census held on 27^th^ March 2011. |
| Education Wales (pre 16) (EDUW) | Schools and pupil data for Wales, which covers state funded learning centres. This describes learning centres, outcomes for learners, special educational needs (SEN), attendance summary (prior to 2020), and free school meals (FSM). |
| Emergency Department Dataset (EDDS) | Register of all attendances at Accident & Emergency departments and Minor Injury Units (MIUs) in Welsh hospitals. |
| Maternity indicators dataset (MIDS) | Data relating to the woman at initial assessment and to mother and baby (or babies) for all births in Wales. |
| Millenium Cohort Study Dataset (MCSD) | Survey data on the conditions of social, economic and health advantages and disadvantages facing children born at the start of the 21st Century. |
| National Community Child Health Database (NCCH) | Data including birth registration and monitoring of child health examinations and immunisations. |
| Outpatient Database for Wales (OPDW) | Attendance information for all NHS Wales hospital outpatient appointments. This dataset contains all scheduled outpatient appointments, including those where the patient failed to attend. |
| Patient Episode Database for Wales (PEDW) | Data on all inpatient and day case activity undertaken in NHS Wales plus data on Welsh residents treated in English Trusts. |
| Wales Results Reporting Service (WRRS) | Register of all results for pathology requests and any other associated results across all health boards in Wales, from both primary and secondary care. |
| Welsh Demographic Service Dataset (WDSD) | Register of all individuals registered with a Welsh GP, including individuals anonymised addresses and practice history. |
| Welsh Longitudinal General Practice data (WLGP) | Attendance and clinical information for all general practice interactions: includes patient’s symptoms, investigations, diagnoses, prescribed medication and referrals to tertiary care. |

**Supplementary Appendix A:** How to treat lost to follow-up individuals in SMC and SMYC.

*Loss to follow-up* is one of the cohort censorship criteria adopted in this study. An individual is considered *lost to follow up* if their participation in the study is discontinued, which in the specific case of the MELD-B project, means that the participant moves outside of Wales. Although it is possible for an individual to re-join the cohort if they return to Wales, the MELD-B has taken a collective decision not to allow this situation following an analysis performed on the WDSD.

From WDSD we calculated that 352,550 (6.8%) individuals in SMC have a break in their residence data as they leave Wales before returning to the country after a period of at least 30 days. They account for the 24.8% of the total number of individuals censored because lost to follow up. Referring to *N periods* as the number of time an individual enters the cohort, assuming we have allowed people to re-join once they return to Wales, in the following table we present the number of individuals that join the cohort one, two, three and four or more times:

| **N periods** | **N individuals** |
| --- | --- |
| **1** | 4,828,060 (93.2%) |
| **2** | 323,630 (6.3%) |
| **3** | 25,860 (0.5%) |
| **4+** | 3,060 (<0.1%) |

Most individuals (93.2%) join the cohort only once: once they are included in the cohort they die, move permanently outside Wales or remain in the country until the end of the study period. The vast majority of individuals with a break in their residency data leave and return to Wales only once (N = 2), and only the 0.6% of SMC have more than one break in their residency data. This result suggests to consider individuals with residency breaks as lost to follow up on the date they firstly move outside of Wales and to *not allow them to be re-included in the cohort.*

**Supplementary Figure 1:** Number of SMC individuals using healthcare services recorded in multiple datasources.

**
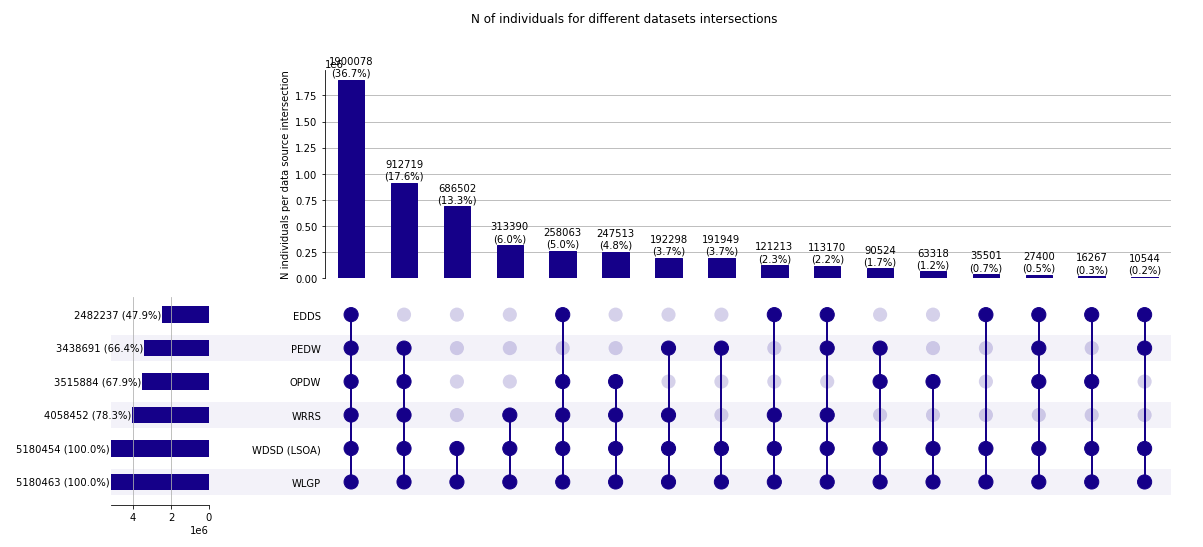
**

**Supplementary Figure 2:** Number of SMYC individuals using healthcare services recorded in multiple datasources.

**
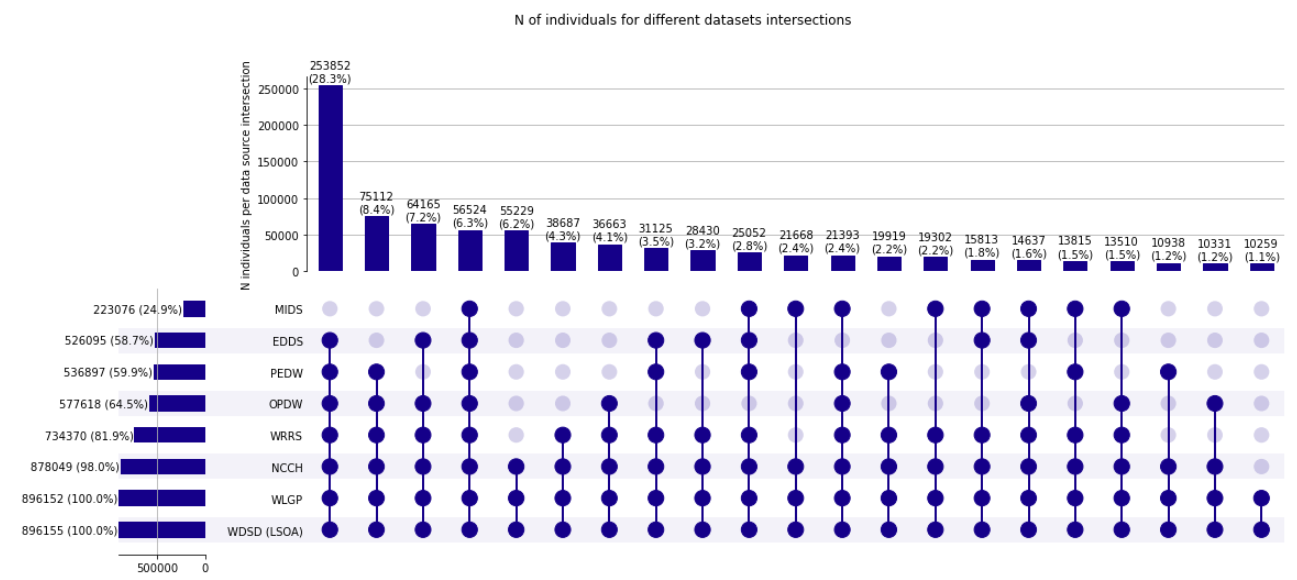
**

**Supplementary Figure 3:** On the left total number of individuals on the 1st January of each year during the study period. On the right, the number of females/males on the 1st January of every year during the study period for SMC and SMYC


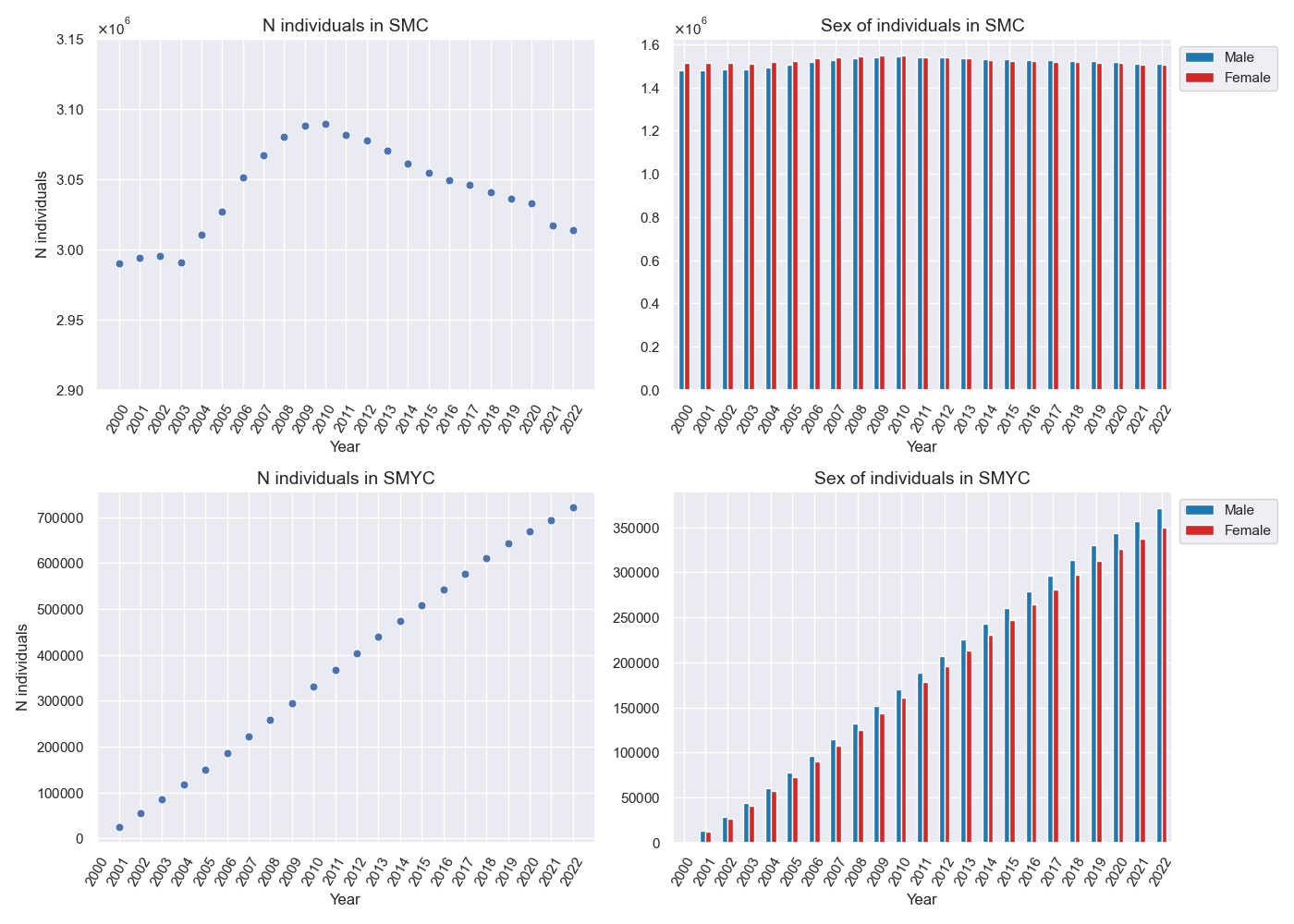


**Supplementary Figure 4:** Number of individuals for each age onset group for each of the 20 most common concepts in SMC.


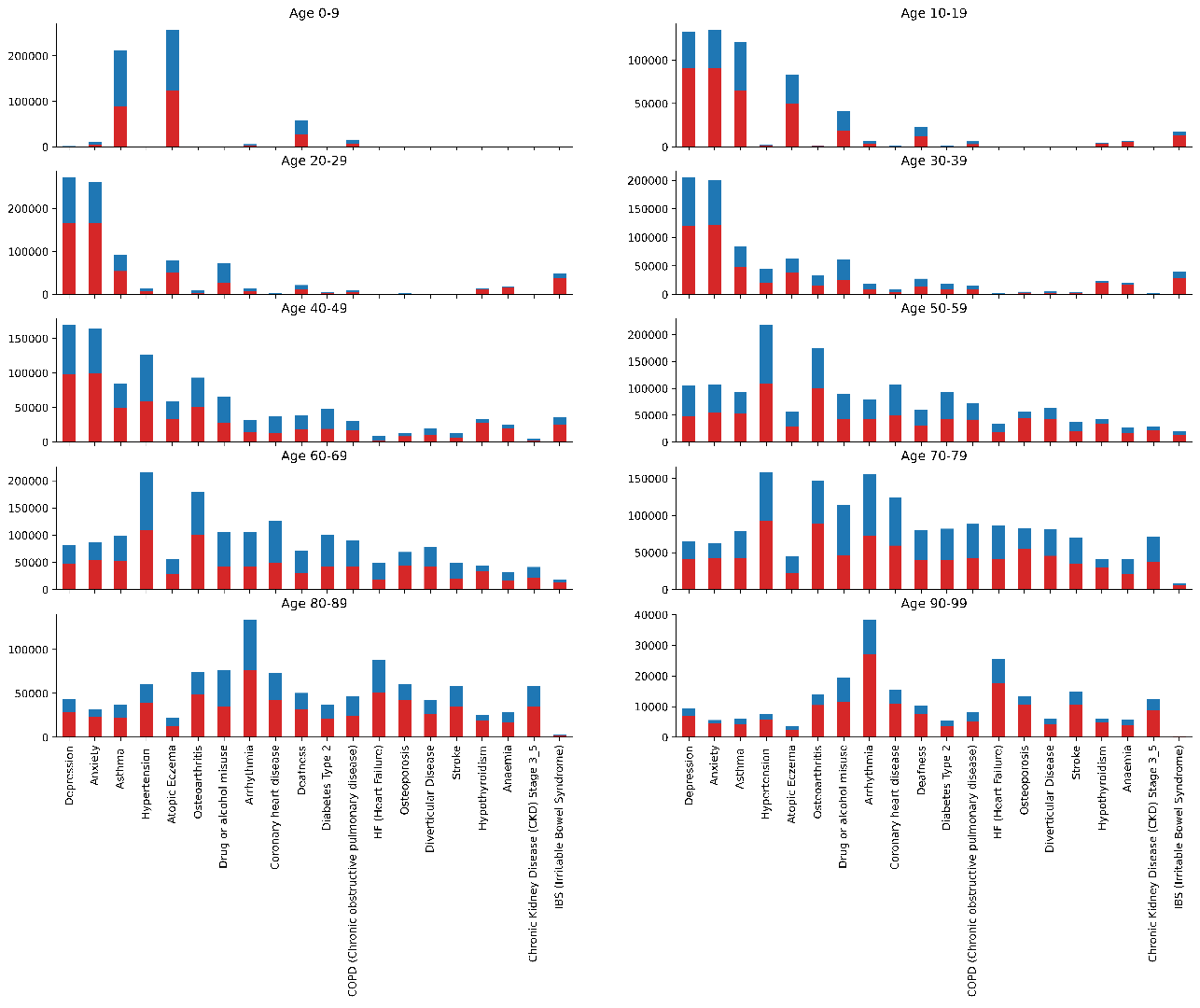


**Supplementary Figure 5:** Number of individuals for each age onset group for each of the 20 most common concepts in SMYC.


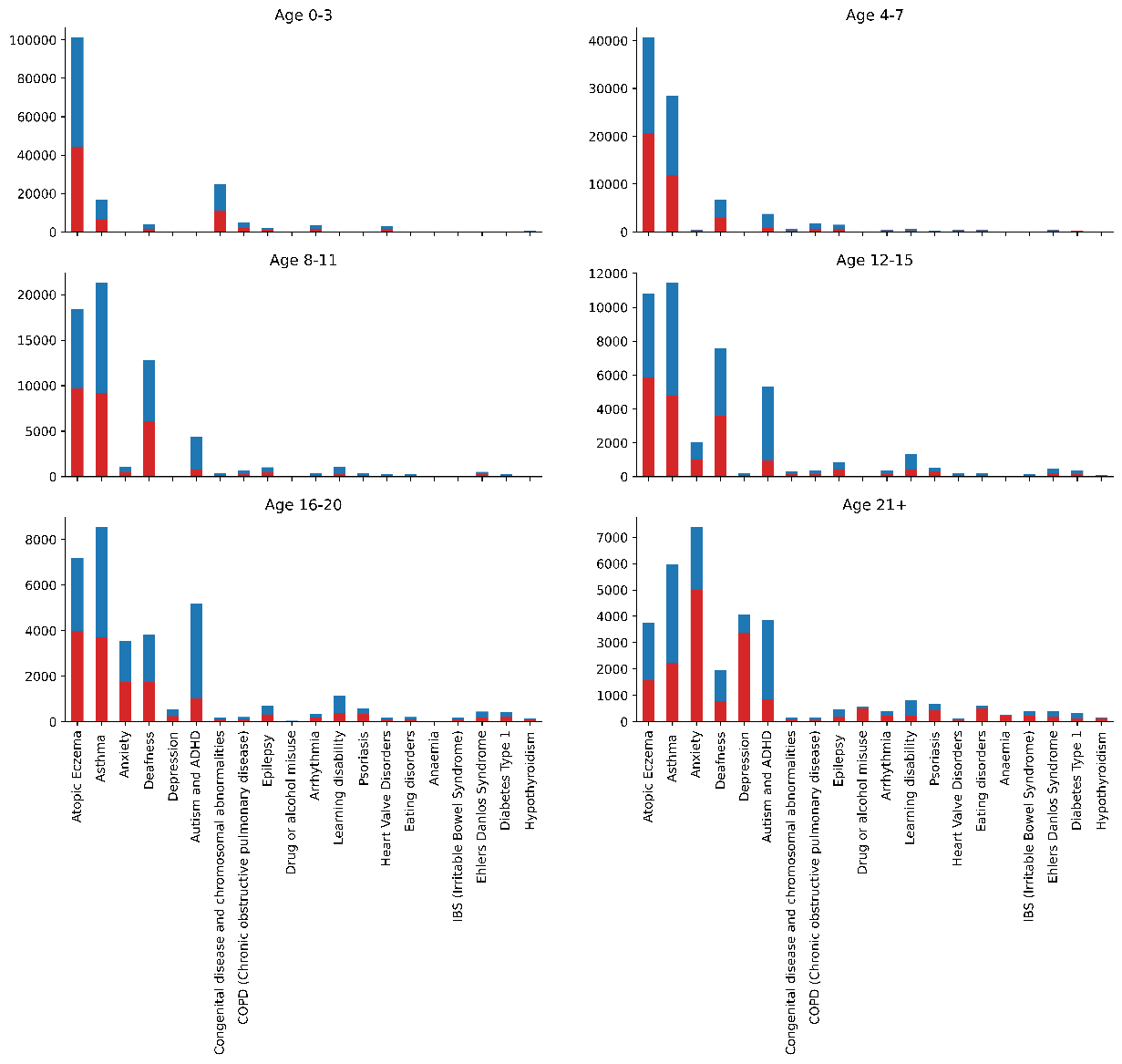


**Supplementary Table 2:** List of long-term conditions concepts approved and imported into SAIL at the initial stage

In the following table we report the provisional conditions selected by the MELD-B clinical group, their source, and their code lists sources. These concepts have gone through the full concept pipeline. All the concepts and associated code lists in this table are derived from [50] [51] and [52].

| ***CONCEPT*** | ***Code list source^[[1]](#footnote-1)^*** |
| --- | --- |
| Addison's disease | [52]: Addison_Disease_birm_cam |
| Anaemia | \| [52] : B12deficiency_birm_cam, \| \| --- \| \| PerniciousAnaemia_birm_cam, \| \| Folate%20deficiency_birm_cam \| |
| Aneurysm | [52] : AorticAneurysm_Bham_CAM |
| Ankylosing Spondylitis | [52] : AnkylosingSpondylitis_MM_birm_cam |
| Anxiety | [51]  [52]: Anxiety_birm_cam |
| Arrhythmia | [51]  [52]: Arrhythmia_Bham_CAM |
| Asthma | [51]  [52]: Asthma_PUSHAsthma |
| Atopic Eczema | [52]: AtopicEczema_birm_cam |
| Autism and ADHD | [52]: ADHD_mm_birm_cam, Autism_birm_cam |
| Bipolar Disorder | [52]: Bipolar_birm_cam |
| Blindness and low vision | [51]  [52]: Visual_Impairment_Birm_Cam |
| Breast Cancer | [52]: BreastCancer_birm_cam |
| Bronchiectasis | [51]  [52]: Bronchiectasis_birm_cam |
| Chronic Back Pain | [52]: Chronicbackpain_MM_birm_cam |
| Chronic Fatigue Syndrome | [52]: ChronicFatigueSyndromeMM_birm_cam |
| Chronic Liver Disease | [51]  [52]:ChronicLiverDisease_MM_birm_cam, Chronic_liver_disease_alcohol_birm_cam, Autoimmune_liver_disease_birm_cam, Hepatitis_B_birm_cam, Hepatitis_C_birm_cam, NAFLD_birm_cam |
| Chronic Pain | [52]: ComplexPainMM_birm_cam, Chronicbackpain_MM_birm_cam |
| Chronic Sinusitis | [51]  [52]: ChronicSinusitis_birm_cam |
| Chronic Kidney Disease (CKD) Stage 3_5 | [52]: CKDstage3to5_Bham_CAM |
| Coeliac Disease | [52]: Coeliac_disease_birm_cam |
| Colon Cancer | [52]: Colon_cancer__bham_cam |
| Congenital disease and chromosomal abnormalities | [52]: CongenitalHrtDx_NoSurg_Bham_CAM |
| COPD (Chronic obstructive pulmonary disease) | [51]  [52]: COPD_birm_cam |
| Coronary heart disease | [51]  [52]: IHD_noMI_Bham_CAM, MInfarction_Bham_CAM |
| Cystic fibrosis | [52]: CysticFibrosis_birm_cam |
| Deafness | [51]  [52]: SevereDeafHearingLoss_birm_cam, Any_Deafness_Hearing_Loss_birm_cam |
| Dementia alzheimer | [51]  [52]: DementiaOther_birm_cam, Alzheimers_birm_cam |
| Depression | [51]  [52]: Depression_birm_cam |
| Diabetes Type 1 | [52]: Type1DM_11_3_21_birm_cam |
| Diabetes Type 2 | [52]: Type2Diabetes_11_3_21_birm_cam |
| Diabetic retinopathy | [52]: diabetic_retinopathy_090121_birm_cam |
| Dialysis | [52] Dialysis MM_birm_cam |
| Diverticular Disease | [51]  [52]: diverticular_disease_birm_cam |
| Drug or alcohol misuse | [51]  [52]:AlcoholMisuse_birm_cam, SubstanceMisuse_birm_cam |
| Eating disorders | [51]  [52]: EatingDisorders_birm_cam |
| Ehlers Danlos Syndrome | [52]: EhlersDanlosSyndrome3_birm_cam |
| Endometriosis | [52]: Endometriosis_Adenomyosis_birm_cam |
| Epilepsy | [51]  [52]: Epilepsy_birm_cam |
| Fibromyalgia | [52]: Fibromyalgia_Bham_CAM |
| Glaucoma | [51]  [52]: Glaucoma_Prevalence_Birm_Cam |
| Gout | [52]: Gout_MM_birm_cam |
| Heart Valve Disorders | [52]: ValvularDiseases_BhaM_CAM |
| HF (Heart Failure) | [51]  [52]: HF_Bham_CAM_Final |
| HIV/AIDS | [52]: HIVaids_birm_cam |
| Hypertension | [51]  [52]: Hypertension_BhaM_CAM |
| Hyperthyroidism | [52]: Hyperthyroidism V2_birm_cam |
| Hypothyroidism | [52]: Hypothyroidism draft V1_birm_cam |
| IBD (Inflammatory bowel disease) | [51]  [52] : Crohns_disease_birm_cam, Ulcerative_colitis_birm_cam |
| IBS (Irritable Bowel Syndrome) | [51] |
| ILD (Inflammatory Lung Disease) | [52] : ILD_sh_20092020_birm_cam |
| Learning disability | [51]  [52]: Learningdisability_birm_cam |
| Leukaemia | [52]: leukaemia_prevalence_birm_cam |
| Lymphoma | [52]: lymphoma_prevalence_birm_cam |
| Marfan Syndrome | [52]: MarfanSyndrome_birm_cam |
| Meniere's disease | [52]: Menieresdisease_birm_cam |
| Metastatic cancers | [52]: MetastaticCancer_birm_cam |
| Multiple sclerosis | [51]  [52]: MS_120421_birm_cam |
| Osteoarthritis | [52]: Osteoarthritis_MM_birm_cam |
| Osteoporosis | [52]: Osteoporosis_birm_cam |
| Pancreatic disease | [52]: ChronicPancreatitis_MM_birm_cam |
| Paralysis | [52]: Hemi_Para_Quadriplegia_birm_cam |
| Parkinson's disease | [51]  [52]: Parkinsons_birm_cam |
| PCOS (Polycystic ovary syndrome) | [52]: Polycystic_ovarian_syndrome_PCOS_birm_cam_v2 |
| Plasmacell | [52]: plasmacell_neoplasm_birm_cam |
| PMR (Polymyalgia reheumatica) | [52]: Polymyalgiarheumatica_MM |
| Primary Lung Cancer | [52]: Primary lung cancer_birm_cam |
| Prostate Cancer | [52]: PrimaryProstateCa_prevalence_120421_birm_cam |
| Prostate disorders | [51]  [52]: Benign Prostate Hyperplasia_birm_cam |
| Psoriasis | [52]: Psoriasis_birm_cam |
| Psoriasis Arthritis | [52]: PsoriaticArthritis2021_MM_birm_cam |
| PTSD (Post-traumatic stress disorders) | [52]: PTSD_TLC_V2 |
| PVD (Pheripheral vascular disease) | [51]  [52] : PVD_Bham_CAM |
| Renal Transplant | [52] : Renal transplant_MM_birm_cam |
| Rheumatoid arthritis | [52]: RheumatoidArthritis_MM_birm_cam |
| Schizophrenia | [51]  [52]: SchizophreniaMM |
| Skin Cancer | [52]: SkinCancer_200421_birm_cam |
| Stroke | [52]: StrokeUnspecified_Bham_CAM, Stroke_Haemrgic_Bham_CAM |
| Systemic Lupus Erythematosus | [52]: Systemic_lupus_erythematosus_MM_birm_cam |
| Systemic sclerosis | [52]: Systemic_Sclerosis_MM_birm_cam |
| Thalassaemia | [52]: Thalassaemia_birm_cam |
| TIA (Transient ischaemic attack) | [52]: TIA_Bham_CAM |
| Viral Hepatitis | [51]  [52]: Hepatitis_B_birm_cam, Hepatitis_C_birm_cam |
| VTD (Venous thrombotic disease) | [52]: VTEnoPE_birm_cam |

**Supplementary Table 3:** SMC Evolution over the e-cohort study period

| **Year** | **Total (N)** | **Male**  **(N, %)** | **Female**  **(N ,%)** | **WIMD = 1 (%)** | **WIMD = 2 %** | **WIMD = 3 (%)** | **WIMD = 4 (%)** | **WIMD = 5 (%)** | **People leaving (N)** | **Deaths**  **(N, %)** | **Lost to follow-up**  **(N, %)** | **People joining (N)** | **New born**  **(N , %)** |
| --- | --- | --- | --- | --- | --- | --- | --- | --- | --- | --- | --- | --- | --- |
| **2000** | 2,990,123 | 1,477,430 (49.41) | 1,512,693 (50.59) | 20.57 | 20.07 | 20.37 | 19.70 | 19.26 | 101,648 | 31,919 (31.4) | 69,729  (68.6) | 105,654 | 26,248 (24.84) |
| **2001** | 2,994,184 | 1,481,187 (49.47) | 1,512,997 (50.53) | 20.41 | 19.99 | 20.41 | 19.73 | 19.41 | 94,869 | 31,236 (32.93) | 63,633 (67.07) | 95,940 | 25,733 (26.82) |
| **2002** | 2,995,269 | 1,483,212 (49.52) | 1,512,057 (50.48) | 20.24 | 19.91 | 20.37 | 19.80 | 19.64 | 104,774 | 31,041 (29.63) | 73,733 (70.37) | 100,048 | 25,022 (25.01) |
| **2003** | 2,990,558 | 1,481,707 (49.55) | 1,508,851 (50.45) | 20.05 | 19.82 | 20.37 | 19.87 | 19.85 | 84,111 | 32,548 (38.7) | 51,563  (61.3) | 103,729 | 26,586 (25.63) |
| **2004** | 3,010,192 | 1,493,705 (49.62) | 1,516,487 (50.38) | 19.96 | 19.75 | 20.41 | 19.93 | 19.91 | 83,055 | 31,695 (38.16) | 51,360 (61.84) | 99,527 | 27,245 (27.37) |
| **2005** | 3,026,683 | 1,503,414 (49.67) | 1,523,269 (50.33) | 19.91 | 19.74 | 20.41 | 19.97 | 19.93 | 79,923 | 31,723 (39.69) | 48,200 (60.31) | 104,258 | 28,439 (27.28) |
| **2006** | 3,0510,44 | 1,516,967 (49.72) | 1,534,077 (50.28) | 19.89 | 19.74 | 20.40 | 20.00 | 19.93 | 80,919 | 30,653 (37.88) | 50,266 (62.12) | 97,075 | 29,670 (30.56) |
| **2007** | 3,067,222 | 1,526,490 (49.77) | 1,540,732 (50.23) | 19.87 | 19.72 | 20.43 | 20.04 | 19.91 | 82,019 | 31,401 (38.29) | 50,618 (61.71) | 94,865 | 30,081 (31.71) |
| **2008** | 3,080,096 | 1,534,652 (49.82) | 1,545,444 (50.18) | 19.84 | 19.70 | 20.43 | 20.06 | 19.92 | 85,327 | 31,741 (37.2) | 53,586  (62.8) | 93,286 | 31,194 (33.44) |
| **2009** | 3,088,059 | 1,540,893 (49.9) | 1,547,166 (50.1) | 19.81 | 19.72 | 20.43 | 20.05 | 19.95 | 87,761 | 30,555 (34.82) | 57,206 (65.18) | 89,007 | 30,610 (34.39) |
| **2010** | 3,089,310 | 1,543,193 (49.95) | 1,546,117 (50.05) | 19.80 | 19.73 | 20.44 | 20.05 | 19.95 | 99,335 | 30,561 (30.77) | 68,774 (69.23) | 91,584 | 31,657 (34.57) |
| **2011** | 3,081,559 | 1,540,737 (50) | 1,540,822 (50) | 19.80 | 19.77 | 20.43 | 20.03 | 19.95 | 97,299 | 29,682 (30.51) | 67,617 (69.49) | 93,357 | 31,821 (34.09) |
| **2012** | 3,077,617 | 1,539,702 (50.03) | 1,5379,15 (49.97) | 19.83 | 19.77 | 20.43 | 19.99 | 19.95 | 99,588 | 30,825 (30.95) | 68,763 (69.05) | 92,103 | 31,875 (34.61) |
| **2013** | 3,070,132 | 1,536,640 (50.05) | 1,533,492 (49.95) | 19.88 | 19.77 | 20.43 | 20.01 | 19.90 | 103,938 | 31,005 (29.83) | 72,933 (70.17) | 94,646 | 30,156 (31.86) |
| **2014** | 3,060,841 | 1,532,653 (50.07) | 1,528,188 (49.93) | 19.90 | 19.75 | 20.36 | 20.01 | 19.94 | 98,647 | 30,687 (31.11) | 67,960 (68.89) | 92,464 | 30,124 (32.58) |
| **2015** | 3,054,662 | 1,530,274 (50.1) | 1,524,388 (49.9) | 19.95 | 19.73 | 20.37 | 20.01 | 19.91 | 97,303 | 31,597 (32.47) | 65,706 (67.53) | 92,003 | 30,082  (32.7) |
| **2016** | 3,049,364 | 1,528,550 (50.13) | 1,520,814 (49.87) | 20.02 | 19.71 | 20.37 | 20.01 | 19.87 | 97,241 | 32,073 (32.98) | 65,168 (67.02) | 93,787 | 29,778 (31.75) |
| **2017** | 3,045,910 | 1,526,866 (50.13) | 1,519,044 (49.87) | 20.06 | 19.73 | 20.35 | 20.01 | 19.83 | 98,619 | 32,007 (32.46) | 66,612 (67.54) | 93,177 | 29,352  (31.5) |
| **2018** | 3,040,468 | 1,523,050 (50.09) | 1,517,418 (49.91) | 20.09 | 19.73 | 20.33 | 20.02 | 19.80 | 96,033 | 32,917 (34.28) | 63,116 (65.72) | 91,973 | 28,644 (31.14) |
| **2019** | 3,036,408 | 1,520,715 (50.08) | 1,515,693 (49.92) | 20.15 | 19.73 | 20.33 | 20.01 | 19.75 | 95,119 | 32,089 (33.74) | 63,030 (66.26) | 91,317 | 26,706 (29.25) |
| **2020** | 3,032,606 | 1,518,740 (50.08) | 1,513,866 (49.92) | 20.18 | 19.72 | 20.37 | 19.99 | 19.72 | 90,097 | 36,157 (40.13) | 53,940 (59.87) | 74,609 | 25,750 (34.51) |
| **2021** | 3,017,118 | 1,511,147 (50.09) | 1,505,971 (49.91) | 20.17 | 19.70 | 20.34 | 20.05 | 19.71 | 105,720 | 34,727 (32.85) | 70,993 (67.15) | 102,100 | 26,006 (25.47) |

**Supplementary Table 4:** SMYC Evolution over the e-cohort study period

| **Year** | **Total (N)** | **Male**  **(N, %)** | **Female**  **(N ,%)** | **WIMD = 1 (%)** | **WIMD = 2 %** | **WIMD = 3 (%)** | **WIMD = 4 (%)** | **WIMD = 5 (%)** | **People leaving (N)** | **Deaths**  **(N, %)** | **Lost to follow-up**  **(N, %)** | **People joining (N)** | **New born**  **(N , %)** |
| --- | --- | --- | --- | --- | --- | --- | --- | --- | --- | --- | --- | --- | --- |
| **2000** | NA | NA | NA | NA | NA | NA | NA | NA | 259 | 16  (6.17) | 243 (93.82) | 26,184 | 26,184 (100) |
| **2001** | 25,926 | 13,453 (51.89) | 12,473 (48.11) | 25.28 | 20.57 | 18.44 | 17.97 | 25.28 | 980 | 35 (3.57) | 945 (96.43) | 30,754 | 25,693 (83.54) |
| **2002** | 55,700 | 28,779 (51.67) | 26,921 (48.33) | 24.68 | 20.53 | 18.27 | 18.11 | 24.68 | 1,765 | 40  (2.26) | 1,725 (97.74) | 31,514 | 24,994 (79.31) |
| **2003** | 85,451 | 44,226 (51.76) | 41,225 (48.24) | 24.16 | 20.57 | 18.51 | 18.15 | 24.16 | 2,049 | 51 (2.48) | 1,998 (97.51) | 34,108 | 26,573 (77.91) |
| **2004** | 117,510 | 60,651 (51.61) | 56,859 (48.39) | 23.99 | 20.40 | 18.62 | 18.24 | 23.99 | 2,392 | 63 (2.63) | 2,329 (97.37) | 35,153 | 27,237 (77.48) |
| **2005** | 150,274 | 77,416 (51.52) | 72,858 (48.48) | 24.12 | 20.33 | 18.49 | 18.33 | 24.12 | 2,789 | 59 (2.11) | 2,730 (97.89) | 39,170 | 28,438 (72.60) |
| **2006** | 186,660 | 96,131 (51.50) | 90,529 (48.50) | 24.29 | 20.30 | 18.48 | 18.21 | 24.29 | 3,386 | 76 (2.24) | 3,310 (97.76) | 38,766 | 29,670 (76.54) |
| **2007** | 222,032 | 114,254 (51.46) | 107,778 (48.54) | 24.19 | 20.31 | 18.55 | 18.26 | 24.19 | 3,706 | 71 (1.91) | 3,635 (98.09) | 39,558 | 30,081 (76.04) |
| **2008** | 257,893 | 132,625 (51.43) | 125,268 (48.57) | 24.15 | 20.33 | 18.49 | 18.32 | 24.15 | 4,550 | 60 (1.31) | 4,490 (98.69) | 41,829 | 31,194 (74.58) |
| **2009** | 295,161 | 151,832 (51.44) | 143,329 (46.56) | 24.04 | 20.37 | 18.62 | 18.25 | 24.04 | 4,977 | 76 (1.52) | 4,901 (98.48) | 40,648 | 30,610 (75.31) |
| **2010** | 330,831 | 170,056 (51.40) | 160,775 (48.60) | 23.90 | 20.42 | 18.65 | 18.34 | 23.90 | 6,516 | 69 (1.05) | 6,447 (98.95) | 42,213 | 31,656 (74.99) |
| **2011** | 366,528 | 188,301 (51.37) | 178,227 (48.63) | 23.82 | 20.52 | 18.70 | 18.39 | 23.82 | 6,561 | 62 (0.94) | 6,499 (99.06) | 42,826 | 31,821 (74.30) |
| **2012** | 402,793 | 206,930 (51.37) | 195,863 (48.63) | 23.84 | 20.48 | 18.73 | 18.36 | 23.84 | 6,816 | 79 (1.15) | 6,737 (99.85) | 42,714 | 31,875 (74.62) |
| **2013** | 438,691 | 225,158 (51.32) | 213,533 (48.68) | 23.84 | 20.46 | 18.77 | 18.46 | 23.84 | 7,230 | 84 (1.16) | 7,146 (98.84) | 42,027 | 30,156 (74.75) |
| **2014** | 473,488 | 243,107 (51.34) | 230,381 (48.66) | 23.83 | 20.51 | 18.73 | 18.47 | 23.83 | 7,376 | 59 (0.79) | 7,317 (99.21) | 41,976 | 30,124 (71.76) |
| **2015** | 508,090 | 260,838 (51.34) | 247,252 (48.66) | 23.81 | 20.44 | 18.80 | 18.50 | 23.81 | 7,162 | 75 (1.04) | 7,087 (99.96) | 42,137 | 30,054 (71.32) |
| **2016** | 543,065 | 278,678 (51.32) | 264,387 (48.68) | 23.83 | 20.38 | 18.81 | 18.55 | 23.83 | 7,707 | 80 (1.03) | 7,627 (98.97) | 41,875 | 29,443 (70.31) |
| **2017** | 577,231 | 296,134 (51.30) | 281,097 (48.70) | 23.83 | 20.39 | 18.75 | 18.59 | 23.83 | 8,433 | 97 (1.15) | 8,336 (98.85) | 42,211 | 29,342 (69.51) |
| **2018** | 611,009 | 313,493 (51.31) | 297,516 (48.69) | 23.81 | 20.34 | 18.77 | 18.64 | 23.81 | 10,150 | 100 (0.98) | 10,050 (99.02) | 41,433 | 28,639 (69.12) |
| **2019** | 642,292 | 329,670 (51.33) | 312,622 (46.67) | 23.86 | 20.30 | 18.80 | 18.59 | 23.86 | 12,562 | 81 (0.64) | 12,481 (99.36) | 39,830 | 26,696 (67.02) |
| **2020** | 669,560 | 343,844 (51.35) | 325,716 (48.65) | 23.89 | 20.28 | 18.86 | 18.54 | 23.89 | 11,440 | 91 (0.79) | 11,349 (99.21) | 35,881 | 25,727 (71.70) |
| **2021** | 694,001 | 356,678 (51.39) | 337,323 (48.61) | 23.89 | 20.24 | 18.90 | 18.58 | 23.89 | 13,062 | 79 (0.60) | 12,983 (99.40) | 39,552 | 25,993 (65.72) |
| **2022** | 720,491 | 370,805 (51.47) | 349,686 (48.53) | 23.78 | 20.19 | 19.05 | 18.58 | 23.78 | 13,553 | 82 (0.61) | 13,471 (99.39) | 43,796 | 25,948 (59.25) |

**Supplementary Table 5:** Number of male and female individuals and number of admissions for each concept and each cohort.

|  | **SMC** | | | | **SMYC** | | | |
| --- | --- | --- | --- | --- | --- | --- | --- | --- |
| **Concept** | **N of male individuals (WLGP or PEDW)** | **N of female individuals (PEDW or WLGP)** | **N of admissions for concept (WLGP)** | **N of admissions for concept (PEDW)** | **N of male individuals (WLGP or PEDW)** | **N of female individuals (WLGP or PEDW)** | **N of admissions for concept (WLGP)** | **N of admissions for concept (PEDW)** |
| Addison's disease | 1,819 | 2,485 | 5,288 | 13,883 | 110 | 80 | 210 | 260 |
| Anaemia | 75,178 | 136,276 | 2,854,711 | 43,691 | 860 | 3,020 | 20,720 | 70 |
| Aneurysm | 30,231 | 11,975 | 43,718 | 83,306 | 40 | 30 | 40 | 60 |
| Ankylosing Spondylitis | 5,876 | 2,818 | 11,596 | 17,459 | 10 | 10 | 30 | 10 |
| Anxiety | 421,188 | 711,418 | 2,761,797 | 393,537 | 19,380 | 34,510 | 85,470 | 10,690 |
| Arrhythmia | 294,364 | 285,688 | 899,258 | 1,365,715 | 3,840 | 3,430 | 6,640 | 5,080 |
| Asthma | 436,608 | 491,065 | 7,657,263 | 1,243,152 | 59,010 | 44,830 | 618,420 | 68,940 |
| Atopic Eczema | 352,562 | 421,954 | 1,575,992 | 5,486 | 98,710 | 91,260 | 403,530 | 1,130 |
| Autism and ADHD | 46,209 | 17,825 | 92,767 | 42,716 | 22,680 | 7,460 | 40,690 | 17,920 |
| Bipolar Disorder | 12,190 | 19,209 | 45,588 | 64,734 | 70 | 210 | 200 | 240 |
| Blindness and low vision | 29,722 | 39,555 | 100,881 | 22,806 | 1,000 | 860 | 2,590 | 1,260 |
| Breast Cancer | 1,224 | 113,038 | 121,975 | 965,366 | 10 | 460 | 560 | 20 |
| Bronchiectasis | 14,744 | 16,377 | 38,469 | 72,452 | 190 | 130 | 380 | 70 |
| Chronic Back Pain | 27,762 | 37,263 | 135,174 | // | 90 | 110 | 220 | // |
| Chronic Fatigue Syndrome | 24,647 | 30,621 | 78,743 | // | 380 | 490 | 1,150 | // |
| Chronic Liver Disease | 46,417 | 35,886 | 86,452 | 174,139 | 360 | 300 | 620 | 1,660 |
| Chronic Pain | 35,937 | 51,797 | 165,087 | 17,724 | 180 | 350 | 600 | 330 |
| Chronic Sinusitis | 29,457 | 46,726 | 105,900 | // | 540 | 580 | 1,320 | // |
| Chronic Kidney Disease (CKD) Stage 3_5 | 101,539 | 129,334 | 545,909 | 345,884 | 160 | 80 | 360 | 5,200 |
| Coeliac Disease | 6,680 | 13,505 | 26,577 | 38,808 | 780 | 1,310 | 2,600 | 1,930 |
| Colon Cancer | 39,596 | 32,318 | 62,307 | 416,141 | 30 | 40 | 10 | 100 |
| Congenital disease and chromosomal abnormalities | 26,786 | 24,591 | 42,443 | 70,887 | 14,870 | 11,990 | 16,810 | 42,760 |
| COPD (Chronic obstructive pulmonary disease) | 192,803 | 189,528 | 1,939,227 | 1,008,129 | 5,090 | 3,320 | 9,530 | 890 |
| Coronary heart disease | 274,050 | 218,947 | 1,392,422 | 1,732,122 | 390 | 330 | 690 | 390 |
| Cystic fibrosis | 689 | 1,106 | 5,181 | 19,498 | 220 | 180 | 1,020 | 3,850 |
| Deafness | 233,517 | 229,120 | 718,602 | // | 21,950 | 18,850 | 56,120 | // |
| Dementia alzheimer | 61,089 | 104,528 | 370,160 | 314,632 | 10 | 20 | 10 | 10 |
| Depression | 451,108 | 698,921 | 4,006,408 | 621,124 | 13,500 | 24,760 | 75,250 | 7,640 |
| Diabetes Type 1 | 30,827 | 25,663 | 79,215 | 216,754 | 1,480 | 1,340 | 3,950 | 9,670 |
| Diabetes Type 2 | 211,979 | 173,817 | 1,332,350 | 1,603,897 | 130 | 160 | 250 | 270 |
| Diabetic retinopathy | 76,158 | 57,434 | 351,522 | 120,183 | 200 | 210 | 620 | 70 |
| Dialysis | 8,082 | 4,931 | 14,090 | 319,702 | 80 | 70 | 170 | 4,240 |
| Diverticular Disease | 127,107 | 159,618 | 291,313 | 491,128 | 245,360 | 238,390 | 1,554,550 | // |
| Drug or alcohol misuse | 375,499 | 272,349 | 769,385 | 1,468,796 | 70 | 50 | 100 | 60 |
| Eating disorders | 4,110 | 24,927 | 45,481 | 12,652 | 3,690 | 3,820 | 6,060 | 4,830 |
| Ehlers Danlos Syndrome | 2,823 | 6,741 | 11,627 | // | 1,220 | 3,310 | 5,740 | 2,490 |
| Endometriosis | 39 | 30,451 | 51,833 | No PEDW data available | 1,620 | 1,720 | 3,860 | // |
| Epilepsy | 52,461 | 50,490 | 526,112 | 311,820 | 10 | 290 | 460 | // |
| Fibromyalgia | 5,514 | 39,935 | 75,012 | 55,132 | 4,440 | 3,730 | 17,630 | 26,050 |
| Glaucoma | 49,018 | 55,239 | 157,411 | 152,108 | 30 | 210 | 290 | 120 |
| Gout | 133,558 | 47,001 | 408,496 | 123,877 | 130 | 100 | 360 | 470 |
| Heart Valve Disorders | 98,338 | 99,546 | 218,429 | 416,839 | 40 | 20 | 90 | 10 |
| HF (Heart Failure) | 149,378 | 143,531 | 383,330 | 590,623 | 2,640 | 2,100 | 5,820 | 9,460 |
| HIV/AIDS | 0 | 0 | 0 | 0 | 640 | 510 | 870 | 1,790 |
| Hypertension | 536,164 | 566,459 | 3,389,947 | 3,531,075 | 430 | 370 | 970 | 1,100 |
| Hyperthyroidism | 11,379 | 44,722 | 81,067 | 57,427 | 110 | 290 | 540 | 370 |
| Hypothyroidism | 50,268 | 192,504 | 428,688 | 609,098 | 780 | 1,380 | 3,180 | 4,000 |
| IBD (Inflammatory bowel disease) | 26,638 | 30,031 | 116,043 | 234,526 | 670 | 500 | 1,500 | 8,150 |
| IBS (Irritable Bowel Syndrome) | 58,948 | 157,163 | 336,107 | // | 1,380 | 2,450 | 4,650 | // |
| ILD (Inflammatory Lung Disease) | 19,190 | 11,291 | 28,097 | 55,374 | 50 | 50 | 80 | 50 |
| Learning disability | 22,309 | 13,467 | 145,763 | // | 4,630 | 2,220 | 10,700 | // |
| Leukaemia | 15,596 | 12,028 | 22,352 | 291,177 | 360 | 240 | 620 | 27,590 |
| Lymphoma | 8,713 | 6,974 | 28,942 | // | 120 | 70 | 260 | // |
| Marfan Syndrome | 508 | 254 | 1,281 | // | 70 | 40 | 170 | // |
| Meniere's disease | 13,239 | 26,571 | 62,201 | // | 30 | 40 | 70 | // |
| Metastatic cancers | 81,795 | 82,920 | 58,810 | 1,354,577 | 110 | 100 | 60 | 6,090 |
| Multiple sclerosis | 3,473 | 7,967 | 26,555 | 76,537 | 20 | 40 | 60 | 0 |
| Osteoarthritis | 314,975 | 430,019 | 1,160,525 | 949,936 | 190 | 200 | 350 | 140 |
| Osteoporosis | 93,772 | 195,569 | 214,880 | 812,551 | 300 | 290 | 110 | 890 |
| Pancreatic disease | 6,206 | 3,886 | 9,087 | 29,866 | 20 | 30 | 30 | 230 |
| Paralysis | 32,652 | 32,684 | 22,914 | 136,276 | 980 | 690 | 1,330 | 5,730 |
| Parkinson's disease | 20,529 | 15,187 | 49,600 | 115,684 | 10 | 10 | 10 | 0 |
| PCOS (Polycystic ovary syndrome) | 51 | 36,678 | 55,874 | // | 0 | 1,240 | 1,520 | // |
| Plasmacell | 4,029 | 3,488 | 9,423 | 134,540 | 10 | 10 | 10 | 10 |
| PMR (Polymyalgia reheumatica) | 16,730 | 34,542 | 116,063 | 79,353 | 10 | 10 | 10 | 0 |
| Primary Lung Cancer | 30,387 | 24,748 | 42,979 | 280,280 | 30 | 10 | 30 | 100 |
| Prostate Cancer | 63,638 | 43 | 67,667 | 398,883 | 10 | 10 | 10 | 0 |
| Prostate disorders | 151,471 | 129 | 322,084 | // | 30 | 10 | 40 | // |
| Psoriasis | 70,848 | 80,034 | 354,750 | 237,866 | 2,040 | 2,750 | 7,580 | 3,040 |
| Psoriasis Arthritis | 5,820 | 6,578 | 20,335 | 22,165 | 20 | 40 | 50 | 110 |
| PTSD (Post-traumatic stress disorders) | 15,780 | 16,695 | 38,886 | 21,040 | 300 | 830 | 1,020 | 900 |
| Pheripheral vascular disease | 122,079 | 128,337 | 411,730 | 225,698 | 70 | 40 | 180 | 2,290 |
| Renal transplant | 2,716 | 1,665 | 21,562 | 41,356 | 50 | 80 | 120 | 60 |
| Rheumatoid Arthritis | 22,446 | 48,748 | 199,980 | 253,548 | 160 | 120 | 260 | 170 |
| Schizophrenia | 16,676 | 12,195 | 49,896 | 84,754 | 160 | 190 | 350 | 120 |
| Skin Cancer | 127,493 | 136,543 | 482,073 | 145,245 | 580 | 350 | 980 | 360 |
| Stroke | 1,107 | 5,325 | 9,068 | 18,840 | 400 | 270 | 600 | 390 |
| Systemic Lupus Erythematosus | 543 | 2,114 | 3,903 | 11,474 | 20 | 50 | 70 | 240 |
| Systemic sclerosis | 1,485 | 2,446 | 2,898 | 9,496 | 20 | 30 | 50 | 70 |
| Thalassaemia | 55,324 | 59,117 | 138,729 | 52,448 | 180 | 170 | 300 | 1,210 |
| TIA (Transient ischaemic attack) | 6,876 | 6,510 | 15,049 | 5,528 | 60 | 40 | 90 | 30 |
| Viral Hepatitis | 0 | 0 | 0 | 0 | 180 | 170 | 330 | 270 |
| VTD (Venous thrombotic disease) | 46,620 | 60,261 | 140,423 | 56,167 | 6,330 | 5,040 | 25,880 | // |

Note that the number related to SMYC have been rounded up to the closest 10 to avoid disclosure control.

1. If the code list is derived from [52], we add the folder(s) the code lists are extracted from. [↑](#footnote-ref-1)
