# Supplementary material for: Cohort profile: creation of the SAIL MELD-B e-cohort (SMC) and SAIL MELD-B children and young adult e-cohort (SMYC) to investigate the lived experience of the ‘burdensomeness’ of multimorbidity
Source: BMJ Open. 2025 Jan 7;15(1):e087946. doi: 10.1136/bmjopen-2024-087946 (PMC11792564; doi:10.1136/bmjopen-2024-087946)
Supplement: online supplemental file 2 [file bmjopen-15-1-s002.pdf]

ReadV2 codes

CONCEPT: Addison's disease

READ_CODE	DESCRIPTION_SAIL	SENSITIVE_CODE
A176.	Tuberculosis of adrenal glands - Addison's disease	0
C153.	Other corticoadrenal overactivity	0
C154.	Corticoadrenal insufficiency	0
C1540	Acute adrenal insufficiency	0
C1541	Addison's disease	0
C1545	Postprocedural adrenocortical(-medullary) hypofunction	0
C1546	Addisonian crisis	0
C154z	Corticoadrenal insufficiency NOS	0
C155.	Other adrenal hypofunction	0
C1550	Adrenal medullary insufficiency	0
C155z	Other adrenal hypofunction NOS	0
F3950	Myopathy due to Addison's disease	0
PK13.	Hypoplasia of adrenal gland	0

CONCEPT: Anaemia

READ_CODE	DESCRIPTION_SAIL	SENSITIVE_CODE
1452.	H/O: Anaemia vit.B12 deficient	0
2126H	Vitamin B12 deficiency resolved	0
42T2.	Serum vitamin B12 low	0
42U2.	Serum folate low	0
42U8.	RBC folate low	0
42UB.	Whole blood folate low	0
42UD.	RBC folate abnormal	0
66E..	B12 deficiency monitoring	0
66E3.	B12 injections started	0
66E4.	B12 injections - at home	0
66E5.	B12 injections - at surgery	0
66E8.	B12 injections stopped	0
66EZ.	B12 defic. monitoring NOS	0
7L185	Intramuscular injection of vitamin B12	0
C2620	Folic acid deficiency	0
C2621	Vitamin B12 deficiency	0
D010.	Pernicious anaemia	0
D011.	Other vitamin B12 deficiency anaemias	0
D0110	Vitamin B12 deficiency anaemia due to dietary causes	0
D0111	Vitamin B12 deficiency anaemia due to malabsorption with proteinuria	0
D0112	Transcobalamin II deficiency	0
D011X	Vitamin B12 deficiency anaemia, unspecified	0
D011z	Other vitamin B12 deficiency anaemia NOS	0
D012.	Folate-deficiency anaemia	0
D0120	Congenital folate malabsorption anaemia	0
D0121	Folate-deficiency anaemia due to dietary causes	0
D0122	Folate-deficiency anaemia, drug induced	0
D0123	Folate-deficiency anaemia due to malabsorption	0
D0124	Folate-deficiency anaemia due to liver disorders	0
D012z	Folate-deficiency anaemia NOS	0
D0130	Combined B12 and folate deficiency anaemia	0
Dyu01	[X]Other dietary vitamin B12 deficiency anaemia	0
Dyu02	[X]Other vitamin B12 deficiency anaemias	0
Dyu03	[X]Other folate deficiency anaemias	0
Dyu06	[X]Vitamin B12 deficiency anaemia, unspecified	0
F11x6	Cerebral degeneration due to vitamin B12 deficiency	0
F3815	Myasthenic syndrome due to pernicious anaemia	0

CONCEPT: Aneurysm

READ_CODE	DESCRIPTION_SAIL	SENSITIVE_CODE
14AE.	H/O: aortic aneurysm	0
585I0	Abdominal aortic aneurysm screen ultrasound scan abnormal	0
68B51	Aortic aneurysm screening abnormal	0
7A11.	Replacement of aneurysmal bifurcation of aorta	0
7A110	Emergency replacement of aneurysmal bifurcation of aorta by anastomosis of aorta to femoral artery	0
7A111	Replacement of aneurysmal bifurcation of aorta by anastomosis of aorta to femoral artery	0
7A112	Emergency replacement of aneurysmal bifurcation of aorta by anastomosis of aorta to iliac artery	0
7A113	Replacement of aneurysmal bifurcation of aorta by anastomosis of aorta to iliac artery	0
7A11y	Other specified replacement of aneurysmal bifurcation of aorta	0
7A11z	Replacement of aneurysmal bifurcation of aorta NOS	0
7A13.	Emergency replacement of aneurysmal segment of aorta	0
7A130	Emergency replacement of aneurysmal segment of ascending aorta by anastomosis of aorta to aorta	0
7A131	Emergency replacement of aneurysmal segment of thoracic aorta by anastomosis of aorta to aorta	0
7A132	Emergency replacement of aneurysmal segment of suprarenal abdominal aorta by anastomosis of aorta to aorta	0
7A133	Emergency replacement of aneurysmal segment of infrarenal abdominal aorta by anastomosis of aorta to aorta	0
7A134	Emergency replacement of aneurysmal segment of abdominal aorta by anastomosis of aorta to aorta NEC	0
7A13y	Other specified emergency replacement of aneurysmal segment of aorta	0
7A13z	Emergency replacement of aneurysmal segment of aorta NOS	0
7A14.	Other replacement of aneurysmal segment of aorta	0
7A140	Replacement of aneurysmal segment of ascending aorta by anastomosis of aorta to aorta NEC	0
7A141	Replacement of aneurysmal segment of thoracic aorta by anastomosis of aorta to aorta NEC	0
7A142	Replacement of aneurysmal segment of suprarenal abdominal aorta by anastomosis of aorta to aorta NEC	0
7A143	Replacement of aneurysmal segment of infrarenal abdominal aorta by anastomosis of aorta to aorta NEC	0
7A144	Replacement of aneurysmal segment of abdominal aorta by anastomosis of aorta to aorta NEC	0
7A14y	Other specified other replacement of aneurysmal segment of aorta	0
7A14z	Other replacement of aneurysmal segment of aorta NOS	0
7A15.	Other emergency bypass of segment of aorta	0
7A150	Emergency bypass of segment of ascending aorta by anastomosis of aorta to aorta NEC	0
7A151	Emergency bypass of segment of thoracic aorta by anastomosis of aorta to aorta NEC	0
7A152	Emergency bypass of segment of suprarenal abdominal aorta by anastomosis of aorta to aorta NEC	0
7A153	Emergency bypass of segment of infrarenal abdominal aorta by anastomosis of aorta to aorta NEC	0
7A154	Emergency bypass of segment of abdominal aorta by anastomosis of aorta to aorta NEC	0
7A15y	Other specified other emergency bypass of segment of aorta	0
7A15z	Other emergency bypass of segment of aorta NOS	0
7A180	Plastic repair of aorta and end to end anastomosis of aorta	0
7A194	Operation on aneurysm of aorta NEC	0
7A1B.	Transluminal operations on aneurysmal segment of aorta	0

7A1B0	Endovascular stenting of infrarenal abdominal aortic aneurysm	0
7A1B1	Endovascular stenting of suprarenal aortic aneurysm	0
7A1B2	Endovascular stenting of thoracic aortic aneurysm	0
7A1B5	Endovascular stenting of aorto-uniiliac aneurysm	0
7A1B6	Endovascular stenting for aortic aneurysm of bifurcation NEC	0
7A1B7	Endovascular stenting for aorto-uniiliac aneurysm	0
7A1B8	Endovascular insertion of stent for infrarenal abdominal aortic aneurysm	0
7A1B9	Endovascular insertion of stent for suprarenal aortic aneurysm	0
7A1BA	Endovascular insertion of stent for thoracic aortic aneurysm	0
7A1BC	Endovascular insertion of stent for aortic aneurysm of bifurcation NEC	0
7A1BD	Endovascular insertion of stent for aorto-uniiliac aneurysm	0
7A1By	Other specified transluminal operations on aneurysmal segment of aorta	0
7A1Bz	Transluminal operations on aneurysmal segment of aorta NOS	0
7A1C.	Transluminal insertion of stent graft for aneurysmal segment of aorta	0
7A1C0	Endovascular insertion of stent graft for infrarenal abdominal aortic aneurysm	0
7A1C1	Endovascular insertion of stent graft for suprarenal aortic aneurysm	0
7A1C2	Endovascular insertion of stent graft for thoracic aortic aneurysm	0
7A1C5	Endovascular insertion of stent graft for aorto-uniiliac aneurysm	0
7A1Cy	Other specified transluminal insertion of stent graft for aneurysmal segment of aorta	0
7A1Cz	Transluminal insertion of stent graft for aneurysmal segment of aorta NOS	0
A930.	Syphilitic aortic aneurysm	1
G71..	Aortic aneurysm	0
G710.	Dissecting aortic aneurysm	0
G711.	Thoracic aortic aneurysm which has ruptured	0
G712.	Thoracic aortic aneurysm without mention of rupture	0
G713.	Abdominal aortic aneurysm which has ruptured	0
G7130	Ruptured suprarenal aortic aneurysm	0
G714.	Abdominal aortic aneurysm without mention of rupture	0
G7140	Juxtarenal aortic aneurysm	0
G7141	Inflammatory abdominal aortic aneurysm	0
G7142	Infrarenal abdominal aortic aneurysm	0
G7143	Aneurysm of suprarenal aorta	0
G715.	Ruptured aortic aneurysm NOS	0
G7150	Thoracoabdominal aortic aneurysm, ruptured	0
G716.	Aortic aneurysm without mention of rupture NOS	0
G7160	Thoracoabdominal aortic aneurysm, without mention of rupture	0
G717.	Aortic aneurysm - syphilitic	1
G718.	Leaking abdominal aortic aneurysm	0
G71z.	Aortic aneurysm NOS	0
Gyu71	[X]Aortic aneurysm of unspecified site, ruptured	0
Gyu72	[X]Aortic aneurysm of unspecified site, nonruptured	0

CONCEPT: Ankylosing Spondylitis

READ_CODE	DESCRIPTION_SAIL	SENSITIVE_CODE
2377.	O/E - ankyl.spondyl.chest def.	0
388p.	BASDAI - Bath ankylosing spondylitis disease activity index	0
388p0	Bath Ankylosing Spondylitis Functional Index	0
38QL.	Bath Ankylosing Spondylitis Metrology Index	0
N0401	Other rheumatoid arthritis of spine	0
N0450	Juvenile ankylosing spondylitis	0
N100.	Ankylosing spondylitis	0

CONCEPT: Anxiety

READ_CODE	DESCRIPTION_SAIL	SENSITIVE_CODE
1466.	H/O: anxiety state	0
146G.	H/O: agoraphobia	0
1B13.	Anxiousness	0
2126J	Anxiety resolved	0
2258.	O/E - anxious	0
225J.	O/E - panic attack	0
285..	Neurotic condition, insight present	0
286..	Poor insight into neurotic condition	0
8G94.	Anxiety management training	0
8HHp.	Referral for guided self-help for anxiety	0
8IH53	Referral for guided self-help for anxiety declined	0
8IH57	Referral for psychological management of anxiety declined	0
8T23.	Referral for psychological management of anxiety	0
E2...	Neurotic, personality and other nonpsychotic disorders	0
E20..	Neurotic disorders	0
E200.	Anxiety states	0
E2000	Anxiety state unspecified	0
E2001	Panic disorder	0
E2002	Generalised anxiety disorder	0
E2003	Anxiety with depression	0
E2004	Chronic anxiety	0
E2005	Recurrent anxiety	0
E200z	Anxiety state NOS	0
E201.	Hysteria	0
E2010	Hysteria unspecified	0
E2011	Hysterical blindness	0
E2012	Hysterical deafness	0
E2013	Hysterical tremor	0
E2014	Hysterical paralysis	0
E2015	Hysterical seizures	0
E2016	Other conversion disorder	0
E2017	Hysterical amnesia	0
E2018	Hysterical fugue	0
E2019	Multiple personality	0
E201A	Dissociative reaction unspecified	0
E201B	Compensation neurosis	0
E201C	Phantom pregnancy	0
E201z	Hysteria NOS	0
E202.	Phobic disorders	0
E2020	Phobia unspecified	0
E2021	Agoraphobia with panic attacks	0
E2022	Agoraphobia without mention of panic attacks	0
E2023	Social phobia, fear of eating in public	0
E2024	Social phobia, fear of public speaking	0
E2025	Social phobia, fear of public washing	0
E2026	Acrophobia	0
E2027	Animal phobia	0

E2028	Claustrophobia	0
E2029	Fear of crowds	0
E202A	Fear of flying	0
E202B	Cancer phobia	0
E202C	Dental phobia	0
E202D	Fear of death	0
E202E	Fear of pregnancy	0
E202z	Phobic disorder NOS	0
E203.	Obsessive-compulsive disorders	0
E2030	Compulsive neurosis	0
E2031	Obsessional neurosis	0
E203z	Obsessive-compulsive disorder NOS	0
E204.	Neurotic depression reactive type	0
E205.	Neurasthenia - nervous debility	0
E206.	Depersonalisation syndrome	0
E207.	Hypochondriasis	0
E20y.	Other neurotic disorders	0
E20y0	Somatization disorder	0
E20y1	Writer's cramp neurosis	0
E20y2	Other occupational neurosis	0
E20y3	Psychasthenic neurosis	0
E20yz	Other neurotic disorder NOS	0
E20z.	Neurotic disorder NOS	0
E21..	Personality disorders	0
E210.	Paranoid personality disorder	0
E211.	Affective personality disorder	0
E2110	Unspecified affective personality disorder	0
E2111	Hypomanic personality disorder	0
E2112	Depressive personality disorder	0
E2113	Cyclothymic personality disorder	0
E211z	Affective personality disorder NOS	0
E212.	Schizoid personality disorder	0
E2120	Unspecified schizoid personality disorder	0
E2121	Introverted personality	0
E2122	Schizotypal personality	0
E212z	Schizoid personality disorder NOS	0
E213.	Explosive personality disorder	0
E214.	Compulsive personality disorders	0
E2140	Anankastic personality	0
E2141	Obsessional personality	0
E214z	Compulsive personality disorder NOS	0
E215.	Histrionic personality disorders	0
E2150	Unspecified histrionic personality disorder	0
E2151	Munchausen's syndrome	0
E2152	Emotionally unstable personality	0
E2153	Psychoinfantile personality	0
E215z	Histrionic personality disorder NOS	0
E216.	Inadequate personality disorder	0
E217.	Antisocial or sociopathic personality disorder	0
E21y.	Other personality disorders	0
E21y0	Narcissistic personality disorder	0
E21y1	Avoidant personality disorder	0
E21y2	Borderline personality disorder	0
E21y3	Passive-aggressive personality disorder	0
E21y4	Eccentric personality disorder	0
E21y5	Immature personality disorder	0
E21y6	Masochistic personality disorder	0
E21y7	Psychoneurotic personality disorder	0
E21yz	Other personality disorder NOS	0
E21z.	Personality disorder NOS	0
E26..	Physiological malfunction arising from mental factors	0
E260.	Psychogenic musculoskeletal symptoms	0
E2600	Psychogenic paralysis	0
E2601	Psychogenic torticollis	0
E260z	Psychogenic musculoskeletal symptoms NOS	0
E261.	Psychogenic respiratory symptoms	0
E2610	Psychogenic air hunger	0
E2611	Psychogenic cough	0
E2612	Psychogenic hiccough	0
E2613	Psychogenic hyperventilation	0
E2614	Psychogenic yawning	0
E2615	Psychogenic aphonia	0
E261z	Psychogenic respiratory symptom NOS	0
E262.	Psychogenic cardiovascular symptoms	0
E2620	Cardiac neurosis	0
E2621	Cardiovascular neurosis	0
E2622	Neurocirculatory asthenia	0
E2623	Psychogenic cardiovascular disorder	0
E262z	Psychogenic cardiovascular symptom NOS	0
E263.	Psychogenic skin symptoms	0
E2630	Psychogenic pruritus	0
E263z	Psychogenic skin symptoms NOS	0
E264.	Psychogenic gastrointestinal tract symptoms	0
E2640	Psychogenic aerophagy	0
E2642	Cyclical vomiting - psychogenic	0
E2643	Psychogenic diarrhoea	0
E2644	Psychogenic dyspepsia	0
E2645	Psychogenic constipation	0
E264z	Psychogenic gastrointestinal tract symptom NOS	0
E265.	Psychogenic genitourinary tract symptoms	0
E2650	Psychogenic genitourinary tract malfunction unspecified	0
E2651	Psychogenic vaginismus	0
E2652	Psychogenic dysmenorrhea	0
E2653	Psychogenic dysuria	0
E265z	Psychogenic genitourinary tract symptom NOS	0
E266.	Psychogenic endocrine malfunction	0
E267.	Psychogenic symptom of special sense organ	0
E26y.	Other psychogenic malfunction	0
E26y0	Bruxism (teeth grinding)	0
E26yz	Other psychogenic malfunction NOS	0
E26z.	Psychosomatic disorder NOS	0
E278.	Psychalgia	0
E2780	Psychogenic pain unspecified	0

E2781	Tension headache	0
E2782	Psychogenic backache	0
E278z	Psychalgia NOS	0
E28..	Acute reaction to stress	0
E280.	Acute panic state due to acute stress reaction	0
E281.	Acute fugue state due to acute stress reaction	0
E282.	Acute stupor state due to acute stress reaction	0
E283.	Other acute stress reactions	0
E2830	Acute situational disturbance	0
E2831	Acute posttrauma stress state	0
E283z	Other acute stress reaction NOS	0
E284.	Stress reaction causing mixed disturbance of emotion and conduct	0
E28z.	Acute stress reaction NOS	0
E29..	Adjustment reaction	0
E2900	Grief reaction	0
E292.	Adjustment reaction with predominant disturbance of other emotions	0
E2920	Separation anxiety disorder	0
E2921	Adolescent emancipation disorder	0
E2922	Early adult emancipation disorder	0
E2923	Specific academic or work inhibition	0
E2924	Adjustment reaction with anxious mood	0
E2925	Culture shock	0
E292y	Adjustment reaction with mixed disturbance of emotion	0
E292z	Adjustment reaction with disturbance of other emotion NOS	0
E293.	Adjustment reaction with predominant disturbance of conduct	0
E2930	Adjustment reaction with aggression	0
E2931	Adjustment reaction with antisocial behaviour	0
E2932	Adjustment reaction with destructiveness	0
E293z	Adjustment reaction with predominant disturbance of conduct NOS	0
E294.	Adjustment reaction with mixed disturbance of emotion and conduct	0
E29y.	Other adjustment reactions	0
E29y0	Concentration camp syndrome	0
E29y1	Other post-traumatic stress disorder	0
E29y2	Adjustment reaction with physical symptoms	0
E29y3	Elective mutism due to an adjustment reaction	0
E29y4	Adjustment reaction due to hospitalisation	0
E29y5	Other adjustment reaction with withdrawal	0
E29yz	Other adjustment reactions NOS	0
E29z.	Adjustment reaction NOS	0
E2D0.	Disturbance of anxiety and fearfulness in childhood and adolescence	0
E2D0z	Disturbance of anxiety and fearfulness in childhood and adolescence NOS	0
Eu054	[X]Organic anxiety disorder	0
Eu341	[X]Dysthymia	0
Eu4..	[X]Neurotic, stress - related and somoform disorders	0
Eu40.	[X]Phobic anxiety disorders	0
Eu400	[X]Agoraphobia	0
Eu401	[X]Social phobias	0
Eu402	[X]Specific (isolated) phobias	0
Eu403	[X]Needle phobia	0
Eu40y	[X]Other phobic anxiety disorders	0
Eu40z	[X]Phobic anxiety disorder, unspecified	0
Eu41.	[X]Other anxiety disorders	0
Eu410	[X]Panic disorder [episodic paroxysmal anxiety]	0
Eu411	[X]Generalized anxiety disorder	0
Eu412	[X]Mixed anxiety and depressive disorder	0
Eu413	[X]Other mixed anxiety disorders	0
Eu41y	[X]Other specified anxiety disorders	0
Eu41z	[X]Anxiety disorder, unspecified	0
Eu42.	[X]Obsessive - compulsive disorder	0
Eu420	[X]Predominantly obsessional thoughts or ruminations	0
Eu421	[X]Predominantly compulsive acts [obsessional rituals]	0
Eu422	[X]Mixed obsessional thoughts and acts	0
Eu42y	[X]Other obsessive-compulsive disorders	0
Eu42z	[X]Obsessive-compulsive disorder, unspecified	0
Eu43.	[X]Reaction to severe stress, and adjustment disorders	0
Eu430	[X]Acute stress reaction	0
Eu431	[X]Post - traumatic stress disorder	0
Eu432	[X]Adjustment disorders	0
Eu43y	[X]Other reactions to severe stress	0
Eu43z	[X]Reaction to severe stress, unspecified	0
Eu44.	[X]Dissociative [conversion] disorders	0
Eu440	[X]Dissociative amnesia	0
Eu441	[X]Dissociative fugue	0
Eu442	[X]Dissociative stupor	0
Eu443	[X]Trance and possession disorders	0
Eu444	[X]Dissociative motor disorders	0
Eu445	[X]Dissociative convulsions	0
Eu446	[X]Dissociative anaesthesia and sensory loss	0
Eu447	[X]Mixed dissociative [conversion] disorders	0
Eu44y	[X]Other dissociative [conversion] disorders	0
Eu44z	[X]Dissociative [conversion] disorder, unspecified	0
Eu45.	[X]Somatoform disorders	0
Eu450	[X]Somatization disorder	0
Eu451	[X]Undifferentiated somatoform disorder	0
Eu452	[X]Hypochondriacal disorder	0
Eu453	[X]Somatoform autonomic dysfunction	0
Eu454	[X]Persistent somatoform pain disorder	0
Eu455	[X]Globus pharyngeus	0
Eu45y	[X]Other somatoform disorders	0
Eu45z	[X]Somatoform disorder, unspecified	0
Eu46.	[X]Other neurotic disorders	0
Eu460	[X]Neurasthenia	0
Eu461	[X]Depersonalization - derealization syndrome	0
Eu46y	[X]Other specified neurotic disorders	0
Eu46z	[X]Neurotic disorder, unspecified	0
Eu515	[X]Nightmares	0
Eu606	[X]Anxious [avoidant] personality disorder	0
Eu930	[X]Separation anxiety disorder of childhood	0
Eu931	[X]Phobic anxiety disorder of childhood	0
Eu932	[X]Social anxiety disorder of childhood	0
Eu93y	[X]Other childhood emotional disorders	0
M240E	Alopecia neurotica	0

CONCEPT: Arrhythmia

READ_CODE	DESCRIPTION_SAIL	SENSITIVE_CODE
14AD.	H/O ventricular fibrillation	0
14AN.	H/O: atrial fibrillation	0
14AP.	History of ventricular tachycardia	0
14AQ.	History of supraventricular tachycardia	0
14AR.	History of atrial flutter	0
14O71	At risk of atrial fibrillation	0
14V1.	H/O: cardiac pacemaker in situ	0
1R00.	For attempted cardiopulmonary resuscitation	0
1R10.	Not for attempted CPR (cardiopulmonary resuscitation)	0
212R.	Atrial fibrillation resolved	0
2241.	O/E - collapse -cardiac arrest	0
2JS..	Patient with internal cardiac defibrillator pacemaker	0
32130	Exercise ECG normal	0
32131	Exercise ECG abnormal	0
32141	Ambulatory ECG abnormal	0
3217.	ECG abnormal	0
3218.	ECG - improved	0
3219.	ECG equivocal	0
321C.	ECG sinus rhythm	0
321C0	ECG: sinus bradycardia	0
321C1	ECG: sinus tachycardia	0
322..	ECG: myocardial ischaemia	0
3222.	ECG:shows myocardial ischaemia	0
322Z.	ECG: myocardial ischaemia NOS	0
323..	ECG: myocardial infarction	0
3232.	ECG: old myocardial infarction	0
3233.	ECG: antero-septal infarct.	0
3234.	ECG:posterior/inferior infarct	0
3235.	ECG: subendocardial infarct	0
3236.	ECG: lateral infarction	0
323Z.	ECG: myocardial infarct NOS	0
324..	ECG:left ventricle hypertrophy	0
3242.	ECG: shows LVH	0
324Z.	ECG: LVH NOS	0
325..	ECG:right ventricle hypertrop.	0
3252.	ECG: shows RVH	0
325Z.	ECG: RVH NOS	0
326..	ECG: ectopic beats	0
3262.	ECG: extrasystole	0
3263.	ECG: ventricular ectopics	0
3264.	ECG: atrial ectopics	0
326Z.	ECG: ectopic beats NOS	0
327..	ECG: supraventricular arrhythmia	0
3272.	ECG: atrial fibrillation	0
3273.	ECG: atrial flutter	0
3274.	ECG: paroxysmal atrial tachy.	0
327Z.	ECG: supraventric. arryth. NOS	0
328..	ECG: ventricular arrhythmia	0
3282.	ECG: ventricular tachycardia	0
3283.	ECG: ventricular fibrillation	0
328Z.	ECG: ventricular arrhythmia NOS	0
329..	ECG: heart block	0
3292.	ECG: partial sinu-atrial block	0
3293.	ECG:complete sinu-atrial block	0
3294.	ECG:partial A-V block-long P-R	0
3295.	ECG: partial A-V block - 2:1	0
3296.	ECG: partial A-V block - 3:1	0
3297.	ECG: Wenckebach phenomenon	0
3298.	ECG: complete A-V block	0
3299.	ECG: right bundle branch block	0
329A.	ECG: left bundle branch block	0
329B.	ECG: trifascicular block	0
329C.	ECG: bifascicular block	0
329D.	ECG: left anterior fascicular block	0
329E.	ECG: left posterior fascicular block	0
329F.	ECG: right bundle branch and left anterior fascicular block	0
329G.	ECG: right bundle branch and left posterior fascicular block	0
329H.	Electrocardiogram: Mobitz type 2 second degree atrioventricular block	0
329Z.	ECG: heart block NOS	0
32A2.	ECG: P wave abnormal	0
32A3.	ECG: P mitrale	0
32A4.	ECG: P pulmonale	0
32B..	ECG: Q wave	0
32B2.	ECG: Q wave abnormal	0
32B3.	ECG: Q wave pathological	0
32BZ.	ECG: Q wave NOS	0
32C..	ECG: R wave	0
32C2.	ECG: R wave abnormal	0
32C3.	ECG: R wave tall	0
32CZ.	ECG: R wave NOS	0
32D2.	ECG: S wave abnormal	0
32D3.	ECG: S wave deep	0
32E2.	ECG: S-T interval abnormal	0
32E3.	ECG: S-T elevation	0
32E4.	ECG: S-T depression	0
32F2.	ECG: T wave abnormal	0
32F3.	ECG: T wave flattened	0
32F4.	ECG: T wave inverted	0
32FZ.	ECG: T wave NOS	0
32G2.	ECG: U wave abnormal	0
32G3.	ECG: U wave exaggerated	0
32GZ.	ECG: U wave NOS	0
32H..	ECG: F wave	0
32H1.	ECG: F wave absent	0
32H2.	ECG: F wave present	0
32HZ.	ECG: F wave NOS	0
32I2.	ECG: P-R interval abnormal	0
32I3.	ECG: P-R interval prolonged	0
32I4.	ECG: P-R interval shortened	0
32J2.	ECG: QRS complex abnormal	0

32J3.	ECG: QRS complex prolonged	0
32J4.	ECG: QRS complex shortened	0
32J5.	Left axis deviation	0
32J6.	Right axis deviation	0
32K2.	ECG: Q-T interval abnormal	0
32K3.	ECG: Q-T interval prolonged	0
32K4.	ECG: Q-T interval shortened	0
32L..	ECG: left ventricular strain	0
662S.	Atrial fibrillation monitoring	0
6A9..	Atrial fibrillation annual review	0
79321	Open chest massage of heart	0
7936.	Introduction of cardiac pacemaker system via vein	0
79360	Implantation of intravenous cardiac pacemaker system	0
79361	Resiting of lead of intravenous pacemaker system	0
79362	Maintenance of battery of intravenous cardiac pacemaker system	0
79363	Maintenance of intravenous cardiac pacemaker system NEC	0
79364	Removal of intravenous cardiac pacemaker system	0
79365	Implantation of emergency intravenous cardiac pacemaker	0
79366	Implantation of permanent intravenous cardiac pacemaker	0
79367	Implantation of intravenous fixed-rate cardiac pacemaker	0
79368	Implantation of intravenous triggered cardiac pacemaker	0
79369	Implantation of intravenous atrial overdrive pacemaker	0
7936A	Implantation of intravenous pacemaker for atrial fibrillation	0
7936B	Implantation of simple one wire intravenous cardiac pacemaker	0
7936C	Implantation of complex one wire intravenous cardiac pacemaker	0
7936D	Implantation of complex two wire intravenous cardiac pacemaker	0
7936E	Implantation of intravenous dual chamber permanent pacemaker	0
7936F	Renewal of intravenous cardiac pacemaker system	0
7936G	Implantation of intravenous single chamber cardiac pacemaker system	0
7936H	Implantation of intravenous dual chamber cardiac pacemaker system	0
7936J	Implantation of intravenous biventricular cardiac pacemaker system	0
7936K	Implantation of intravenous cardiac pacemaker system NEC	0
7936y	Other specified cardiac pacemaker system introduced via vein	0
7936z	Cardiac pacemaker system introduced via vein NOS	0
7937.	Other cardiac pacemaker system	0
79370	Implantation of cardiac pacemaker system NEC	0
79371	Resiting of lead of cardiac pacemaker system NEC	0
79372	Maintenance of battery of cardiac pacemaker system NEC	0
79373	Maintenance of cardiac pacemaker system NEC	0
79374	Removal of cardiac pacemaker system NEC	0
79375	Implantation of internal cardiac defibrillator	0
79376	Removal of internal cardiac defibrillator	0
79377	Implantation of single chamber cardiac pacemaker system	0
79378	Implantation of dual chamber cardiac pacemaker system	0
79379	Implantation of biventricular cardiac pacemaker system	0
7937y	Other specified other cardiac pacemaker system	0
7937z	Other cardiac pacemaker system NOS	0
793F.	Cardioverter defibrillator introduced through the vein	0
793F0	Implantation of cardioverter defibrillator using one electrode lead	0
793F1	Implantation of cardioverter defibrillator using two electrode leads	0
793F2	Resiting of lead of cardioverter defibrillator	0
793F3	Renewal of cardioverter defibrillator	0
793F4	Removal of cardioverter defibrillator	0
793F5	Implantation of cardioverter defibrillator using three electrode leads	0
793Fy	Other specified cardioverter defibrillator introduced through the vein	0
793Fz	Cardioverter defibrillator introduced through the vein NOS	0
793M1	Percutaneous transluminal ablation of atrial wall for atrial flutter	0
793M2	Percutaneous transluminal internal cardioversion NEC	0
793M3	Percutaneous transluminal ablation of conducting system of heart for atrial flutter NEC	0
793P.	Other cardioverter defibrillator	0
793P0	Implantation of subcutaneous cardioverter defibrillator	0
793P1	Resiting of lead of subcutaneous cardioverter defibrillator	0
793P2	Renewal of subcutaneous cardioverter defibrillator	0
793P3	Removal of subcutaneous cardioverter defibrillator	0
793Py	Other specified other cardioverter defibrillator	0
793Pz	Unspecified other cardioverter defibrillator	0
7L1H.	External resuscitation	0
7L1H0	Direct current cardioversion	0
7L1H1	External cardioversion NEC	0
7L1H2	Internal electrode cardioversion	0
7L1H6	Advanced cardiopulmonary resuscitation	0
7L1H7	External ventricular defibrillation	0
7L1H8	Chemical cardioversion	0
7P19.	Pacemaker testing	0
7P190	Distant pacemaker test	0
7P19y	Other specified pacemaker testing	0
7P19z	Pacemaker testing NOS	0
7P1D.	Evaluation of cardioverter defibrillator	0
840..	Direct current cardioversion planned	0
853..	Cardiac massage - external	0
8531.	Closed cardiac massage alone	0
8532.	Closed cardiac massage+ventil.	0
853Z.	External cardiac massage NOS	0
88A9.	Deactivation of automatic implantable cardioverter defibrillator	0
88AA.	Automatic implantable cardioverter defibrillator check	0
8HRF.	Referral for cardiac pacemaker check	0
8HTy.	Referral to atrial fibrillation clinic	0
8OAD.	Provision of written information about atrial fibrillation	0
9N2b.	Seen by cardiac pacemaker technician	0
F2560	Hypsarrhythmia	0
G559.	Arrhythmogenic right ventricular cardiomyopathy	0
G56..	Conduction disorders	0
G560.	Complete atrioventricular block	0
G561.	Partial atrioventricular block	0
G5610	Atrioventricular block unspecified	0
G5611	First degree atrioventricular block	0
G5612	Mobitz type II atrioventricular block	0
G5613	Mobitz type I (Wenckebach) atrioventricular block	0
G5614	Second degree atrioventricular block	0
G561z	Atrioventricular block NOS	0
G562.	Left bundle branch hemiblock	0
G5620	Left anterior fascicular block	0
G5621	Left posterior fascicular block	0

G562z	Left bundle branch hemiblock NOS	0
G563.	Left main stem bundle branch block	0
G564.	Right bundle branch block	0
G565.	Other bundle branch block	0
G5650	Bundle branch block unspecified	0
G5651	Right bundle branch block with left posterior fascicular block	0
G5652	Right bundle branch block with left anterior fascicular block	0
G5653	Other bilateral bundle branch block	0
G5654	Trifascicular block	0
G5655	Bifascicular block	0
G565z	Other bundle branch block NOS	0
G566.	Other heart block	0
G5660	Sinoatrial block	0
G5661	Interventricular block NOS	0
G5662	Right fascicular block	0
G566z	Other heart block NOS	0
G567.	Anomalous atrioventricular excitation	0
G5670	Accelerated atrioventricular conduction	0
G5671	Accessory atrioventricular conduction	0
G5672	Pre-excitation atrioventricular conduction	0
G5673	Ventricular pre-excitation	0
G5674	Wolff-Parkinson-White syndrome	0
G567z	Anomalous atrioventricular excitation NOS	0
G56y.	Other conduction disorders	0
G56y0	Lown-Ganong-Levine syndrome	0
G56y1	Atrioventricular dissociation	0
G56y2	Romano - Ward syndrome	0
G56y3	Jervell and Lange-Nielsen syndrome	0
G56y4	Right fascicular block	0
G56y5	Long Q-T syndrome	0
G56y6	Pacemaker twiddler's syndrome	0
G56yz	Other conduction disorders NOS	0
G56z.	Conduction disorders unspecified	0
G56z0	Stokes-Adams syndrome	0
G56zz	Conduction disorders NOS	0
G57..	Cardiac dysrhythmias	0
G570.	Paroxysmal supraventricular tachycardia	0
G5700	Paroxysmal atrial tachycardia	0
G5701	Paroxysmal atrioventricular tachycardia	0
G5702	Paroxysmal junctional tachycardia	0
G5703	Paroxysmal nodal tachycardia	0
G570z	Paroxysmal supraventricular tachycardia NOS	0
G571.	Paroxysmal ventricular tachycardia	0
G572.	Paroxysmal tachycardia unspecified	0
G5720	Essential paroxysmal tachycardia	0
G5721	Bouveret-Hoffmann syndrome	0
G572z	Paroxysmal tachycardia NOS	0
G573.	Atrial fibrillation and flutter	0
G5730	Atrial fibrillation	0
G5731	Atrial flutter	0
G5732	Paroxysmal atrial fibrillation	0
G5733	Non-rheumatic atrial fibrillation	0
G5734	Permanent atrial fibrillation	0
G5735	Persistent atrial fibrillation	0
G5736	Paroxysmal atrial flutter	0
G5737	Chronic atrial fibrillation	0
G5738	Typical atrial flutter	0
G5739	Atypical atrial flutter	0
G573z	Atrial fibrillation and flutter NOS	0
G574.	Ventricular fibrillation and flutter	0
G5740	Ventricular fibrillation	0
G5741	Ventricular flutter	0
G574z	Ventricular fibrillation and flutter NOS	0
G575.	Cardiac arrest	0
G5750	Cardiac arrest with successful resuscitation	0
G5751	Sudden cardiac death, so described	0
G5752	Electromechanical dissociation with successful resuscitation	0
G5753	Electromechanical dissociation	0
G575z	Cardiac arrest, unspecified	0
G576.	Ectopic beats	0
G5760	Ectopic beats unspecified	0
G5761	Supraventricular ectopic beats	0
G5762	Ventricular ectopic beats	0
G5763	Atrial premature depolarization	0
G5764	Junctional premature depolarization	0
G5765	Ventricular premature depolarization	0
G576z	Ectopic beats NOS	0
G577.	Sinus arrhythmia	0
G578.	Atrial standstill	0
G57y.	Other cardiac dysrhythmias	0
G57y0	Persistent sinus bradycardia	0
G57y1	Severe sinus bradycardia	0
G57y2	Brugada syndrome	0
G57y3	Sick sinus syndrome	0
G57y4	Sinoatrial node dysfunction NOS	0
G57y5	Wandering atrial pacemaker	0
G57y6	Nodal rhythm disorder	0
G57y7	Sinus tachycardia	0
G57y8	Bigeminal pulse	0
G57y9	Supraventricular tachycardia NOS	0
G57yA	Re-entry ventricular arrhythmia	0
G57yz	Other cardiac dysrhythmia NOS	0
G57z.	Cardiac dysrhythmia NOS	0
Gyu5U	[X]Other and unspecified atrioventricular block	0
Gyu5W	[X]Other and unspecified right bundle-branch block	0
Gyu5X	[X]Other specified heart block	0
Gyu5Z	[X]Other and unspecified premature depolarization	0
Gyu5a	[X]Other specified cardiac arrhythmias	0
L0911	Defibrination syndrome following abortive pregnancy	1
L09y1	Cardiac arrest following abortive pregnancy	1
P6y5z	Congenital heart block NOS	0
PG57.	Infantile cortical hyperostosis	0
Q491.	Neonatal cardiac dysrhythmia	0

R0026	[D]Asystolic vasovagal syncope	0
R1431	[D]Electrocardiogram (ECG) abnormal	0
SP001	Mechanical complication of cardiac pacemaker	0
SP110	Cardiac arrest as a complication of care	0
TB010	Implant of cardiac pacemaker as the cause of abnormal reaction of patient, or of later complication, without mention of misadventure at the time of operation	0
ZV450	[V]Cardiac pacemaker in situ	0
ZV45M	[V]Biventricular pacemaker in situ	0
ZV530	[V]Fitting or adjustment of device related to nervous system or special senses	0
ZV533	[V]Fitting or adjustment of cardiac pacemaker	0

CONCEPT: Asthma

READ_CODE	DESCRIPTION_SAIL	SENSITIVE_CODE
13Y4.	Asthma society member	0
14B4.	H/O: asthma	0
14Ok0	At risk of severe asthma exacerbation	0
173A.	Exercise induced asthma	0
173c.	Occupational asthma	0
173d.	Work aggravated asthma	0
178..	Asthma trigger	0
1780.	Aspirin induced asthma	0
1781.	Asthma trigger - pollen	0
1782.	Asthma trigger - tobacco smoke	0
1783.	Asthma trigger - warm air	0
1784.	Asthma trigger - emotion	0
1785.	Asthma trigger - damp	0
1786.	Asthma trigger - animals	0
1787.	Asthma trigger - seasonal	0
1788.	Asthma trigger - cold air	0
1789.	Asthma trigger - respiratory infection	0
178A.	Asthma trigger - airborne dust	0
178B.	Asthma trigger - exercise	0
1J70.	Suspected asthma	0
1O2..	Asthma confirmed	0
21262	Asthma resolved	0
212G.	Asthma resolved	0
388t0	Royal College of Physicians asthma assessment three questions score	0
38B8.	Severe asthma exacerbation risk assessment	0
38DL.	Asthma control test	0
38DT.	Asthma control questionnaire	0
38DV.	Mini asthma quality of life questionnaire	0
38QM.	Childhood Asthma Control Test	0
661M1	Asthma self-management plan agreed	0
661N1	Asthma self-management plan review	0
663..	Respiratory disease monitoring	0
6636.	Inhaler technique shown	0
663I.	Inhaler technique - poor	0
663N.	Asthma disturbing sleep	0
663N0	Asthma causing night waking	0
663N1	Asthma disturbs sleep weekly	0
663N2	Asthma disturbs sleep frequently	0
663O.	Asthma not disturbing sleep	0
663O0	Asthma never disturbs sleep	0
663P.	Asthma limiting activities	0
663P0	Asthma limits activities 1 to 2 times per month	0
663P1	Asthma limits activities 1 to 2 times per week	0
663P2	Asthma limits activities most days	0
663Q.	Asthma not limiting activities	0
663U.	Asthma management plan given	0
663V.	Asthma severity	0
663V0	Occasional asthma	0
663V1	Mild asthma	0
663V2	Moderate asthma	0
663V3	Severe asthma	0
663W.	Asthma prophylactic medication used	0
663d.	Emergency asthma admission since last appointment	0
663e.	Asthma restricts exercise	0
663e0	Asthma sometimes restricts exercise	0
663e1	Asthma severely restricts exercise	0
663f.	Asthma never restricts exercise	0
663h.	Asthma - currently dormant	0
663j.	Asthma - currently active	0
663m.	Asthma accident and emergency attendance since last visit	0
663n.	Asthma treatment compliance satisfactory	0
663p.	Asthma treatment compliance unsatisfactory	0
663q.	Asthma daytime symptoms	0
663r.	Asthma causes night symptoms 1 to 2 times per month	0
663s.	Asthma never causes daytime symptoms	0
663t.	Asthma causes daytime symptoms 1 to 2 times per month	0
663u.	Asthma causes daytime symptoms 1 to 2 times per week	0
663v.	Asthma causes daytime symptoms most days	0
663w.	Asthma limits walking up hills or stairs	0
663x.	Asthma limits walking on the flat	0
663y.	Number of asthma exacerbations in past year	0
66Y5.	Change in asthma management plan	0
66Y9.	Step up change in asthma management plan	0
66YA.	Step down change in asthma management plan	0
66YC.	Absent from work or school due to asthma	0
66YE.	Asthma monitoring due	0
66YJ.	Asthma annual review	0
66YK.	Asthma follow-up	0
66YP.	Asthma night-time symptoms	0
66YQ.	Asthma monitoring by nurse	0
66YR.	Asthma monitoring by doctor	0
66YZ.	Does not have asthma management plan	0
66Yp.	Asthma review using Royal College of Physicians three questions	0
66Yq.	Asthma causes night time symptoms 1 to 2 times per week	0
66Yr.	Asthma causes symptoms most nights	0
66Ys.	Asthma never causes night symptoms	0
66Yu.	Number of days absent from school due to asthma in past 6 months	0
66Yz0	Asthma management plan declined	0
66Yz5	Telehealth asthma monitoring	0
679J.	Health education - asthma	0

679J0	Health education - asthma self management	0
679J1	Health education - structured asthma discussion	0
679J2	Health education - structured patient focused asthma discussion	0
68C3.	Asthma screening	0
6AP..	Review of patient at risk of asthma	0
8791.	Further asthma - drug prevent.	0
8793.	Asthma control step 0	0
8794.	Asthma control step 1	0
8795.	Asthma control step 2	0
8796.	Asthma control step 3	0
8797.	Asthma control step 4	0
8798.	Asthma control step 5	0
8B3j.	Asthma medication review	0
8CMA0	Patient has a written asthma personal action plan	0
8CR0.	Asthma clinical management plan	0
8H2P.	Emergency admission, asthma	0
9N1d.	Seen in asthma clinic	0
9N1d0	Seen in school asthma clinic	0
9N4Q.	DNA - Did not attend asthma clinic	0
9NI8.	Asthma outreach clinic	0
9NNX.	Under care of asthma specialist nurse	0
9OJ..	Asthma monitoring admin.	0
9OJ1.	Attends asthma monitoring	0
9OJ2.	Refuses asthma monitoring	0
9OJ3.	Asthma monitor offer default	0
9OJ4.	Asthma monitor 1st letter	0
9OJ5.	Asthma monitor 2nd letter	0
9OJ6.	Asthma monitor 3rd letter	0
9OJ7.	Asthma monitor verbal invite	0
9OJ8.	Asthma monitor phone invite	0
9OJ9.	Asthma monitoring deleted	0
9OJA.	Asthma monitoring check done	0
9OJB.	Asthma monitoring invitation SMS (short message service) text message	0
9OJB0	Asthma monitoring SMS (short message service) text message first invitation	0
9OJB1	Asthma monitoring SMS (short message service) text message second invitation	0
9OJB2	Asthma monitoring SMS (short message service) text message third invitation	0
9OJC.	Asthma monitoring invitation email	0
9OJZ.	Asthma monitoring admin.NOS	0
9Oj0.	Hypothyroidism monitoring first letter	0
9Q21.	Patient in asthma study	0
9hA..	Exception reporting: asthma quality indicators	0
9hA1.	Excepted from asthma quality indicators: Patient unsuitable	0
9hA2.	Excepted from asthma quality indicators: Informed dissent	0
G581.	Left ventricular failure	0
H3120	Chronic asthmatic bronchitis	0
H33..	Asthma	0
H330.	Extrinsic (atopic) asthma	0
H3300	Extrinsic asthma without status asthmaticus	0
H3301	Extrinsic asthma with status asthmaticus	0
H330z	Extrinsic asthma NOS	0
H331.	Intrinsic asthma	0
H3310	Intrinsic asthma without status asthmaticus	0
H3311	Intrinsic asthma with status asthmaticus	0
H331z	Intrinsic asthma NOS	0
H332.	Mixed asthma	0
H333.	Acute exacerbation of asthma	0
H334.	Brittle asthma	0
H335.	Chronic asthma with fixed airflow obstruction	0
H33z.	Asthma unspecified	0
H33z0	Status asthmaticus NOS	0
H33z1	Asthma attack	0
H33z2	Late-onset asthma	0
H33zz	Asthma NOS	0
H35y6	Sequoiosis (red-cedar asthma)	0
H35y7	Wood asthma	0
H3B..	Asthma-chronic obstructive pulmonary disease overlap syndrome	0
H47y0	Detergent asthma	0
SLF7.	Antiasthmatic poisoning	0
SLF7z	Antiasthmatic poisoning NOS	0
TJF7.	Adverse reaction to antiasthmatics	0
TJF73	Adverse reaction to theophylline (asthma)	0
TJF7z	Adverse reaction to antiasthmatic NOS	0
U60F6	[X]Antiasthmatics causing adverse effects in therapeutic use, not elsewhere classified	0

CONCEPT: Atopic Eczema

READ_CODE	DESCRIPTION_SAIL	SENSITIVE_CODE
14F1.	H/O: eczema	0
M07y.	Other specified local infection of skin or subcutaneous tissue	0
M102.	Infectious eczematoid dermatitis	0
M11..	Atopic dermatitis and related conditions	0
M111.	Atopic dermatitis/eczema	0
M113.	Flexural eczema	0
M114.	Allergic (intrinsic) eczema	0
M115.	Besnier's prurigo	0
M117.	Neurodermatitis - atopic	0
M11z.	Atopic dermatitis NOS	0
M12z1	Eczema NOS	0
M12z2	Infected eczema	0
M12z4	Erythrodermic eczema	0
Myu2.	[X]Dermatitis and eczema	0
Myu22	[X]Exacerbation of eczema	0

CONCEPT: Autism and ADHD

READ_CODE	DESCRIPTION_SAIL	SENSITIVE_CODE
6A61.	Attention deficit hyperactivity disorder annual review	0
8BPT.	Drug therapy for ADHD (attention deficit hyperactivity disorder)	0
8BPT0	Stimulant drug therapy for ADHD (attention deficit hyperactivity disorder)	0
8BPT1	Non-stimulant drug therapy for ADHD (attention deficit hyperactivity disorder)	0
9Ngp.	On drug therapy for ADHD (attention deficit hyperactivity disorder)	0
9Ngp0	On stimulant drug therapy for ADHD (attention deficit hyperactivity disorder)	0
9Ngp1	On non-stimulant drug therapy for ADHD (attention deficit hyperactivity disorder)	0

9OI8.	Attention deficit hyperactivity disorder monitoring invitation first letter	0
9OI9.	Attention deficit hyperactivity disorder monitoring invitation second letter	0
9OIA.	Attention deficit hyperactivity disorder monitoring invitation third letter	0
E140.	Infantile autism	0
E1400	Active infantile autism	0
E1401	Residual infantile autism	0
E140z	Infantile autism NOS	0
E2E..	Childhood hyperkinetic syndrome	0
E2E0.	Child attention deficit disorder	0
E2E01	Attention deficit with hyperactivity	0
E2E0z	Child attention deficit disorder NOS	0
E2E1.	Hyperkinesis with developmental delay	0
Eu840	[X]Childhood autism	0
Eu841	[X]Atypical autism	0
Eu845	[X]Asperger's syndrome	0
Eu84z	[X]Pervasive developmental disorder, unspecified	0
Eu900	[X]Disturbance of activity and attention	0
Eu902	[X]Deficits in attention, motor control and perception	0
Eu9y7	[X]Attention deficit disorder	0

CONCEPT: Bipolar Disorder

READ_CODE	DESCRIPTION_SAIL	SENSITIVE_CODE
146D.	H/O: manic depressive disorder	0
212T.	Psychosis, schizophrenia and bipolar affective disorder resolved	0
212V.	Bipolar affective disorder resolved	0
E11..	Affective psychoses	0
E110.	Manic disorder, single episode	0
E1100	Single manic episode, unspecified	0
E1101	Single manic episode, mild	0
E1102	Single manic episode, moderate	0
E1103	Single manic episode, severe without mention of psychosis	0
E1104	Single manic episode, severe, with psychosis	0
E1105	Single manic episode in partial or unspecified remission	0
E1106	Single manic episode in full remission	0
E110z	Manic disorder, single episode NOS	0
E111.	Recurrent manic episodes	0
E1110	Recurrent manic episodes, unspecified	0
E1111	Recurrent manic episodes, mild	0
E1112	Recurrent manic episodes, moderate	0
E1113	Recurrent manic episodes, severe without mention of psychosis	0
E1114	Recurrent manic episodes, severe, with psychosis	0
E1115	Recurrent manic episodes, in partial or unspecified remission	0
E1116	Recurrent manic episodes, in full remission	0
E111z	Recurrent manic episode NOS	0
E114.	Bipolar affective disorder, currently manic	0
E1140	Bipolar affective disorder, currently manic, unspecified	0
E1141	Bipolar affective disorder, currently manic, mild	0
E1142	Bipolar affective disorder, currently manic, moderate	0
E1143	Bipolar affective disorder, currently manic, severe, without mention of psychosis	0
E1144	Bipolar affective disorder, currently manic, severe, with psychosis	0
E1145	Bipolar affective disorder, currently manic, in partial or unspecified remission	0
E1146	Bipolar affective disorder, currently manic, in full remission	0
E114z	Bipolar affective disorder, currently manic, NOS	0
E115.	Bipolar affective disorder, currently depressed	0
E1150	Bipolar affective disorder, currently depressed, unspecified	0
E1151	Bipolar affective disorder, currently depressed, mild	0
E1152	Bipolar affective disorder, currently depressed, moderate	0
E1153	Bipolar affective disorder, currently depressed, severe, without mention of psychosis	0
E1154	Bipolar affective disorder, currently depressed, severe, with psychosis	0
E1155	Bipolar affective disorder, currently depressed, in partial or unspecified remission	0
E1156	Bipolar affective disorder, currently depressed, in full remission	0
E115z	Bipolar affective disorder, currently depressed, NOS	0
E116.	Mixed bipolar affective disorder	0
E1160	Mixed bipolar affective disorder, unspecified	0
E1161	Mixed bipolar affective disorder, mild	0
E1162	Mixed bipolar affective disorder, moderate	0
E1163	Mixed bipolar affective disorder, severe, without mention of psychosis	0
E1164	Mixed bipolar affective disorder, severe, with psychosis	0
E1165	Mixed bipolar affective disorder, in partial or unspecified remission	0
E1166	Mixed bipolar affective disorder, in full remission	0
E116z	Mixed bipolar affective disorder, NOS	0
E117.	Unspecified bipolar affective disorder	0
E1170	Unspecified bipolar affective disorder, unspecified	0
E1171	Unspecified bipolar affective disorder, mild	0
E1172	Unspecified bipolar affective disorder, moderate	0
E1173	Unspecified bipolar affective disorder, severe, without mention of psychosis	0
E1174	Unspecified bipolar affective disorder, severe, with psychosis	0
E1175	Unspecified bipolar affective disorder, in partial or unspecified remission	0
E1176	Unspecified bipolar affective disorder, in full remission	0
E117z	Unspecified bipolar affective disorder, NOS	0
E11y.	Other and unspecified manic-depressive psychoses	0
E11y0	Unspecified manic-depressive psychoses	0
E11y3	Other mixed manic-depressive psychoses	0
E11yz	Other and unspecified manic-depressive psychoses NOS	0
Eu30.	[X]Manic episode	0
Eu301	[X]Mania without psychotic symptoms	0
Eu302	[X]Mania with psychotic symptoms	0
Eu30y	[X]Other manic episodes	0
Eu30z	[X]Manic episode, unspecified	0
Eu31.	[X]Bipolar affective disorder	0
Eu310	[X]Bipolar affective disorder, current episode hypomanic	0
Eu311	[X]Bipolar affective disorder, current episode manic without psychotic symptoms	0
Eu312	[X]Bipolar affective disorder, current episode manic with psychotic symptoms	0
Eu313	[X]Bipolar affective disorder, current episode mild or moderate depression	0
Eu314	[X]Bipolar affective disorder, current episode severe depression without psychotic symptoms	0
Eu315	[X]Bipolar affective disorder, current episode severe depression with psychotic symptoms	0
Eu316	[X]Bipolar affective disorder, current episode mixed	0
Eu317	[X]Bipolar affective disorder, currently in remission	0
Eu318	[X]Bipolar affective disorder type I	0
Eu319	[X]Bipolar affective disorder type II	0
Eu31y	[X]Other bipolar affective disorders	0
Eu31z	[X]Bipolar affective disorder, unspecified	0

Eu332	[X]Recurrent depressive disorder, current episode severe without psychotic symptoms	0
Eu333	[X]Recurrent depressive disorder, current episode severe with psychotic symptoms	0
Eu340	[X]Cyclothymia	0
ZV111	[V]Personal history of affective disorder	0

CONCEPT: Blindness and low vision

READ_CODE	DESCRIPTION_SAIL	SENSITIVE_CODE
13o8J	Uses deafblind intervener	0
13o8N	Uses Deafblind Manual Alphabet	0
1B75.	Loss of vision	0
1B77.	Deteriorating vision	0
1a000	Uses guide dog for the blind	0
2B6B.	O/E - R-eye completely blind	0
2B6S.	O/E - pinhole R-eye completely blind	0
2B7B.	O/E - L-eye completely blind	0
2B7S.	O/E - pinhole L-eye completely blind	0
2BBr.	Impaired vision due to diabetic retinopathy	0
6688.	Registered partially sighted	0
6689.	Registered blind	0
668B.	Poor visual acuity	0
668C.	Certificate of vision impairment	0
668D.	Registered sight impaired	0
8F6..	Specific disability rehab.	0
8F61.	Blind rehabilitation	0
8F62.	Blind lead dog rehabilitation	0
9NfB.	Requires deafblind communicator guide	0
9Nfa.	Requires deafblind manual alphabet interpreter	0
9Nfb.	Requires deafblind block alphabet interpreter	0
9Nfc.	Requires deafblind haptic communication interpreter	0
9m08.	Excluded from diabetic retinopathy screening as blind	0
C245.	Vitamin A deficiency with night blindness	0
E2011	Hysterical blindness	0
F4041	Blind hypotensive eye	0
F4042	Blind hypertensive eye	0
F4653	After-cataract with vision obscured	0
F4811	Sudden visual loss	0
F4814	Other transient visual loss	0
F486.	Night blindness	0
F4860	Unspecified night blindness	0
F4862	Hereditary night blindness NOS	0
F4863	Congenital night blindness NOS	0
F4864	Acquired night blindness	0
F486z	Night blindness NOS	0
F49..	Blindness and low vision	0
F490.	Blindness, both eyes	0
F4900	Unspecified blindness both eyes	0
F4901	Both eyes total visual impairment	0
F4902	Better eye: near total visual impairment, Lesser eye: unspecified	0
F4903	Better eye: near total visual impairment, Lesser eye: total visual impairment	0
F4904	Better eye: near total visual impairment, Lesser eye: near total visual impairment	0
F4905	Better eye: profound visual impairment, Lesser eye: unspecified	0
F4906	Better eye: profound visual impairment, Lesser eye: total visual impairment	0
F4907	Better eye: profound visual impairment, Lesser eye: near total visual impairment	0
F4908	Better eye: profound visual impairment, Lesser eye: profound visual impairment	0
F4909	Acquired blindness, both eyes	0
F490z	Blindness both eyes NOS	0
F491.	Better eye: low vision, Lesser eye: profound visual impairment	0
F4910	One eye blind, one eye low vision	0
F4911	Better eye: severe visual impairment, Lesser eye: blind, unspecified	0
F4912	Better eye: severe visual impairment, Lesser eye: total visual impairment	0
F4913	Better eye: severe visual impairment, Lesser eye: near total visual impairment	0
F4914	Better eye: severe visual impairment, Lesser eye: profound visual impairment	0
F4915	Better eye: moderate visual impairment, Lesser eye: blind, unspecified	0
F4916	Better eye: moderate visual impairment, Lesser eye: total visual impairment	0
F4917	Better eye: moderate visual impairment, Lesser eye: near total visual impairment	0
F4918	Better eye: moderate visual impairment, Lesser eye: profound visual impairment	0
F491z	One eye blind, one eye low vision NOS	0
F492.	Low vision, both eyes	0
F4920	Low vision, both eyes unspecified	0
F4921	Better eye: severe visual impairment, Lesser eye: low vision unspecified	0
F4922	Better eye: severe visual impairment, Lesser eye: severe visual impairment	0
F4923	Better eye: moderate visual impairment, Lesser eye: low vision unspecified	0
F4924	Better eye: moderate visual impairment, Lesser eye: severe visual impairment	0
F4925	Better eye: moderate visual impairment, Lesser eye: moderate visual impairment	0
F492z	Low vision, both eyes NOS	0
F493.	Visual loss, both eyes unqualified	0
F494.	Legal blindness USA	0
F495.	Profound impairment, one eye	0
F4950	Blindness, one eye, unspecified	0
F4951	Lesser eye: total visual impairment, Better eye: unspecified	0
F4952	Lesser eye: total visual impairment, Better eye: near normal vision	0
F4953	Lesser eye: total visual impairment, Better eye: normal vision	0
F4954	Lesser eye: near total visual impairment, Better eye: unspecified	0
F4955	Lesser eye: near total visual impairment, Better eye: near normal vision	0
F4956	Lesser eye: near total visual impairment, Better eye: normal vision	0
F4957	Lesser eye: profound visual impairment, Better eye: unspecified	0
F4958	Lesser eye: profound visual impairment, Better eye: near normal vision	0
F4959	Lesser eye: profound visual impairment, Better eye: normal vision	0
F495A	Acquired blindness, one eye	0
F495z	Profound impairment one eye NOS	0
F496.	Low vision, one eye	0
F4960	Low vision, one eye, unspecified	0
F4961	Lesser eye: severe visual impairment, Better eye: unspecified	0
F4962	Lesser eye: severe visual impairment, Better eye: near normal vision	0
F4963	Lesser eye: severe visual impairment, Better eye: normal vision	0
F4964	Lesser eye: moderate visual impairment, Better eye: unspecified	0
F4965	Lesser eye: moderate visual impairment, Better eye: near normal vision	0
F4966	Lesser eye: moderate visual impairment, Better eye: normal vision	0
F496z	Low vision, one eye NOS	0
F497.	Severe visual impairment, binocular	0
F49D.	Visual impairment	0
F49y.	Visual loss, one eye, unqualified	0

F49z.	Visual loss NOS	0
F4H73	Cortical blindness	0
Fy1..	Combined visual and hearing impairment	0
FyuL.	[X]Visual disturbances and blindness	0
P3410	Congenital corneal opacity with visual deficit	0
P3411	Congenital corneal opacity without visual deficit	0
SJ0z.	Optic nerve or pathway injury NOS	0

CONCEPT: Breast Cancer

READ_CODE	DESCRIPTION_SAIL	SENSITIVE_CODE
B34..	Malignant neoplasm of female breast	0
B340.	Malignant neoplasm of nipple and areola of female breast	0
B3400	Malignant neoplasm of nipple of female breast	0
B3401	Malignant neoplasm of areola of female breast	0
B340z	Malignant neoplasm of nipple or areola of female breast NOS	0
B341.	Malignant neoplasm of central part of female breast	0
B342.	Malignant neoplasm of upper-inner quadrant of female breast	0
B343.	Malignant neoplasm of lower-inner quadrant of female breast	0
B344.	Malignant neoplasm of upper-outer quadrant of female breast	0
B345.	Malignant neoplasm of lower-outer quadrant of female breast	0
B346.	Malignant neoplasm of axillary tail of female breast	0
B347.	Malignant neoplasm, overlapping lesion of breast	0
B34y.	Malignant neoplasm of other site of female breast	0
B34yz	Malignant neoplasm of other site of female breast NOS	0
B34z.	Malignant neoplasm of female breast NOS	0
B35..	Malignant neoplasm of male breast	0
B350.	Malignant neoplasm of nipple and areola of male breast	0
B3500	Malignant neoplasm of nipple of male breast	0
B3501	Malignant neoplasm of areola of male breast	0
B350z	Malignant neoplasm of nipple or areola of male breast NOS	0
B35z.	Malignant neoplasm of other site of male breast	0
B35zz	Malignant neoplasm of male breast NOS	0
B36..	Local recurrence of malignant tumour of breast	0
B77..	Benign neoplasm of breast	0
B770.	Benign neoplasm of female breast	0
B771.	Benign neoplasm of male breast	0
B77z.	Benign neoplasm of breast NOS	0
B83..	Carcinoma in situ of breast and genitourinary system	0
B830.	Carcinoma in situ of breast	0
B8300	Lobular carcinoma in situ of breast	0
B8301	Intraductal carcinoma in situ of breast	0
BB94.	[M]Juvenile breast carcinoma	0
BB9J.	[M]Paget's disease, mammary	0
BB9K.	[M]Paget's disease and infiltrating breast duct carcinoma	0
BB9K0	[M]Paget's disease and intraductal carcinoma of breast	0
Byu6.	[X]Malignant neoplasm of breast	0
ByuFG	[X]Other carcinoma in situ of breast	0
ZV103	[V]Personal history of malignant neoplasm of breast	0
ZV13A	[V]Personal history of non-neoplastic breast disease	0

CONCEPT: Bronchiectasis

READ_CODE	DESCRIPTION_SAIL	SENSITIVE_CODE
14BA.	H/O: bronchiectasis	0
A115.	Tuberculous bronchiectasis	0
H34..	Bronchiectasis	0
H340.	Recurrent bronchiectasis	0
H341.	Post-infective bronchiectasis	0
H34z.	Bronchiectasis NOS	0
P861.	Congenital bronchiectasis	0

CONCEPT: Chronic Back Pain

READ_CODE	DESCRIPTION_SAIL	SENSITIVE_CODE
16C9.	Chronic low back pain	0
8HTH.	Referral to back pain clinic	0

CONCEPT: Chronic Fatigue Syndrome

READ_CODE	DESCRIPTION_SAIL	SENSITIVE_CODE
F03y.	Other causes of encephalitis	0
F286.	Chronic fatigue syndrome	0
F2860	Mild chronic fatigue syndrome	0
F2861	Moderate chronic fatigue syndrome	0
F2862	Severe chronic fatigue syndrome	0
G8522	Oesophageal varices in cirrhosis of the liver	0
J601.	Subacute necrosis of liver	0
J6010	Subacute hepatic failure	0
J6011	Subacute hepatitis - noninfective	0
J6012	Subacute yellow atrophy	0
J601z	Subacute necrosis of liver NOS	0
J60z.	Acute and subacute liver necrosis NOS	0
J61..	Cirrhosis and chronic liver disease	0
J614.	Chronic hepatitis	0
J6140	Chronic persistent hepatitis	0
J6141	Chronic active hepatitis	0
J6142	Chronic aggressive hepatitis	0
J6143	Recurrent hepatitis	0
J6144	Chronic lobular hepatitis	0
J614y	Chronic hepatitis unspecified	0
J614z	Chronic hepatitis NOS	0
J615.	Cirrhosis - non alcoholic	0
J6150	Unilobular portal cirrhosis	0
J6151	Multilobular portal cirrhosis	0
J6152	Mixed portal cirrhosis	0
J6153	Diffuse nodular cirrhosis	0
J6154	Fatty portal cirrhosis	0
J6155	Hypertrophic portal cirrhosis	0
J6156	Capsular portal cirrhosis	0
J6157	Cardiac portal cirrhosis	0
J6158	Juvenile portal cirrhosis	0
J6159	Pigmentary portal cirrhosis	0
J615A	Pipe-stem portal cirrhosis	0

J615B	Toxic portal cirrhosis	0
J615C	Xanthomatous portal cirrhosis	0
J615D	Bacterial portal cirrhosis	0
J615E	Cardituberculous cirrhosis	0
J615F	Syphilitic portal cirrhosis	1
J615G	Zooparasitic portal cirrhosis	0
J615H	Infectious cirrhosis NOS	0
J615y	Portal cirrhosis unspecified	0
J615z	Non-alcoholic cirrhosis NOS	0
J616.	Biliary cirrhosis	0
J6160	Primary biliary cirrhosis	0
J6161	Secondary biliary cirrhosis	0
J6162	Biliary cirrhosis of children	0
J616z	Biliary cirrhosis NOS	0
J617.	Alcoholic hepatitis	0
J6170	Chronic alcoholic hepatitis	0
J61y.	Other non-alcoholic chronic liver disease	0
J61y0	Chronic yellow liver atrophy	0
J61y1	Non-alcoholic fatty liver	0
J61y2	Hepatosplenomegaly	0
J61y3	Portal fibrosis without cirrhosis	0
J61y4	Hepatic fibrosis	0
J61y5	Hepatic sclerosis	0
J61y6	Hepatic fibrosis with hepatic sclerosis	0
J61y7	Steatosis of liver	0
J61y8	Nonalcoholic steatohepatitis	0
J61yz	Other non-alcoholic chronic liver disease NOS	0
J61z.	Chronic liver disease NOS	0
J623.	Portal hypertension	0
J624.	Hepatorenal syndrome	0
J62y.	Other sequelae of chronic liver disease	0
J62z.	Liver abscess and chronic liver disease causing sequelae NOS	0
J630.	Chronic passive liver congestion	0
Jyu71	[X]Other and unspecified cirrhosis of liver	0
PB62.	Congenital cystic liver disease	0
PB620	Congenital polycystic liver disease	0

CONCEPT: Chronic Liver Disease

READ_CODE	DESCRIPTION_SAIL	SENSITIVE_CODE
141E.	History of hepatitis B	1
14i..	H/O hepatitis C antiviral drug therapy	1
21267	Hepatitis C resolved	1
2J12.	Hepatitis C non immune	0
43B4.	Hepatitis B surface antig +ve	1
43B6.	Hepatitis B non immune	0
43B7.	Hepatitis C non-immune	0
43X3.	Hepatitis C antibody test positive	1
4JQ3.	Hepatitis C virus genotype	1
4JQD.	Hepatitis C viral ribonucleic acid polymerase chain reaction positive	1
4JQF.	Hepatitis C antigen positive	1
7609.	Open operations on oesophageal varices	0
76093	Local ligation of oesophageal varices	0
76094	Open injection sclerotherapy to oesophageal varices	0
7609y	Other specified open operation on oesophageal varices	0
7609z	Open operation on oesophageal varices NOS	0
760C5	Fibreoptic endoscopic banding of oesophageal varices	0
760F3	Endoscopic injection sclerotherapy to varices of oesophagus using rigid oesophagoscope	0
760F4	Rigid oesophagoscopic banding of oesophageal varices	0
7L1f.	Compensation for liver failure	0
7L1fz	Compensation for liver failure NOS	0
7Q052	Hepatitis B treatment drugs Band 1	1
7Q053	Respiratory syncytial virus treatment and Hepatitis C treatment drugs Band 1	0
8BB5.	12 week virologic response to hepatitis C treatment	1
9NgR.	On hepatitis C treatment plan	1
9kR..	Chronic hepatitis annual review - enhanced services administration	1
9kV..	Hepatitis C screening positive - enhanced services administration	0
9kZ..	Hepatitis B screening positive - enhanced services administration	1
A702.	Viral hepatitis B with coma	0
A703.	Viral (serum) hepatitis B	1
A7040	Viral hepatitis C with coma	0
A7050	Viral hepatitis C without mention of hepatic coma	1
A7051	Acute delta-(super)infection of hepatitis B carrier	1
A707.	Chronic viral hepatitis	1
A7070	Chronic viral hepatitis B with delta-agent	1
A7071	Chronic viral hepatitis B without delta-agent	1
A7072	Chronic viral hepatitis C	1
A7073	Chronic viral hepatitis B	1
A707X	Chronic viral hepatitis, unspecified	1
A70A.	Hepatitis C genotype 1	1
A70B.	Hepatitis C genotype 2	1
A70C.	Hepatitis C genotype 3	1
A70D.	Hepatitis C genotype 4	1
A70E.	Hepatitis C genotype 5	0
A70F.	Hepatitis C genotype 6	0
A70G.	Acute hepatitis C	0
A70z0	Hepatitis C	1
AyuB1	[X]Other chronic viral hepatitis	1
AyuB2	[X]Chronic viral hepatitis, unspecified	1
C3104	Glycogenosis with hepatic cirrhosis	0
C3500	Haemochromatosis	0
C3707	Liver disease due to cystic fibrosis	0
G85..	Other varicose veins	0
G850.	Oesophageal varices with bleeding	0
G851.	Oesophageal varices without bleeding	0
G852.	Oesophageal varices in diseases EC	0
G8520	Oesophageal varices with bleeding in diseases EC	0
G8521	Oesophageal varices without bleeding in diseases EC	0
G8522	Oesophageal varices in cirrhosis of the liver	0
G8523	Oesophageal varices in alcoholic cirrhosis of the liver	0
G852z	Oesophageal varices in diseases EC NOS	0
G858.	Oesophageal varices NOS	0
Gyu94	[X]Oesophageal varices in diseases classified elsewhere	0

J61..	Cirrhosis and chronic liver disease	0
J610.	Alcoholic fatty liver	0
J611.	Acute alcoholic hepatitis	0
J612.	Alcoholic cirrhosis of liver	0
J6120	Alcoholic fibrosis and sclerosis of liver	0
J613.	Alcoholic liver damage unspecified	0
J6130	Alcoholic hepatic failure	0
J614.	Chronic hepatitis	0
J6140	Chronic persistent hepatitis	0
J6141	Chronic active hepatitis	0
J6142	Chronic aggressive hepatitis	0
J6143	Recurrent hepatitis	0
J6144	Chronic lobular hepatitis	0
J614y	Chronic hepatitis unspecified	0
J614z	Chronic hepatitis NOS	0
J615.	Cirrhosis - non alcoholic	0
J6150	Unilobular portal cirrhosis	0
J6151	Multilobular portal cirrhosis	0
J6152	Mixed portal cirrhosis	0
J6153	Diffuse nodular cirrhosis	0
J6154	Fatty portal cirrhosis	0
J6155	Hypertrophic portal cirrhosis	0
J6156	Capsular portal cirrhosis	0
J6157	Cardiac portal cirrhosis	0
J6158	Juvenile portal cirrhosis	0
J6159	Pigmentary portal cirrhosis	0
J615A	Pipe-stem portal cirrhosis	0
J615B	Toxic portal cirrhosis	0
J615C	Xanthomatous portal cirrhosis	0
J615D	Bacterial portal cirrhosis	0
J615E	Cardituberculous cirrhosis	0
J615F	Syphilitic portal cirrhosis	1
J615G	Zooparasitic portal cirrhosis	0
J615H	Infectious cirrhosis NOS	0
J615y	Portal cirrhosis unspecified	0
J615z	Non-alcoholic cirrhosis NOS	0
J616.	Biliary cirrhosis	0
J6160	Primary biliary cirrhosis	0
J6161	Secondary biliary cirrhosis	0
J6162	Biliary cirrhosis of children	0
J616z	Biliary cirrhosis NOS	0
J617.	Alcoholic hepatitis	0
J6170	Chronic alcoholic hepatitis	0
J61y.	Other non-alcoholic chronic liver disease	0
J61y0	Chronic yellow liver atrophy	0
J61y1	Non-alcoholic fatty liver	0
J61y3	Portal fibrosis without cirrhosis	0
J61y4	Hepatic fibrosis	0
J61y5	Hepatic sclerosis	0
J61y6	Hepatic fibrosis with hepatic sclerosis	0
J61y8	Nonalcoholic steatohepatitis	0
J61yz	Other non-alcoholic chronic liver disease NOS	0
J61z.	Chronic liver disease NOS	0
J62..	Liver abscess and sequelae of chronic liver disease	0
J623.	Portal hypertension	0
J624.	Hepatorenal syndrome	0
J625.	[X] Hepatic failure	0
J62y.	Other sequelae of chronic liver disease	0
J62z.	Liver abscess and chronic liver disease causing sequelae NOS	0
J6353	Toxic liver disease with chronic persistent hepatitis	0
J6354	Toxic liver disease with chronic lobular hepatitis	0
J6355	Toxic liver disease with chronic active hepatitis	0
J6356	Toxic liver disease with fibrosis and cirrhosis of liver	0
J63B.	Autoimmune hepatitis	0
J6617	Primary sclerosing cholangitis	0
Jyu71	[X]Other and unspecified cirrhosis of liver	0
PB621	Fibrocystic liver disease	0
Q4091	Congenital hepatitis B infection	1
Q48yz	Other perinatal condition NOS	0
SP142	Hepatic failure as a complication of care	0
SP143	Hepatorenal syndrome as a complication of care	0
ZV02B	[V]Hepatitis B carrier	1
ZV02C	[V]Hepatitis C carrier	1

CONCEPT: Chronic Pain

READ_CODE	DESCRIPTION_SAIL	SENSITIVE_CODE
16C9.	Chronic low back pain	0
1M52.	Chronic pain	0
8HTH.	Referral to back pain clinic	0
F347.	Complex regional pain syndrome type II	0
F369.	Complex regional pain syndrome	0
N337.	Algoneurodystrophy	0
N3370	Disuse atrophy of bone	0
N3371	Sudek's atrophy	0
N3372	Algodystrophy of hand	0
N3373	Algodystrophy of knee	0
N3374	Algodystrophy of foot	0
N337z	Algoneurodystrophy NOS	0
N33C.	Complex regional pain syndrome type I	0
R00zC	[D]Chronic intractable pain	0
Ryu70	[X]Other chronic pain	0

CONCEPT: Chronic Sinusitis

READ_CODE	DESCRIPTION_SAIL	SENSITIVE_CODE
H13..	Chronic sinusitis	0
H130.	Chronic maxillary sinusitis	0
H131.	Chronic frontal sinusitis	0
H132.	Chronic ethmoidal sinusitis	0
H133.	Chronic sphenoidal sinusitis	0
H134.	Fistula of nasal sinus	0
H135.	Recurrent sinusitis	0

H13y.	Other chronic sinusitis	0
H13y0	Chronic pansinusitis	0
H13y1	Pansinusitis	0
H13yz	Other chronic sinusitis NOS	0
H13z.	Chronic sinusitis NOS	0
Hyu22	[X]Other chronic sinusitis	0
J0835	Oroantral fistula	0

CONCEPT: Chronic Kidney Disease (CKD) Stage 3_5

READ_CODE	DESCRIPTION_SAIL	SENSITIVE_CODE
1Z12.	Chronic kidney disease stage 3	0
1Z13.	Chronic kidney disease stage 4	0
1Z14.	Chronic kidney disease stage 5	0
1Z15.	Chronic kidney disease stage 3A	0
1Z16.	Chronic kidney disease stage 3B	0
1Z1B.	Chronic kidney disease stage 3 with proteinuria	0
1Z1C.	Chronic kidney disease stage 3 without proteinuria	0
1Z1D.	Chronic kidney disease stage 3A with proteinuria	0
1Z1E.	Chronic kidney disease stage 3A without proteinuria	0
1Z1F.	Chronic kidney disease stage 3B with proteinuria	0
1Z1G.	Chronic kidney disease stage 3B without proteinuria	0
1Z1H.	Chronic kidney disease stage 4 with proteinuria	0
1Z1J.	Chronic kidney disease stage 4 without proteinuria	0
1Z1K.	Chronic kidney disease stage 5 with proteinuria	0
1Z1L.	Chronic kidney disease stage 5 without proteinuria	0
1Z1T.	CKD G3aA1 - chronic kidney disease with glomerular filtration rate category G3a and albuminuria category A1	0
1Z1V.	CKD G3aA2 - chronic kidney disease with glomerular filtration rate category G3a and albuminuria category A2	0
1Z1W.	CKD G3aA3 - chronic kidney disease with glomerular filtration rate category G3a and albuminuria category A3	0
1Z1X.	CKD G3bA1 - chronic kidney disease with glomerular filtration rate category G3b and albuminuria category A1	0
1Z1Y.	CKD G3bA2 - chronic kidney disease with glomerular filtration rate category G3b and albuminuria category A2	0
1Z1Z.	CKD G3bA3 - chronic kidney disease with glomerular filtration rate category G3b and albuminuria category A3	0
1Z1a.	CKD G4A1 - chronic kidney disease with glomerular filtration rate category G4 and albuminuria category A1	0
1Z1b.	CKD G4A2 - chronic kidney disease with glomerular filtration rate category G4 and albuminuria category A2	0
1Z1c.	CKD G4A3 - chronic kidney disease with glomerular filtration rate category G4 and albuminuria category A3	0
1Z1d.	CKD G5A1 - chronic kidney disease with glomerular filtration rate category G5 and albuminuria category A1	0
1Z1e.	CKD G5A2 - chronic kidney disease with glomerular filtration rate category G5 and albuminuria category A2	0
1Z1f.	CKD G5A3 - chronic kidney disease with glomerular filtration rate category G5 and albuminuria category A3	0
K05..	Chronic renal failure	0
K050.	End stage renal failure	0
K053.	Chronic kidney disease stage 3	0
K054.	Chronic kidney disease stage 4	0
K055.	Chronic kidney disease stage 5	0

CONCEPT: Coeliac Disease

READ_CODE	DESCRIPTION_SAIL	SENSITIVE_CODE
6648.	Coeliac disease monitoring	0
66480	Coeliac disease annual review	0
68W4.	Coeliac disease autoantibody profile positive	0
8IAp.	Coeliac disease annual review declined	0
9mB..	Coeliac disease monitoring invitation	0
9mB0.	Coeliac disease monitoring telephone invitation	0
9mB1.	Coeliac disease monitoring invitation first letter	0
9mB2.	Coeliac disease monitoring invitation second letter	0
9mB3.	Coeliac disease monitoring invitation third letter	0
J690.	Coeliac disease	0
J6900	Congenital coeliac disease	0
J6901	Acquired coeliac disease	0
J690z	Coeliac disease NOS	0

CONCEPT: Colon Cancer

READ_CODE	DESCRIPTION_SAIL	SENSITIVE_CODE
68W25	Bowel scope (flexible sigmoidoscopy) screen: suspected cancer detected	0
9Ow1.	Bowel cancer detected by national screening programme	0
B0720	Malignant neoplasm of pharyngeal recess	0
B13..	Malignant neoplasm of colon	0
B130.	Malignant neoplasm of hepatic flexure of colon	0
B131.	Malignant neoplasm of transverse colon	0
B132.	Malignant neoplasm of descending colon	0
B133.	Malignant neoplasm of sigmoid colon	0
B134.	Malignant neoplasm of caecum	0
B135.	Malignant neoplasm of appendix	0
B136.	Malignant neoplasm of ascending colon	0
B137.	Malignant neoplasm of splenic flexure of colon	0
B138.	Malignant neoplasm, overlapping lesion of colon	0
B139.	Hereditary nonpolyposis colon cancer	0
B13y.	Malignant neoplasm of other specified sites of colon	0
B13z.	Malignant neoplasm of colon NOS	0
B14..	Malignant neoplasm of rectum, rectosigmoid junction and anus	0
B140.	Malignant neoplasm of rectosigmoid junction	0
B141.	Malignant neoplasm of rectum	0
B142.	Malignant neoplasm of anal canal	0
B1420	Malignant neoplasm of cloacogenic zone	0
B143.	Malignant neoplasm of anus unspecified	0
B14y.	Malignant neoplasm of other sites of rectum, rectosigmoid junction and anus	0
B14z.	Malignant neoplasm of rectum, rectosigmoid junction and anus NOS	0
B18y2	Malignant neoplasm of mesorectum	0
B1z0.	Malignant neoplasm of intestinal tract, part unspecified	0
B575.	Secondary malignant neoplasm of large intestine and rectum	0
B5750	Secondary malignant neoplasm of colon	0
B5751	Secondary malignant neoplasm of rectum	0
B575z	Secondary malignant neoplasm of large intestine or rectum NOS	0
B803.	Carcinoma in situ of colon	0
B8030	Carcinoma in situ of hepatic flexure of colon	0
B8031	Carcinoma in situ of transverse colon	0
B8032	Carcinoma in situ of descending colon	0
B8033	Carcinoma in situ of sigmoid colon	0
B8036	Carcinoma in situ of ascending colon	0
B8037	Carcinoma in situ of splenic flexure of colon	0
B8038	High grade dysplasia of colon	0
B803z	Carcinoma in situ of colon NOS	0
B804.	Carcinoma in situ of rectum and rectosigmoid junction	0

B8040	Carcinoma in situ of rectosigmoid junction	0
B8041	Carcinoma in situ of rectum	0
B804z	Carcinoma in situ of rectum or rectosigmoid junction NOS	0
B902.	Neoplasm of uncertain behaviour of stomach, intestines and rectum	0
B9024	Neoplasm of uncertain behaviour of colon	0
B9025	Neoplasm of uncertain behaviour of rectum	0
B902z	Neoplasm of uncertain behaviour of stomach, intestine or rectum NOS	0
BB57.	[M]Adenocarcinoma, intestinal type	0
BB5N.	[M]Adenomatous and adenocarcinomatous polyps of colon	0
BB5Nz	[M]Adenomatous or adenocarcinomatous polyps of the colon NOS	0
PB5B.	Dysplasia of colon	0
ZV100	[V]Personal history of malignant neoplasm of gastrointestinal tract	0

CONCEPT: Congenital disease and chromosomal abnormalities

READ_CODE	DESCRIPTION_SAIL	SENSITIVE_CODE
12J3.	FH: Congenital CVS anomaly	0
14AV.	History of ventricular septal defect	0
21268	Ostium secundum atrial septal defect resolved	0
66g..	Congenital heart condition monitoring	0
7903y	Other specified atrial inversion operation for transposition of great vessels	0
79063	Closure of persistent ostium primum	0
790A1	Open atrial septostomy	0
790N4	Relief of left ventricular outflow tract obstruction	0
9RD0.	Transfer of care from paediatric congenital heart service	0
G5y9.	Cardiac septal defect, acquired	0
L185.	Congenital cardiovascular disorders during pregnancy, childbirth and the puerperium	0
L1850	Congenital cardiovascular disorder - unspecified whether during pregnancy or the puerperium	0
L1851	Congenital cardiovascular disorder during pregnancy - baby delivered	0
L1852	Congenital cardiovascular disorder in the puerperium - baby delivered during current episode of care	0
L1853	Congenital cardiovascular disorder during pregnancy - baby not yet delivered	0
L1854	Congenital cardiovascular disorder in the puerperium - baby delivered during previous episode of care	0
L185z	Congenital cardiovascular disorder during pregnancy, childbirth or the puerperium NOS	0
P5...	Bulbus cordis and cardiac septal closure anomalies	0
P50..	Common aorto-pulmonary trunk	0
P500.	Absent septum between aorta and pulmonary artery	0
P501.	Aortic septal defect	0
P502.	Persistent truncus arteriosus	0
P50z.	Common aorto-pulmonary trunk NOS	0
P51..	Transposition of great vessels	0
P510.	Total great vessel transposition	0
P511.	Double outlet right ventricle	0
P5110	Double outlet right ventricle, unspecified	0
P5111	Dextratransposition of aorta	0
P5112	Incomplete great vessel transposition	0
P5113	Taussig-Bing syndrome	0
P511z	Double outlet right ventricle NOS	0
P512.	Corrected great vessel transposition	0
P51y.	Other specified transposition of great vessels	0
P51z.	Great vessel transposition NOS	0
P52..	Tetralogy of Fallot	0
P520.	Tetralogy of Fallot, unspecified	0
P521.	Pentalogy of Fallot	0
P52z.	Tetralogy of Fallot NOS	0
P53..	Common ventricle	0
P54..	Ventricular septal defect	0
P540.	Ventricular septal defect, unspecified	0
P541.	Interventricular septal defect	0
P542.	Left ventricle to right atrial communication	0
P543.	Eisenmenger's complex	0
P544.	Gerbode's defect	0
P545.	Roger's disease	0
P54y.	Other specified ventricular septal defect	0
P54z.	Ventricular septal defect NOS	0
P55..	Ostium secundum atrial septal defect	0
P550.	Atrial septal defect NOS	0
P551.	Patent foramen ovale	0
P552.	Persistent ostium secundum	0
P553.	Lutembacher's syndrome	0
P55y.	Other specified ostium secundum atrial septal defect	0
P55z.	Ostium secundum atrial septal defect NOS	0
P56..	Endocardial cushion defects	0
P560.	Endocardial cushion defects, unspecified	0
P561.	Ostium primum defect	0
P56y.	Other specified endocardial cushion defects	0
P56z.	Endocardial cushion defects NOS	0
P56z0	Common atrium	0
P56z1	Common atrioventricular canal	0
P56z2	Common atrioventricular-type ventricular septal defect	0
P56zz	Endocardial cushion defects NOS	0
P57..	Cor biloculare	0
P58..	Double outlet left ventricle	0
P59..	Isomerism of atrial appendages	0
P5X..	Congenital malformations of cardiac chambers and connections	0
P5y..	Other heart bulb and septal closure defect	0
P5z..	Heart bulb or septal closure defects NOS	0
P6...	Other congenital heart anomalies	0
P60..	Pulmonary valve anomalies	0
P600.	Pulmonary valve anomaly, unspecified	0
P601.	Congenital atresia of the pulmonary valve	0
P6010	Hypoplasia of pulmonary valve	0
P601z	Congenital atresia of pulmonary valve NOS	0
P602.	Congenital pulmonary stenosis	0
P6020	Congenital fusion of pulmonic cusps	0
P6021	Congenital fusion of pulmonary valve segment	0
P602z	Congenital pulmonary stenosis NOS	0
P603.	Right hypoplastic heart syndrome	0
P60z.	Other pulmonary valve anomalies	0
P60z0	Congenital insufficiency of the pulmonary valve	0
P60z1	Fallot's trilogy	0
P60z2	Supernumerary pulmonary valve cusps	0
P60zz	Other pulmonary valve anomaly NOS	0
P61..	Congenital tricuspid atresia and stenosis	0

P610.	Congenital tricuspid atresia	0
P611.	Congenital tricuspid stenosis	0
P61z.	Congenital tricuspid atresia or stenosis NOS	0
P62..	Ebstein's anomaly	0
P63..	Congenital aortic valve stenosis	0
P64..	Congenital aortic valve insufficiency	0
P640.	Congenital aortic valve insufficiency, unspecified	0
P641.	Bicuspid aortic valve	0
P64z.	Congenital aortic valve insufficiency NOS	0
P65..	Congenital mitral stenosis	0
P650.	Congenital mitral stenosis, unspecified	0
P651.	Fused commissure of the mitral valve	0
P652.	Parachute deformity of the mitral valve	0
P653.	Supernumerary cusps of the mitral valve	0
P65z.	Congenital mitral stenosis NOS	0
P66..	Congenital mitral insufficiency	0
P67..	Hypoplastic left heart syndrome	0
P68..	Congenital heart disease	0
P69..	Left ventricular outflow tract obstruction	0
P6W..	Congenital malformation of aortic and mitral valves unspecified	0
P6X..	Congenital malformation of tricuspid valve, unspecified	0
P6y..	Other specified heart anomalies	0
P6y0.	Subaortic stenosis	0
P6y1.	Cor triatriatum	0
P6y2.	Pulmonary infundibular stenosis	0
P6y3.	Obstructive heart anomaly NEC	0
P6y30	Uhl's disease	0
P6y31	Right ventricular outflow tract obstruction	0
P6y3z	Obstructive heart anomaly NEC NOS	0
P6y4.	Coronary artery anomaly	0
P6y40	Congenital absence of coronary artery	0
P6y41	Single coronary artery	0
P6y42	Coronary artery from aorta	0
P6y43	Coronary artery from pulmonary trunk	0
P6y44	Anomalous coronary artery communication	0
P6y45	Congenital coronary aneurysm	0
P6y46	Congenital stricture of coronary artery	0
P6y4z	Coronary artery anomaly NOS	0
P6y5.	Congenital heart block	0
P6y50	Congenital heart block, unspecified	0
P6y51	Congenital complete atrio-ventricular heart block	0
P6y52	Congenital incomplete atrio-ventricular heart block	0
P6y5z	Congenital heart block NOS	0
P6y6.	Heart and cardiac apex malposition	0
P6y60	Dextrocardia	0
P6y61	Levocardia	0
P6y62	Mesocardia	0
P6y63	Ectopia cordis	0
P6y64	Abdominal heart	0
P6y6z	Heart or cardiac apex malposition NOS	0
P6y7.	Myocardial bridge of coronary artery	0
P6y8.	Congenital dextroposition of heart	0
P6yy.	Other specified heart anomalies	0
P6yy0	Atresia of cardiac vein	0
P6yy1	Hypoplasia of cardiac vein	0
P6yy2	Congenital cardiomegaly	0
P6yy3	Congenital left ventricular diverticulum	0
P6yy4	Congenital pericardial defect	0
P6yy5	Congenital anomaly of myocardium	0
P6yy6	Congenital aneurysm of heart	0
P6yy7	Atresia of heart valve NEC	0
P6yy8	Cor triloculare	0
P6yy9	Congenital epicardial cyst	0
P6yyA	Hemicardia	0
P6yyB	Supernumerary heart valve cusps NEC	0
P6yyC	Fusion of mitral valve cusps	0
P6yyD	Fusion of heart valve cusps NEC	0
P6yyz	Other specified heart anomalies NOS	0
P6z..	Congenital heart anomaly NOS	0
P6z0.	Unspecified anomaly of heart valve	0
P6z1.	Anomalous bands of heart	0
P6z10	Anomalous atrial bands	0
P6z11	Anomalous ventricular bands	0
P6z1z	Anomalous bands of heart NOS	0
P6z2.	Acyanotic congenital heart disease NOS	0
P6z3.	Cyanotic congenital heart disease NOS	0
P6zz.	Congenital heart anomaly NOS	0
P7...	Other congenital circulatory system anomalies	0
P70..	Patent ductus arteriosus	0
P71..	Coarctation of aorta	0
P710.	Hypoplasia of aortic arch, unspecified	0
P711.	Preductal coarctation of aorta	0
P712.	Postductal coarctation of aorta	0
P713.	Interruption of aortic arch	0
P71z.	Coarctation of aorta NOS	0
P72..	Other anomalies of aorta	0
P720.	Anomaly of aorta, unspecified	0
P721.	Aortic arch anomalies	0
P7210	Anomalous origin of the aortic arch	0
P7211	Dextraposition of aorta	0
P7212	Double aortic arch	0
P7213	Kommerell's diverticulum	0
P7214	Persistent aortic arch convolutions	0
P7215	Persistent right aortic arch	0
P7216	Vascular ring, aorta	0
P7217	Overriding aorta	0
P721z	Aortic arch anomalies NOS	0
P722.	Atresia and stenosis of aorta	0
P7220	Congenital absence of aorta	0
P7221	Aplasia of aorta	0
P7222	Hypoplasia of aorta	0
P7223	Stricture of aorta	0
P7224	Supra-valvular aortic stenosis	0

P7225	Atresia of aorta	0
P722z	Atresia or stenosis of aorta NOS	0
P72z.	Other anomalies of aorta NOS	0
P72z0	Aneurysm of sinus of Valsalva	0
P72z1	Congenital aneurysm of aorta	0
P72zz	Other anomaly of aorta NOS	0
P73..	Pulmonary artery anomalies	0
P730.	Pulmonary artery anomaly, unspecified	0
P731.	Pulmonary artery agenesis	0
P732.	Pulmonary artery atresia	0
P733.	Coarctation of the pulmonary artery	0
P734.	Hypoplasia of the pulmonary artery	0
P735.	Stenosis of pulmonary artery	0
P736.	Pulmonary arterio-venous aneurysm	0
P737.	Pulmonary artery aneurysm	0
P738.	Atresia of pulmonary artery with septal defect	0
P73y.	Other specified anomaly of pulmonary artery	0
P73z.	Pulmonary artery anomaly NOS	0
P74..	Anomalies of great veins	0
P740.	Anomaly of great veins, unspecified	0
P7400	Anomaly of the pulmonary veins, unspecified	0
P7401	Anomaly of the vena cava, unspecified	0
P740z	Unspecified anomaly of the great veins, NOS	0
P741.	Total anomalous pulmonary venous return - TAPVR	0
P7410	Subdiaphragmatic total anomalous pulmonary venous return	0
P7411	Supradiaphragmatic total anomalous pulmonary venous return	0
P741z	Total anomalous pulmonary venous return NOS	0
P742.	Partial anomalous pulmonary venous return	0
P743.	Anomalous portal vein termination	0
P744.	Portal vein - hepatic artery fistula	0
P74z.	Other great vein anomalies	0
P74z0	Absence of inferior vena cava	0
P74z1	Absence of superior vena cava	0
P74z2	Stenosis of inferior vena cava	0
P74z3	Stenosis of superior vena cava	0
P74z4	Persistent left posterior cardinal vein	0
P74z5	Persistent left superior vena cava	0
P74z6	Scimitar syndrome	0
P74z7	Transposition of pulmonary veins	0
P74z8	Atresia of pulmonary vein	0
P74zz	Other great vein anomaly NOS	0
P76..	Other peripheral vascular system anomalies	0
P760.	Absence of artery NEC	0
P761.	Anomaly of artery NEC	0
P762.	Atresia of artery NEC	0
P763.	Absence of vein NEC	0
P7X..	Congenital malformation of great arteries, unspecified	0
PK33.	Complete situs inversus with dextrocardia	0
PK34.	Situs inversus with levocardia	0
Pyu20	[X]Other congenital malformations of cardiac chambers and connections	0
Pyu21	[X]Other congenital malformations of cardiac septa	0
Pyu22	[X]Other congenital malformations of pulmonary valve	0
Pyu23	[X]Other congenital malformations of tricuspid valve	0
Pyu24	[X]Other congenital malformations of aortic and mitral valves	0
Pyu25	[X]Other specified congenital malformations of the heart	0
Pyu26	[X]Other congenital malformations of aorta	0
Pyu27	[X]Other congenital malformations of pulmonary artery	0
Pyu28	[X]Other congenital malformations of great arteries	0
Pyu29	[X]Other congenital malformations of great veins	0
Pyu2F	[X]Congenital malformations of cardiac chambers and connections	0
Pyu2G	[X]Congenital malformation of tricuspid valve, unspecified	0
Pyu2H	[X]Congenital malformation of aortic and mitral valves unspecified	0
Pyu2J	[X]Congenital malformation of great arteries, unspecified	0
Q48y1	Congenital cardiac failure	0

CONCEPT: COPD (Chronic obstructive pulmonary disease)

READ_CODE	DESCRIPTION_SAIL	SENSITIVE_CODE
14B3.	H/O: chr.obstr. airway disease	0
14OJ.	At risk of chronic obstructive pulmonary disease	0
14OX.	At risk of chronic obstructive pulmonary disease exacerbation	0
1J71.	Suspected chronic obstructive pulmonary disease	0
661M3	Chronic obstructive pulmonary disease self-management plan agreed	0
661N3	Chronic obstructive pulmonary disease self-management plan review	0
66YB.	Chronic obstructive pulmonary disease monitoring	0
66YB0	Chronic obstructive pulmonary disease 3 monthly review	0
66YB1	Chronic obstructive pulmonary disease 6 monthly review	0
66YB2	Telehealth chronic obstructive pulmonary disease monitoring	0
66YD.	Chronic obstructive pulmonary disease monitoring due	0
66YI.	Chronic obstructive pulmonary disease self-management plan given	0
66YL.	Chronic obstructive pulmonary disease follow-up	0
66YM.	Chronic obstructive pulmonary disease annual review	0
66YS.	Chronic obstructive pulmonary disease monitoring by nurse	0
66YT.	Chronic obstructive pulmonary disease monitoring by doctor	0
66Yd.	Chronic obstructive pulmonary disease accident and emergency attendance since last visit	0
66Ye.	Emergency chronic obstructive pulmonary disease admission since last appointment	0
66Yf.	Number of chronic obstructive pulmonary disease exacerbations in past year	0
66Yg.	Chronic obstructive pulmonary disease disturbs sleep	0
66Yh.	Chronic obstructive pulmonary disease does not disturb sleep	0
66Yi.	Multiple chronic obstructive pulmonary disease emergency hospital admissions	0
66Yz2	Shared care chronic obstructive pulmonary disease monitoring	0
679V.	Health education - chronic obstructive pulmonary disease	0
8BMW.	Issue of chronic obstructive pulmonary disease rescue pack	0
8CE6.	Chronic obstructive pulmonary disease leaflet given	0
8CMV.	Has chronic obstructive pulmonary disease care plan	0
8CMW5	Chronic obstructive pulmonary disease care pathway	0
8CR1.	Chronic obstructive pulmonary disease clinical management plan	0
8CeD.	Preferred place of care for next exacerbation of chronic obstructive pulmonary disease	0
8H2R.	Admit COPD emergency	0
8Hkw.	Referral to chronic obstructive pulmonary disease community nursing team	0
8IEZ.	Chronic obstructive pulmonary disease rescue pack declined	0
9N4W.	DNA - Did not attend chronic obstructive pulmonary disease clinic	0
9NgP.	On chronic obstructive pulmonary disease supportive care pathway	0

9Nk70	Seen in chronic obstructive pulmonary disease clinic	0
9Oi..	Chronic obstructive pulmonary disease monitoring administration	0
9Oi0.	Chronic obstructive pulmonary disease monitoring 1st letter	0
9Oi1.	Chronic obstructive pulmonary disease monitoring 2nd letter	0
9Oi2.	Chronic obstructive pulmonary disease monitoring 3rd letter	0
9Oi3.	Chronic obstructive pulmonary disease monitoring verbal invite	0
9Oi4.	Chronic obstructive pulmonary disease monitoring phone invite	0
9e03.	General practitioner out of hours service notified of chronic obstructive pulmonary disease care plan	0
9kf..	Chronic obstructive pulmonary disease - enhanced services administration	0
9kf0.	Chronic obstructive pulmonary disease patient unsuitable for pulmonary rehabilitation - enhanced services administration	0
9kf1.	Referred for chronic obstructive pulmonary disease structured smoking assessment - enhanced services administration	0
9kf2.	Chronic obstructive pulmonary disease structured smoking assessment declined - enhanced services administration	0
H3...	Chronic obstructive pulmonary disease	0
H30..	Bronchitis unspecified	0
H300.	Tracheobronchitis NOS	0
H301.	Laryngotracheobronchitis	0
H302.	Wheezy bronchitis	0
H30z.	Bronchitis NOS	0
H31..	Chronic bronchitis	0
H310.	Simple chronic bronchitis	0
H3100	Chronic catarrhal bronchitis	0
H3101	Smokers' cough	0
H310z	Simple chronic bronchitis NOS	0
H311.	Mucopurulent chronic bronchitis	0
H3110	Purulent chronic bronchitis	0
H3111	Fetid chronic bronchitis	0
H311z	Mucopurulent chronic bronchitis NOS	0
H312.	Obstructive chronic bronchitis	0
H3120	Chronic asthmatic bronchitis	0
H3121	Emphysematous bronchitis	0
H3122	Acute exacerbation of chronic obstructive airways disease	0
H312z	Obstructive chronic bronchitis NOS	0
H313.	Mixed simple and mucopurulent chronic bronchitis	0
H31y.	Other chronic bronchitis	0
H31y0	Chronic tracheitis	0
H31y1	Chronic tracheobronchitis	0
H31yz	Other chronic bronchitis NOS	0
H31z.	Chronic bronchitis NOS	0
H32..	Emphysema	0
H320.	Chronic bullous emphysema	0
H3200	Segmental bullous emphysema	0
H3201	Zonal bullous emphysema	0
H3202	Giant bullous emphysema	0
H3203	Bullous emphysema with collapse	0
H320z	Chronic bullous emphysema NOS	0
H321.	Panlobular emphysema	0
H322.	Centrilobular emphysema	0
H32y.	Other emphysema	0
H32y0	Acute vesicular emphysema	0
H32y1	Atrophic (senile) emphysema	0
H32y2	MacLeod's unilateral emphysema	0
H32yz	Other emphysema NOS	0
H32z.	Emphysema NOS	0
H36..	Mild chronic obstructive pulmonary disease	0
H37..	Moderate chronic obstructive pulmonary disease	0
H38..	Severe chronic obstructive pulmonary disease	0
H39..	Very severe chronic obstructive pulmonary disease	0
H3A..	End stage chronic obstructive airways disease	0
H3B..	Asthma-chronic obstructive pulmonary disease overlap syndrome	0
H3y..	Other specified chronic obstructive airways disease	0
H3y0.	Chronic obstructive pulmonary disease with acute lower respiratory infection	0
H3y1.	Chronic obstructive pulmonary disease with acute exacerbation, unspecified	0
H3z..	Chronic obstructive airways disease NOS	0
H4640	Chronic emphysema due to chemical fumes	0
H4641	Obliterative bronchiolitis due to chemical fumes	0
H581.	Interstitial emphysema	0
H5832	Eosinophilic bronchitis	0
Hyu3.	[X]Chronic lower respiratory diseases	0
Hyu30	[X]Other emphysema	0
Hyu31	[X]Other specified chronic obstructive pulmonary disease	0

CONCEPT: Coronary heart disease

READ_CODE	DESCRIPTION_SAIL	SENSITIVE_CODE
14A..	H/O: cardiovascular disease	0
14A5.	H/O: angina pectoris	0
14AA.	H/O: heart disease NOS	0
14AJ.	H/O: Angina in last year	0
14AL.	H/O: Treatment for ischaemic heart disease	0
14AW.	H/O acute coronary syndrome	0
14Oh0	High risk of cardiovascular disease	0
187..	Frequency of angina	0
1J61.	Suspected ischaemic heart disease	0
33B95	Exercise tolerance test abnormal	0
3889.	Euroscore for angina	0
388E.	Canadian Cardiovascular Society classification of angina	0
44H3.	Cardiac enzymes abnormal	0
44H30	Cardiac enzymes abnormal - first set	0
5533.	Angiocardiography abnormal	0
5543.	Coronary arteriograph.abnormal	0
5C11.	Radionuclide heart study abnormal	0
661M0	Angina self-management plan agreed	0
661N0	Angina self-management plan review	0
662K.	Angina control	0
662K0	Angina control - good	0
662K1	Angina control - poor	0
662K2	Angina control - improving	0
662K3	Angina control - worsening	0
662K4	Angina self management plan commenced	0
662K5	Angina self management plan completed	0
662Kz	Angina control NOS	0
68B2.	Ischaemic heart disease screen	0
6A2..	Coronary heart disease annual review	0

6A4..	Coronary heart disease review	0
8B27.	Antianginal therapy	0
8B3k.	Coronary heart disease medication review	0
8B63.	Salicylate prophylaxis	0
8F9..	Cardiac rehabilitation	0
8F90.	Cardiac rehabilitation - phase 1	0
8F91.	Cardiac rehabilitation - phase 2	0
8F92.	Cardiac rehabilitation - phase 3	0
8F93.	Cardiac rehabilitation - phase 4	0
8F97.	Cardiac rehabilitation programme completed	0
8F98.	Cardiac rehabilitation programme offered	0
8F99.	Cardiac rehabilitation initial assessment offered	0
8H11.	Admit to cardiac ITU	0
8H2Q.	Admit cardiology emergency	0
8H2V.	Admit ischaemic heart disease emergency	0
8H440	Referral to cardiology multidisciplinary team	0
8H7v.	Referral to cardiac rehabilitation nurse	0
8Hkk.	Referral to cardiac rehabilitation programme	0
8Hkl.	Referral to cardiac rehabilitation service by secondary care	0
8Hkt.	Referral to community cardiology service	0
8I3a.	Cardiac rehabilitation declined	0
8IB9.	Referral to cardiac rehabilitation programme not indicated	0
8IE3.	Referral to cardiac rehabilitation programme declined	0
8IEY.	Referral to Angina Plan self-management programme declined	0
8T04.	Referral to Angina Plan self-management programme	0
G....	Circulatory system diseases	0
G3...	Ischaemic heart disease	0
G30..	Acute myocardial infarction	0
G30B.	Acute posterolateral myocardial infarction	0
G31..	Other acute and subacute ischaemic heart disease	0
G310.	Postmyocardial infarction syndrome	0
G311.	Preinfarction syndrome	0
G3110	Myocardial infarction aborted	0
G3111	Unstable angina	0
G3112	Angina at rest	0
G3113	Refractory angina	0
G3114	Worsening angina	0
G3115	Acute coronary syndrome	0
G311z	Preinfarction syndrome NOS	0
G312.	Coronary thrombosis not resulting in myocardial infarction	0
G31y.	Other acute and subacute ischaemic heart disease	0
G31y0	Acute coronary insufficiency	0
G31y1	Microinfarction of heart	0
G31y2	Subendocardial ischaemia	0
G31y3	Transient myocardial ischaemia	0
G31yz	Other acute and subacute ischaemic heart disease NOS	0
G32..	Old myocardial infarction	0
G33..	Angina pectoris	0
G330.	Angina decubitus	0
G3300	Nocturnal angina	0
G330z	Angina decubitus NOS	0
G331.	Prinzmetal's angina	0
G332.	Coronary artery spasm	0
G33z.	Angina pectoris NOS	0
G33z0	Status anginosus	0
G33z1	Stenocardia	0
G33z2	Syncope anginosa	0
G33z3	Angina on effort	0
G33z4	Ischaemic chest pain	0
G33z5	Post infarct angina	0
G33z6	New onset angina	0
G33z7	Stable angina	0
G33zz	Angina pectoris NOS	0
G34..	Other chronic ischaemic heart disease	0
G340.	Coronary atherosclerosis	0
G3400	Single coronary vessel disease	0
G3401	Double coronary vessel disease	0
G341.	Aneurysm of heart	0
G3410	Ventricular cardiac aneurysm	0
G3411	Other cardiac wall aneurysm	0
G3412	Aneurysm of coronary vessels	0
G341z	Aneurysm of heart NOS	0
G342.	Atherosclerotic cardiovascular disease	0
G343.	Ischaemic cardiomyopathy	0
G344.	Silent myocardial ischaemia	0
G34y.	Other specified chronic ischaemic heart disease	0
G34y0	Chronic coronary insufficiency	0
G34y1	Chronic myocardial ischaemia	0
G34yz	Other specified chronic ischaemic heart disease NOS	0
G34z.	Other chronic ischaemic heart disease NOS	0
G34z0	Asymptomatic coronary heart disease	0
G35..	Subsequent myocardial infarction	0
G350.	Subsequent myocardial infarction of anterior wall	0
G351.	Subsequent myocardial infarction of inferior wall	0
G353.	Subsequent myocardial infarction of other sites	0
G35X.	Subsequent myocardial infarction of unspecified site	0
G36..	Certain current complications following acute myocardial infarction	0
G360.	Haemopericardium as current complication following acute myocardial infarction	0
G361.	Atrial septal defect as current complication following acute myocardial infarction	0
G362.	Ventricular septal defect as current complication following acute myocardial infarction	0
G363.	Rupture of cardiac wall without haemopericardium as current complication following acute myocardial infarction	0
G364.	Rupture of chordae tendinae as current complication following acute myocardial infarction	0
G365.	Rupture of papillary muscle as current complication following acute myocardial infarction	0
G366.	Thrombosis of atrium, auricular appendage, and ventricle as current complications following acute myocardial infarction	0
G37..	Cardiac syndrome X	0
G38..	Postoperative myocardial infarction	0
G380.	Postoperative transmural myocardial infarction of anterior wall	0
G381.	Postoperative transmural myocardial infarction of inferior wall	0
G382.	Postoperative transmural myocardial infarction of other sites	0
G383.	Postoperative transmural myocardial infarction of unspecified site	0
G384.	Postoperative subendocardial myocardial infarction	0
G38z.	Postoperative myocardial infarction, unspecified	0
G39..	Coronary microvascular disease	0

G3y..	Other specified ischaemic heart disease	0
G3z..	Ischaemic heart disease NOS	0
G501.	Post infarction pericarditis	0
G5y2.	Cardiovascular arteriosclerosis unspecified	0
G72B1	Coronary artery dissection	0
Gyu3.	[X]Ischaemic heart diseases	0
Gyu30	[X]Other forms of angina pectoris	0
Gyu31	[X]Other current complications following acute myocardial infarction	0
Gyu32	[X]Other forms of acute ischaemic heart disease	0
Gyu33	[X]Other forms of chronic ischaemic heart disease	0
Gyu35	[X]Subsequent myocardial infarction of other sites	0
Gyu36	[X]Subsequent myocardial infarction of unspecified site	0
J421.	Chronic intestinal vascular insufficiency	0
ZV579	[V]Cardiac rehabilitation	0

CONCEPT: Cystic fibrosis

READ_CODE	DESCRIPTION_SAIL	SENSITIVE_CODE
66k..	Cystic fibrosis monitoring	0
66k0.	Cystic fibrosis annual review	0
C10N1	Cystic fibrosis related diabetes mellitus	0
C370.	Cystic fibrosis	0
C3700	Cystic fibrosis with no meconium ileus	0
C3701	Cystic fibrosis with meconium ileus	0
C3702	Cystic fibrosis with pulmonary manifestations	0
C3703	Cystic fibrosis with intestinal manifestations	0
C3704	Arthropathy in cystic fibrosis	0
C3705	Cystic fibrosis with distal intestinal obstruction syndrome	0
C3707	Liver disease due to cystic fibrosis	0
C3708	Cystic fibrosis related cirrhosis	0
C3709	Exacerbation of cystic fibrosis	0
C370y	Cystic fibrosis with other manifestations	0
C370z	Cystic fibrosis NOS	0
Q4352	Perinatal jaundice due to mucoviscidosis	0

CONCEPT: Deafness

READ_CODE	DESCRIPTION_SAIL	SENSITIVE_CODE
13ZL.	Using lip-reading	0
13ZM.	Using British sign language	0
13ZP.	Using Makaton sign language	0
13o5.	Preferred method of communication: Makaton	0
13o6.	Preferred method of communication: British Sign Language	0
13o85	Uses British Sign Language interpreter	0
13o8M	Uses sign language	0
1493.	H/O: hearing problem	0
1C12.	Hearing difficulty	0
1C13.	Deafness	0
1C131	Unilateral deafness	0
1C132	Partial deafness	0
1C133	Bilateral deafness	0
1C16.	Deteriorating hearing	0
1C17.	Hearing aid problem	0
1C18.	Difficulty hearing with background noise	0
1C19.	Difficulty hearing whispers	0
1C1Z.	Hearing symptom NOS	0
2BL2.	O/E - slightly deaf	0
2BL3.	O/E - significantly deaf	0
2BL4.	O/E - very deaf	0
2BL5.	O/E - completely deaf	0
2BM2.	O/E -tune fork=conductive deaf	0
2BM3.	O/E tune fork=perceptive deaf	0
2BM4.	O/E - High tone deafness	0
2DG..	Hearing aid worn	0
2DH0.	Uses hearing loop	0
2DH1.	Does use hearing aid	0
31343	Hearing test abnormal	0
31344	Hearing test bilateral abnormality	0
31345	Hearing test left abnormality	0
31346	Hearing test right abnormality	0
73084	Placement of hearing implant in external ear	0
73085	Attention to hearing implant in external ear	0
73086	Removal of hearing implant from external ear	0
7311A	Insertion of bone anchors for subcutaneous bone anchored hearing aid	0
7317C	Placement of hearing implant in middle ear	0
7317D	Attention to hearing implant in middle ear	0
7317E	Removal of hearing implant from middle ear	0
7319.	Attachment of bone anchored hearing prosthesis	0
73190	Insertion of fixtures for bone anchored hearing prosthesis Stage 1	0
73191	Insertion of fixtures for bone anchored hearing prosthesis Stage 2	0
73192	Reduction of soft tissue for bone anchored hearing prosthesis	0
73193	Attention to fixtures for bone anchored hearing prosthesis	0
73194	One stage insertion of fixtures for bone anchored hearing prosthesis	0
73195	Fitting of external hearing prosthesis to bone anchored fixtures	0
73196	First stage insertion of fixtures for bone anchored hearing prosthesis	0
7319y	Other specified attachment of bone anchored hearing prosthesis	0
7319z	Attachment of bone anchored hearing prosthesis NOS	0
73210	Implantation of intracochlear prosthesis	0
73211	Implantation of extracochlear prosthesis	0
73212	Attention to cochlear prosthesis	0
73218	Removal of cochlear prosthesis	0
8D2..	Auditory aid	0
8D21.	Provide head worn hearing aid	0
8D22.	Provide body worn hearing aid	0
8D23.	Ear fitting hearing aid	0
8D24.	Replace hearing aid battery	0
8D26.	Provision of replacement hearing aid	0
8E31.	Lip reading training	0
8M41.	Hearing aid requested	0
8O0E.	Provision of support as Makaton user	0
8O0F.	Provision of support as signing user	0
9NUw.	Interpreter needed - British Sign Language	0
9NUx.	Interpreter needed - Makaton Sign Language	0

9NfL.	Requires information in Makaton	0
9NnR.	Visual frame sign language interpreter needed	0
9NnS.	Hands-on signing interpreter needed	0
F5801	Presbyacusis	0
F5812	Noise-induced hearing loss	0
F582.	Unspecified sudden hearing loss	0
F59..	Hearing loss	0
F590.	Conductive hearing loss	0
F5900	Unspecified conductive hearing loss	0
F5901	Conductive hearing loss due to disorder of external ear	0
F5902	Conductive hearing loss due to disorder of tympanic membrane	0
F5903	Conductive hearing loss due to disorder of middle ear	0
F5904	Conductive hearing loss due to disorder of inner ear	0
F5905	Conductive hearing loss, bilateral	0
F5906	Conductive hearing loss, unilateral with unrestricted hearing on the contralateral side	0
F590y	Combined conductive hearing loss	0
F590z	Conductive hearing loss NOS	0
F591.	Sensorineural hearing loss	0
F5910	Unspecified perceptive hearing loss	0
F5911	Sensory hearing loss	0
F5912	Neural hearing loss	0
F5913	Central hearing loss	0
F5914	Congenital sensorineural deafness	0
F5915	Ototoxicity - deafness	0
F5916	Sensorineural hearing loss, bilateral	0
F5917	Sensorineural hearing loss, unilateral with unrestricted hearing on the contralateral side	0
F5918	Congenital prelingual deafness	0
F5919	Bilateral profound sensorineural hearing loss	0
F591B	Profound sensorineural hearing loss	0
F591E	Severe sensorineural hearing loss	0
F591y	Combined perceptive hearing loss	0
F591z	Perceptive hearing loss NOS	0
F592.	Mixed conductive and sensorineural deafness	0
F5920	Mixed conductive and sensorineural hearing loss, unilateral with unrestricted hearing on the contralateral side	0
F5921	Mixed conductive and sensorineural hearing loss, bilateral	0
F593.	Deaf mutism, NEC	0
F594.	High frequency deafness	0
F595.	Low frequency deafness	0
F596.	Maternally inherited deafness	0
F597.	Mild acquired hearing loss	0
F598.	Moderate acquired hearing loss	0
F599.	Severe acquired hearing loss	0
F59A.	Profound acquired hearing loss	0
F59y.	Other specified forms of hearing loss	0
F59z.	Deafness NOS	0
F5A..	Hearing impairment	0
FyuU0	[X]Deaf mutism, not elsewhere classified	0
FyuU1	[X]Other specified hearing loss	0
P40..	Ear anomalies with hearing impairment	0
P400.	Ear anomalies with hearing impaired, unspecified	0
P402.	Other external ear anomaly with hearing impairment	0
P402z	Other external ear anomaly with hearing impairment NOS	0
P40z.	Other and unspecified ear anomaly with hearing impaired	0
P40zz	Ear anomaly with hearing impaired NOS	0
ZV412	[V]Problems with hearing	0
ZV45G	[V]Presence of external hearing-aid	0
ZV45N	[V]Bone anchored hearing aid in situ	0
ZV532	[V]Fitting or adjustment of hearing aid	0
ZV53D	[V]Adjustment and management of implanted hearing device	0

CONCEPT: Dementia alzheimer

READ_CODE	DESCRIPTION_SAIL	SENSITIVE_CODE
1461.	H/O: dementia	0
66h..	Dementia monitoring	0
6AB..	Dementia annual review	0
8BM02	Dementia medication review	0
8BPa.	Antipsychotic drug therapy for dementia	0
8CMG2	Review of dementia advance care plan	0
8CMZ.	Dementia care plan	0
8CMZ0	Dementia care plan agreed	0
8CMZ1	Dementia care plan reviewed	0
8CMZ2	Dementia care plan declined	0
8CMZ3	Dementia care plan review declined	0
8CMe0	Dementia advance care plan	0
8CSA.	Dementia advance care plan agreed	0
8IAe0	Dementia advance care plan declined	0
8IAe2	Dementia advance care plan review declined	0
9Ou..	Dementia monitoring administration	0
9Ou1.	Dementia monitoring first letter	0
9Ou2.	Dementia monitoring second letter	0
9Ou3.	Dementia monitoring third letter	0
9Ou4.	Dementia monitoring verbal invite	0
9Ou5.	Dementia monitoring telephone invite	0
9hD..	Exception reporting: dementia quality indicators	0
9hD0.	Excepted from dementia quality indicators: Patient unsuitable	0
9hD1.	Excepted from dementia quality indicators: Informed dissent	0
A411.	Jakob-Creutzfeldt disease	0
A4110	Sporadic Creutzfeldt-Jakob disease	0
E00..	Senile and presenile organic psychotic conditions	0
E000.	Uncomplicated senile dementia	0
E001.	Presenile dementia	0
E0010	Uncomplicated presenile dementia	0
E0011	Presenile dementia with delirium	0
E0012	Presenile dementia with paranoia	0
E0013	Presenile dementia with depression	0
E001z	Presenile dementia NOS	0
E002.	Senile dementia with depressive or paranoid features	0
E0020	Senile dementia with paranoia	0
E0021	Senile dementia with depression	0
E002z	Senile dementia with depressive or paranoid features NOS	0
E003.	Senile dementia with delirium	0
E004.	Arteriosclerotic dementia	0

E0040	Uncomplicated arteriosclerotic dementia	0
E0041	Arteriosclerotic dementia with delirium	0
E0042	Arteriosclerotic dementia with paranoia	0
E0043	Arteriosclerotic dementia with depression	0
E004z	Arteriosclerotic dementia NOS	0
E00z.	Senile or presenile psychoses NOS	0
E012.	Other alcoholic dementia	0
E02y1	Drug-induced dementia	0
E040.	Non-alcoholic amnestic syndrome	0
E041.	Dementia in conditions EC	0
Eu00.	[X]Dementia in Alzheimer's disease	0
Eu000	[X]Dementia in Alzheimer's disease with early onset	0
Eu001	[X]Dementia in Alzheimer's disease with late onset	0
Eu002	[X]Dementia in Alzheimer's dis, atypical or mixed type	0
Eu00z	[X]Dementia in Alzheimer's disease, unspecified	0
Eu01.	[X]Vascular dementia	0
Eu010	[X]Vascular dementia of acute onset	0
Eu011	[X]Multi-infarct dementia	0
Eu012	[X]Subcortical vascular dementia	0
Eu013	[X]Mixed cortical and subcortical vascular dementia	0
Eu01y	[X]Other vascular dementia	0
Eu01z	[X]Vascular dementia, unspecified	0
Eu02.	[X]Dementia in other diseases classified elsewhere	0
Eu020	[X]Dementia in Pick's disease	0
Eu021	[X]Dementia in Creutzfeldt-Jakob disease	0
Eu022	[X]Dementia in Huntington's disease	0
Eu023	[X]Dementia in Parkinson's disease	0
Eu024	[X]Dementia in human immunodef virus [HIV] disease	1
Eu025	[X]Lewy body dementia	0
Eu02y	[X]Dementia in other specified diseases classif elsewhere	0
Eu02z	[X] Unspecified dementia	0
Eu041	[X]Delirium superimposed on dementia	0
Eu107	[X]Mental and behavioural disorders due to use of alcohol: residual and late-onset psychotic disorder	0
F110.	Alzheimer's disease	0
F1100	Alzheimer's disease with early onset	0
F1101	Alzheimer's disease with late onset	0
F111.	Pick's disease	0
F112.	Senile degeneration of brain	0
F1130	Normal pressure hydrocephalus	0
F116.	Lewy body disease	0
F118.	Frontotemporal degeneration	0
F11x7	Cerebral degeneration due to Jakob - Creutzfeldt disease	0
Fyu30	[X]Other Alzheimer's disease	0

CONCEPT: Depression

READ_CODE	DESCRIPTION_SAIL	SENSITIVE_CODE
12K8.	Maternal postnatal depression	0
1465.	H/O: depression	0
1B17.	Depressed	0
1B1U.	Symptoms of depression	0
1BT..	Depressed mood	0
212S.	Depression resolved	0
2257.	O/E - depressed	0
62T1.	Puerperal depression	0
66590	Antidepressant drug treatment started	0
6G00.	Postnatal depression counselling	0
8BK0.	Depression management programme	0
8CAa.	Patient given advice about management of depression	0
8HHq.	Referral for guided self-help for depression	0
8HHq0	Referral for depression self-help video	0
8IH52	Referral for guided self-help for depression declined	0
9H90.	Depression annual review	0
9H91.	Depression medication review	0
9H92.	Depression interim review	0
9HA0.	On depression register	0
9Ov..	Depression monitoring administration	0
9Ov0.	Depression monitoring first letter	0
9Ov1.	Depression monitoring second letter	0
9Ov2.	Depression monitoring third letter	0
9Ov3.	Depression monitoring verbal invite	0
9Ov4.	Depression monitoring telephone invite	0
9k4..	Depression - enhanced services administration	0
9k40.	Depression - enhanced service completed	0
9kQ..	On full dose long term treatment for depression - enhanced services administration	0
E0013	Presenile dementia with depression	0
E002.	Senile dementia with depressive or paranoid features	0
E0021	Senile dementia with depression	0
E002z	Senile dementia with depressive or paranoid features NOS	0
E0043	Arteriosclerotic dementia with depression	0
E02y3	Drug-induced depressive state	0
E11..	Affective psychoses	0
E112.	Single major depressive episode	0
E1120	Single major depressive episode, unspecified	0
E1121	Single major depressive episode, mild	0
E1122	Single major depressive episode, moderate	0
E1123	Single major depressive episode, severe, without mention of psychosis	0
E1124	Single major depressive episode, severe, with psychosis	0
E1125	Single major depressive episode, in partial or unspecified remission	0
E1126	Single major depressive episode, in full remission	0
E112z	Single major depressive episode NOS	0
E113.	Recurrent major depressive episode	0
E1130	Recurrent major depressive episodes, unspecified	0
E1131	Recurrent major depressive episodes, mild	0
E1132	Recurrent major depressive episodes, moderate	0
E1133	Recurrent major depressive episodes, severe, without mention of psychosis	0
E1134	Recurrent major depressive episodes, severe, with psychosis	0
E1135	Recurrent major depressive episodes, in partial or unspecified remission	0
E1136	Recurrent major depressive episodes, in full remission	0
E1137	Recurrent depression	0
E113z	Recurrent major depressive episode NOS	0
E118.	Seasonal affective disorder	0
E11y2	Atypical depressive disorder	0

E11z2	Masked depression	0
E130.	Reactive depressive psychosis	0
E135.	Agitated depression	0
E2003	Anxiety with depression	0
E204.	Neurotic depression reactive type	0
E290.	Brief depressive reaction	0
E290z	Brief depressive reaction NOS	0
E291.	Prolonged depressive reaction	0
E2B..	Depressive disorder NEC	0
E2B0.	Postviral depression	0
E2B1.	Chronic depression	0
Eu204	[X]Post-schizophrenic depression	0
Eu251	[X]Schizoaffective disorder, depressive type	0
Eu32.	[X]Depressive episode	0
Eu320	[X]Mild depressive episode	0
Eu321	[X]Moderate depressive episode	0
Eu322	[X]Severe depressive episode without psychotic symptoms	0
Eu323	[X]Severe depressive episode with psychotic symptoms	0
Eu324	[X]Mild depression	0
Eu325	[X]Major depression, mild	0
Eu326	[X]Major depression, moderately severe	0
Eu327	[X]Major depression, severe without psychotic symptoms	0
Eu328	[X]Major depression, severe with psychotic symptoms	0
Eu329	[X]Single major depressive episode, severe, with psychosis, psychosis in remission	0
Eu32A	[X]Recurrent major depressive episodes, severe, with psychosis, psychosis in remission	0
Eu32B	[X]Antenatal depression	0
Eu32y	[X]Other depressive episodes	0
Eu32z	[X]Depressive episode, unspecified	0
Eu33.	[X]Recurrent depressive disorder	0
Eu330	[X]Recurrent depressive disorder, current episode mild	0
Eu331	[X]Recurrent depressive disorder, current episode moderate	0
Eu332	[X]Recurrent depressive disorder, current episode severe without psychotic symptoms	0
Eu333	[X]Recurrent depressive disorder, current episode severe with psychotic symptoms	0
Eu334	[X]Recurrent depressive disorder, currently in remission	0
Eu33y	[X]Other recurrent depressive disorders	0
Eu33z	[X]Recurrent depressive disorder, unspecified	0
Eu341	[X]Dysthymia	0
Eu3y1	[X]Other recurrent mood affective disorders	0
Eu412	[X]Mixed anxiety and depressive disorder	0
Eu530	[X]Mild mental and behavioural disorders associated with the puerperium, not elsewhere classified	0
Eu920	[X]Depressive conduct disorder	0
R007z	[D]Malaise and fatigue NOS	0

CONCEPT: Diabetes Type 1

READ_CODE	DESCRIPTION_SAIL	SENSITIVE_CODE
66At0	Type I diabetic dietary review	0
C1000	Diabetes mellitus, juvenile type, with no mention of complication	0
C108.	Insulin dependent diabetes mellitus	0
C1080	Insulin-dependent diabetes mellitus with renal complications	0
C1081	Insulin-dependent diabetes mellitus with ophthalmic complications	0
C1082	Insulin-dependent diabetes mellitus with neurological complications	0
C1083	Insulin dependent diabetes mellitus with multiple complications	0
C1084	Unstable insulin dependent diabetes mellitus	0
C1085	Insulin dependent diabetes mellitus with ulcer	0
C1086	Insulin dependent diabetes mellitus with gangrene	0
C1087	Insulin dependent diabetes mellitus with retinopathy	0
C1088	Insulin dependent diabetes mellitus - poor control	0
C1089	Insulin dependent diabetes maturity onset	0
C108A	Insulin-dependent diabetes without complication	0
C108B	Insulin dependent diabetes mellitus with mononeuropathy	0
C108C	Insulin dependent diabetes mellitus with polyneuropathy	0
C108D	Insulin dependent diabetes mellitus with nephropathy	0
C108E	Insulin dependent diabetes mellitus with hypoglycaemic coma	0
C108F	Insulin dependent diabetes mellitus with diabetic cataract	0
C108G	Insulin dependent diabetes mellitus with peripheral angiopathy	0
C108H	Insulin dependent diabetes mellitus with arthropathy	0
C108J	Insulin dependent diabetes mellitus with neuropathic arthropathy	0
C10E.	Type 1 diabetes mellitus	0
C10E0	Type 1 diabetes mellitus with renal complications	0
C10E1	Type 1 diabetes mellitus with ophthalmic complications	0
C10E2	Type 1 diabetes mellitus with neurological complications	0
C10E3	Type 1 diabetes mellitus with multiple complications	0
C10E4	Unstable type 1 diabetes mellitus	0
C10E5	Type 1 diabetes mellitus with ulcer	0
C10E6	Type 1 diabetes mellitus with gangrene	0
C10E7	Type 1 diabetes mellitus with retinopathy	0
C10E8	Type 1 diabetes mellitus - poor control	0
C10E9	Type 1 diabetes mellitus maturity onset	0
C10EA	Type 1 diabetes mellitus without complication	0
C10EB	Type 1 diabetes mellitus with mononeuropathy	0
C10EC	Type 1 diabetes mellitus with polyneuropathy	0
C10ED	Type 1 diabetes mellitus with nephropathy	0
C10EE	Type 1 diabetes mellitus with hypoglycaemic coma	0
C10EF	Type 1 diabetes mellitus with diabetic cataract	0
C10EG	Type 1 diabetes mellitus with peripheral angiopathy	0
C10EH	Type 1 diabetes mellitus with arthropathy	0
C10EJ	Type 1 diabetes mellitus with neuropathic arthropathy	0
C10EK	Type 1 diabetes mellitus with persistent proteinuria	0
C10EL	Type 1 diabetes mellitus with persistent microalbuminuria	0
C10EM	Type 1 diabetes mellitus with ketoacidosis	0
C10EN	Type 1 diabetes mellitus with ketoacidotic coma	0
C10EP	Type 1 diabetes mellitus with exudative maculopathy	0
C10EQ	Type 1 diabetes mellitus with gastroparesis	0
C10P0	Type I diabetes mellitus in remission	0

CONCEPT: Diabetes Type 2

READ_CODE	DESCRIPTION_SAIL	SENSITIVE_CODE
66A3.	Diabetic on diet only	0
66A4.	Diabetic on oral treatment	0
66AH3	Conversion to non-insulin injectable medication	0
66o2.	Diabetic on non-insulin injectable medication	0

66o5.	Diabetic on oral treatment and glucagon-like peptide 1 receptor agonist	0
C1001	Diabetes mellitus, adult onset, with no mention of complication	0
C1074	NIDDM with peripheral circulatory disorder	0
C109.	Non-insulin dependent diabetes mellitus	0
C1090	Non-insulin-dependent diabetes mellitus with renal complications	0
C1091	Non-insulin-dependent diabetes mellitus with ophthalmic complications	0
C1092	Non-insulin-dependent diabetes mellitus with neurological complications	0
C1093	Non-insulin-dependent diabetes mellitus with multiple complications	0
C1094	Non-insulin dependent diabetes mellitus with ulcer	0
C1095	Non-insulin dependent diabetes mellitus with gangrene	0
C1096	Non-insulin-dependent diabetes mellitus with retinopathy	0
C1097	Non-insulin dependent diabetes mellitus - poor control	0
C1099	Non-insulin-dependent diabetes mellitus without complication	0
C109A	Non-insulin dependent diabetes mellitus with mononeuropathy	0
C109B	Non-insulin dependent diabetes mellitus with polyneuropathy	0
C109C	Non-insulin dependent diabetes mellitus with nephropathy	0
C109D	Non-insulin dependent diabetes mellitus with hypoglycaemic coma	0
C109E	Non-insulin dependent diabetes mellitus with diabetic cataract	0
C109F	Non-insulin-dependent diabetes mellitus with peripheral angiopathy	0
C109G	Non-insulin dependent diabetes mellitus with arthropathy	0
C109H	Non-insulin dependent diabetes mellitus with neuropathic arthropathy	0
C109J	Insulin treated Type 2 diabetes mellitus	0
C109K	Hyperosmolar non-ketotic state in type 2 diabetes mellitus	0
C10F.	Type 2 diabetes mellitus	0
C10F0	Type 2 diabetes mellitus with renal complications	0
C10F1	Type 2 diabetes mellitus with ophthalmic complications	0
C10F2	Type 2 diabetes mellitus with neurological complications	0
C10F3	Type 2 diabetes mellitus with multiple complications	0
C10F4	Type 2 diabetes mellitus with ulcer	0
C10F5	Type 2 diabetes mellitus with gangrene	0
C10F6	Type 2 diabetes mellitus with retinopathy	0
C10F7	Type 2 diabetes mellitus - poor control	0
C10F9	Type 2 diabetes mellitus without complication	0
C10FA	Type 2 diabetes mellitus with mononeuropathy	0
C10FB	Type 2 diabetes mellitus with polyneuropathy	0
C10FC	Type 2 diabetes mellitus with nephropathy	0
C10FD	Type 2 diabetes mellitus with hypoglycaemic coma	0
C10FE	Type 2 diabetes mellitus with diabetic cataract	0
C10FF	Type 2 diabetes mellitus with peripheral angiopathy	0
C10FG	Type 2 diabetes mellitus with arthropathy	0
C10FH	Type 2 diabetes mellitus with neuropathic arthropathy	0
C10FJ	Insulin treated Type 2 diabetes mellitus	0
C10FK	Hyperosmolar non-ketotic state in type 2 diabetes mellitus	0
C10FL	Type 2 diabetes mellitus with persistent proteinuria	0
C10FM	Type 2 diabetes mellitus with persistent microalbuminuria	0
C10FN	Type 2 diabetes mellitus with ketoacidosis	0
C10FP	Type 2 diabetes mellitus with ketoacidotic coma	0
C10FQ	Type 2 diabetes mellitus with exudative maculopathy	0
C10FR	Type 2 diabetes mellitus with gastroparesis	0
C10P1	Type II diabetes mellitus in remission	0
L1806	Pre-existing diabetes mellitus, non-insulin-dependent	0

CONCEPT: Diabetic retinopathy

READ_CODE	DESCRIPTION_SAIL	SENSITIVE_CODE
2BBF.	Retinal abnormality - diabetes related	0
2BBL.	O/E - diabetic maculopathy present both eyes	0
2BBP.	O/E - right eye background diabetic retinopathy	0
2BBQ.	O/E - left eye background diabetic retinopathy	0
2BBR.	O/E - right eye preproliferative diabetic retinopathy	0
2BBS.	O/E - left eye preproliferative diabetic retinopathy	0
2BBT.	O/E - right eye proliferative diabetic retinopathy	0
2BBV.	O/E - left eye proliferative diabetic retinopathy	0
2BBW.	O/E - right eye diabetic maculopathy	0
2BBX.	O/E - left eye diabetic maculopathy	0
2BBk.	O/E - right eye stable treated proliferative diabetic retinopathy	0
2BBl.	O/E - left eye stable treated proliferative diabetic retinopathy	0
2BBo.	O/E - sight threatening diabetic retinopathy	0
2BBr.	Impaired vision due to diabetic retinopathy	0
7276.	Pan retinal photocoagulation for diabetes	0
8HBG.	Diabetic retinopathy 12 month review	0
8HBH.	Diabetic retinopathy 6 month review	0
C1087	Insulin dependent diabetes mellitus with retinopathy	0
C1096	Non-insulin-dependent diabetes mellitus with retinopathy	0
C10E7	Type 1 diabetes mellitus with retinopathy	0
C10EP	Type 1 diabetes mellitus with exudative maculopathy	0
C10F6	Type 2 diabetes mellitus with retinopathy	0
C10FQ	Type 2 diabetes mellitus with exudative maculopathy	0
F420.	Diabetic retinopathy	0
F4200	Background diabetic retinopathy	0
F4201	Proliferative diabetic retinopathy	0
F4202	Preproliferative diabetic retinopathy	0
F4203	Advanced diabetic maculopathy	0
F4204	Diabetic maculopathy	0
F4205	Advanced diabetic retinal disease	0
F4206	Non proliferative diabetic retinopathy	0
F4207	High risk proliferative diabetic retinopathy	0
F4208	High risk non proliferative diabetic retinopathy	0
F420z	Diabetic retinopathy NOS	0

CONCEPT: Dialysis

READ_CODE	DESCRIPTION_SAIL	SENSITIVE_CODE
14V2.	H/O: renal dialysis	0
4I29.	Peritoneal dialysis sample	0
4N...	Dialysis fluid examination	0
4N0..	Dialysis fluid urea level	0
4N1..	Dialysis fluid creatinine level	0
4N2..	Dialysis fluid glucose level	0
4N3..	Peritoneal dialysis fluid cell count	0
4N4..	Dialysis fluid potassium level	0
4N5..	Dialysis fluid sodium level	0
7A606	Creation of graft fistula for dialysis	0

7A619	Ligation of arteriovenous dialysis fistula	0
7A61A	Ligation of arteriovenous dialysis graft	0
7L1A.	Compensation for renal failure	0
7L1A0	Renal dialysis	0
7L1A1	Peritoneal dialysis	0
7L1A2	Haemodialysis NEC	0
7L1A3	Haemofiltration	0
7L1A4	Automated peritoneal dialysis	0
7L1A5	Continuous ambulatory peritoneal dialysis	0
7L1A6	Peritoneal dialysis NEC	0
7L1B.	Placement of ambulatory apparatus for compensation for renal failure	0
7L1B0	Insertion of ambulatory peritoneal dialysis catheter	0
7L1B1	Removal of ambulatory peritoneal dialysis catheter	0
7L1B2	Flushing of peritoneal dialysis catheter	0
7L1C0	Insertion of temporary peritoneal dialysis catheter	0
7L1f0	Extracorporeal albumin haemodialysis	0
8882.	Intestinal dialysis	0
8DD..	Dependence on renal dialysis	0
G72C.	Ruptured aneurysm of dialysis vascular access	0
G72D.	Aneurysm of dialysis arteriovenous fistula	0
G72D0	Aneurysm of superficialised artery of dialysis arteriovenous fistula	0
G72D1	Aneurysm of needle site of dialysis arteriovenous fistula	0
G72D2	Aneurysm of anastomotic site of dialysis arteriovenous fistula	0
G72E.	Aneurysm of dialysis vascular access	0
Gy1..	Stenosis of dialysis vascular access	0
Gy10.	Stenosis of dialysis arteriovenous graft	0
Gy11.	Stenosis of dialysis arteriovenous shunt	0
Gy110	Stenosis of arterial side of dialysis arteriovenous shunt	0
Gy111	Stenosis of venous side of dialysis arteriovenous shunt	0
Gy2..	Thrombosis of dialysis vascular access	0
Gy20.	Thrombosis of dialysis arteriovenous graft	0
Gy21.	Thrombosis of dialysis arteriovenous fistula	0
Gy22.	Thrombosis of dialysis arteriovenous shunt	0
Gy3..	Occlusion of dialysis vascular access	0
Gy30.	Occlusion of dialysis arteriovenous graft	0
Gy31.	Occlusion of dialysis arteriovenous fistula	0
Gy32.	Occlusion of dialysis arteriovenous shunt	0
Gy4..	Infection of dialysis vascular access	0
Gy40.	Infection of dialysis arteriovenous graft	0
Gy41.	Infection of dialysis arteriovenous fistula	0
Gy42.	Infection of dialysis arteriovenous shunt	0
Gy5..	Haemorrhage of dialysis vascular access	0
Gy50.	Haemorrhage of dialysis arteriovenous graft	0
Gy51.	Haemorrhage of dialysis arteriovenous fistula	0
Gy52.	Haemorrhage of dialysis arteriovenous shunt	0
Gy6..	Rupture of dialysis vascular access	0
Gy60.	Rupture of dialysis arteriovenous graft	0
Gy61.	Rupture of dialysis arteriovenous fistula	0
Gy62.	Rupture of dialysis arteriovenous shunt	0
SP015	Mechanical complication of dialysis catheter	0
SP056	[X]Infection and inflammatory reaction due to other internal prosthetic devices, implants and grafts	0
SP06B	Continuous ambulatory peritoneal dialysis associated peritonitis	0
SP07G	Stenosis of arteriovenous dialysis fistula	0
SP0E.	Disorders associated with peritoneal dialysis	0
SP0E0	Bloodstained peritoneal dialysis effluent	0
SP0E1	Thrombus in peritoneal dialysis catheter	0
SP0F.	Haemodialysis first use syndrome	0
SP0G.	Anaphylactoid reaction due to haemodialysis	0
SP0H.	Disorder associated with dialysis	0
SP0H0	Dialysis disequilibrium	0
SP3y9	Acute hypercalcaemia of dialysis	0
TA02.	Accidental cut, puncture, perforation or haemorrhage during kidney dialysis or other perfusion	0
TA020	Accidental cut, puncture, perforation or haemorrhage during kidney dialysis	0
TA120	Foreign object left in body during kidney dialysis	0
TA220	Failure of sterile precautions during kidney dialysis	0
TA420	Mechanical failure of instrument or apparatus during kidney dialysis	0
TB11.	Kidney dialysis as the cause of abnormal reaction of patient, or of later complication, without mention of misadventure at the time of procedure	0
U641.	[X]Kidney dialysis as the cause of abnormal reaction of the patient, or of later complication, without mention of misadventure at the time of the procedure	0
ZV451	[V]Renal dialysis status	0
ZV56.	[V]Aftercare involving intermittent dialysis	0
ZV560	[V]Aftercare involving extracorporeal dialysis	0
ZV561	[V]Preparatory care for dialysis	0
ZV56y	[V]Other specified aftercare involving intermittent dialysis	0
ZV56z	[V]Unspecified aftercare involving intermittent dialysis	0
ZVu3G	[X]Other dialysis	0

CONCEPT: Diverticular Disease

READ_CODE	DESCRIPTION_SAIL	SENSITIVE_CODE
76410	Excision of Meckel diverticulum	0
77180	Excision of diverticulum of colon	0
J171.	Gastric diverticulum	0
J23z3	Appendicular diverticulum	0
J51..	Diverticula of intestine	0
J510.	Diverticulosis	0
J5100	Diverticulosis of the duodenum	0
J5101	Diverticulosis of the jejunum	0
J5102	Diverticulosis of the ileum	0
J5103	Diverticulosis of the small intestine unspecified	0
J5104	Diverticulosis of the small intestine NOS	0
J5105	Diverticulosis of the colon	0
J5106	Diverticulosis of the large intestine unspecified	0
J5107	Diverticulosis of the large intestine NOS	0
J5108	Diverticular disease of both small and large intestine without perforation or abscess	0
J5109	Bleeding diverticulosis	0
J510y	Diverticulosis unspecified	0
J510z	Diverticulosis NOS	0
J511.	Diverticulitis	0
J5110	Diverticulitis of the duodenum	0
J5111	Diverticulitis of the jejunum	0
J5112	Diverticulitis of the ileum	0
J5113	Diverticulitis of the small intestine unspecified	0
J5114	Diverticulitis of the small intestine NOS	0

J5115	Diverticulitis of the colon	0
J5116	Diverticulitis of the large intestine unspecified	0
J5117	Diverticulitis of the large intestine NOS	0
J511y	Diverticulitis unspecified	0
J511z	Diverticulitis NOS	0
J512.	Perforated diverticulum	0
J5120	Perforated diverticulum of duodenum	0
J5121	Perforated diverticulum of jejunum	0
J5122	Perforated diverticulum of ileum	0
J5123	Perforated diverticulum of small intestine unspecified	0
J5124	Perforated diverticulum of small intestine NOS	0
J5125	Perforated diverticulum of colon	0
J5126	Perforated diverticulum of large intestine unspecified	0
J5127	Perforated diverticulum of large intestine NOS	0
J5128	Diverticular disease of both small and large intestine with perforation and abscess	0
J512y	Perforated diverticulum unspecified	0
J512z	Perforated diverticulum of intestine NOS	0
J513.	Diverticular abscess	0
J51z.	Diverticula of the intestine NOS	0

CONCEPT: Drug or alcohol misuse

READ_CODE	DESCRIPTION_SAIL	SENSITIVE_CODE
136S.	Hazardous alcohol use	0
136T.	Harmful alcohol use	0
13Y8.	Alcoholics anonymous	0
13ZY.	Disqualified from driving due to excess alcohol	0
13c..	Drug user	0
13c0.	Injecting drug user	0
13c1.	Intravenous drug user	0
13c2.	Never injecting drug user	0
13c3.	Intramuscular drug user	0
13c4.	Intranasal drug user	0
13c5.	Substance misuse increased	0
13c6.	Substance misuse decreased	0
13c7.	Current drug user	0
13c8.	Reduced drugs misuse	0
13c9.	Subcutaneous drug user	0
13cA.	Smokes drugs	0
13cB.	Misuses drugs orally	0
13cC.	Continuous use of drugs	0
13cD.	Episodic use of drugs	0
13cE.	Prolonged high dose use of cannabis	0
13cF.	Preoccupied with substance misuse	0
13cH.	Persistent substance misuse	0
13cJ.	Previously injecting drug user	0
13cK.	Current non recreational drug user	0
13cM.	Substance misuse	0
13cM0	Novel psychoactive substance misuse	0
13cM1	Opioid analgesic dependence	0
13cN.	Has never shared drug injection equipment	0
13cQ.	Behavioural tolerance to drug	0
13cR.	Physical tolerance to drug	0
13cS.	Psychological tolerance to drug	0
13cT.	Reverse tolerance to drug	0
13r0.	Abstinent from drug misuse	0
13r1.	Abstinent from drug misuse in normal environment	0
13r2.	Abstinent from drug misuse in protected environment	0
13r3.	Abstinent from drug misuse on maintenance replacement	0
13r4.	Abstinent from drug misuse when receiving blocking therapy	0
13r5.	Abstinent from substance misuse	0
1462.	H/O: alcoholism	0
1463.	H/O: drug dependency	0
146E.	H/O: recreational drug use	0
146F.	H/O: drug abuse	0
1B1c.	Alcohol induced hallucinations	0
1P30.	Compulsive uncontrollable drug taking	0
1P31.	Compulsive drug taking	0
1P6..	Craving for drugs	0
1P60.	Craves for drugs	0
1P62.	Abnormal craving for drugs	0
1P63.	Excessive craving for drugs	0
1P64.	Irresistible craving for drugs	0
1T...	History of substance misuse	0
1T0..	H/O heroin misuse	0
1T00.	H/O daily heroin misuse	0
1T01.	H/O weekly heroin misuse	0
1T02.	Previous history of heroin misuse	0
1T03.	H/O infrequent heroin misuse	0
1T1..	H/O methadone misuse	0
1T10.	H/O daily methadone misuse	0
1T11.	H/O weekly methadone misuse	0
1T12.	H/O infrequent methadone misuse	0
1T13.	Previous history of methadone misuse	0
1T2..	H/O ecstasy misuse	0
1T20.	H/O daily ecstasy misuse	0
1T21.	H/O weekly ecstasy misuse	0
1T22.	H/O infrequent ecstasy misuse	0
1T23.	Previous history of ecstasy misuse	0
1T3..	H/O benzodiazepine misuse	0
1T30.	H/O daily benzodiazepine misuse	0
1T31.	H/O weekly benzodiazepine misuse	0
1T32.	H/O infrequent benzodiazepine misuse	0
1T33.	Previous history of benzodiazepine misuse	0
1T4..	H/O amphetamine misuse	0
1T40.	H/O daily amphetamine misuse	0
1T41.	H/O weekly amphetamine misuse	0
1T42.	H/O infrequent amphetamine misuse	0
1T43.	Previous history of amphetamine misuse	0
1T5..	H/O cocaine misuse	0
1T50.	H/O daily cocaine misuse	0
1T51.	H/O weekly cocaine misuse	0
1T52.	H/O infrequent cocaine misuse	0

1T53.	Previous history of cocaine misuse	0
1T6..	H/O crack cocaine misuse	0
1T60.	H/O daily crack cocaine misuse	0
1T61.	H/O weekly crack cocaine misuse	0
1T62.	H/O infrequent crack cocaine misuse	0
1T63.	Previous history of crack cocaine misuse	0
1T7..	H/O hallucinogen misuse	0
1T70.	H/O daily hallucinogen misuse	0
1T71.	H/O weekly hallucinogen misuse	0
1T72.	H/O infrequent hallucinogen misuse	0
1T73.	Previous history of hallucinogen misuse	0
1T8..	H/O cannabis misuse	0
1T80.	H/O daily cannabis misuse	0
1T81.	H/O weekly cannabis misuse	0
1T82.	H/O infrequent cannabis misuse	0
1T83.	Previous history of cannabis misuse	0
1T9..	H/O solvent misuse	0
1T90.	H/O daily solvent misuse	0
1T91.	H/O weekly solvent misuse	0
1T92.	H/O infrequent solvent misuse	0
1T93.	Previous history of solvent misuse	0
1TA..	H/O barbiturate misuse	0
1TA0.	H/O daily barbiturate misuse	0
1TA1.	H/O weekly barbiturate misuse	0
1TA2.	H/O infrequent barbiturate misuse	0
1TA3.	Previous history of barbiturate misuse	0
1TB..	H/O major tranquilliser misuse	0
1TB0.	H/O daily major tranquilliser misuse	0
1TB1.	H/O weekly major tranquilliser misuse	0
1TB2.	H/O infrequent major tranquilliser misuse	0
1TB3.	Previous history of major tranquilliser misuse	0
1TC..	H/O anti-depressant misuse	0
1TC0.	H/O daily anti-depressant misuse	0
1TC1.	H/O weekly anti-depressant misuse	0
1TC2.	H/O infrequent anti-depressant misuse	0
1TC3.	Previous history of anti-depressant misuse	0
1TD..	H/O opiate misuse	0
1TD0.	H/O daily opiate misuse	0
1TD1.	H/O weekly opiate misuse	0
1TD2.	H/O infrequent opiate misuse	0
1TD3.	Previous history of opiate misuse	0
1TE..	Uses heroin on top of substitution therapy	0
1TF..	Does not use heroin on top of substitution therapy	0
1TG..	H/O novel psychoactive substance misuse	0
1V...	Drug misuse behaviour	0
1V0..	Misuses drugs	0
1V00.	Occasional drug user	0
1V01.	Long-term drug misuser	0
1V02.	Poly-drug misuser	0
1V03.	Misuses drugs sublingually	0
1V04.	Misuses drugs rectally	0
1V05.	Misuses drugs vaginally	0
1V0E.	Health problem secondary to drug misuse	0
1V2..	Frequency of drug misuse	0
1V22.	Age at starting drug misuse	0
1V23.	Time since stopped drug misuse	0
1V24.	Total time drugs misused	0
1V26.	Misused drugs in past	0
1V64.	Illicit drug use	0
1V65.	Heroin misuse	0
1V66.	Ecstasy misuse	0
2126C	Alcohol dependence resolved	0
66e..	Alcohol disorder monitoring	0
66e0.	Alcohol abuse monitoring	0
67H3.	Lifestyle advice regarding drug misuse	0
68U..	Drugs of abuse screening	0
68U0.	Drugs of abuse urine screening	0
7P221	Delivery of rehabilitation for alcohol addiction	0
8AA..	Drug abuse monitoring	0
8B23.	Drug addiction therapy	0
8B2N.	Drug addiction detoxification therapy - methadone	0
8B2P.	Drug addiction maintenance therapy - methadone	0
8B2Q.	Drug addiction maintenance therapy - buprenorphine	0
8B2R.	Drug addiction detoxification therapy - buprenorphine	0
8B2S.	Opioid agonist substitution therapy	0
8B2T.	Opioid antagonist therapy	0
8BA8.	Alcohol detoxification	0
8BA9.	Detoxification dependence drug	0
8BAW.	Drug dependence self detoxification	0
8BAX.	Drug dependence home detoxification	0
8BAc.	Substance misuse management stopped - self withdrawal	0
8BAd.	Opiate dependence detoxification	0
8BAo.	Benzodiazepine dependence detoxification	0
8BE0.	Reinduction to methadone maintenance therapy	0
8BE1.	Reinduction to buprenorphine maintenance therapy	0
8FB..	Drug rehabilitation	0
8FB0.	Drug detoxification programme completed	0
8G22.	Family intervention for substance misuse	0
8G32.	Aversion therapy - alcoholism	0
8H35.	Admitted to alcohol detoxification centre	0
8H7p.	Referral to community alcohol team	0
8HkG.	Referral to specialist alcohol treatment service	0
8HkJ.	Referral to alcohol brief intervention service	0
8Hq..	Admission to substance misuse detoxification centre	0
8I2N.	Drug dependence home detoxification contraindicated	0
8IAF.	Brief intervention for excessive alcohol consumption declined	0
8IAt.	Extended intervention for excessive alcohol consumption declined	0
8T24.	Referral for brief intervention for substance misuse	0
8T25.	Referral for specialist substance misuse intervention	0
9HC..	Substance misuse monitoring	0
9HC0.	Initial substance misuse assessment	0
9HC1.	Follow up substance misuse assessment	0
9HC2.	Substance misuse clinical management plan agreed	0

9HC3.	Substance misuse clinical management plan reviewed	0
9HC4.	Substance misuse treatment withdrawn	0
9HC5.	Substance misuse treatment programme completed	0
9HC6.	Substance misuse treatment declined	0
9HC7.	Substance misuse treatment not available	0
9HC8.	Declined to give substance misuse history	0
9HCC.	On substance misuse programme	0
9N1yJ	Seen in drug misuse clinic	0
9NN2.	Under care of community alcohol team	0
9NX2.	In-house substance misuse treatment	0
9NdN.	Declined consent for notification of drug misuse	0
9No5.	Seen in substance misuse clinic	0
9k1A.	Brief intervention for excessive alcohol consumption completed	0
9k1B.	Extended intervention for excessive alcohol consumption completed	0
9k50.	Drug misuse - enhanced service completed	0
9k51.	Shared care drug misuse treatment - enhanced services administration	0
9k52.	Drug misuse treatment in primary care - enhanced services administration	0
9k53.	Pharmacy attended for drug misuse - enhanced services administration	0
C1505	Alcohol-induced pseudo-Cushing's syndrome	0
E01..	Alcoholic psychoses	0
E010.	Alcohol withdrawal delirium	0
E011.	Alcohol amnestic syndrome	0
E0110	Korsakov's alcoholic psychosis	0
E0111	Korsakov's alcoholic psychosis with peripheral neuritis	0
E0112	Wernicke-Korsakov syndrome	0
E011z	Alcohol amnestic syndrome NOS	0
E012.	Other alcoholic dementia	0
E0120	Chronic alcoholic brain syndrome	0
E013.	Alcohol withdrawal hallucinosis	0
E014.	Pathological alcohol intoxication	0
E015.	Alcoholic paranoia	0
E01y.	Other alcoholic psychosis	0
E01y0	Alcohol withdrawal syndrome	0
E01yz	Other alcoholic psychosis NOS	0
E01z.	Alcoholic psychosis NOS	0
E02..	Drug psychoses	0
E020.	Drug withdrawal syndrome	0
E021.	Drug-induced paranoia or hallucinatory states	0
E0210	Drug-induced paranoid state	0
E0211	Drug-induced hallucinosis	0
E021z	Drug-induced paranoia or hallucinatory state NOS	0
E022.	Pathological drug intoxication	0
E02y.	Other drug psychoses	0
E02y0	Drug-induced delirium	0
E02y1	Drug-induced dementia	0
E02y2	Drug-induced amnestic syndrome	0
E02y3	Drug-induced depressive state	0
E02y4	Drug-induced personality disorder	0
E02yz	Other drug psychoses NOS	0
E02z.	Drug psychosis NOS	0
E23..	Alcohol dependence syndrome	0
E230.	Acute alcoholic intoxication in alcoholism	0
E2300	Acute alcoholic intoxication, unspecified, in alcoholism	0
E2301	Continuous acute alcoholic intoxication in alcoholism	0
E2302	Episodic acute alcoholic intoxication in alcoholism	0
E2303	Acute alcoholic intoxication in remission, in alcoholism	0
E230z	Acute alcoholic intoxication in alcoholism NOS	0
E231.	Chronic alcoholism	0
E2310	Unspecified chronic alcoholism	0
E2311	Continuous chronic alcoholism	0
E2312	Episodic chronic alcoholism	0
E2313	Chronic alcoholism in remission	0
E231z	Chronic alcoholism NOS	0
E23z.	Alcohol dependence syndrome NOS	0
E24..	Drug dependence	0
E240.	Opioid type drug dependence	0
E2400	Unspecified opioid dependence	0
E2401	Continuous opioid dependence	0
E2402	Episodic opioid dependence	0
E2403	Opioid dependence in remission	0
E240z	Opioid drug dependence NOS	0
E241.	Hypnotic or anxiolytic dependence	0
E2410	Hypnotic or anxiolytic dependence, unspecified	0
E2411	Hypnotic or anxiolytic dependence, continuous	0
E2412	Hypnotic or anxiolytic dependence, episodic	0
E2413	Hypnotic or anxiolytic dependence in remission	0
E241z	Hypnotic or anxiolytic dependence NOS	0
E242.	Cocaine type drug dependence	0
E2420	Cocaine dependence, unspecified	0
E2421	Cocaine dependence, continuous	0
E2422	Cocaine dependence, episodic	0
E2423	Cocaine dependence in remission	0
E242z	Cocaine drug dependence NOS	0
E243.	Cannabis type drug dependence	0
E2430	Cannabis dependence, unspecified	0
E2431	Cannabis dependence, continuous	0
E2432	Cannabis dependence, episodic	0
E2433	Cannabis dependence in remission	0
E243z	Cannabis drug dependence NOS	0
E244.	Amphetamine or other psychostimulant dependence	0
E2440	Amphetamine or psychostimulant dependence, unspecified	0
E2441	Amphetamine or psychostimulant dependence, continuous	0
E2442	Amphetamine or psychostimulant dependence, episodic	0
E2443	Amphetamine or psychostimulant dependence in remission	0
E244z	Amphetamine or psychostimulant dependence NOS	0
E245.	Hallucinogen dependence	0
E2450	Hallucinogen dependence, unspecified	0
E2451	Hallucinogen dependence, continuous	0
E2452	Hallucinogen dependence, episodic	0
E2453	Hallucinogen dependence in remission	0
E245z	Hallucinogen dependence NOS	0
E246.	Glue sniffing dependence	0
E2460	Glue sniffing dependence, unspecified	0

E2461	Glue sniffing dependence, continuous	0
E2462	Glue sniffing dependence, episodic	0
E2463	Glue sniffing dependence in remission	0
E246z	Glue sniffing dependence NOS	0
E247.	Other specified drug dependence	0
E2470	Other specified drug dependence, unspecified	0
E2471	Other specified drug dependence, continuous	0
E2472	Other specified drug dependence, episodic	0
E2473	Other specified drug dependence in remission	0
E247z	Other specified drug dependence NOS	0
E248.	Combined opioid with other drug dependence	0
E2480	Combined opioid with other drug dependence, unspecified	0
E2481	Combined opioid with other drug dependence, continuous	0
E2482	Combined opioid with other drug dependence, episodic	0
E2483	Combined opioid with other drug dependence in remission	0
E248z	Combined opioid with other drug dependence NOS	0
E249.	Combined drug dependence, excluding opioids	0
E2490	Combined drug dependence, excluding opioid, unspecified	0
E2491	Combined drug dependence, excluding opioid, continuous	0
E2492	Combined drug dependence, excluding opioid, episodic	0
E2493	Combined drug dependence, excluding opioid, in remission	0
E249z	Combined drug dependence, excluding opioid, NOS	0
E24A.	Ecstasy type drug dependence	0
E24z.	Drug dependence NOS	0
E25..	Nondependent abuse of drugs	0
E250.	Nondependent alcohol abuse	0
E2500	Nondependent alcohol abuse, unspecified	0
E2501	Nondependent alcohol abuse, continuous	0
E2502	Nondependent alcohol abuse, episodic	0
E2503	Nondependent alcohol abuse in remission	0
E250z	Nondependent alcohol abuse NOS	0
E252.	Nondependent cannabis abuse	0
E2520	Nondependent cannabis abuse, unspecified	0
E2521	Nondependent cannabis abuse, continuous	0
E2522	Nondependent cannabis abuse, episodic	0
E2523	Nondependent cannabis abuse in remission	0
E252z	Nondependent cannabis abuse NOS	0
E253.	Nondependent hallucinogen abuse	0
E2530	Nondependent hallucinogen abuse, unspecified	0
E2531	Nondependent hallucinogen abuse, continuous	0
E2532	Nondependent hallucinogen abuse, episodic	0
E2533	Nondependent hallucinogen abuse in remission	0
E253z	Nondependent hallucinogen abuse NOS	0
E254.	Nondependent hypnotic or anxiolytic abuse	0
E2540	Nondependent hypnotic or anxiolytic abuse, unspecified	0
E2541	Nondependent hypnotic or anxiolytic abuse, continuous	0
E2542	Nondependent hypnotic or anxiolytic abuse, episodic	0
E2543	Nondependent hypnotic or anxiolytic abuse in remission	0
E254z	Nondependent hypnotic or anxiolytic abuse NOS	0
E255.	Nondependent opioid abuse	0
E2550	Nondependent opioid abuse, unspecified	0
E2551	Nondependent opioid abuse, continuous	0
E2552	Nondependent opioid abuse, episodic	0
E2553	Nondependent opioid abuse in remission	0
E255z	Nondependent opioid abuse NOS	0
E256.	Nondependent cocaine abuse	0
E2560	Nondependent cocaine abuse, unspecified	0
E2561	Nondependent cocaine abuse, continuous	0
E2562	Nondependent cocaine abuse, episodic	0
E2563	Nondependent cocaine abuse in remission	0
E256z	Nondependent cocaine abuse NOS	0
E257.	Nondependent amphetamine or other psychostimulant abuse	0
E2570	Nondependent amphetamine or psychostimulant abuse, unspecified	0
E2571	Nondependent amphetamine or psychostimulant abuse, continuous	0
E2572	Nondependent amphetamine or psychostimulant abuse, episodic	0
E2573	Nondependent amphetamine or psychostimulant abuse in remission	0
E257z	Nondependent amphetamine or psychostimulant abuse NOS	0
E258.	Nondependent antidepressant type drug abuse	0
E2580	Nondependent antidepressant type drug abuse, unspecified	0
E2581	Nondependent antidepressant type drug abuse, continuous	0
E2582	Nondependent antidepressant type drug abuse, episodic	0
E2583	Nondependent antidepressant type drug abuse in remission	0
E258z	Nondependent antidepressant type drug abuse NOS	0
E259.	Nondependent mixed drug abuse	0
E2590	Nondependent mixed drug abuse, unspecified	0
E2591	Nondependent mixed drug abuse, continuous	0
E2592	Nondependent mixed drug abuse, episodic	0
E2593	Nondependent mixed drug abuse in remission	0
E2594	Misuse of prescription only drugs	0
E259z	Nondependent mixed drug abuse NOS	0
E25y.	Nondependent other drug abuse	0
E25y0	Nondependent other drug abuse, unspecified	0
E25y1	Nondependent other drug abuse, continuous	0
E25y2	Nondependent other drug abuse, episodic	0
E25y3	Nondependent other drug abuse in remission	0
E25yz	Nondependent other drug abuse NOS	0
E25z.	Misuse of drugs NOS	0
Eu04.	[X]Delirium, not induced by alcohol and other psychoactive subs	0
Eu1..	[X]Mental and behavioural disorders due to psychoactive substance use	0
Eu10.	[X]Mental and behavioural disorders due to use of alcohol	0
Eu100	[X]Mental and behavioural disorders due to use of alcohol: acute intoxication	0
Eu101	[X]Mental and behavioural disorders due to use of alcohol: harmful use	0
Eu102	[X]Mental and behavioural disorders due to use of alcohol: dependence syndrome	0
Eu103	[X]Mental and behavioural disorders due to use of alcohol: withdrawal state	0
Eu104	[X]Mental and behavioural disorders due to use of alcohol: withdrawal state with delirium	0
Eu105	[X]Mental and behavioural disorders due to use of alcohol: psychotic disorder	0
Eu106	[X]Mental and behavioural disorders due to use of alcohol: amnesic syndrome	0
Eu107	[X]Mental and behavioural disorders due to use of alcohol: residual and late-onset psychotic disorder	0
Eu108	[X]Alcohol withdrawal-induced seizure	0
Eu10y	[X]Mental and behavioural disorders due to use of alcohol: other mental and behavioural disorders	0
Eu10z	[X]Mental and behavioural disorders due to use of alcohol: unspecified mental and behavioural disorder	0
Eu11.	[X]Mental and behavioural disorders due to use of opioids	0
Eu110	[X]Mental and behavioural disorders due to use of opioids: acute intoxication	0

Eu111	[X]Mental and behavioural disorders due to use of opioids: harmful use	0
Eu112	[X]Mental and behavioural disorders due to use of opioids: dependence syndrome	0
Eu113	[X]Mental and behavioural disorders due to use of opioids: withdrawal state	0
Eu114	[X]Mental and behavioural disorders due to use of opioids: withdrawal state with delirium	0
Eu115	[X]Mental and behavioural disorders due to use of opioids: psychotic disorder	0
Eu116	[X]Mental and behavioural disorders due to use of opioids: amnesic syndrome	0
Eu117	[X]Mental and behavioural disorders due to use of opioids: residual and late-onset psychotic disorder	0
Eu11y	[X]Mental and behavioural disorders due to use of opioids: other mental and behavioural disorders	0
Eu11z	[X]Mental and behavioural disorders due to use of opioids: unspecified mental and behavioural disorder	0
Eu12.	[X]Mental and behavioural disorders due to use of cannabinoids	0
Eu120	[X]Mental and behavioural disorders due to use of cannabinoids: acute intoxication	0
Eu121	[X]Mental and behavioural disorders due to use of cannabinoids: harmful use	0
Eu122	[X]Mental and behavioural disorders due to use of cannabinoids: dependence syndrome	0
Eu123	[X]Mental and behavioural disorders due to use of cannabinoids: withdrawal state	0
Eu124	[X]Mental and behavioural disorders due to use of cannabinoids: withdrawal state with delirium	0
Eu125	[X]Mental and behavioural disorders due to use of cannabinoids: psychotic disorder	0
Eu126	[X]Mental and behavioural disorders due to use of cannabinoids: amnesic syndrome	0
Eu127	[X]Mental and behavioural disorders due to use of cannabinoids: residual and late-onset psychotic disorder	0
Eu12y	[X]Mental and behavioural disorders due to use of cannabinoids: other mental and behavioural disorders	0
Eu12z	[X]Mental and behavioural disorders due to use of cannabinoids: unspecified mental and behavioural disorder	0
Eu13.	[X]Mental and behavioural disorders due to use of sedatives or hypnotics	0
Eu130	[X]Mental and behavioural disorders due to use of sedatives or hypnotics: acute intoxication	0
Eu131	[X]Mental and behavioural disorders due to use of sedatives or hypnotics: harmful use	0
Eu132	[X]Mental and behavioural disorders due to use of sedatives or hypnotics: dependence syndrome	0
Eu133	[X]Mental and behavioural disorders due to use of sedatives or hypnotics: withdrawal state	0
Eu134	[X]Mental and behavioural disorders due to use of sedatives or hypnotics: withdrawal state with delirium	0
Eu135	[X]Mental and behavioural disorders due to use of sedatives or hypnotics: psychotic disorder	0
Eu136	[X]Mental and behavioural disorders due to use of sedatives or hypnotics: amnesic syndrome	0
Eu137	[X]Mental and behavioural disorders due to use of sedatives or hypnotics: residual and late-onset psychotic disorder	0
Eu13y	[X]Mental and behavioural disorders due to use of sedatives or hypnotics: other mental and behavioural disorders	0
Eu13z	[X]Mental and behavioural disorders due to use of sedatives or hypnotics: unspecified mental and behavioural disorder	0
Eu14.	[X]Mental and behavioural disorders due to use of cocaine	0
Eu140	[X]Mental and behavioural disorders due to use of cocaine: acute intoxication	0
Eu141	[X]Mental and behavioural disorders due to use of cocaine: harmful use	0
Eu142	[X]Mental and behavioural disorders due to use of cocaine: dependence syndrome	0
Eu143	[X]Mental and behavioural disorders due to use of cocaine: withdrawal state	0
Eu144	[X]Mental and behavioural disorders due to use of cocaine: withdrawal state with delirium	0
Eu145	[X]Mental and behavioural disorders due to use of cocaine: psychotic disorder	0
Eu146	[X]Mental and behavioural disorders due to use of cocaine: amnesic syndrome	0
Eu147	[X]Mental and behavioural disorders due to use of cocaine: residual and late-onset psychotic disorder	0
Eu14y	[X]Mental and behavioural disorders due to use of cocaine: other mental and behavioural disorders	0
Eu14z	[X]Mental and behavioural disorders due to use of cocaine: unspecified mental and behavioural disorder	0
Eu16.	[X]Mental and behavioural disorders due to use of hallucinogens	0
Eu160	[X]Mental and behavioural disorders due to use of hallucinogens: acute intoxication	0
Eu161	[X]Mental and behavioural disorders due to use of hallucinogens: harmful use	0
Eu162	[X]Mental and behavioural disorders due to use of hallucinogens: dependence syndrome	0
Eu163	[X]Mental and behavioural disorders due to use of hallucinogens: withdrawal state	0
Eu164	[X]Mental and behavioural disorders due to use of hallucinogens: withdrawal state with delirium	0
Eu165	[X]Mental and behavioural disorders due to use of hallucinogens: psychotic disorder	0
Eu166	[X]Mental and behavioural disorders due to use of hallucinogens: amnesic syndrome	0
Eu167	[X]Mental and behavioural disorders due to use of hallucinogens: residual and late-onset psychotic disorder	0
Eu16y	[X]Mental and behavioural disorders due to use of hallucinogens: other mental and behavioural disorders	0
Eu16z	[X]Mental and behavioural disorders due to use of hallucinogens: unspecified mental and behavioural disorder	0
Eu18.	[X]Mental and behavioural disorders due to use of volatile solvents	0
Eu180	[X]Mental and behavioural disorders due to use of volatile solvents: acute intoxication	0
Eu181	[X]Mental and behavioural disorders due to use of volatile solvents: harmful use	0
Eu182	[X]Mental and behavioural disorders due to use of volatile solvents: dependence syndrome	0
Eu183	[X]Mental and behavioural disorders due to use of volatile solvents: withdrawal state	0
Eu184	[X]Mental and behavioural disorders due to use of volatile solvents: withdrawal state with delirium	0
Eu185	[X]Mental and behavioural disorders due to use of volatile solvents: psychotic disorder	0
Eu186	[X]Mental and behavioural disorders due to use of volatile solvents: amnesic syndrome	0
Eu187	[X]Mental and behavioural disorders due to use of volatile solvents: residual and late-onset psychotic disorder	0
Eu18y	[X]Mental and behavioural disorders due to use of volatile solvents: other mental and behavioural disorders	0
Eu18z	[X]Mental and behavioural disorders due to use of volatile solvents: unspecified mental and behavioural disorder	0
Eu19.	[X]Mental and behavioural disorders due to multiple drug use and use of other psychoactive substances	0
Eu190	[X]Mental and behavioural disorders due to multiple drug use and use of other psychoactive substances: acute intoxication	0
Eu191	[X]Mental and behavioural disorders due to multiple drug use and use of other psychoactive substances: harmful use	0
Eu192	[X]Mental and behavioural disorders due to multiple drug use and use of other psychoactive substances: dependence syndrome	0
Eu193	[X]Mental and behavioural disorders due to multiple drug use and use of other psychoactive substances: withdrawal state	0
Eu194	[X]Mental and behavioural disorders due to multiple drug use and use of other psychoactive substances: withdrawal state with delirium	0
Eu195	[X]Mental and behavioural disorders due to multiple drug use and use of other psychoactive substances: psychotic disorder	0
Eu196	[X]Mental and behavioural disorders due to multiple drug use and use of other psychoactive substances: amnesic syndrome	0
Eu197	[X]Mental and behavioural disorders due to multiple drug use and use of other psychoactive substances: residual and late-onset psychotic	0
Eu19y	[X]Mental and behavioural disorders due to multiple drug use and use of other psychoactive substances: other mental and behavioural disorders	0
Eu19z	[X]Mental and behavioural disorders due to multiple drug use and use of other psychoactive substances: unspecified mental and behavioural disorder	0
Eu1A.	[X]Mental and behavioural disorders due to use of crack cocaine	0
Eu1A0	[X]Mental and behavioural disorders due to use of crack cocaine: acute intoxication	0
Eu1A1	[X]Mental and behavioural disorders due to use of crack cocaine: harmful use	0
Eu1A2	[X]Mental and behavioural disorders due to use of crack cocaine: dependence syndrome	0
Eu1A3	[X]Mental and behavioural disorders due to use of crack cocaine: withdrawal state	0
Eu1A4	[X]Mental and behavioural disorders due to use of crack cocaine: withdrawal state with delirium	0
Eu1A5	[X]Mental and behavioural disorders due to use of crack cocaine: psychotic disorder	0
Eu1A6	[X]Mental and behavioural disorders due to use of crack cocaine: amnesic syndrome	0
Eu1A7	[X]Mental and behavioural disorders due to use of crack cocaine: residual and late-onset psychotic disorder	0
Eu1Ay	[X]Mental and behavioural disorders due to use of crack cocaine: other mental and behavioural disorders	0
Eu1Az	[X]Mental and behavioural disorders due to use of crack cocaine: unspecified mental and behavioural disorder	0
Eu55.	[X]Abuse of non-dependence-producing substances	0
F11x0	Cerebral degeneration due to alcoholism	0
F1440	Cerebellar ataxia due to alcoholism	0
F25B.	Alcohol-induced epilepsy	0
F375.	Alcoholic polyneuropathy	0
F3941	Alcoholic myopathy	0
G555.	Alcoholic cardiomyopathy	0
G801E	Deep vein thrombosis of leg related to intravenous drug use	0
G8523	Oesophageal varices in alcoholic cirrhosis of the liver	0
J153.	Alcoholic gastritis	0
J610.	Alcoholic fatty liver	0
J611.	Acute alcoholic hepatitis	0
J612.	Alcoholic cirrhosis of liver	0
J6120	Alcoholic fibrosis and sclerosis of liver	0
J613.	Alcoholic liver damage unspecified	0
J6130	Alcoholic hepatic failure	0
J615.	Cirrhosis - non alcoholic	0

J617.	Alcoholic hepatitis	0
J6170	Chronic alcoholic hepatitis	0
J6708	Alcohol-induced acute pancreatitis	0
J6710	Alcohol-induced chronic pancreatitis	0
L183.	Drug dependence during pregnancy, childbirth and the puerperium	0
L1830	Drug dependence - unspecified whether during pregnancy or the puerperium	0
L1831	Drug dependence during pregnancy - baby delivered	0
L1832	Drug dependence in the puerperium - baby delivered	0
L1833	Drug dependence during pregnancy - baby not yet delivered	0
L1834	Drug dependence in the puerperium - baby delivered during previous episode of care	0
Q4850	Neonatal withdrawal symptoms from maternal use of drugs of addiction	0
U6097	[X]Psychostimulants with abuse potential causing adverse effects in therapeutic use	0
ZV113	[V]Personal history of alcoholism	0
ZV114	[V]Personal history of psychoactive substance abuse	0
ZV115	[V]Personal history of drug abuse by injection	0
ZV4K1	[V]Drug use	0
ZVu54	[X]Other problems related to care provider dependency	0

CONCEPT: Eating disorders

READ_CODE	DESCRIPTION_SAIL	SENSITIVE_CODE
1467.	H/O: anorexia nervosa	0
1JZ..	Suspected binge eating disorder	0
8HTN.	Referral to eating disorders clinic	0
9Nk9.	Seen in eating disorder clinic	0
E2642	Cyclical vomiting - psychogenic	0
E271.	Anorexia nervosa	0
E275.	Other and unspecified non-organic eating disorders	0
E2750	Unspecified non-organic eating disorder	0
E2751	Bulimia (non-organic overeating)	0
E2752	Pica	0
E2754	Psychogenic vomiting NOS	0
E275y	Other specified non-organic eating disorder	0
E275z	Non-organic eating disorder NOS	0
Eu50.	[X]Eating disorders	0
Eu500	[X]Anorexia nervosa	0
Eu501	[X]Atypical anorexia nervosa	0
Eu502	[X]Bulimia nervosa	0
Eu503	[X]Atypical bulimia nervosa	0
Eu504	[X]Overeating associated with other psychological disturbances	0
Eu505	[X]Vomiting associated with other psychological disturbances	0
Eu50y	[X]Other eating disorders	0
Eu50z	[X]Eating disorder, unspecified	0
Fy05.	Nocturnal sleep-related eating disorder	0
R0360	[D]Excessive eating	0

CONCEPT: Ehlers Danlos Syndrome

READ_CODE	DESCRIPTION_SAIL	SENSITIVE_CODE
N235.	Hypermobility syndrome	0
PGy22	Ehlers-Danlos syndrome type III	0

CONCEPT: Endometriosis

READ_CODE	DESCRIPTION_SAIL	SENSITIVE_CODE
7E0D8	Laparoscopic laser destruction of endometriosis	0
BBL1.	[M]Endolymphatic stromal myosis	0
K50..	Endometriosis	0
K500.	Endometriosis of uterus	0
K5000	Internal endometriosis	0
K5001	Endometriosis of myometrium	0
K5002	Endometriosis of cervix	0
K500z	Endometriosis of uterus NOS	0
K501.	Endometriosis of ovary	0
K502.	Endometriosis of the fallopian tube	0
K503.	Endometriosis of the pelvic peritoneum	0
K5030	Endometriosis of the broad ligament	0
K5031	Endometriosis of the pouch of Douglas	0
K5032	Endometriosis of the parametrium	0
K5033	Endometriosis of the round ligament	0
K503z	Endometriosis of the pelvic peritoneum NOS	0
K504.	Endometriosis of the rectovaginal septum and vagina	0
K5040	Endometriosis of the rectovaginal septum	0
K5041	Endometriosis of the vagina	0
K504z	Endometriosis of the rectovaginal septum and vagina NOS	0
K505.	Endometriosis of the intestine	0
K5050	Endometriosis of the appendix	0
K5051	Endometriosis of the colon	0
K5052	Endometriosis of the rectum	0
K505z	Endometriosis of the intestine NOS	0
K506.	Endometriosis in scar of skin	0
K50y.	Other endometriosis	0
K50y0	Endometriosis of the bladder	0
K50y1	Endometriosis of the lung	0
K50y2	Endometriosis of the umbilicus	0
K50y3	Endometriosis of the vulva	0
K50yz	Other endometriosis NOS	0
K50z.	Endometriosis NOS	0
Kyu90	[X]Other endometriosis	0

CONCEPT: Epilepsy

READ_CODE	DESCRIPTION_SAIL	SENSITIVE_CODE
1473.	H/O: epilepsy	0
14On.	At risk of sudden unexpected death in epilepsy	0
1B1W.	Transient epileptic amnesia	0
1O30.	Epilepsy confirmed	0
2828.	Absence seizure	0
667..	Epilepsy monitoring	0
6671.	Initial epilepsy assessment	0
6672.	Follow-up epilepsy assessment	0
6673.	Driving licence	0
6674.	Epilepsy associated problems	0
6675.	Fit frequency	0

6676.	Last fit	0
6677.	Epilepsy drug side effects	0
6678.	Epilepsy treatment changed	0
6679.	Epilepsy treatment started	0
667A.	Epilepsy treatment stopped	0
667B.	Nocturnal epilepsy	0
667C.	Epilepsy control good	0
667D.	Epilepsy control poor	0
667E.	Epilepsy care arrangement	0
667G.	Epilepsy restricts employment	0
667H.	Epilepsy prevents employment	0
667J.	Epilepsy impairs education	0
667K.	Epilepsy limits activities	0
667L.	Epilepsy does not limit activities	0
667M.	Epilepsy management plan given	0
667N.	Epilepsy severity	0
667Q.	1 to 12 seizures a year	0
667R.	2 to 4 seizures a month	0
667S.	1 to 7 seizures a week	0
667T.	Daily seizures	0
667V.	Many seizures a day	0
667W.	Emergency epilepsy treatment since last appointment	0
667X.	No epilepsy drug side effects	0
667Z.	Epilepsy monitoring NOS	0
8B66.	Anticonvulsant therapy	0
8BIF.	Epilepsy medication review	0
Eu052	[X]Organic delusional [schizophrenia-like] disorder	0
Eu05y	[X]Other specified mental disorder due to brain damage and dysfunction and to physical disease	0
Eu060	[X]Organic personality disorder	0
Eu803	[X]Acquired aphasia with epilepsy [Landau - Kleffner]	0
F1321	Progressive myoclonic epilepsy	0
F25..	Epilepsy	0
F250.	Generalised nonconvulsive epilepsy	0
F2500	Petit mal (minor) epilepsy	0
F2501	Pykno-epilepsy	0
F2502	Epileptic seizures - atonic	0
F2503	Epileptic seizures - akinetic	0
F2504	Juvenile absence epilepsy	0
F2505	Lennox-Gastaut syndrome	0
F250y	Other specified generalised nonconvulsive epilepsy	0
F250z	Generalised nonconvulsive epilepsy NOS	0
F251.	Generalised convulsive epilepsy	0
F2510	Grand mal (major) epilepsy	0
F2511	Neonatal myoclonic epilepsy	0
F2512	Epileptic seizures - clonic	0
F2513	Epileptic seizures - myoclonic	0
F2514	Epileptic seizures - tonic	0
F2515	Tonic-clonic epilepsy	0
F2516	Grand mal seizure	0
F251y	Other specified generalised convulsive epilepsy	0
F251z	Generalised convulsive epilepsy NOS	0
F252.	Petit mal status	0
F253.	Grand mal status	0
F254.	Partial epilepsy with impairment of consciousness	0
F2540	Temporal lobe epilepsy	0
F2541	Psychomotor epilepsy	0
F2542	Psychosensory epilepsy	0
F2543	Limbic system epilepsy	0
F2544	Epileptic automatism	0
F2545	Complex partial epileptic seizure	0
F254z	Partial epilepsy with impairment of consciousness NOS	0
F255.	Partial epilepsy without mention of impairment of consciousness	0
F2550	Jacksonian, focal or motor epilepsy	0
F2551	Sensory induced epilepsy	0
F2552	Somatosensory epilepsy	0
F2553	Visceral reflex epilepsy	0
F2554	Visual reflex epilepsy	0
F2555	Unilateral epilepsy	0
F2556	Simple partial epileptic seizure	0
F255y	Other specified partial epilepsy without mention of impairment of consciousness	0
F255z	Partial epilepsy without mention of impairment of consciousness NOS	0
F256.	Infantile spasms	0
F2560	Hypsarrhythmia	0
F2561	Salaam attacks	0
F256z	Infantile spasms NOS	0
F257.	Kojevnikov's epilepsy	0
F258.	Post-ictal state	0
F259.	Early infantile epileptic encephalopathy with suppression bursts	0
F25A.	Juvenile myoclonic epilepsy	0
F25B.	Alcohol-induced epilepsy	0
F25C.	Drug-induced epilepsy	0
F25D.	Menstrual epilepsy	0
F25E.	Stress-induced epilepsy	0
F25F.	Photosensitive epilepsy	0
F25G.	Severe myoclonic epilepsy in infancy	0
F25X.	Status epilepticus, unspecified	0
F25y.	Other forms of epilepsy	0
F25y0	Cursive (running) epilepsy	0
F25y1	Gelastic epilepsy	0
F25y2	Localization-related(focal)(partial)idiopathic epilepsy and epileptic syndromes with seizures of localised onset	0
F25y3	Complex partial status epilepticus	0
F25y4	Benign Rolandic epilepsy	0
F25y5	Panayiotopoulos syndrome	0
F25yz	Other forms of epilepsy NOS	0
F25z.	Epilepsy NOS	0
Fyu50	[X]Other generalized epilepsy and epileptic syndromes	0
Fyu51	[X]Other epilepsy	0
Fyu52	[X]Other status epilepticus	0
Fyu59	[X]Status epilepticus, unspecified	0
SC200	Traumatic epilepsy	0

CONCEPT: Fibromyalgia

READ_CODE	DESCRIPTION_SAIL	SENSITIVE_CODE
N239.	Fibromyalgia	0
N248.	Fibromyalgia	0

CONCEPT: Glaucoma

READ_CODE	DESCRIPTION_SAIL	SENSITIVE_CODE
1482.	H/O: glaucoma	0
66T1.	Glaucoma monitoring	0
7259.	Operations following glaucoma surgery	0
72590	Needling of bleb following glaucoma surgery	0
72591	Injection of bleb following glaucoma surgery	0
72592	Revision of bleb NEC following glaucoma surgery	0
72593	Removal of releasable suture following glaucoma surgery	0
72594	Laser suture lysis following glaucoma surgery	0
7259y	Other specified operations following glaucoma surgery	0
7259z	Operations following glaucoma surgery NOS	0
7275.	Pan retinal photocoagulation for glaucoma	0
F4042	Blind hypertensive eye	0
F4421	Glaucomatocyclitic crises	0
F45..	Glaucoma	0
F450.	Borderline glaucoma	0
F4500	Unspecified preglaucoma	0
F4501	Open angle glaucoma with borderline intraocular pressure	0
F4502	Borderline glaucoma with anatomical narrow angle	0
F4503	Borderline glaucoma steroid responder	0
F450z	Borderline glaucoma NOS	0
F451.	Open-angle glaucoma	0
F4510	Unspecified open-angle glaucoma	0
F4511	Primary open-angle glaucoma	0
F4512	Low tension glaucoma	0
F4513	Pigmentary glaucoma	0
F4514	Glaucoma of childhood	0
F4515	Open-angle glaucoma residual stage	0
F451z	Open-angle glaucoma NOS	0
F452.	Primary angle-closure glaucoma	0
F4520	Unspecified primary angle-closure glaucoma	0
F4521	Intermittent primary angle-closure glaucoma	0
F4522	Acute primary angle-closure glaucoma	0
F4523	Chronic primary angle-closure glaucoma	0
F4524	Primary angle-closure glaucoma residual stage	0
F452z	Primary angle-closure glaucoma NOS	0
F453.	Steroid-induced glaucoma	0
F4530	Steroid-induced glaucoma glaucomatous stage	0
F4531	Steroid-induced glaucoma residual stage	0
F453z	Steroid-induced glaucoma NOS	0
F454.	Glaucoma due to disease EC	0
F4540	Glaucoma due to chamber angle anomaly	0
F4541	Glaucoma due to iris anomaly	0
F4542	Glaucoma due to other anterior segment anomaly	0
F4543	Glaucoma due to systemic syndrome	0
F4544	Glaucoma in endocrine, nutritional and metabolic diseases	0
F454z	Glaucoma due to disease NOS	0
F455.	Glaucoma associated with disorders of the lens	0
F4550	Phacolytic glaucoma	0
F4551	Pseudoexfoliation glaucoma	0
F455z	Glaucoma associated with disorders of the lens NOS	0
F456.	Glaucoma associated with other ocular disorders	0
F4560	Glaucoma due to unspecified ocular disorder	0
F4561	Glaucoma due to pupillary block	0
F4562	Glaucoma due to ocular inflammation	0
F4563	Glaucoma due to ocular vascular disorder	0
F4564	Glaucoma due to ocular tumour or cyst	0
F4565	Glaucoma due to ocular trauma	0
F4566	Neovascular glaucoma	0
F456z	Glaucoma associated with other ocular disorders NOS	0
F45y.	Other specified forms of glaucoma	0
F45y0	Hypersecretion glaucoma	0
F45y1	Glaucoma due to episode of increased venous pressure	0
F45y2	Low tension glaucoma	0
F45yz	Other specified glaucoma NOS	0
F45z.	Glaucoma NOS	0
F4631	Glaucomatous subcapsular flecks	0
F4H14	Optic disc glaucomatous atrophy	0
FyuG.	[X]Glaucoma	0
FyuG0	[X]Other glaucoma	0
FyuG1	[X]Glaucoma in endocrine, nutritional and metabolic diseases classified elsewhere	0
FyuG2	[X]Glaucoma in other diseases classified elsewhere	0
P3200	Congenital glaucoma	0
Q20y7	Traumatic glaucoma due to birth trauma	0

CONCEPT: Gout

READ_CODE	DESCRIPTION_SAIL	SENSITIVE_CODE
1443.	H/O: gout	0
2D52.	O/E - auricle of ear - tophi	0
669..	Gout monitoring	0
6691.	Initial gout assessment	0
6692.	Follow-up gout assessment	0
6693.	Joints gout affected	0
6695.	Date gout treatment started	0
6696.	Date of last gout attack	0
6697.	Gout associated problems	0
6698.	Gout drug side effects	0
6699.	Gout treatment changed	0
669A.	Date gout treatment stopped	0
669Z.	Gout monitoring NOS	0
C34..	Gout	0
C340.	Gouty arthropathy	0
C341.	Gouty nephropathy	0
C3410	Gouty nephropathy unspecified	0
C341z	Gouty nephropathy NOS	0
C342.	Idiopathic gout	0

C343.	Lead-induced gout	0
C344.	Drug-induced gout	0
C345.	Gout due to impairment of renal function	0
C346.	Acute exacerbation of gout	0
C34y.	Other specified gouty manifestation	0
C34y0	Gouty tophi of ear	0
C34y1	Gouty tophi of heart	0
C34y2	Gouty tophi of other sites	0
C34y3	Gouty iritis	0
C34y4	Gouty neuritis	0
C34y5	Gouty tophi of hand	0
C34y6	Gouty tophi of toe	0
C34yz	Other specified gouty manifestation NOS	0
C34z.	Gout NOS	0
G5573	Gouty tophi of heart	0
N023.	Gouty arthritis	0
N0230	Gouty arthritis of unspecified site	0
N0231	Gouty arthritis of the shoulder region	0
N0232	Gouty arthritis of the upper arm	0
N0233	Gouty arthritis of the forearm	0
N0234	Gouty arthritis of the hand	0
N0235	Gouty arthritis of the pelvic region and thigh	0
N0236	Gouty arthritis of the lower leg	0
N0237	Gouty arthritis of the ankle and foot	0
N0238	Gouty arthritis of toe	0
N023x	Gouty arthritis of multiple sites	0
N023y	Gouty arthritis of other specified site	0
N023z	Gouty arthritis NOS	0
Nyu17	[X]Other secondary gout	0
Nyu1C	[X]Gouty arthropathy due to enzyme defects and other inherited disorders classified elsewhere	0

CONCEPT: Heart Valve Disorders

READ_CODE	DESCRIPTION_SAIL	SENSITIVE_CODE
0Z1A.	Valveman	0
14S4.	H/O: heart valve recipient	0
14T3.	H/O: artificial heart valve	0
24G..	Aortic valve gradient	0
70101	Creation of ventriculovascular anastomosis	0
70114	Renewal of valve of cerebroventricular shunt	0
70160	Drainage of ventricle of brain NEC	0
74493	Insertion of endobronchial valve	0
744D1	Changing of tracheooesophageal valve	0
74583	Bag valve mask ventilation	0
76112	Partial gastrectomy and anastomosis of stomach to jejunum NEC	0
79020	Correction of tetralogy of Fallot using valved right ventricular outflow conduit	0
79025	Repair of tetralogy of Fallot with absent pulmonary valve	0
790D.	Creation of valved cardiac conduit	0
790D0	Creation of valved conduit between atrium and ventricle of heart	0
790D1	Creation of valved conduit between right atrium and pulmonary artery	0
790D2	Creation of valved conduit between right ventricle of heart and pulmonary artery	0
790D3	Creation of valved conduit between left ventricle of heart and aorta	0
790D4	Revision of valved cardiac conduit	0
790D6	Creation of valved conduit between left ventricle of heart and pulmonary artery	0
790D7	Replacement of valved cardiac conduit	0
790Dy	Other specified creation of valved cardiac conduit	0
790Dz	Creation of valved cardiac conduit NOS	0
790M3	Radical aortopulmonary reconstruction with right ventricle to pulmonary arterial valveless conduit	0
790M8	Aortopulmonary reconstruction with right ventricle to pulmonary arterial valveless conduit	0
791..	Valves of heart and adjacent structures operations	0
7910.	Plastic repair of mitral valve	0
79100	Allograft replacement of mitral valve	0
79101	Xenograft replacement of mitral valve	0
79102	Prosthetic replacement of mitral valve	0
79103	Replacement of mitral valve NEC	0
79104	Mitral valvuloplasty NEC	0
7910y	Other specified plastic repair of mitral valve	0
7910z	Plastic repair of mitral valve NOS	0
7911.	Plastic repair of aortic valve	0
79110	Allograft replacement of aortic valve	0
79111	Xenograft replacement of aortic valve	0
79112	Prosthetic replacement of aortic valve	0
79113	Replacement of aortic valve NEC	0
79114	Aortic valvuloplasty NEC	0
79115	Transapical aortic valve implantation	0
79116	Transluminal aortic valve implantation	0
7911y	Other specified plastic repair of aortic valve	0
7911z	Plastic repair of aortic valve NOS	0
7912.	Plastic repair of tricuspid valve	0
79120	Allograft replacement of tricuspid valve	0
79121	Xenograft replacement of tricuspid valve	0
79122	Prosthetic replacement of tricuspid valve	0
79123	Replacement of tricuspid valve NEC	0
79124	Repositioning of tricuspid valve	0
79125	Tricuspid valvuloplasty NEC	0
7912y	Other specified plastic repair of tricuspid valve	0
7912z	Plastic repair of tricuspid valve NOS	0
7913.	Plastic repair of pulmonary valve	0
79130	Allograft replacement of pulmonary valve	0
79131	Xenograft replacement of pulmonary valve	0
79132	Prosthetic replacement of pulmonary valve	0
79133	Replacement of pulmonary valve NEC	0
79134	Pulmonary valvuloplasty NEC	0
7913y	Other specified plastic repair of pulmonary valve	0
7913z	Plastic repair of pulmonary valve NOS	0
7914.	Plastic repair of unspecified valve of heart	0
79140	Allograft replacement of valve of heart NEC	0
79141	Xenograft replacement of valve of heart NEC	0
79142	Prosthetic replacement of valve of heart NEC	0
79143	Replacement of valve of heart NEC	0
79144	Valvuloplasty of heart NEC	0
79145	Truncal valve repair	0
79146	Replacement of truncal valve	0

7914y	Other specified plastic repair of unspecified valve of heart	0
7914z	Plastic repair of unspecified valve of heart NOS	0
7915.	Revision of plastic repair of valve of heart	0
79150	Revision of plastic repair of mitral valve	0
79151	Revision of plastic repair of aortic valve	0
79152	Revision of plastic repair of tricuspid valve	0
79153	Revision of plastic repair of pulmonary valve	0
79154	Revision of plastic repair of truncal valve	0
7915y	Other specified revision of plastic repair of valve of heart	0
7915z	Revision of plastic repair of valve of heart NOS	0
7916.	Open incision of heart valve	0
79160	Open mitral valvotomy	0
79161	Open aortic valvotomy	0
79162	Open tricuspid valvotomy	0
79163	Open pulmonary valvotomy	0
79164	Open truncal valvotomy	0
7916y	Other specified open specified incision of valve of heart	0
7916z	Open incision of valve of heart NOS	0
7917.	Closed incision of heart valve	0
79170	Closed mitral valvotomy	0
79171	Closed aortic valvotomy	0
79172	Closed tricuspid valvotomy	0
79173	Closed pulmonary valvotomy	0
7917y	Other specified closed incision of valve of heart	0
7917z	Closed incision of valve of heart NOS	0
7918.	Other open operations on valve of heart	0
79180	Annuloplasty of mitral valve	0
79181	Annuloplasty of tricuspid valve	0
79182	Annuloplasty of valve of heart NEC	0
79183	Excision of vegetations of valve of heart	0
79184	Closure of tricuspid valve	0
79185	Closure of pulmonary valve	0
7918y	Other specified other open operation on valve of heart	0
7918z	Other open operation on valve of heart NOS	0
7919.	Therapeutic transluminal operations on valve of heart	0
79190	Percutaneous transluminal mitral valvotomy	0
79191	Percutaneous transluminal aortic valvotomy	0
79192	Percutaneous transluminal tricuspid valvotomy	0
79193	Percutaneous transluminal pulmonary valvotomy	0
79194	Percutaneous transluminal valvuloplasty	0
79195	Percutaneous transluminal pulmonary valve perforation and dilation	0
79196	Percutaneous transluminal pulmonary valve replacement	0
7919y	Other specified therapeutic transluminal operation on valve of heart	0
7919z	Therapeutic transluminal operation on heart valve NOS	0
791A.	Removal of obstruction from structure adjacent to valve of heart	0
791A0	Infundibulectomy of heart using patch	0
791A1	Infundibulectomy of heart NEC	0
791A2	Repair of subaortic stenosis	0
791A3	Repair of supraaortic stenosis	0
791A4	Excision of supramitral ring	0
791A5	Aortoventriculoplasty	0
791Ay	Other specified removal of obstruction from structure adjacent to valve of heart	0
791Az	Removal of obstruction from structure adjacent to valve of heart NOS	0
791B.	Other operations on structure adjacent to valve of heart	0
791B0	Operation on papillary muscle	0
791B1	Operation on chordae tendineae	0
791B2	Operations on mitral subvalvar apparatus	0
791B3	Closure of aorto-left ventricular tunnel	0
791B5	Repair of aortic sinus of Valsalva aneurysm	0
791By	Other specified operation on structure adjacent to valve of heart	0
791Bz	Operation on structure adjacent to valve of heart NOS	0
791C0	Aortic root replacement using pulmonary valve autograft with right ventricle to pulmonary artery valved conduit	0
791C1	Aortic root replacement using pulmonary valve autograft with right ventricle to pulmonary artery valved conduit and aortoventriculoplasty	0
791C5	Aortoventriculoplasty with pulmonary valve autograft	0
791D.	Excision of valve of heart	0
791D0	Tricuspid valvectomy	0
791D1	Pulmonary valvectomy	0
791Dy	Other specified excision of valve of heart	0
791Dz	Excision of valve of heart NOS	0
791y.	Other specified operations on heart valve or adjacent structures	0
791z.	Heart valve and adjacent structures operations NOS	0
79310	Inspection of valve of heart	0
7A65.	Repair of valve of vein	0
7A650	Transposition of valve of vein	0
7A651	Interposition of valve of vein	0
7A65y	Other specified repair of valve of vein	0
7A65z	Repair of valve of vein NOS	0
7B402	Open excision of urethral lesion	0
7B435	Endoscopic destruction of posterior urethral valve	0
7B43A	Endoscopic destruction of urethral valves	0
7B456	Hook ablation of posterior urethral valve	0
7N40.	[SO]Valve of heart	0
7N400	[SO]Mitral valve	0
7N401	[SO]Aortic valve	0
7N402	[SO]Tricuspid valve	0
7N403	[SO]Pulmonary valve	0
7N404	[SO]Truncal valve	0
7N40y	[SO]Specified valve of heart NEC	0
7N40z	[SO]Valve of heart NEC	0
A932.	Syphilitic endocarditis	1
A9320	Syphilitic endocarditis of unspecified valve	1
A9321	Syphilitic endocarditis of mitral valve	1
A9322	Syphilitic endocarditis of aortic valve	1
A9323	Syphilitic endocarditis of tricuspid valve	1
A9324	Syphilitic endocarditis of pulmonary valve	1
A932z	Syphilitic endocarditis of heart valve NOS	1
B713.	Benign neoplasm of colon	0
B7139	Benign neoplasm of ileocaecal valve	0
G11..	Mitral valve diseases	0
G110.	Mitral stenosis	0
G111.	Rheumatic mitral insufficiency	0
G112.	Mitral stenosis with insufficiency	0
G113.	Nonrheumatic mitral valve stenosis	0

G114.	Ruptured mitral valve cusp	0
G11z.	Mitral valve disease NOS	0
G12..	Rheumatic aortic valve disease	0
G120.	Rheumatic aortic stenosis	0
G121.	Rheumatic aortic insufficiency	0
G122.	Rheumatic aortic stenosis with insufficiency	0
G12z.	Rheumatic aortic valve disease NOS	0
G13..	Diseases of mitral and aortic valves	0
G130.	Mitral and aortic stenosis	0
G131.	Mitral stenosis and aortic insufficiency	0
G132.	Mitral insufficiency and aortic stenosis	0
G133.	Mitral and aortic incompetence	0
G13y.	Multiple mitral and aortic valve involvement	0
G13z.	Mitral and aortic valve disease NOS	0
G14..	Other chronic rheumatic endocardial disease	0
G140.	Tricuspid valve disease NEC	0
G1400	Rheumatic tricuspid stenosis	0
G1401	Rheumatic tricuspid insufficiency	0
G1402	Rheumatic tricuspid stenosis and insufficiency	0
G1403	Tricuspid stenosis, cause unspecified	0
G1404	Tricuspid insufficiency, cause unspecified	0
G1405	Tricuspid stenosis and insufficiency, cause unspecified	0
G140z	Rheumatic tricuspid valve disease NOS	0
G141.	Rheumatic pulmonary valve disease	0
G1410	Rheumatic pulmonary stenosis	0
G1411	Rheumatic pulmonary insufficiency	0
G1412	Rheumatic pulmonary stenosis and insufficiency	0
G141z	Rheumatic pulmonary valve disease NOS	0
G14z.	Rheumatic endocarditis NOS	0
G1y..	Other specified chronic rheumatic heart disease	0
G54..	Other diseases of endocardium	0
G540.	Mitral valve incompetence	0
G5400	Mitral incompetence, non-rheumatic	0
G5401	Mitral incompetence, cause unspecified	0
G5402	Mitral valve prolapse	0
G5403	Mitral valve leaf prolapse	0
G540z	Mitral valve disorders NOS	0
G541.	Aortic valve disorders	0
G5410	Aortic incompetence, non-rheumatic	0
G5411	Aortic stenosis, non-rheumatic	0
G5412	Aortic incompetence alone, cause unspecified	0
G5413	Aortic stenosis alone, cause unspecified	0
G5414	Aortic valve stenosis with insufficiency	0
G5415	Aortic stenosis	0
G5416	Aortic valve sclerosis	0
G5417	Aortic valve calcification	0
G541z	Aortic valve disorders NOS	0
G542.	Tricuspid valve disorders, non-rheumatic	0
G5420	Tricuspid incompetence, non-rheumatic	0
G5421	Tricuspid stenosis, non-rheumatic	0
G5422	Nonrheumatic tricuspid valve stenosis with insufficiency	0
G542X	Nonrheumatic tricuspid valve disorder, unspecified	0
G542z	Tricuspid valve disorders NOS	0
G543.	Pulmonary valve disorders	0
G5430	Pulmonary incompetence, non-rheumatic	0
G5431	Pulmonary stenosis, non-rheumatic	0
G5432	Pulmonary incompetence, cause unspecified	0
G5433	Pulmonary stenosis, cause unspecified	0
G5434	Pulmonary valve stenosis with insufficiency	0
G543z	Pulmonary valve disorders NOS	0
G544.	Multiple valve diseases	0
G5440	Disorders of both aortic and tricuspid valves	0
G5441	Disorders of both mitral and tricuspid valves	0
G5442	Combined disorders of mitral, aortic and tricuspid valves	0
G544X	Multiple valve disease, unspecified	0
G54z.	Endocarditis, valve unspecified	0
G54z0	Incompetence of unspecified heart valve	0
G54z1	Stenosis of unspecified heart valve	0
G54z2	Chronic cardiac valvulitis NOS	0
G54z3	Endocarditis, valve unspecified, OS	0
G54z5	Valvular heart disease	0
G54zz	Endocarditis, valve unspecified, NOS	0
G5804	Congestive heart failure due to valvular disease	0
Gyu10	[X]Other mitral valve diseases	0
Gyu11	[X]Other rheumatic aortic valve diseases	0
Gyu12	[X]Other tricuspid valve diseases	0
Gyu13	[X]Other multiple valve diseases	0
Gyu15	[X]Multiple valve disease, unspecified	0
Gyu55	[X]Other nonrheumatic mitral valve disorders	0
Gyu56	[X]Other aortic valve disorders	0
Gyu57	[X]Other nonrheumatic tricuspid valve disorders	0
Gyu58	[X]Other pulmonary valve disorders	0
Gyu59	[X]Mitral valve disorders in diseases classified elsewhere	0
Gyu5A	[X]Aortic valve disorders in diseases classified elsewhere	0
Gyu5B	[X]Tricuspid valve disorders in diseases classified elsewhere	0
Gyu5C	[X]Pulmonary valve disorders in diseases classified elsewhere	0
Gyu5D	[X]Multiple valve disorders in diseases classified elsewhere	0
Gyu5E	[X]Endocarditis, valve unspecified, in diseases classified elsewhere	0
Gyu5f	[X]Nonrheumatic tricuspid valve disorder, unspecified	0
P60..	Pulmonary valve anomalies	0
P600.	Pulmonary valve anomaly, unspecified	0
P601.	Congenital atresia of the pulmonary valve	0
P6010	Hypoplasia of pulmonary valve	0
P601z	Congenital atresia of pulmonary valve NOS	0
P602.	Congenital pulmonary stenosis	0
P6021	Congenital fusion of pulmonary valve segment	0
P602z	Congenital pulmonary stenosis NOS	0
P60z.	Other pulmonary valve anomalies	0
P60z0	Congenital insufficiency of the pulmonary valve	0
P60z2	Supernumerary pulmonary valve cusps	0
P60zz	Other pulmonary valve anomaly NOS	0
P611.	Congenital tricuspid stenosis	0
P62..	Ebstein's anomaly	0

P63..	Congenital aortic valve stenosis	0
P64..	Congenital aortic valve insufficiency	0
P640.	Congenital aortic valve insufficiency, unspecified	0
P641.	Bicuspid aortic valve	0
P64z.	Congenital aortic valve insufficiency NOS	0
P65..	Congenital mitral stenosis	0
P650.	Congenital mitral stenosis, unspecified	0
P651.	Fused commissure of the mitral valve	0
P652.	Parachute deformity of the mitral valve	0
P653.	Supernumerary cusps of the mitral valve	0
P65z.	Congenital mitral stenosis NOS	0
P66..	Congenital mitral insufficiency	0
P6W..	Congenital malformation of aortic and mitral valves unspecified	0
P6X..	Congenital malformation of tricuspid valve, unspecified	0
P6y0.	Subaortic stenosis	0
P6y2.	Pulmonary infundibular stenosis	0
P6yy.	Other specified heart anomalies	0
P6yy7	Atresia of heart valve NEC	0
P6yyB	Supernumerary heart valve cusps NEC	0
P6yyC	Fusion of mitral valve cusps	0
P6yyD	Fusion of heart valve cusps NEC	0
P6z0.	Unspecified anomaly of heart valve	0
P711.	Preductal coarctation of aorta	0
P712.	Postductal coarctation of aorta	0
P7224	Supra-valvular aortic stenosis	0
PD21.	Occlusion of ureter	0
PD62.	Congenital urethral valvular stricture	0
PD67.	Congenital posterior urethral valves	0
Pyu22	[X]Other congenital malformations of pulmonary valve	0
Pyu23	[X]Other congenital malformations of tricuspid valve	0
Pyu24	[X]Other congenital malformations of aortic and mitral valves	0
Pyu2G	[X]Congenital malformation of tricuspid valve, unspecified	0
Pyu2H	[X]Congenital malformation of aortic and mitral valves unspecified	0
SP002	Mechanical complication of heart valve prosthesis	0
SP004	Infection and inflammatory reaction due to cardiac valve prosthesis	0
SyuK6	[X]Other complications of cardiac and vascular prosthetic devices, implants and grafts	0
TB012	Implant of heart valve prosthesis as the cause of abnormal reaction of patient, or of later complication, without mention of misadventure at the time of operation	0
ZV422	[V]Heart valve transplanted	0
ZV433	[V]Has artificial heart valve	0
ZV452	[V]Has cerebrospinal fluid drainage device in situ	0
ZV45H	[V]Presence of prosthetic heart valve	0
ZVu6e	[X]Presence of other heart valve replacement	0

CONCEPT: HF (Heart Failure)

READ_CODE	DESCRIPTION_SAIL	SENSITIVE_CODE
14A6.	H/O: heart failure	0
14AM.	H/O: Heart failure in last year	0
14S3.	H/O: heart recipient	0
14T7.	H/O: artificial heart	0
1O1..	Heart failure confirmed	0
23E1.	O/E - pulmonary oedema	0
2JZ..	On optimal heart failure therapy	0
33BA.	Impaired left ventricular function	0
388D.	New York Heart Association classification of heart failure symptoms	0
585f.	Echocardiogram shows left ventricular systolic dysfunction	0
585g.	Echocardiogram shows left ventricular diastolic dysfunction	0
662T.	Congestive heart failure monitoring	0
662W.	Heart failure annual review	0
662f.	New York Heart Association classification - class I	0
662g.	New York Heart Association classification - class II	0
662h.	New York Heart Association classification - class III	0
662i.	New York Heart Association classification - class IV	0
662p.	Heart failure 6 month review	0
7900.	Transplantation of heart and lung	0
79000	Allotransplantation of heart and lung	0
79001	Revision of transplantation of heart and lung	0
7900y	Other specified transplantation of heart and lung	0
7900z	Transplantation of heart and lung NOS	0
7901.	Other transplantation of heart	0
79010	Allotransplantation of heart NEC	0
79011	Xenotransplantation of heart	0
79012	Implantation of prosthetic heart	0
79013	Piggyback transplantation of heart	0
79015	Revision of transplantation of heart NEC	0
7901y	Other specified other transplantation of heart	0
7901z	Other transplantation of heart NOS	0
8B29.	Cardiac failure therapy	0
8CeC.	Preferred place of care for next exacerbation of heart failure	0
8H2S.	Admit heart failure emergency	0
8HBE.	Heart failure follow-up	0
8HHb.	Referral to heart failure nurse	0
8HHz.	Referral to heart failure exercise programme	0
8HTL.	Referral to heart failure clinic	0
8HTL0	Referral to rapid access heart failure clinic	0
8HgD.	Discharge from heart failure nurse service	0
8Hk0.	Referred to heart failure education group	0
8IE1.	Referral to heart failure exercise programme declined	0
9N0k.	Seen in heart failure clinic	0
9N2p.	Seen by community heart failure nurse	0
9N6T.	Referred by heart failure nurse specialist	0
9Or0.	Heart failure review completed	0
G1yz1	Rheumatic left ventricular failure	0
G21..	Hypertensive heart disease	0
G210.	Malignant hypertensive heart disease	0
G2100	Malignant hypertensive heart disease without congestive cardiac failure	0
G2101	Malignant hypertensive heart disease with congestive cardiac failure	0
G210z	Malignant hypertensive heart disease NOS	0
G211.	Benign hypertensive heart disease	0
G2110	Benign hypertensive heart disease without congestive cardiac failure	0
G2111	Benign hypertensive heart disease with congestive cardiac failure	0
G211z	Benign hypertensive heart disease NOS	0
G21z.	Hypertensive heart disease NOS	0

G21z1	Hypertensive heart disease NOS with congestive cardiac failure	0
G21zz	Hypertensive heart disease NOS	0
G23..	Hypertensive heart and renal disease	0
G230.	Malignant hypertensive heart and renal disease	0
G231.	Benign hypertensive heart and renal disease	0
G232.	Hypertensive heart and renal disease with (congestive) heart failure	0
G233.	Hypertensive heart and renal disease with renal failure	0
G234.	Hypertensive heart and renal disease with both (congestive) heart failure and renal failure	0
G23z.	Hypertensive heart and renal disease NOS	0
G400.	Acute cor pulmonale	0
G41z.	Chronic pulmonary heart disease NOS	0
G5540	Congestive cardiomyopathy	0
G5571	Beriberi heart disease	0
G58..	Heart failure	0
G580.	Congestive heart failure	0
G5800	Acute congestive heart failure	0
G5801	Chronic congestive heart failure	0
G5802	Decompensated cardiac failure	0
G5803	Compensated cardiac failure	0
G5804	Congestive heart failure due to valvular disease	0
G581.	Left ventricular failure	0
G5810	Acute left ventricular failure	0
G582.	Acute heart failure	0
G583.	Heart failure with normal ejection fraction	0
G584.	Right ventricular failure	0
G58z.	Heart failure NOS	0
G5y34	Ventricular hypertrophy	0
G5y4z	Post cardiac operation heart failure NOS	0
G5yy9	Left ventricular systolic dysfunction	0
G5yyA	Left ventricular diastolic dysfunction	0
G5yyD	Left ventricular cardiac dysfunction	0
Gyu5M	[X]Other hypertrophic cardiomyopathy	0
H54..	Pulmonary congestion and hypostasis	0
H541.	Pulmonary congestion	0
H5410	Chronic pulmonary oedema	0
H541z	Pulmonary oedema NOS	0
H54z.	Pulmonary congestion and hypostasis NOS	0
H584.	Acute pulmonary oedema unspecified	0
H584z	Acute pulmonary oedema NOS	0
L09y2	Cardiac failure following abortive pregnancy	1
L1280	Pre-existing hypertensive heart disease complicating pregnancy, childbirth and the puerperium	0
L1281	Pre-existing hypertensive heart and renal disease complicating pregnancy, childbirth and the puerperium	0
P69..	Left ventricular outflow tract obstruction	0
Q48y1	Congenital cardiac failure	0
Q490.	Neonatal cardiac failure	0
R2y10	[D]Cardiorespiratory failure	0
SP111	Cardiac insufficiency as a complication of care	0
TB000	Transplantation of heart as the cause of abnormal reaction of patient, or of later complication, without mention of misadventure at the time of operation	0
ZV421	[V]Heart transplanted	0
ZV432	[V]Has artificial heart	0

CONCEPT: HIV/AIDS

READ_CODE	DESCRIPTION_SAIL	SENSITIVE_CODE
43C3.	HTLV-3 antibody positive	1
43j7.	HIV 1 nucleic acid detection	1
4J3P.	Human immunodeficiency virus type 1 subtype identification	1
65QA.	AIDS carrier	1
A78..	Other viral or chlamydial diseases	1
A788.	Acquired immune deficiency syndrome	1
A7880	Acute human immunodeficiency virus infection	1
A7881	Asymptomatic human immunodeficiency virus infection	1
A7882	Human immunodeficiency virus infection with persistent generalised lymphadenopathy	1
A7883	Human immunodeficiency virus with constitutional disease	1
A7884	Human immunodeficiency virus with neurological disease	1
A7885	Human immunodeficiency virus infection with secondary clinical infectious disease	1
A7886	Human immunodeficiency virus with secondary cancers	1
A788U	HIV disease resulting in haematological and immunological abnormalities, not elsewhere classified	1
A788V	HIV disease resulting in multiple diseases classified elsewhere	1
A788W	HIV disease resulting in unspecified malignant neoplasm	1
A788X	HIV disease resulting in unspecified infectious and parasitic disease	1
A788y	Human immunodeficiency virus with other clinical findings	1
A788z	Acquired human immunodeficiency virus infection syndrome NOS	1
A789.	Human immunodef virus resulting in other disease	1
A7890	HIV disease resulting in mycobacterial infection	1
A7891	HIV disease resulting in cytomegaloviral disease	1
A7892	HIV disease resulting in candidiasis	1
A7893	HIV disease resulting in Pneumocystis carinii pneumonia	1
A7894	HIV disease resulting in multiple infections	1
A7895	HIV disease resulting in Kaposi's sarcoma	1
A7896	HIV disease resulting in Burkitt's lymphoma	1
A7897	HIV disease resulting in other types of non-Hodgkin's lymphoma	1
A7898	HIV disease resulting in multiple malignant neoplasms	1
A7899	HIV disease resulting in lymphoid interstitial pneumonitis	1
A789A	HIV disease resulting in wasting syndrome	1
A789X	HIV disease resulting in other malignant neoplasms of lymphoid, haematopoietic and related tissue	1
AyuC.	[X]Human immunodeficiency virus disease	1
AyuC0	[X]HIV disease resulting in other bacterial infections	1
AyuC1	[X]HIV disease resulting in other viral infections	1
AyuC2	[X]HIV disease resulting in other mycoses	1
AyuC3	[X]HIV disease resulting in multiple infections	1
AyuC4	[X]Hiv disease resulting in other infectious and parasitic diseases	1
AyuC5	[X]Hiv disease resulting in unspecified infectious and parasitic disease	1
AyuC6	[X]HIV disease resulting in other non-Hodgkin's lymphoma	1
AyuC7	[X]Hiv disease resulting in other malignant neoplasms of lymphoid, haematopoietic and related tissue	1
AyuC8	[X]HIV disease resulting in other malignant neoplasms	1
AyuC9	[X]HIV disease resulting in unspecified malignant neoplasm	1
AyuCA	[X]Hiv disease resulting in multiple diseases classified elsewhere	1
AyuCB	[X]Hiv disease resulting in haematological and immunological abnormalities, not elsewhere classified	1
AyuCC	[X]HIV disease resulting in other specified conditions	1
AyuCD	[X]Unspecified human immunodeficiency virus [HIV] disease	1
Eu024	[X]Dementia in human immunodef virus [HIV] disease	1
L179.	Human immunodeficiency [HIV] disease complicating pregnancy, childbirth and the puerperium	1

R109.	[D]Laboratory evidence of human immunodeficiency virus [HIV]	1
ZV01A	[V]Asymptomatic human immunodeficiency virus infection status	1

CONCEPT: Hypertension

READ_CODE	DESCRIPTION_SAIL	SENSITIVE_CODE
14A2.	H/O: hypertension	0
21261	Hypertension resolved	0
212K.	Hypertension resolved	0
61462	Hypertension induced by oral contraceptive pill	0
6624.	Borderline hyperten:yearly obs	0
6627.	Good hypertension control	0
6628.	Poor hypertension control	0
6629.	Hypertension:follow-up default	0
662F.	Hypertension treatm. started	0
662G.	Hypertensive treatm.changed	0
662H.	Hypertension treatm.stopped	0
662O.	On treatment for hypertension	0
662P.	Hypertension monitoring	0
662P0	Hypertension 9 month review	0
662b.	Moderate hypertension control	0
662c.	Hypertension six month review	0
662d.	Hypertension annual review	0
662q.	Trial reduction of antihypertensive therapy	0
662r.	Trial withdrawal of antihypertensive therapy	0
7Q01.	High cost hypertension drugs	0
7Q01y	Other specified high cost hypertension drugs	0
7Q01z	High cost hypertension drugs NOS	0
8B26.	Antihypertensive therapy	0
8BL0.	Patient on maximal tolerated antihypertensive therapy	0
8CR4.	Hypertension clinical management plan	0
8I3N.	Hypertension treatment refused	0
8IA5.	Trial withdrawal of antihypertensive therapy declined	0
8IA6.	Trial reduction of antihypertensive therapy declined	0
9N03.	Seen in hypertension clinic	0
9N1y2	Seen in hypertension clinic	0
9OI9.	Hypertens.monitor deleted	0
F4042	Blind hypertensive eye	0
F4213	Hypertensive retinopathy	0
G2...	Hypertensive disease	0
G20..	Essential hypertension	0
G200.	Malignant essential hypertension	0
G201.	Benign essential hypertension	0
G202.	Systolic hypertension	0
G203.	Diastolic hypertension	0
G20z.	Essential hypertension NOS	0
G21..	Hypertensive heart disease	0
G210.	Malignant hypertensive heart disease	0
G2100	Malignant hypertensive heart disease without congestive cardiac failure	0
G2101	Malignant hypertensive heart disease with congestive cardiac failure	0
G210z	Malignant hypertensive heart disease NOS	0
G211.	Benign hypertensive heart disease	0
G2110	Benign hypertensive heart disease without congestive cardiac failure	0
G2111	Benign hypertensive heart disease with congestive cardiac failure	0
G211z	Benign hypertensive heart disease NOS	0
G21z.	Hypertensive heart disease NOS	0
G21z0	Hypertensive heart disease NOS without congestive cardic failure	0
G21z1	Hypertensive heart disease NOS with congestive cardiac failure	0
G21zz	Hypertensive heart disease NOS	0
G22..	Hypertensive renal disease	0
G220.	Malignant hypertensive renal disease	0
G221.	Benign hypertensive renal disease	0
G222.	Hypertensive renal disease with renal failure	0
G22z.	Hypertensive renal disease NOS	0
G23..	Hypertensive heart and renal disease	0
G230.	Malignant hypertensive heart and renal disease	0
G231.	Benign hypertensive heart and renal disease	0
G232.	Hypertensive heart and renal disease with (congestive) heart failure	0
G233.	Hypertensive heart and renal disease with renal failure	0
G234.	Hypertensive heart and renal disease with both (congestive) heart failure and renal failure	0
G23z.	Hypertensive heart and renal disease NOS	0
G24..	Secondary hypertension	0
G240.	Secondary malignant hypertension	0
G2400	Secondary malignant renovascular hypertension	0
G240z	Secondary malignant hypertension NOS	0
G241.	Secondary benign hypertension	0
G2410	Secondary benign renovascular hypertension	0
G241z	Secondary benign hypertension NOS	0
G244.	Hypertension secondary to endocrine disorders	0
G24z.	Secondary hypertension NOS	0
G24z0	Secondary renovascular hypertension NOS	0
G24z1	Hypertension secondary to drug	0
G24zz	Secondary hypertension NOS	0
G25..	Stage 1 hypertension (NICE - National Institute for Health and Clinical Excellence 2011)	0
G250.	Stage 1 hypertension (NICE 2011) without evidence of end organ damage	0
G251.	Stage 1 hypertension (NICE 2011) with evidence of end organ damage	0
G26..	Severe hypertension (NICE - National Institute for Health and Clinical Excellence 2011)	0
G27..	Hypertension resistant to drug therapy	0
G28..	Stage 2 hypertension (NICE - National Institute for Health and Clinical Excellence 2011)	0
G2y..	Other specified hypertensive disease	0
G2z..	Hypertensive disease NOS	0
G672.	Hypertensive encephalopathy	0
Gyu2.	[X]Hypertensive diseases	0
Gyu20	[X]Other secondary hypertension	0
Gyu21	[X]Hypertension secondary to other renal disorders	0
L120.	Benign essential hypertension complicating pregnancy, childbirth and the puerperium	0
L122.	Other pre-existing hypertension in preg/childbirth/puerp	0
L1220	Other pre-existing hypertension complicating pregnancy, childbirth and the puerperium unspecified	0
L1221	Other pre-existing hypertension complicating pregnancy, childbirth and the puerperium - delivered	0
L1223	Other pre-existing hypertension complicating pregnancy, childbirth and the puerperium - not delivered	0
L1224	Other pre-existing hypertension complicating pregnancy, childbirth and the puerperium with postnatal complication	0
L122z	Other pre-existing hypertension complicating pregnancy, childbirth and the puerperium NOS	0
L127z	Pre-eclampsia or eclampsia with pre-existing hypertension NOS	0

L128.	Pre-existing hypertension complicating pregnancy, childbirth and puerperium	0
L1280	Pre-existing hypertensive heart disease complicating pregnancy, childbirth and the puerperium	0
Q000.	Fetus or neonate affected by maternal hypertensive disease	0
SLC6.	Other hypertensive agent poisoning	0
TJC7.	Adverse reaction to other antihypertensives	0
TJC7z	Adverse reaction to antihypertensives NOS	0
U60C5	[X]Other antihypertensive drugs causing adverse effects in therapeutic use, not elsewhere classified	0

CONCEPT: Hyperthyroidism

READ_CODE	DESCRIPTION_SAIL	SENSITIVE_CODE
1431.	H/O: hyperthyroidism	0
212P.	Hyperthyroidism resolved	0
C02..	Thyrotoxicosis	0
C020.	Toxic diffuse goitre	0
C0200	Toxic diffuse goitre with no crisis	0
C0201	Toxic diffuse goitre with crisis	0
C020z	Toxic diffuse goitre NOS	0
C021.	Toxic uninodular goitre	0
C0210	Toxic uninodular goitre with no crisis	0
C0211	Toxic uninodular goitre with crisis	0
C021z	Toxic uninodular goitre NOS	0
C022.	Toxic multinodular goitre	0
C0220	Toxic multinodular goitre with no crisis	0
C0221	Toxic multinodular goitre with crisis	0
C022z	Toxic multinodular goitre NOS	0
C023.	Toxic nodular goitre unspecified	0
C0230	Toxic nodular goitre unspecified with no crisis	0
C0231	Toxic nodular goitre unspecified with crisis	0
C023z	Toxic nodular goitre NOS	0
C024.	Thyrotoxicosis from ectopic thyroid nodule	0
C0240	Thyrotoxicosis from ectopic thyroid nodule with no crisis	0
C0241	Thyrotoxicosis from ectopic thyroid nodule with crisis	0
C024z	Thyrotoxicosis from ectopic thyroid nodule NOS	0
C02y.	Thyrotoxicosis of other specified origin	0
C02y0	Thyrotoxicosis of other specified origin with no crisis	0
C02y1	Thyrotoxicosis of other specified origin with crisis	0
C02yz	Thyrotoxicosis of other specified origin NOS	0
C02z.	Thyrotoxicosis without mention of goitre or other cause	0
C02z0	Thyrotoxicosis without mention of goitre or other cause with no crisis	0
C02z1	Thyrotoxicosis without mention of goitre or other cause with crisis	0
C02zz	Thyrotoxicosis NOS	0
F4G20	Thyrotoxic exophthalmos	0
G5575	Thyrotoxic heart disease	0

CONCEPT: Hypothyroidism

READ_CODE	DESCRIPTION_SAIL	SENSITIVE_CODE
1432.	H/O: hypothyroidism	0
66BB.	Hypothyroidism annual review	0
71100	Total thyroidectomy	0
71110	Excision of substernal thyroid tissue	0
8CR5.	Hypothyroidism clinical management plan	0
9Oj..	Hypothyroidism monitoring administration	0
9Oj0.	Hypothyroidism monitoring first letter	0
9Oj1.	Hypothyroidism monitoring second letter	0
9Oj2.	Hypothyroidism monitoring third letter	0
9Oj3.	Hypothyroidism monitoring verbal invite	0
9Oj4.	Hypothyroidism monitoring telephone invitation	0
C03..	Congenital hypothyroidism	0
C031.	Goitrous cretin	0
C03y.	Other specified congenital hypothyroidism	0
C03y0	Congenital hypothyroidism with diffuse goitre	0
C03y1	Congenital hypothyroidism without goitre	0
C03z.	Congenital hypothyroidism NOS	0
C04..	Acquired hypothyroidism	0
C040.	Postsurgical hypothyroidism	0
C041.	Other postablative hypothyroidism	0
C0410	Irradiation hypothyroidism	0
C041z	Postablative hypothyroidism NOS	0
C042.	Iodine hypothyroidism	0
C043.	Other iatrogenic hypothyroidism	0
C0430	Hypothyroidism resulting from para-aminosalicylic acid	0
C0431	Hypothyroidism resulting from phenylbutazone	0
C0432	Hypothyroidism resulting from resorcinol	0
C043z	Iatrogenic hypothyroidism NOS	0
C044.	Postinfectious hypothyroidism	0
C045.	Acquired atrophy of thyroid	0
C046.	Autoimmune myxoedema	0
C04y.	Other acquired hypothyroidism	0
C04z.	Hypothyroidism NOS	0
C04z0	Premature puberty due to hypothyroidism	0
C04z1	Myxoedema coma	0
C06y1	Thyroid atrophy	0
C0A1.	Congenital iodine-deficiency syndrome, myxoedematous type	0
Cyu11	[X]Other sp cified hypothyroidism	0
F11x5	Cerebral degeneration due to myxoedema	0
F1441	Cerebellar ataxia due to myxoedema	0
F3814	Myasthenic syndrome due to hypothyroidism	0
F3953	Myopathy due to myxoedema	0
Fyu15	[X]Systemic atrophy affecting the central nervous system in myxoedema	0
Q4337	Neonatal jaundice with congenital hypothyroidism	0

CONCEPT: IBD (Inflammatory bowel disease)

READ_CODE	DESCRIPTION_SAIL	SENSITIVE_CODE
14C4.	H/O: colitis	0
J08z9	Orofacial Crohn's disease	0
J40..	Regional enteritis - Crohn's disease	0
J400.	Regional enteritis of the small bowel	0
J4000	Regional enteritis of the duodenum	0
J4001	Regional enteritis of the jejunum	0
J4002	Crohn's disease of the terminal ileum	0
J4003	Crohn's disease of the ileum unspecified	0

J4004	Crohn's disease of the ileum NOS	0
J4005	Exacerbation of Crohn's disease of small intestine	0
J400z	Crohn's disease of the small bowel NOS	0
J401.	Regional enteritis of the large bowel	0
J4010	Regional enteritis of the colon	0
J4011	Regional enteritis of the rectum	0
J4012	Exacerbation of Crohn's disease of large intestine	0
J401z	Crohn's disease of the large bowel NOS	0
J402.	Regional ileocolitis	0
J40z.	Regional enteritis NOS	0
J41..	Idiopathic proctocolitis	0
J410.	Ulcerative proctocolitis	0
J4100	Ulcerative ileocolitis	0
J4101	Ulcerative colitis	0
J4102	Ulcerative rectosigmoiditis	0
J4103	Ulcerative proctitis	0
J4104	Exacerbation of ulcerative colitis	0
J410z	Ulcerative proctocolitis NOS	0
J411.	Ulcerative (chronic) enterocolitis	0
J412.	Ulcerative (chronic) ileocolitis	0
J413.	Ulcerative pancolitis	0
J41y.	Other idiopathic proctocolitis	0
J4212	Chronic ischaemic enterocolitis	0
J4213	Chronic ischaemic colitis	0
J42z0	Ischaemic colitis NOS	0
J4302	Radiation enterocolitis	0
J4303	Radiation colitis	0
J4312	Toxic enterocolitis	0
J4313	Toxic colitis	0
J4322	Allergic enterocolitis	0
J4323	Allergic colitis	0
J4332	Dietetic enterocolitis	0
J4333	Dietetic colitis	0
J436.	Microscopic colitis	0
J4360	Collagenous colitis	0
J4361	Lymphocytic colitis	0
J437.	Colitis	0
J4z3.	Non-infective colitis NOS	0
J4z5.	Exacerbation of non-infective colitis	0
J4z6.	Indeterminate colitis	0
Jyu4.	[X]Noninfective enteritis and colitis	0
Jyu40	[X]Other Crohn's disease	0
Jyu41	[X]Other ulcerative colitis	0
N0310	Arthropathy in ulcerative colitis	0
N0311	Arthropathy in Crohn's disease	0
N0453	Juvenile arthritis in Crohn's disease	0
N0454	Juvenile arthritis in ulcerative colitis	0

CONCEPT: IBS (Irritable Bowel Syndrome)

READ_CODE	DESCRIPTION_SAIL	SENSITIVE_CODE
14CF.	History of irritable bowel syndrome	0
J521.	Irritable colon - Irritable bowel syndrome	0
J5210	Irritable bowel syndrome with diarrhoea	0
J529.	Generalised intestinal dysmotility	0
J52y.	Other functional disorders of the intestine	0
J52yz	Other functional disorder of the intestine NOS	0
J52z.	Functional gastrointestinal tract disorders NEC NOS	0
Jyu53	[X]Other specified functional disorders of intestine	0

CONCEPT: ILD (Inflammatory Lung Disease)

READ_CODE	DESCRIPTION_SAIL	SENSITIVE_CODE
H35..	Extrinsic allergic alveolitis	0
H350.	Farmers' lung	0
H351.	Bagassosis	0
H352.	Bird-fancier's lung	0
H3520	Budgerigar-fanciers' lung	0
H3521	Pigeon-fanciers' lung	0
H352z	Bird-fancier's lung NOS	0
H353.	Suberosis (cork-handlers' lung)	0
H354.	Malt workers' lung	0
H355.	Mushroom workers' lung	0
H356.	Maple bark strippers' lung	0
H35y.	Other allergic alveolitis	0
H35y0	Cheese-washers' lung	0
H35y1	Coffee-workers' lung	0
H35y2	Fish-meal workers' lung	0
H35y5	Pituitary snuff-takers' disease	0
H35y8	Air-conditioner and humidifier lung	0
H35yz	Other allergic alveolitis NOS	0
H35z.	Allergic alveolitis and pneumonitis NOS	0
H35z0	Allergic extrinsic alveolitis NOS	0
H35zz	Allergic alveolitis and pneumonitis NOS	0
H4...	Lung disease due to external agents	0
H40..	Coal workers' pneumoconiosis	0
H42..	Silica and silicate pneumoconiosis	0
H420.	Talc pneumoconiosis	0
H421.	Simple silicosis	0
H422.	Complicated silicosis	0
H423.	Massive silicotic fibrosis	0
H42z.	Silica pneumoconiosis NOS	0
H43..	Pneumoconiosis due to other inorganic dust	0
H431.	Bauxite fibrosis of lung	0
H432.	Berylliosis	0
H433.	Graphite fibrosis of lung	0
H434.	Siderosis	0
H435.	Stannosis	0
H43z.	Pneumoconiosis due to inorganic dust NOS	0
H45..	Pneumoconiosis NOS	0
H450.	Pneumoconiosis associated with tuberculosis	0
H4642	Chronic pulmonary fibrosis due to chemical fumes	0
H4y10	Chronic pulmonary fibrosis following radiation	0

H4y2.	Drug-induced interstitial lung disorders	0
H4y20	Acute drug-induced interstitial lung disorders	0
H4y21	Chronic drug-induced interstitial lung disorders	0
H55..	Postinflammatory pulmonary fibrosis	0
H563.	Idiopathic fibrosing alveolitis	0
H5631	Diffuse pulmonary fibrosis	0
H5632	Pulmonary fibrosis	0
H5633	Usual interstitial pneumonitis	0
H563z	Idiopathic fibrosing alveolitis NOS	0
H58y3	Interstitial lung disease NEC	0
H58y5	Respiratory bronchiolitis associated interstitial lung disease	0
H58y6	Interstitial lung disease due to collagen vascular disease	0
H58y7	Interstitial lung disease due to connective tissue disease	0
Hyu40	[X]Pneumoconiosis due to other dust containing silica	0
Hyu48	[X]Chronic and other pulmonary manifestations due to radiation	0
Hyu5.	[X]Other respiratory diseases principally affecting the interstitium	0
Hyu50	[X]Other interstitial pulmonary diseases with fibrosis	0
Hyu51	[X]Other specified interstitial pulmonary diseases	0
N04y0	Rheumatoid lung	0
Q3171	Prematurity with interstitial pulmonary fibrosis	0

CONCEPT: Learning disability

READ_CODE	DESCRIPTION_SAIL	SENSITIVE_CODE
12K6.	Maternal learning disability	0
12L5.	Paternal learning disability	0
13Z4E	Learning difficulties	0
1IN..	Learning disability excluded	0
69DB.	Learning disability health examination	0
918W.	Carer of a person with learning disability	0
918e.	On learning disability register	0
949H.	Died in learning disability unit	0
9HB..	Learning disabilities administration status	0
9HB1.	Learning disabilities health action plan offered	0
9HB2.	Learning disabilities health action plan reviewed	0
9HB3.	Learning disabilities health assessment	0
9HB4.	Learning disabilities health action plan completed	0
9HB5.	Learning disabilities annual health assessment	0
E2F2.	Other specific learning difficulty	0
E3...	Mental retardation	0
E30..	Mild mental retardation, IQ in range 50-70	0
E31..	Other specified mental retardation	0
E310.	Moderate mental retardation, IQ in range 35-49	0
E311.	Severe mental retardation, IQ in range 20-34	0
E312.	Profound mental retardation with IQ less than 20	0
E31z.	Other specified mental retardation NOS	0
E3y..	Other specified mental retardation	0
E3z..	Mental retardation NOS	0
Eu7..	[X]Mental retardation	0
Eu70.	[X]Mild mental retardation	0
Eu700	[X]Mild mental retardation with the statement of no, or minimal, impairment of behaviour	0
Eu701	[X]Mild mental retardation, significant impairment of behaviour requiring attention or treatment	0
Eu70y	[X]Mild mental retardation, other impairments of behaviour	0
Eu70z	[X]Mild mental retardation without mention of impairment of behaviour	0
Eu71.	[X]Moderate mental retardation	0
Eu710	[X]Moderate mental retardation with the statement of no, or minimal, impairment of behaviour	0
Eu711	[X]Moderate mental retardation, significant impairment of behaviour requiring attention or treatment	0
Eu71y	[X]Mod retard oth behav impair	0
Eu71z	[X]Moderate mental retardation without mention of impairment of behaviour	0
Eu72.	[X]Severe mental retardation	0
Eu720	[X]Severe mental retardation with the statement of no, or minimal, impairment of behaviour	0
Eu721	[X]Severe mental retardation, significant impairment of behaviour requiring attention or treatment	0
Eu72y	[X]Severe mental retardation, other impairments of behaviour	0
Eu72z	[X]Severe mental retardation without mention of impairment of behaviour	0
Eu73.	[X]Profound mental retardation	0
Eu730	[X]Profound mental retardation with the statement of no, or minimal, impairment of behaviour	0
Eu731	[X]Profound mental retardation, significant impairment of behaviour requiring attention or treatment	0
Eu73y	[X]Profound mental retardation, other impairments of behaviour	0
Eu73z	[X]Profound mental retardation without mention of impairment of behaviour	0
Eu7y.	[X]Other mental retardation	0
Eu7y0	[X]Other mental retardation with the statement of no, or minimal, impairment of behaviour	0
Eu7y1	[X]Other mental retardation, significant impairment of behaviour requiring attention or treatment	0
Eu7yy	[X]Other mental retardation, other impairments of behaviour	0
Eu7yz	[X]Other mental retardation without mention of impairment of behaviour	0
Eu7z.	[X]Unspecified mental retardation	0
Eu7z0	[X]Unspecified mental retardation with the statement of no, or minimal, impairment of behaviour	0
Eu7z1	[X]Unspecified mental retardation, significant impairment of behaviour requiring attention or treatment	0
Eu7zy	[X]Unspecified mental retardation, other impairments of behaviour	0
Eu7zz	[X]Unspecified mental retardation without mention of impairment of behaviour	0
Eu814	[X]Moderate learning disability	0
Eu815	[X]Severe learning disability	0
Eu816	[X]Mild learning disability	0
Eu817	[X]Profound learning disability	0
Eu818	[X]Specific learning disability	0
Eu81z	[X]Developmental disorder of scholastic skills, unspecified	0

CONCEPT: Leukaemia

READ_CODE	DESCRIPTION_SAIL	SENSITIVE_CODE
1429.	H/O: * leukaemia	0
B624.	Leukaemic reticuloendotheliosis	0
B64..	Lymphoid leukaemia	0
B640.	Acute lymphoid leukaemia	0
B6400	B-cell acute lymphoblastic leukaemia	0
B641.	Chronic lymphoid leukaemia	0
B6410	B-cell chronic lymphocytic leukaemia	0
B6411	Clinical stage A chronic lymphocytic leukaemia	0
B6412	Clinical stage B chronic lymphocytic leukaemia	0
B6413	Clinical stage C chronic lymphocytic leukaemia	0
B642.	Subacute lymphoid leukaemia	0
B64y.	Other lymphoid leukaemia	0
B64y0	Aleukaemic lymphoid leukaemia	0
B64y1	Prolymphocytic leukaemia	0

B64y2	Adult T-cell leukaemia	0
B64y3	B-cell prolymphocytic leukaemia	0
B64y4	T-cell prolymphocytic leukaemia	0
B64y5	Adult T-cell lymphoma/leukaemia (HTLV-1-associated)	0
B64yz	Other lymphoid leukaemia NOS	0
B64z.	Lymphoid leukaemia NOS	0
B65..	Myeloid leukaemia	0
B650.	Acute myeloid leukaemia	0
B6500	Acute myeloid leukaemia with 11q23 abnormality	0
B6501	Acute myeloid leukaemia with multilineage dysplasia	0
B651.	Chronic myeloid leukaemia	0
B6510	Chronic eosinophilic leukaemia	0
B6511	Chronic myeloid leukaemia, BCR/ABL positive	0
B6512	Chronic neutrophilic leukaemia	0
B6513	Atypical chronic myeloid leukaemia, BCR/ABL negative	0
B651z	Chronic myeloid leukaemia NOS	0
B652.	Subacute myeloid leukaemia	0
B653.	Myeloid sarcoma	0
B6530	Chloroma	0
B6531	Granulocytic sarcoma	0
B653z	Myeloid sarcoma NOS	0
B654.	Acute myeloblastic leukaemia	0
B65y.	Other myeloid leukaemia	0
B65y0	Aleukaemic myeloid leukaemia	0
B65y1	Acute promyelocytic leukaemia	0
B65yz	Other myeloid leukaemia NOS	0
B65z.	Myeloid leukaemia NOS	0
B66..	Monocytic leukaemia	0
B660.	Acute monocytic leukaemia	0
B661.	Chronic monocytic leukaemia	0
B662.	Subacute monocytic leukaemia	0
B663.	Acute monoblastic leukaemia	0
B66y.	Other monocytic leukaemia	0
B66y0	Aleukaemic monocytic leukaemia	0
B66yz	Other monocytic leukaemia NOS	0
B66z.	Monocytic leukaemia NOS	0
B67..	Other specified leukaemia	0
B670.	Acute erythraemia and erythroleukaemia	0
B671.	Chronic erythraemia	0
B672.	Megakaryocytic leukaemia	0
B673.	Mast cell leukaemia	0
B674.	Acute panmyelosis	0
B675.	Acute myelofibrosis	0
B676.	Acute erythroid leukaemia	0
B677.	Myelodysplastic and myeloproliferative disease	0
B67y.	Other and unspecified leukaemia	0
B67y0	Lymphosarcoma cell leukaemia	0
B67yz	Other and unspecified leukaemia NOS	0
B67z.	Other specified leukaemia NOS	0
B68..	Leukaemia of unspecified cell type	0
B680.	Acute leukaemia NOS	0
B681.	Chronic leukaemia NOS	0
B682.	Subacute leukaemia NOS	0
B68y.	Other leukaemia of unspecified cell type	0
B68z.	Leukaemia NOS	0
B69..	Myelomonocytic leukaemia	0
B690.	Acute myelomonocytic leukaemia	0
B691.	Chronic myelomonocytic leukaemia	0
B692.	Subacute myelomonocytic leukaemia	0
B693.	Juvenile myelomonocytic leukaemia	0
B6y..	Malignant neoplasm of lymphatic or haematopoietic tissue otherwise specified	0
B6y1.	Myelosclerosis with myeloid metaplasia	0
B6z..	Malignant neoplasm lymphatic or haematopoietic tissue NOS	0
B937.	Neoplasm of uncertain behaviour of other lymphatic and haematopoietic tissue	0
BBr..	[M]Leukaemias	0
BBr0.	[M]Leukaemias unspecified	0
BBr00	[M]Leukaemia NOS	0
BBr01	[M]Acute leukaemia NOS	0
BBr02	[M]Subacute leukaemia NOS	0
BBr03	[M]Chronic leukaemia NOS	0
BBr04	[M]Aleukaemic leukaemia NOS	0
BBr0z	[M]Leukaemia unspecified, NOS	0
BBr1.	[M]Compound leukaemias	0
BBr10	[M]Compound leukaemia	0
BBr1z	[M]Compound leukaemia NOS	0
BBr2.	[M]Lymphoid leukaemias	0
BBr20	[M]Lymphoid leukaemia NOS	0
BBr21	[M]Acute lymphoid leukaemia	0
BBr22	[M]Subacute lymphoid leukaemia	0
BBr23	[M]Chronic lymphoid leukaemia	0
BBr24	[M]Aleukaemic lymphoid leukaemia	0
BBr25	[M]Prolymphocytic leukaemia	0
BBr26	[M]Burkitt's cell leukaemia	0
BBr2z	[M]Other lymphoid leukaemia NOS	0
BBr4.	[M]Erythroleukaemias	0
BBr40	[M]Erythroleukaemia	0
BBr4z	[M]Erythroleukaemia NOS	0
BBr5.	[M]Lymphosarcoma cell leukaemias	0
BBr50	[M]Lymphosarcoma cell leukaemia	0
BBr5z	[M]Lymphosarcoma cell leukaemia NOS	0
BBr6.	[M]Myeloid leukaemias	0
BBr60	[M]Myeloid leukaemia NOS	0
BBr61	[M]Acute myeloid leukaemia	0
BBr62	[M]Subacute myeloid leukaemia	0
BBr63	[M]Chronic myeloid leukaemia	0
BBr64	[M]Aleukaemic myeloid leukaemia	0
BBr65	[M]Neutrophilic leukaemia	0
BBr66	[M]Acute promyelocytic leukaemia	0
BBr67	[M]Acute myelomonocytic leukaemia	0
BBr68	[M]Chronic myelomonocytic leukaemia	0
BBr69	[M]Juvenile myelomonocytic leukaemia	0
BBr6z	[M]Other myeloid leukaemia NOS	0
BBr7.	[M]Basophilic leukaemias	0

BBr70	[M]Basophilic leukaemia	0
BBr7z	[M]Basophilic leukaemia NOS	0
BBr8.	[M]Eosinophilic leukaemias	0
BBr80	[M]Eosinophilic leukaemia	0
BBr8z	[M]Eosinophilic leukaemia NOS	0
BBr9.	[M]Monocytic leukaemias	0
BBr90	[M]Monocytic leukaemia NOS	0
BBr91	[M]Acute monocytic leukaemia	0
BBr92	[M]Subacute monocytic leukaemia	0
BBr93	[M]Chronic monocytic leukaemia	0
BBr94	[M]Aleukaemic monocytic leukaemia	0
BBr9z	[M]Other monocytic leukaemia NOS	0
BBrA.	[M]Miscellaneous leukaemias	0
BBrA0	[M]Mast cell leukaemia	0
BBrA1	[M]Megakaryocytic leukaemia	0
BBrA3	[M]Myeloid sarcoma	0
BBrA4	[M]Hairy cell leukaemia	0
BBrA5	[M]Acute megakaryoblastic leukaemia	0
BBrA6	[M]Acute panmyelosis	0
BBrA7	[M]Acute myelofibrosis	0
BBrAz	[M]Miscellaneous leukaemia NOS	0
BBrz.	[M]Leukaemia NOS	0
BBs1.	[M]Acute panmyelosis	0
BBs3.	[M]Myelosclerosis with myeloid metaplasia	0
ByuD.	[X]Malignant neoplasms of lymphoid, haematopoietic and related tissue	0
ByuD5	[X]Other lymphoid leukaemia	0
ByuD6	[X]Other myeloid leukaemia	0
ByuD7	[X]Other monocytic leukaemia	0
ByuD8	[X]Other specified leukaemias	0
ByuD9	[X]Other leukaemia of unspecified cell type	0
D41y1	Myelofibrosis	0
ZV106	[V]Personal history of leukaemia	0
ZV678	[V]Follow-up examination after chemotherapy for malignant neoplasm	0

CONCEPT: Lymphoma

READ_CODE	DESCRIPTION_SAIL	SENSITIVE_CODE
4M20.	Lymphoma stage I	0
4M21.	Lymphoma stage II	0
4M22.	Lymphoma stage III	0
4M23.	Lymphoma stage IV	0
A7896	HIV disease resulting in Burkitt's lymphoma	1
A7897	HIV disease resulting in other types of non-Hodgkin's lymphoma	1
AyuC6	[X]HIV disease resulting in other non-Hodgkin's lymphoma	1
B5601	Secondary and unspecified malignant neoplasm of mastoid lymph nodes	0
B6...	Malignant neoplasm of lymphatic and haemopoietic tissue	0
B60..	Lymphosarcoma and reticulosarcoma	0
B600.	Reticulosarcoma	0
B6000	Reticulosarcoma of unspecified site	0
B6001	Reticulosarcoma of lymph nodes of head, face and neck	0
B6002	Reticulosarcoma of intrathoracic lymph nodes	0
B6003	Reticulosarcoma of intra-abdominal lymph nodes	0
B6004	Reticulosarcoma of lymph nodes of axilla and upper limb	0
B6005	Reticulosarcoma of lymph nodes of inguinal region and lower limb	0
B6006	Reticulosarcoma of intrapelvic lymph nodes	0
B6007	Reticulosarcoma of spleen	0
B6008	Reticulosarcoma of lymph nodes of multiple sites	0
B600z	Reticulosarcoma NOS	0
B601.	Lymphosarcoma	0
B6010	Lymphosarcoma of unspecified site	0
B6011	Lymphosarcoma of lymph nodes of head, face and neck	0
B6012	Lymphosarcoma of intrathoracic lymph nodes	0
B6013	Lymphosarcoma of intra-abdominal lymph nodes	0
B6014	Lymphosarcoma of lymph nodes of axilla and upper limb	0
B6015	Lymphosarcoma of lymph nodes of inguinal region and lower limb	0
B6016	Lymphosarcoma of intrapelvic lymph nodes	0
B6017	Lymphosarcoma of spleen	0
B6018	Lymphosarcoma of lymph nodes of multiple sites	0
B601z	Lymphosarcoma NOS	0
B602.	Burkitt's lymphoma	0
B6020	Burkitt's lymphoma of unspecified site	0
B6021	Burkitt's lymphoma of lymph nodes of head, face and neck	0
B6022	Burkitt's lymphoma of intrathoracic lymph nodes	0
B6023	Burkitt's lymphoma of intra-abdominal lymph nodes	0
B6024	Burkitt's lymphoma of lymph nodes of axilla and upper limb	0
B6025	Burkitt's lymphoma of lymph nodes of inguinal region and lower limb	0
B6026	Burkitt's lymphoma of intrapelvic lymph nodes	0
B6027	Burkitt's lymphoma of spleen	0
B6028	Burkitt's lymphoma of lymph nodes of multiple sites	0
B602z	Burkitt's lymphoma NOS	0
B60y.	Other specified reticulosarcoma or lymphosarcoma	0
B60z.	Reticulosarcoma or lymphosarcoma NOS	0
B61..	Hodgkin's disease	0
B610.	Hodgkin's paragranuloma	0
B6100	Hodgkin's paragranuloma of unspecified site	0
B6101	Hodgkin's paragranuloma of lymph nodes of head, face, and neck	0
B6102	Hodgkin's paragranuloma of intrathoracic lymph nodes	0
B6103	Hodgkin's paragranuloma of intra-abdominal lymph nodes	0
B6104	Hodgkin's paragranuloma of lymph nodes of axilla and upper limb	0
B6105	Hodgkin's paragranuloma of lymph nodes of inguinal region and lower limb	0
B6106	Hodgkin's paragranuloma of intrapelvic lymph nodes	0
B6107	Hodgkin's paragranuloma of spleen	0
B6108	Hodgkin's paragranuloma of lymph nodes of multiple sites	0
B610z	Hodgkin's paragranuloma NOS	0
B611.	Hodgkin's granuloma	0
B6110	Hodgkin's granuloma of unspecified site	0
B6111	Hodgkin's granuloma of lymph nodes of head, face and neck	0
B6112	Hodgkin's granuloma of intrathoracic lymph nodes	0
B6113	Hodgkin's granuloma of intra-abdominal lymph nodes	0
B6114	Hodgkin's granuloma of lymph nodes of axilla and upper limb	0
B6115	Hodgkin's granuloma of lymph nodes of inguinal region and lower limb	0
B6116	Hodgkin's granuloma of intrapelvic lymph nodes	0
B6117	Hodgkin's granuloma of spleen	0

B6118	Hodgkin's granuloma of lymph nodes of multiple sites	0
B611z	Hodgkin's granuloma NOS	0
B612.	Hodgkin's sarcoma	0
B6120	Hodgkin's sarcoma of unspecified site	0
B6121	Hodgkin's sarcoma of lymph nodes of head, face and neck	0
B6122	Hodgkin's sarcoma of intrathoracic lymph nodes	0
B6123	Hodgkin's sarcoma of intra-abdominal lymph nodes	0
B6124	Hodgkin's sarcoma of lymph nodes of axilla and upper limb	0
B6125	Hodgkin's sarcoma of lymph nodes of inguinal region and lower limb	0
B6126	Hodgkin's sarcoma of intrapelvic lymph nodes	0
B6127	Hodgkin's sarcoma of spleen	0
B6128	Hodgkin's sarcoma of lymph nodes of multiple sites	0
B612z	Hodgkin's sarcoma NOS	0
B613.	Hodgkin's disease, lymphocytic-histiocytic predominance	0
B6130	Hodgkin's disease, lymphocytic-histiocytic predominance of unspecified site	0
B6131	Hodgkin's disease, lymphocytic-histiocytic predominance of lymph nodes of head, face and neck	0
B6132	Hodgkin's disease, lymphocytic-histiocytic predominance of intrathoracic lymph nodes	0
B6133	Hodgkin's disease, lymphocytic-histiocytic predominance of intra-abdominal lymph nodes	0
B6134	Hodgkin's disease, lymphocytic-histiocytic predominance of lymph nodes of axilla and upper limb	0
B6135	Hodgkin's disease, lymphocytic-histiocytic predominance of lymph nodes of inguinal region and lower limb	0
B6136	Hodgkin's disease, lymphocytic-histiocytic predominance of intrapelvic lymph nodes	0
B6137	Hodgkin's, lymphocytic-histiocytic predominance of spleen	0
B6138	Hodgkin's disease, lymphocytic-histiocytic predominance of lymph nodes of multiple sites	0
B613z	Hodgkin's, lymphocytic-histiocytic predominance NOS	0
B614.	Hodgkin's disease, nodular sclerosis	0
B6140	Hodgkin's disease, nodular sclerosis of unspecified site	0
B6141	Hodgkin's disease, nodular sclerosis of lymph nodes of head, face and neck	0
B6142	Hodgkin's disease, nodular sclerosis of intrathoracic lymph nodes	0
B6143	Hodgkin's disease, nodular sclerosis of intra-abdominal lymph nodes	0
B6144	Hodgkin's disease, nodular sclerosis of lymph nodes of axilla and upper limb	0
B6145	Hodgkin's disease, nodular sclerosis of lymph nodes of inguinal region and lower limb	0
B6146	Hodgkin's disease, nodular sclerosis of intrapelvic lymph nodes	0
B6147	Hodgkin's disease, nodular sclerosis of spleen	0
B6148	Hodgkin's disease, nodular sclerosis of lymph nodes of multiple sites	0
B614z	Hodgkin's disease, nodular sclerosis NOS	0
B615.	Hodgkin's disease, mixed cellularity	0
B6150	Hodgkin's disease, mixed cellularity of unspecified site	0
B6151	Hodgkin's disease, mixed cellularity of lymph nodes of head, face and neck	0
B6152	Hodgkin's disease, mixed cellularity of intrathoracic lymph nodes	0
B6153	Hodgkin's disease, mixed cellularity of intra-abdominal lymph nodes	0
B6154	Hodgkin's disease, mixed cellularity of lymph nodes of axilla and upper limb	0
B6155	Hodgkin's disease, mixed cellularity of lymph nodes of inguinal region and lower limb	0
B6156	Hodgkin's disease, mixed cellularity of intrapelvic lymph nodes	0
B6157	Hodgkin's disease, mixed cellularity of spleen	0
B6158	Hodgkin's disease, mixed cellularity of lymph nodes of multiple sites	0
B615z	Hodgkin's disease, mixed cellularity NOS	0
B616.	Hodgkin's disease, lymphocytic depletion	0
B6160	Hodgkin's lymphocytic depletion of unspecified site	0
B6161	Hodgkin's disease, lymphocytic depletion of lymph nodes of head, face and neck	0
B6162	Hodgkin's disease, lymphocytic depletion of intrathoracic lymph nodes	0
B6163	Hodgkin's disease, lymphocytic depletion of intra-abdominal lymph nodes	0
B6164	Hodgkin's disease, lymphocytic depletion of lymph nodes of axilla and upper limb	0
B6165	Hodgkin's disease, lymphocytic depletion of lymph nodes of inguinal region and lower limb	0
B6166	Hodgkin's disease, lymphocytic depletion of intrapelvic lymph nodes	0
B6167	Hodgkin's disease, lymphocytic depletion of spleen	0
B6168	Hodgkin's disease, lymphocytic depletion of lymph nodes of multiple sites	0
B616z	Hodgkin's disease, lymphocytic depletion NOS	0
B617.	Nodular lymphocyte predominant Hodgkin lymphoma	0
B618.	Nodular sclerosis classical Hodgkin lymphoma	0
B619.	Mixed cellularity classical Hodgkin lymphoma	0
B61A.	Lymphocyte depleted classical Hodgkin lymphoma	0
B61B.	Lymphocyte-rich classical Hodgkin lymphoma	0
B61C.	Other classical Hodgkin lymphoma	0
B61z.	Hodgkin's disease NOS	0
B61z0	Hodgkin's disease NOS, unspecified site	0
B61z1	Hodgkin's disease NOS of lymph nodes of head, face and neck	0
B61z2	Hodgkin's disease NOS of intrathoracic lymph nodes	0
B61z3	Hodgkin's disease NOS of intra-abdominal lymph nodes	0
B61z4	Hodgkin's disease NOS of lymph nodes of axilla and upper limb	0
B61z5	Hodgkin's disease NOS of lymph nodes of inguinal region and lower limb	0
B61z6	Hodgkin's disease NOS of intrapelvic lymph nodes	0
B61z7	Hodgkin's disease NOS of spleen	0
B61z8	Hodgkin's disease NOS of lymph nodes of multiple sites	0
B61zz	Hodgkin's disease NOS	0
B62..	Other malignant neoplasm of lymphoid and histiocytic tissue	0
B620.	Nodular lymphoma (Brill - Symmers disease)	0
B6200	Nodular lymphoma of unspecified site	0
B6201	Nodular lymphoma of lymph nodes of head, face and neck	0
B6202	Nodular lymphoma of intrathoracic lymph nodes	0
B6203	Nodular lymphoma of intra-abdominal lymph nodes	0
B6204	Nodular lymphoma of lymph nodes of axilla and upper limb	0
B6205	Nodular lymphoma of lymph nodes of inguinal region and lower limb	0
B6206	Nodular lymphoma of intrapelvic lymph nodes	0
B6207	Nodular lymphoma of spleen	0
B6208	Nodular lymphoma of lymph nodes of multiple sites	0
B620z	Nodular lymphoma NOS	0
B621.	Mycosis fungoides	0
B6210	Mycosis fungoides of unspecified site	0
B6211	Mycosis fungoides of the lymph nodes of head, face and neck	0
B6212	Mycosis fungoides of intrathoracic lymph nodes	0
B6213	Mycosis fungoides of intra-abdominal lymph nodes	0
B6214	Mycosis fungoides of lymph nodes of axilla and upper limb	0
B6215	Mycosis fungoides of lymph nodes of inguinal region and lower limb	0
B6216	Mycosis fungoides of intrapelvic lymph nodes	0
B6217	Mycosis fungoides of spleen	0
B6218	Mycosis fungoides of lymph nodes of multiple sites	0
B621z	Mycosis fungoides NOS	0
B622.	Sezary's disease	0
B6220	Sezary's disease of unspecified site	0
B6221	Sezary's disease of lymph nodes of head, face and neck	0
B6222	Sezary's disease of intrathoracic lymph nodes	0
B6223	Sezary's disease of intra-abdominal lymph nodes	0
B6224	Sezary's disease of lymph nodes of axilla and upper limb	0

B6225	Sezary's disease of lymph nodes of inguinal region and lower limb	0
B6226	Sezary's disease of intrapelvic lymph nodes	0
B6227	Sezary's disease of spleen	0
B6228	Sezary's disease of lymph nodes of multiple sites	0
B622z	Sezary's disease NOS	0
B623.	Malignant histiocytosis	0
B6230	Malignant histiocytosis of unspecified site	0
B6231	Malignant histiocytosis of lymph nodes of head, face and neck	0
B6232	Malignant histiocytosis of intrathoracic lymph nodes	0
B6233	Malignant histiocytosis of intra-abdominal lymph nodes	0
B6234	Malignant histiocytosis of lymph nodes of axilla and upper limb	0
B6235	Malignant histiocytosis of lymph nodes of inguinal region and lower limb	0
B6236	Malignant histiocytosis of intrapelvic lymph nodes	0
B6237	Malignant histiocytosis of spleen	0
B6238	Malignant histiocytosis of lymph nodes of multiple sites	0
B623z	Malignant histiocytosis NOS	0
B624.	Leukaemic reticuloendotheliosis	0
B6240	Leukaemic reticuloendotheliosis of unspecified sites	0
B6241	Leukaemic reticuloendotheliosis of lymph nodes of head, face and neck	0
B6242	Leukaemic reticuloendotheliosis of intrathoracic lymph nodes	0
B6243	Leukaemic reticuloendotheliosis of intra-abdominal lymph nodes	0
B6244	Leukaemic reticuloendotheliosis of lymph nodes of axilla and upper limb	0
B6245	Leukaemic reticuloendotheliosis of lymph nodes of inguinal region and lower limb	0
B6246	Leukaemic reticuloendotheliosis of intrapelvic lymph nodes	0
B6247	Leukaemic reticuloendotheliosis of spleen	0
B6248	Leukaemic reticuloendotheliosis of lymph nodes of multiple sites	0
B624z	Leukaemic reticuloendotheliosis NOS	0
B625.	Letterer-Siwe disease	0
B6250	Letterer-Siwe disease of unspecified sites	0
B6251	Letterer-Siwe disease of lymph nodes of head, face and neck	0
B6252	Letterer-Siwe disease of intrathoracic lymph nodes	0
B6253	Letterer-Siwe disease of intra-abdominal lymph nodes	0
B6254	Letterer-Siwe disease of lymph nodes of axilla and upper limb	0
B6255	Letterer-Siwe disease of lymph nodes of inguinal region and lower limb	0
B6256	Letterer-Siwe disease of intrapelvic lymph nodes	0
B6257	Letterer-Siwe disease of spleen	0
B6258	Letterer-Siwe disease of lymph nodes of multiple sites	0
B625z	Letterer-Siwe disease NOS	0
B626.	Malignant mast cell tumours	0
B6260	Mast cell malignancy of unspecified site	0
B6261	Mast cell malignancy of lymph nodes of head, face and neck	0
B6262	Mast cell malignancy of intrathoracic lymph nodes	0
B6263	Mast cell malignancy of intra-abdominal lymph nodes	0
B6264	Mast cell malignancy of lymph nodes of axilla and upper limb	0
B6265	Mast cell malignancy of lymph nodes of inguinal region and lower limb	0
B6266	Mast cell malignancy of intrapelvic lymph nodes	0
B6267	Mast cell malignancy of spleen	0
B6268	Mast cell malignancy of lymph nodes of multiple sites	0
B626z	Malignant mast cell tumour NOS	0
B627.	Non - Hodgkin's lymphoma	0
B6270	Follicular non-Hodgkin's small cleaved cell lymphoma	0
B6271	Follicular non-Hodgkin's mixed small cleaved and large cell lymphoma	0
B6272	Follicular non-Hodgkin's large cell lymphoma	0
B6273	Diffuse non-Hodgkin's small cell (diffuse) lymphoma	0
B6274	Diffuse non-Hodgkin's small cleaved cell (diffuse) lymphoma	0
B6275	Diffuse non-Hodgkin's mixed small and large cell (diffuse) lymphoma	0
B6276	Diffuse non-Hodgkin's immunoblastic (diffuse) lymphoma	0
B6277	Diffuse non-Hodgkin's lymphoblastic (diffuse) lymphoma	0
B6278	Diffuse non-Hodgkin's lymphoma undifferentiated (diffuse)	0
B6279	Mucosa-associated lymphoma	0
B627A	Diffuse non-Hodgkin's large cell lymphoma	0
B627B	Other types of follicular non-Hodgkin's lymphoma	0
B627C	Follicular non-Hodgkin's lymphoma	0
B627D	Diffuse non-Hodgkin's centroblastic lymphoma	0
B627E	Diffuse large B-cell lymphoma	0
B627F	Extranodal marginal zone B-cell lymphoma of mucosa-associated lymphoid tissue	0
B627G	Mediastinal (thymic) large B-cell lymphoma	0
B627W	Unspecified B-cell non-Hodgkin's lymphoma	0
B627X	Diffuse non-Hodgkin's lymphoma, unspecified	0
B628.	Follicular lymphoma	0
B6280	Follicular lymphoma grade 1	0
B6281	Follicular lymphoma grade 2	0
B6282	Follicular lymphoma grade 3	0
B6283	Follicular lymphoma grade 3a	0
B6284	Follicular lymphoma grade 3b	0
B6285	Diffuse follicle centre lymphoma	0
B6286	Cutaneous follicle centre lymphoma	0
B6287	Other types of follicular lymphoma	0
B629.	Multifocal and multisystemic (disseminated) Langerhans-cell histiocytosis	0
B62A.	Sarcoma of dendritic cells	0
B62B.	Multifocal and unisystemic Langerhans-cell histiocytosis	0
B62C.	Unifocal Langerhans-cell histiocytosis	0
B62D.	Histiocytic sarcoma	0
B62E.	T/NK-cell lymphoma	0
B62E0	Mature T/NK-cell lymphoma	0
B62E1	Anaplastic large cell lymphoma, ALK-positive	0
B62E2	Anaplastic large cell lymphoma, ALK-negative	0
B62E3	Cutaneous T-cell lymphoma	0
B62E4	Extranodal NK/T-cell lymphoma, nasal type	0
B62E5	Hepatosplenic T-cell lymphoma	0
B62E6	Enteropathy-associated T-cell lymphoma	0
B62E7	Subcutaneous panniculitic T-cell lymphoma	0
B62E8	Blastic NK-cell lymphoma	0
B62E9	Angioimmunoblastic T-cell lymphoma	0
B62EA	Primary cutaneous CD30-positive T-cell proliferations	0
B62Ew	Other mature T/NK-cell lymphoma	0
B62F.	Nonfollicular lymphoma	0
B62F0	Small cell B-cell lymphoma	0
B62F1	Mantle cell lymphoma	0
B62F2	Lymphoblastic (diffuse) lymphoma	0
B62Fy	Other non-follicular lymphoma	0
B62x.	Malignant lymphoma otherwise specified	0
B62x0	T-zone lymphoma	0

B62x1	Lymphoepithelioid lymphoma	0
B62x2	Peripheral T-cell lymphoma	0
B62x3	Malignant reticuloendotheliosis	0
B62x4	Malignant reticulosis	0
B62x5	Malignant immunoproliferative small intestinal disease	0
B62x6	True histiocytic lymphoma	0
B62xX	Other and unspecified peripheral and cutaneous T-cell lymphomas	0
B62y.	Malignant lymphoma NOS	0
B62y0	Malignant lymphoma NOS of unspecified site	0
B62y1	Malignant lymphoma NOS of lymph nodes of head, face and neck	0
B62y2	Malignant lymphoma NOS of intrathoracic lymph nodes	0
B62y3	Malignant lymphoma NOS of intra-abdominal lymph nodes	0
B62y4	Malignant lymphoma NOS of lymph nodes of axilla and upper limb	0
B62y5	Malignant lymphoma NOS of lymph nodes of inguinal region and lower limb	0
B62y6	Malignant lymphoma NOS of intrapelvic lymph nodes	0
B62y7	Malignant lymphoma NOS of spleen	0
B62y8	Malignant lymphoma NOS of lymph nodes of multiple sites	0
B62yz	Malignant lymphoma NOS	0
B62z.	Malignant neoplasms of lymphoid and histiocytic tissue NOS	0
B62z0	Unspecified malignant neoplasm of lymphoid and histiocytic tissue of unspecified site	0
B62z1	Unspecified malignant neoplasm of lymphoid and histiocytic tissue of lymph nodes of head, face and neck	0
B62z2	Unspecified malignant neoplasm of lymphoid and histiocytic tissue of intrathoracic lymph nodes	0
B62z3	Unspecified malignant neoplasm of lymphoid and histiocytic tissue of intra-abdominal lymph nodes	0
B62z4	Unspecified malignant neoplasm of lymphoid and histiocytic tissue of lymph nodes of axilla and upper limb	0
B62z5	Unspecified malignant neoplasm of lymphoid and histiocytic tissue of lymph nodes of inguinal region and lower limb	0
B62z6	Unspecified malignant neoplasm of lymphoid and histiocytic tissue of intrapelvic lymph nodes	0
B62z7	Unspecified malignant neoplasm of lymphoid and histiocytic tissue of spleen	0
B62z8	Unspecified malignant neoplasm of lymphoid and histiocytic tissue of lymph nodes of multiple sites	0
B62zz	Lymphoid and histiocytic malignancy NOS	0
B63y.	Other immunoproliferative neoplasms	0
B64y5	Adult T-cell lymphoma/leukaemia (HTLV-1-associated)	0
B67y0	Lymphosarcoma cell leukaemia	0
B6y..	Malignant neoplasm of lymphatic or haematopoietic tissue otherwise specified	0
B6z..	Malignant neoplasm lymphatic or haematopoietic tissue NOS	0
BBGF.	[M]Fibrous histiocytoma, malignant	0
BBg..	[M]Lymphomas, NOS or diffuse	0
BBg1.	[M]Malignant lymphoma NOS	0
BBg10	[M]Malignant lymphoma, diffuse NOS	0
BBg2.	[M]Malignant lymphoma, non Hodgkin's type	0
BBg3.	[M]Malignant lymphoma, undifferentiated cell type NOS	0
BBg4.	[M]Malignant lymphoma, stem cell type	0
BBg5.	[M]Malignant lymphoma, convoluted cell type NOS	0
BBg6.	[M]Lymphosarcoma NOS	0
BBg7.	[M]Malignant lymphoma, lymphoplasmacytoid type	0
BBg8.	[M]Malignant lymphoma, immunoblastic type	0
BBg9.	[M]Malignant lymphoma, mixed lymphocytic-histiocytic NOS	0
BBgA.	[M]Malignant lymphoma, centroblastic-centrocytic, diffuse	0
BBgB.	[M]Malignant lymphoma, follicular centre cell NOS	0
BBgC.	[M]Malignant lymphoma, lymphocytic, well differentiated NOS	0
BBgD.	[M]Malignant lymphoma, lymphocytic, intermediate differentiation NOS	0
BBgE.	[M]Malignant lymphoma, centrocytic	0
BBgF.	[M]Malignant lymphoma, follicular centre cell, cleaved NOS	0
BBgG.	[M]Malignant lymphoma, lymphocytic, poorly differentiated NOS	0
BBgH.	[M]Prolymphocytic lymphosarcoma	0
BBgJ.	[M]Malignant lymphoma, centroblastic type NOS	0
BBgK.	[M]Malignant lymphoma, follicular centre cell, non-cleaved NOS	0
BBgL.	[M]Malignant lymphoma, small lymphocytic NOS	0
BBgM.	[M]Malignant lymphoma, small cleaved cell, diffuse	0
BBgN.	[M]Malignant lymphoma, lymphocytic, intermediate differentiation, diffuse	0
BBgP.	[M]Malignant lymphoma, mixed small and large cell, diffuse	0
BBgQ.	[M]Malignant lymphomatous polyposis	0
BBgR.	[M]Malignant lymphoma, large cell, diffuse NOS	0
BBgS.	[M]Malignant lymphoma, large cell, cleaved, diffuse	0
BBgT.	[M]Malignant lymphoma, large cell, noncleaved, diffuse	0
BBgV.	[M]Malignant lymphoma, small cell, noncleaved, diffuse	0
BBgz.	[M]Lymphoma, diffuse or NOS	0
BBh..	[M]Reticulosarcomas	0
BBh0.	[M]Reticulosarcoma NOS	0
BBh1.	[M]Reticulosarcoma, pleomorphic cell type	0
BBh2.	[M]Reticulosarcoma, nodular	0
BBhz.	[M]Reticulosarcoma NOS	0
BBj..	[M]Hodgkin's disease	0
BBj0.	[M]Hodgkin's disease NOS	0
BBj1.	[M]Hodgkin's disease, lymphocytic predominance	0
BBj10	[M]Hodgkin,s disease, lymphocytic predominance, diffuse	0
BBj11	[M]Hodgkin,s disease, lymphocytic predominance, nodular	0
BBj2.	[M]Hodgkin's disease, mixed cellularity	0
BBj3.	[M]Hodgkin's disease, lymphocytic depletion NOS	0
BBj4.	[M]Hodgkin's disease, lymphocytic depletion, diffuse fibrosis	0
BBj5.	[M]Hodgkin's disease, lymphocytic depletion, reticular type	0
BBj6.	[M]Hodgkin's disease, nodular sclerosis NOS	0
BBj60	[M]Hodgkin,s disease, nodular sclerosis, lymphocytic predominance	0
BBj61	[M]Hodgkin,s disease, nodular sclerosis, mixed cellularity	0
BBj62	[M]Hodgkin,s disease, nodular sclerosis, lymphocytic depletion	0
BBj7.	[M]Hodgkin's disease, nodular sclerosis, cellular phase	0
BBj8.	[M]Hodgkin's paragranuloma	0
BBj9.	[M]Hodgkin's granuloma	0
BBjA.	[M]Hodgkin's sarcoma	0
BBjz.	[M]Hodgkin's disease NOS	0
BBk..	[M]Lymphomas, nodular or follicular	0
BBk0.	[M]Malignant lymphoma, nodular NOS	0
BBk1.	[M]Malignant lymphoma, mixed lymphocytic-histiocytic, nodular	0
BBk2.	[M]Malignant lymphoma, centroblastic-centrocytic, follicular	0
BBk3.	[M]Malignant lymphoma, lymphocytic, well differentiated, nodular	0
BBk4.	[M]Malignant lymphoma, lymphocytic, intermediate differentiation, nodular	0
BBk5.	[M]Malignant lymphoma, follicular centre cell, cleaved, follicular	0
BBk6.	[M]Malignant lymphoma, lymphocytic, poorly differentiated, nodular	0
BBk7.	[M]Malignant lymphoma, centroblastic type, follicular	0
BBkz.	[M]Lymphoma, nodular or follicular NOS	0
BBl..	[M]Mycosis fungoides	0
BBl0.	[M]Mycosis fungoides	0
BBl1.	[M]Sezary's disease	0
BBlz.	[M]Mycosis fungoides NOS	0

BBm1.	[M]Malignant histiocytosis	0
BBm3.	[M]Letterer - Siwe disease	0
BBm4.	[M]True histiocytic lymphoma	0
BBm5.	[M] Peripheral T-cell lymphoma NOS	0
BBm9.	[M] Monocytoid B-cell lymphoma	0
BBmD.	[M] Cutaneous lymphoma	0
BBmG.	[M] Immunoproliferative small intestinal disease	0
BBmH.	[M] Large cell lymphoma	0
BBmK.	[M]Waldenstrom's macroglobulinaemia	0
BBp1.	[M]Mast cell sarcoma	0
BBp2.	[M]Malignant mastocytosis	0
BBq..	[M]Burkitt's tumours	0
BBq0.	[M]Burkitt's tumour	0
BBqz.	[M]Burkitt's tumour NOS	0
BBr27	[M]Adult T-cell leukaemia/lymphoma	0
BBr5.	[M]Lymphosarcoma cell leukaemias	0
BBr50	[M]Lymphosarcoma cell leukaemia	0
BBr5z	[M]Lymphosarcoma cell leukaemia NOS	0
BBv0.	[M]Monocytoid B-cell lymphoma	0
BBv2.	[M]AngiocentricT-cell lymphoma	0
ByuD.	[X]Malignant neoplasms of lymphoid, haematopoietic and related tissue	0
ByuD0	[X]Other Hodgkin's disease	0
ByuD1	[X]Other types of follicular non-Hodgkin's lymphoma	0
ByuD2	[X]Other types of diffuse non-Hodgkin's lymphoma	0
ByuD3	[X]Other specified types of non-Hodgkin's lymphoma	0
ByuD4	[X]Other malignant immunoproliferative diseases	0
ByuDA	[X]Other specified malignant neoplasms of lymphoid, haematopoietic and related tissue	0
ByuDB	[X]Malignant neoplasm of lymphoid, haematopoietic and related tissue, unspecified	0
ByuDC	[X]Diffuse non-Hodgkin's lymphoma, unspecified	0
ByuDD	[X]Other and unspecified peripheral and cutaneous T-cell lymphomas	0
ByuDE	[X]Unspecified B-cell non-Hodgkin's lymphoma	0
ByuDF	[X]Non-Hodgkin's lymphoma, unspecified type	0
C333.	Macroglobulinaemia	0
C3330	Waldenstrom's macroglobulinaemia	0
C333z	Macroglobulinaemia NOS	0
C37y0	Hand - Schuller - Christian disease	0
C37y5	Histiocytosis X , chronic	0
C37y6	Histiocytosis X , unspecified	0
C37yB	Langerhans' cell histiocytosis	0
C37yz	Other specified disorder of metabolism NOS	0
Dyu44	[X]Other histiocytosis syndromes	0
G753.	Lethal midline granuloma	0
M1628	Lymphomatoid papulosis	0
ZV107	[V]Personal history of other lymphatic or haematopoietic neoplasm	0
ZVu6G	[X]Personal history of other malignant neoplasms of lymphoid, haematopoietic and related tissues	0

CONCEPT: Marfan Syndrome

READ_CODE	DESCRIPTION_SAIL	SENSITIVE_CODE
PKy2.	Marfan's syndrome	0

CONCEPT: Meniere's disease

READ_CODE	DESCRIPTION_SAIL	SENSITIVE_CODE
1491.	H/O: vertigo/Meniere's disease	0
F560.	Meniere's disease	0
F5600	Unspecified Meniere's disease	0
F5601	Active cochleovestibular Meniere's disease	0
F5602	Active cochlear Meniere's disease	0
F5603	Active vestibular Meniere's disease	0
F560z	Meniere's disease NOS	0

CONCEPT: Metastatic cancers

READ_CODE	DESCRIPTION_SAIL	SENSITIVE_CODE
1D18.	Pain from metastases	0
5136.	X-ray metastasis control	0
5A12.	Thyroid tumour/metast irradiat	0
5A15.	Bone tumour/metast.irradiat.	0
8Hgg.	Discharge from secondary care cancer service	0
A7886	Human immunodeficiency virus with secondary cancers	1
B153.	Secondary malignant neoplasm of liver	0
B56..	Secondary and unspecified malignant neoplasm of lymph nodes	0
B560.	Secondary and unspecified malignant neoplasm of lymph nodes of head, face and neck	0
B5600	Secondary and unspecified malignant neoplasm of superficial parotid lymph nodes	0
B5601	Secondary and unspecified malignant neoplasm of mastoid lymph nodes	0
B5602	Secondary and unspecified malignant neoplasm of superficial cervical lymph nodes	0
B5603	Secondary and unspecified malignant neoplasm of occipital lymph nodes	0
B5604	Secondary and unspecified malignant neoplasm of deep parotid lymph nodes	0
B5605	Secondary and unspecified malignant neoplasm of submandibular lymph nodes	0
B5606	Secondary and unspecified malignant neoplasm of facial lymph nodes	0
B5607	Secondary and unspecified malignant neoplasm of submental lymph nodes	0
B5608	Secondary and unspecified malignant neoplasm of anterior cervical lymph nodes	0
B5609	Secondary and unspecified malignant neoplasm of deep cervical lymph nodes	0
B560z	Secondary and unspecified malignant neoplasm of lymph nodes of head, face and neck NOS	0
B561.	Secondary and unspecified malignant neoplasm of intrathoracic lymph nodes	0
B5610	Secondary and unspecified malignant neoplasm of internal mammary lymph nodes	0
B5611	Secondary and unspecified malignant neoplasm of intercostal lymph nodes	0
B5612	Secondary and unspecified malignant neoplasm of diaphragmatic lymph nodes	0
B5613	Secondary and unspecified malignant neoplasm of anterior mediastinal lymph nodes	0
B5614	Secondary and unspecified malignant neoplasm of posterior mediastinal lymph nodes	0
B5615	Secondary and unspecified malignant neoplasm of paratracheal lymph nodes	0
B5616	Secondary and unspecified malignant neoplasm of superficial tracheobronchial lymph nodes	0
B5617	Secondary and unspecified malignant neoplasm of inferior tracheobronchial lymph nodes	0
B5618	Secondary and unspecified malignant neoplasm of bronchopulmonary lymph nodes	0
B5619	Secondary and unspec malig neop pulmonary lymph nodes	0
B561z	Secondary and unspecified malignant neoplasm of intrathoracic lymph nodes NOS	0
B562.	Secondary and unspecified malignant neoplasm of intra-abdominal lymph nodes	0
B5620	Secondary and unspecified malignant neoplasm of coeliac lymph nodes	0
B5621	Secondary and unspecified malignant neoplasm of superficial mesenteric lymph nodes	0
B5622	Secondary and unspecified malignant neoplasm of inferior mesenteric lymph nodes	0
B5623	Secondary and unspecified malignant neoplasm of common iliac lymph nodes	0
B5624	Secondary and unspecified malignant neoplasm of external iliac lymph nodes	0
B562z	Secondary and unspecified malignant neoplasm of intra-abdominal lymph nodes NOS	0

B563.	Secondary and unspecified malignant neoplasm of axilla and upper limb lymph nodes	0
B5630	Secondary and unspecified malignant neoplasm of axillary lymph nodes	0
B5631	Secondary and unspecified malignant neoplasm of supratrochlear lymph nodes	0
B5632	Secondary and unspecified malignant neoplasm of infraclavicular lymph nodes	0
B5633	Secondary and unspecified malignant neoplasm of pectoral lymph nodes	0
B563z	Secondary and unspecified malignant neoplasm of axilla and upper limb lymph nodes NOS	0
B564.	Secondary and unspecified malignant neoplasm of inguinal and lower limb lymph nodes	0
B5640	Secondary and unspecified malignant neoplasm of superficial inguinal lymph nodes	0
B5641	Secondary and unspecified malignant neoplasm of deep inguinal lymph nodes	0
B5642	Secondary and unspecified malignant neoplasm of popliteal lymph nodes	0
B564z	Secondary and unspecified malignant neoplasm of inguinal and lower limb lymph nodes NOS	0
B565.	Secondary and unspecified malignant neoplasm of intrapelvic lymph nodes	0
B5650	Secondary and unspecified malignant neoplasm of internal iliac lymph nodes	0
B5651	Secondary and unspecified malignant neoplasm of inferior epigastric lymph nodes	0
B5652	Secondary and unspecified malignant neoplasm of circumflex iliac lymph nodes	0
B5653	Secondary and unspecified malignant neoplasm of sacral lymph nodes	0
B5654	Secondary and unspecified malignant neoplasm of obturator lymph nodes	0
B565z	Secondary and unspecified malignant neoplasm of intrapelvic lymph nodes NOS	0
B56y.	Secondary and unspecified malignant neoplasm of lymph nodes in multiple sites	0
B56z.	Secondary and unspecified malignant neoplasm of lymph nodes NOS	0
B57..	Secondary malignant neoplasm of respiratory and digestive systems	0
B570.	Secondary malignant neoplasm of lung	0
B571.	Secondary malignant neoplasm of mediastinum	0
B572.	Secondary malignant neoplasm of pleura	0
B573.	Secondary malignant neoplasm of other respiratory organs	0
B574.	Secondary malignant neoplasm of small intestine and duodenum	0
B5740	Secondary malignant neoplasm of duodenum	0
B5741	Secondary malignant neoplasm of jejunum	0
B5742	Secondary malignant neoplasm of ileum	0
B574z	Secondary malignant neoplasm of small intestine or duodenum NOS	0
B575.	Secondary malignant neoplasm of large intestine and rectum	0
B5750	Secondary malignant neoplasm of colon	0
B5751	Secondary malignant neoplasm of rectum	0
B575z	Secondary malignant neoplasm of large intestine or rectum NOS	0
B576.	Secondary malignant neoplasm of retroperitoneum and peritoneum	0
B5760	Secondary malignant neoplasm of retroperitoneum	0
B5761	Secondary malignant neoplasm of peritoneum	0
B576z	Secondary malignant neoplasm of retroperitoneum or peritoneum NOS	0
B577.	Secondary malignant neoplasm of liver	0
B5770	Secondary malignant neoplasm of liver and intrahepatic bile duct	0
B57y.	Secondary malignant neoplasm of other digestive organ	0
B57z.	Secondary malignant neoplasm of respiratory or digestive system NOS	0
B58..	Secondary malignant neoplasm of other specified sites	0
B580.	Secondary malignant neoplasm of kidney	0
B581.	Secondary malignant neoplasm of other urinary organs	0
B5810	Secondary malignant neoplasm of ureter	0
B5811	Secondary malignant neoplasm of bladder	0
B5812	Secondary malignant neoplasm of urethra	0
B581z	Secondary malignant neoplasm of other urinary organ NOS	0
B582.	Secondary malignant neoplasm of skin	0
B5820	Secondary malignant neoplasm of skin of head	0
B5821	Secondary malignant neoplasm of skin of face	0
B5822	Secondary malignant neoplasm of skin of neck	0
B5823	Secondary malignant neoplasm of skin of trunk	0
B5824	Secondary malignant neoplasm of skin of shoulder and arm	0
B5825	Secondary malignant neoplasm of skin of hip and leg	0
B5826	Secondary malignant neoplasm of skin of breast	0
B582z	Secondary malignant neoplasm of skin NOS	0
B583.	Secondary malignant neoplasm of brain and spinal cord	0
B5830	Secondary malignant neoplasm of brain	0
B5831	Secondary malignant neoplasm of spinal cord	0
B5832	Cerebral metastasis	0
B583z	Secondary malignant neoplasm of brain or spinal cord NOS	0
B584.	Secondary malignant neoplasm of other part of nervous system	0
B585.	Secondary malignant neoplasm of bone and bone marrow	0
B5850	Pathological fracture due to metastatic bone disease	0
B586.	Secondary malignant neoplasm of ovary	0
B587.	Secondary malignant neoplasm of adrenal gland	0
B58y.	Secondary malignant neoplasm of other specified sites	0
B58y0	Secondary malignant neoplasm of breast	0
B58y1	Secondary malignant neoplasm of uterus	0
B58y2	Secondary malignant neoplasm of cervix uteri	0
B58y3	Secondary malignant neoplasm of vagina	0
B58y4	Secondary malignant neoplasm of vulva	0
B58y5	Secondary malignant neoplasm of prostate	0
B58y6	Secondary malignant neoplasm of testis	0
B58y7	Secondary malignant neoplasm of penis	0
B58y8	Secondary malignant neoplasm of epididymis and vas deferens	0
B58y9	Secondary malignant neoplasm of tongue	0
B58yz	Secondary malignant neoplasm of other specified site NOS	0
B58z.	Secondary malignant neoplasm of other specified site NOS	0
B590.	Disseminated malignancy NOS	0
B594.	Secondary malignant neoplasm of unknown site	0
BB03.	[M]Neoplasm, metastatic	0
BB04.	[M]Neoplasm, malignant, uncertain whether primary or metastatic	0
BB13.	[M]Carcinoma, metastatic, NOS	0
BB14.	[M]Carcinomatosis	0
BB2B.	[M]Squamous cell carcinoma, metastatic NOS	0
BB53.	[M]Adenocarcinoma, metastatic, NOS	0
BB851	[M]Metastatic signet ring cell carcinoma	0
BBy2.	[M]No microscopic confirmation of tumour, clinically metastatic	0
ByuC.	[X]Malignant neoplasm of ill-defined, secondary and unspecified sites	0
ByuC2	[X]Secondary and unspecified malignant neoplasm lymph nodes of multiple regions	0
ByuC3	[X]Secondary malignant neoplasm of other and unspecified respiratory organs	0
ByuC4	[X]Secondary malignant neoplasm of other and unspecified digestive organs	0
ByuC5	[X]Secondary malignant neoplasm of bladder and other and unspecified urinary organs	0
ByuC6	[X]Secondary malignant neoplasm of other and unspecified parts of nervous system	0
ByuC7	[X]Secondary malignant neoplasm of other specified sites	0
C354A	Metastatic calcification	0
F4314	Metastatic disseminated retinitis	0

CONCEPT: Multiple sclerosis

READ_CODE	DESCRIPTION_SAIL	SENSITIVE_CODE
666A.	Multiple sclerosis review	0
666B.	Multiple sclerosis multidisciplinary review	0
8CS1.	Multiple sclerosis care plan agreed	0
8Cc0.	Management of multiple sclerosis in onset phase	0
8Cc1.	Management of multiple sclerosis in early disease phase	0
8Cc2.	Management of multiple sclerosis in stable disability phase	0
8Cc4.	Management of multiple sclerosis in palliative phase	0
8IAb.	Multiple sclerosis review declined	0
9kG..	Specialised services for patients with multiple sclerosis - enhanced services administration	0
9mD..	Multiple sclerosis monitoring administration	0
9mD0.	Multiple sclerosis monitoring first letter	0
9mD1.	Multiple sclerosis monitoring second letter	0
9mD2.	Multiple sclerosis monitoring telephone invitation	0
9mD3.	Multiple sclerosis monitoring third letter	0
F20..	Multiple sclerosis	0
F200.	Multiple sclerosis of the brain stem	0
F201.	Multiple sclerosis of the spinal cord	0
F202.	Generalised multiple sclerosis	0
F203.	Exacerbation of multiple sclerosis	0
F204.	Benign multiple sclerosis	0
F205.	Malignant multiple sclerosis	0
F206.	Primary progressive multiple sclerosis	0
F207.	Relapsing and remitting multiple sclerosis	0
F208.	Secondary progressive multiple sclerosis	0
F20z.	Multiple sclerosis NOS	0

CONCEPT: Osteoarthritis

READ_CODE	DESCRIPTION_SAIL	SENSITIVE_CODE
14G2.	H/O: osteoarthritis	0
7P204	Delivery of rehabilitation for osteoarthritis	0
9hQ..	Exception reporting: osteoarthritis quality indicators	0
9hQ0.	Excepted from osteoarthritis quality indicators: patient unsuitable	0
9hQ1.	Excepted from osteoarthritis quality indicators: informed dissent	0
F1632	Myelopathy due to spondylosis	0
F3372	Nerve root and plexus compressions in spondylosis	0
N0...	Arthropathies and related disorders	0
N05..	Osteoarthritis and allied disorders	0
N050.	Generalised osteoarthritis - OA	0
N0500	Generalised osteoarthritis of unspecified site	0
N0501	Generalised osteoarthritis of the hand	0
N0502	Generalised osteoarthritis of multiple sites	0
N0504	Primary generalized osteoarthrosis	0
N0505	Secondary multiple arthrosis	0
N0506	Erosive osteoarthrosis	0
N0507	Heberden's nodes with arthropathy	0
N050z	Generalised osteoarthritis NOS	0
N051.	Localised, primary osteoarthritis	0
N0510	Localised, primary osteoarthritis of unspecified site	0
N0511	Localised, primary osteoarthritis of the shoulder region	0
N0512	Localised, primary osteoarthritis of the upper arm	0
N0513	Localised, primary osteoarthritis of the forearm	0
N0514	Localised, primary osteoarthritis of the hand	0
N0515	Localised, primary osteoarthritis of the pelvic region and thigh	0
N0516	Localised, primary osteoarthritis of the lower leg	0
N0517	Localised, primary osteoarthritis of the ankle and foot	0
N0518	Localised, primary osteoarthritis of other specified site	0
N0519	Primary coxarthrosis, bilateral	0
N051B	Primary gonarthrosis, bilateral	0
N051C	Primary arthrosis of first carpometacarpal joints, bilateral	0
N051D	Localised, primary osteoarthritis of the wrist	0
N051E	Localised, primary osteoarthritis of toe	0
N051F	Localised, primary osteoarthritis of elbow	0
N051G	Osteoarthritis of spinal facet joint	0
N051z	Localised, primary osteoarthritis NOS	0
N052.	Localised, secondary osteoarthritis	0
N0520	Localised, secondary osteoarthritis of unspecified site	0
N0521	Localised, secondary osteoarthritis of the shoulder region	0
N0522	Localised, secondary osteoarthritis of the upper arm	0
N0523	Localised, secondary osteoarthritis of the forearm	0
N0524	Localised, secondary osteoarthritis of the hand	0
N0525	Localised, secondary osteoarthritis of the pelvic region and thigh	0
N0526	Localised, secondary osteoarthritis of the lower leg	0
N0527	Localised, secondary osteoarthritis of the ankle and foot	0
N0528	Localised, secondary osteoarthritis of other specified site	0
N0529	Post-traumatic coxarthrosis, bilateral	0
N052A	Post-traumatic gonarthrosis, bilateral	0
N052B	Post-traumatic arthrosis of first carpometacarpal joints, bilateral	0
N052C	Post-traumatic gonarthrosis, unilateral	0
N052z	Localised, secondary osteoarthritis NOS	0
N053.	Localised osteoarthritis, unspecified	0
N0530	Localised osteoarthritis, unspecified, of unspecified site	0
N0531	Localised osteoarthritis, unspecified, of the shoulder region	0
N0532	Localised osteoarthritis, unspecified, of the upper arm	0
N0533	Localised osteoarthritis, unspecified, of the forearm	0
N0534	Localised osteoarthritis, unspecified, of the hand	0
N0535	Localised osteoarthritis, unspecified, of the pelvic region and thigh	0
N0536	Localised osteoarthritis, unspecified, of the lower leg	0
N0537	Localised osteoarthritis, unspecified, of the ankle and foot	0
N0538	Localised osteoarthritis, unspecified, of other specified site	0
N0539	Arthrosis of first carpometacarpal joint, unspecified	0
N053z	Localised osteoarthritis, unspecified, NOS	0
N054.	Oligoarticular osteoarthritis, unspecified	0
N0540	Oligoarticular osteoarthritis, unspecified, of unspecified sites	0
N0541	Oligoarticular osteoarthritis, unspecified, of the shoulder region	0
N0542	Oligoarticular osteoarthritis, unspecified, of upper arm	0
N0543	Oligoarticular osteoarthritis, unspecified, of forearm	0
N0544	Oligoarticular osteoarthritis, unspecified, of hand	0
N0545	Oligoarticular osteoarthritis, unspecified, of the pelvic region and thigh	0
N0546	Oligoarticular osteoarthritis, unspecified, of lower leg	0
N0547	Oligoarticular osteoarthritis, unspecified, of ankle and foot	0
N0548	Oligoarticular osteoarthritis, unspecified, of other specified sites	0

N0549	Oligoarticular osteoarthritis, unspecified, of multiple sites	0
N054z	Osteoarthritis of more than one site, unspecified, NOS	0
N05z.	Osteoarthritis NOS	0
N05z0	Osteoarthritis NOS, of unspecified site	0
N05z1	Osteoarthritis NOS, of shoulder region	0
N05z2	Osteoarthritis NOS, of the upper arm	0
N05z3	Osteoarthritis NOS, of the forearm	0
N05z4	Osteoarthritis NOS, of the hand	0
N05z5	Osteoarthritis NOS, pelvic region/thigh	0
N05z6	Osteoarthritis NOS, of the lower leg	0
N05z7	Osteoarthritis NOS, of ankle and foot	0
N05z8	Osteoarthritis NOS, other specified site	0
N05z9	Osteoarthritis NOS, of shoulder	0
N05zA	Osteoarthritis NOS, of sternoclavicular joint	0
N05zB	Osteoarthritis NOS, of acromioclavicular joint	0
N05zC	Osteoarthritis NOS, of elbow	0
N05zD	Osteoarthritis NOS, of distal radio-ulnar joint	0
N05zE	Osteoarthritis NOS, of wrist	0
N05zF	Osteoarthritis NOS, of metacarpophalangeal joint	0
N05zG	Osteoarthritis NOS, of proximal interphalangeal joint of finger	0
N05zH	Osteoarthritis NOS, of distal interphalangeal joint of finger	0
N05zJ	Osteoarthritis NOS, of hip	0
N05zK	Osteoarthritis NOS, of sacro-iliac joint	0
N05zL	Osteoarthritis NOS, of knee	0
N05zM	Osteoarthritis NOS, of tibio-fibular joint	0
N05zN	Osteoarthritis NOS, of ankle	0
N05zP	Osteoarthritis NOS, of subtalar joint	0
N05zQ	Osteoarthritis NOS, of talonavicular joint	0
N05zR	Osteoarthritis NOS, of other tarsal joint	0
N05zS	Osteoarthritis NOS, of 1st metatarsophalangeal joint	0
N05zT	Osteoarthritis NOS, of lesser metatarsophalangeal joint	0
N05zU	Osteoarthritis NOS, of interphalangeal joint of toe	0
N05zz	Osteoarthritis NOS	0
N11..	Spondylosis and allied disorders	0
N110.	Cervical spondylosis without myelopathy	0
N1100	Single-level cervical spondylosis without myelopathy	0
N1101	Two-level cervical spondylosis without myelopathy	0
N1102	Multiple-level cervical spondylosis without myelopathy	0
N111.	Cervical spondylosis with myelopathy	0
N1110	Single-level cervical spondylosis with myelopathy	0
N1111	Two-level cervical spondylosis with myelopathy	0
N1112	Multiple-level cervical spondylosis with myelopathy	0
N112.	Thoracic spondylosis without myelopathy	0
N1120	Single-level thoracic spondylosis without myelopathy	0
N1121	Two-level thoracic spondylosis without myelopathy	0
N1122	Multiple-level thoracic spondylosis without myelopathy	0
N1123	Dorsal spondylosis without myelopathy	0
N113.	Thoracic spondylosis with myelopathy	0
N1130	Single-level thoracic spondylosis with myelopathy	0
N1131	Two-level thoracic spondylosis with myelopathy	0
N1132	Multiple-level thoracic spondylosis with myelopathy	0
N114.	Lumbosacral spondylosis without myelopathy	0
N1140	Single-level lumbosacral spondylosis without myelopathy	0
N1141	Two-level lumbosacral spondylosis without myelopathy	0
N1142	Multiple-level lumbosacral spondylosis without myelopathy	0
N115.	Lumbosacral spondylosis with myelopathy	0
N1150	Single-level lumbosacral spondylosis with myelopathy	0
N1151	Two-level lumbosacral spondylosis with myelopathy	0
N1152	Multiple-level lumbosacral spondylosis with myelopathy	0
N119.	Cervical spondylosis with radiculopathy	0
N1190	Single-level cervical spondylosis with radiculopathy	0
N1191	Two-level cervical spondylosis with radiculopathy	0
N1192	Multiple-level cervical spondylosis with radiculopathy	0
N11A.	Cervical spondylosis with vascular compression	0
N11B.	Thoracic spondylosis with radiculopathy	0
N11B0	Single-level thoracic spondylosis with radiculopathy	0
N11B1	Two-level thoracic spondylosis with radiculopathy	0
N11B2	Multiple-level thoracic spondylosis with radiculopathy	0
N11C.	Lumbosacral spondylosis with radiculopathy	0
N11C0	Single-level lumbosacral spondylosis with radiculopathy	0
N11C1	Two-level lumbosacral spondylosis with radiculopathy	0
N11C2	Multiple-level lumbosacral spondylosis with radiculopathy	0
N11D.	Osteoarthritis of spine	0
N11D0	Osteoarthritis of cervical spine	0
N11D1	Osteoarthritis of thoracic spine	0
N11D2	Osteoarthritis of lumbar spine	0
N11D3	Osteoarthritis of spine NOS	0
N11E.	Cervical spondylosis	0
N11y.	Other spondyloses and allied disorders	0
N11z.	Spondylosis NOS	0
N11z0	Spondylosis without myelopathy, NOS	0
N11z1	Spondylosis with myelopathy, NOS	0
N11zz	Spondylosis NOS	0
Nyu2.	[X]Arthrosis	0
Nyu20	[X]Other polyarthrosis	0
Nyu21	[X]Other primary coxarthrosis	0
Nyu23	[X]Other post-traumatic coxarthrosis	0
Nyu24	[X]Other secondary coxarthrosis, bilateral	0
Nyu25	[X]Other primary gonarthrosis	0
Nyu26	[X]Other post-traumatic gonarthrosis	0
Nyu27	[X]Other secondary gonarthrosis, bilateral	0
Nyu28	[X]Other secondary gonarthrosis	0
Nyu29	[X]Other primary arthrosis of first carpometacarpal joint	0
Nyu2A	[X]Other post-traumatic arthrosis of first carpometacarpal joint	0
Nyu2B	[X]Other secondary arthrosis of first carpometacarpal joints, bilateral	0
Nyu2C	[X]Other secondary arthrosis of first carpometacarpal joint	0
Nyu2D	[X]Other specified arthrosis	0
Nyu2E	[X]Other secondary coxarthrosis	0
Nyu2F	[X]Post-traumatic arthrosis of other joints	0
Nyu62	[X]Other spondylosis with myelopathy	0
Nyu63	[X]Other spondylosis with radiculopathy	0
Nyu64	[X]Other spondylosis	0

CONCEPT: Osteoporosis

READ_CODE	DESCRIPTION_SAIL	SENSITIVE_CODE
14G6.	H/O: fragility fracture	0
14GB.	History of osteoporosis	0
585O.	Quantitative ultrasound scan of heel - result osteoporotic	0
58E4.	Forearm DXA scan result osteoporotic	0
58EA.	Heel DXA scan result osteoporotic	0
58EG.	Hip DXA scan result osteoporotic	0
58EM.	Lumbar DXA scan result osteoporotic	0
58EV.	Femoral neck DEXA scan result osteoporotic	0
66a2.	Osteoporosis treatment started	0
66a3.	Osteoporosis treatment stopped	0
66a4.	Osteoporosis treatment changed	0
66a5.	Osteoporosis - no treatment	0
66aA.	Osteoporosis - treatment response	0
66aB.	Osteoporosis - no treatment response	0
8B31E	Osteoporosis medication compliance review	0
9kj..	Osteoporosis - enhanced services administration	0
9kj0.	Bone sparing drug treatment offered for osteoporosis - enhanced services administration	0
N330.	Osteoporosis	0
N3300	Osteoporosis, unspecified	0
N3301	Senile osteoporosis	0
N3302	Postmenopausal osteoporosis	0
N3303	Idiopathic osteoporosis	0
N3304	Dissuse osteoporosis	0
N3305	Drug-induced osteoporosis	0
N3306	Postoophorectomy osteoporosis	0
N3307	Postsurgical malabsorption osteoporosis	0
N3308	Localized osteoporosis - Lequesne	0
N3309	Osteoporosis in multiple myelomatosis	0
N330A	Osteoporosis in endocrine disorders	0
N330B	Vertebral osteoporosis	0
N330C	Osteoporosis localized to spine	0
N330D	Osteoporosis due to corticosteroids	0
N330z	Osteoporosis NOS	0
N331.	Pathological fracture	0
N3312	Postoophorectomy osteoporosis with pathological fracture	0
N3313	Osteoporosis of disuse with pathological fracture	0
N3314	Postsurgical malabsorption osteoporosis with pathological fracture	0
N3315	Drug-induced osteoporosis with pathological fracture	0
N3316	Idiopathic osteoporosis with pathological fracture	0
N3318	Osteoporosis + pathological fracture lumbar vertebrae	0
N3319	Osteoporosis + pathological fracture thoracic vertebrae	0
N331A	Osteoporosis + pathological fracture cervical vertebrae	0
N331B	Postmenopausal osteoporosis with pathological fracture	0
N331H	Collapse of cervical vertebra due to osteoporosis	0
N331J	Collapse of lumbar vertebra due to osteoporosis	0
N331K	Collapse of thoracic vertebra due to osteoporosis	0
N331L	Collapse of vertebra due to osteoporosis NOS	0
N331M	Fragility fracture due to unspecified osteoporosis	0
N331N	Fragility fracture	0
N3370	Disuse atrophy of bone	0
N3746	Osteoporotic kyphosis	0
NyuB0	[X]Other osteoporosis with pathological fracture	0
NyuB1	[X]Other osteoporosis	0
NyuB2	[X]Osteoporosis in other disorders classified elsewhere	0
NyuB8	[X]Unspecified osteoporosis with pathological fracture	0

CONCEPT: Pancreatic disease

READ_CODE	DESCRIPTION_SAIL	SENSITIVE_CODE
14CH.	History of chronic pancreatitis	0
J671.	Chronic pancreatitis	0
J6710	Alcohol-induced chronic pancreatitis	0
J6711	Gallstone chronic pancreatitis	0
J67y0	Atrophy of pancreas	0
J67y2	Fibrosis of pancreas	0
Jyu84	[X]Other chronic pancreatitis	0

CONCEPT: Paralysis

READ_CODE	DESCRIPTION_SAIL	SENSITIVE_CODE
2833.	O/E - hemiplegia	0
2835.	O/E - paraplegia	0
2836.	O/E - quadriplegia	0
F038.	Tropical spastic paraplegia	0
F141.	Hereditary spastic paraplegia	0
F22..	Hemiplegia	0
F220.	Flaccid hemiplegia	0
F221.	Spastic hemiplegia	0
F222.	Left hemiplegia	0
F223.	Right hemiplegia	0
F22z.	Hemiplegia NOS	0
F230.	Congenital diplegia	0
F2300	Congenital paraplegia	0
F231.	Congenital hemiplegia	0
F232.	Congenital quadriplegia	0
F234.	Infantile hemiplegia NOS	0
F240.	Quadriplegia	0
F2400	Flaccid tetraplegia	0
F2401	Spastic tetraplegia	0
F241.	Paraplegia	0
F2410	Flaccid paraplegia	0
F2411	Spastic paraplegia	0
F2B0.	Spastic quadriplegic cerebral palsy	0
F2B1.	Spastic hemiplegic cerebral palsy	0
N2310	Muscular calcification and ossification, unspecified	0

CONCEPT: Parkinson's disease

READ_CODE	DESCRIPTION_SAIL	SENSITIVE_CODE
147F.	History of Parkinson's disease	0
297A.	O/E - Parkinsonian tremor	0

2987.	O/E -Parkinson flexion posture	0
2994.	O/E-festination-Parkinson gait	0
A94y1	Syphilitic parkinsonism	1
Eu023	[X]Dementia in Parkinson's disease	0
F11x9	Cerebral degeneration in Parkinson's disease	0
F12..	Parkinson's disease	0
F120.	Paralysis agitans	0
F121.	Parkinsonism secondary to drugs	0
F123.	Postencephalitic parkinsonism	0
F124.	Vascular parkinsonism	0
F12W.	Secondary parkinsonism due to other external agents	0
F12X.	Secondary parkinsonism, unspecified	0
F12z.	Parkinson's disease NOS	0
F1303	Parkinsonism with orthostatic hypotension	0
Fyu20	[X]Other drug-induced secondary parkinsonism	0
Fyu21	[X]Other secondary parkinsonism	0
Fyu22	[X]Parkinsonism in diseases classified elsewhere	0
Fyu29	[X]Secondary parkinsonism, unspecified	0
Fyu2B	[X]Secondary parkinsonism due to other external agents	0

CONCEPT: PCOS (Polycystic ovary syndrome)

READ_CODE	DESCRIPTION_SAIL	SENSITIVE_CODE
7E253	Endoscopic drilling of ovary	0
C164.	Polycystic ovaries	0
C165.	Polycystic ovarian syndrome	0

CONCEPT: Plasmacell

READ_CODE	DESCRIPTION_SAIL	SENSITIVE_CODE
4C53.	Bone marrow: myeloma cells	0
B63..	Multiple myeloma and immunoproliferative neoplasms	0
B630.	Multiple myeloma	0
B6300	Malignant plasma cell neoplasm, extramedullary plasmacytoma	0
B6301	Solitary myeloma	0
B6302	Plasmacytoma NOS	0
B6303	Lambda light chain myeloma	0
B6304	Solitary plasmacytoma	0
B631.	Plasma cell leukaemia	0
B63y.	Other immunoproliferative neoplasms	0
B63z.	Immunoproliferative neoplasm or myeloma NOS	0
B936.	Neoplasm of uncertain behaviour of plasma cells	0
BBm6.	[M] Alpha heavy chain disease	0
BBmE.	[M] Gamma heavy chain disease	0
BBn..	[M]Plasma cell tumours	0
BBn0.	[M]Plasma cell myeloma	0
BBn2.	[M]Plasmacytoma NOS	0
BBn3.	[M]Plasma cell tumour, malignant	0
BBnz.	[M]Plasma cell tumour NOS	0
BBr3.	[M]Plasma cell leukaemias	0
BBr30	[M]Plasma cell leukaemia	0
BBr3z	[M]Plasma cell leukaemia NOS	0
C3331	Alpha heavy chain disease	0
C3332	Gamma heavy chain disease	0
C3333	Heavy chain disease	0
N3309	Osteoporosis in multiple myelomatosis	0

CONCEPT: PMR (Polymyalgia reheumatica)

READ_CODE	DESCRIPTION_SAIL	SENSITIVE_CODE
N20..	Polymyalgia rheumatica	0
N200.	Giant cell arteritis with polymyalgia rheumatica	0

CONCEPT: Primary Lung Cancer

READ_CODE	DESCRIPTION_SAIL	SENSITIVE_CODE
1422.	H/O: * bronchus	0
1423.	H/O: * trachea/lung	0
B22..	Malignant neoplasm of trachea, bronchus and lung	0
B220.	Malignant neoplasm of trachea	0
B2200	Malignant neoplasm of cartilage of trachea	0
B2201	Malignant neoplasm of mucosa of trachea	0
B220z	Malignant neoplasm of trachea NOS	0
B221.	Malignant neoplasm of main bronchus	0
B2210	Malignant neoplasm of carina of bronchus	0
B2211	Malignant neoplasm of hilus of lung	0
B221z	Malignant neoplasm of main bronchus NOS	0
B222.	Malignant neoplasm of upper lobe, bronchus or lung	0
B2220	Malignant neoplasm of upper lobe bronchus	0
B2221	Malignant neoplasm of upper lobe of lung	0
B222z	Malignant neoplasm of upper lobe, bronchus or lung NOS	0
B223.	Malignant neoplasm of middle lobe, bronchus or lung	0
B2230	Malignant neoplasm of middle lobe bronchus	0
B2231	Malignant neoplasm of middle lobe of lung	0
B223z	Malignant neoplasm of middle lobe, bronchus or lung NOS	0
B224.	Malignant neoplasm of lower lobe, bronchus or lung	0
B2240	Malignant neoplasm of lower lobe bronchus	0
B2241	Malignant neoplasm of lower lobe of lung	0
B224z	Malignant neoplasm of lower lobe, bronchus or lung NOS	0
B225.	Malignant neoplasm of overlapping lesion of bronchus and lung	0
B22y.	Malignant neoplasm of other sites of bronchus or lung	0
B22z.	Malignant neoplasm of bronchus or lung NOS	0
BB5S2	[M]Bronchiolo-alveolar adenocarcinoma	0
BB5S4	[M]Alveolar adenocarcinoma	0
Byu2.	[X]Malignant neoplasm of respiratory and intrathoracic organs	0
Byu20	[X]Malignant neoplasm of bronchus or lung, unspecified	0
ZV101	[V]Personal history of malignant neoplasm of trachea, bronchus or lung	0

CONCEPT: Prostate Cancer

READ_CODE	DESCRIPTION_SAIL	SENSITIVE_CODE
4M01.	Gleason prostate grade 5-7 (medium)	0
4M02.	Gleason prostate grade 8-10 (high)	0
5B51.	Microwave therapy to prostate	0

7B3B0	Endoscopic cryotherapy of prostate	0
7B3B1	Other endoscopic destruction of lesion of prostate	0
7B3B6	Ultrasound ablation of prostate	0
7B3BC	Endoscopic radiofrequency ablation of lesion of prostate	0
7B3C6	Cryotherapy to prostate via perineal probe	0
7B3E0	High intensity focused ultrasound of prostate	0
8AD0.	Active surveillance of prostate cancer	0
8BAV0	Prostate cancer care review	0
B46..	Malignant neoplasm of prostate	0
B58y5	Secondary malignant neoplasm of prostate	0

CONCEPT: Prostate disorders

READ_CODE	DESCRIPTION_SAIL	SENSITIVE_CODE
14E1.	H/O: prostatism	0
1AA..	Prostatism	0
7B395	Endoscopic resection of prostate using an electrotome	0
8L51.	Prostatectomy planned	0
A1650	Tuberculosis of prostate	0
A9812	Acute gonococcal prostatitis	1
A9832	Chronic gonococcal prostatitis	1
AD103	Trichomonal prostatitis	1
B46..	Malignant neoplasm of prostate	0
B58y5	Secondary malignant neoplasm of prostate	0
B7C2.	Benign neoplasm of prostate	0
B834.	Carcinoma in situ of prostate	0
B8340	High grade prostatic intraepithelial neoplasia	0
B915.	Neoplasm of uncertain behaviour of prostate	0
K20..	Benign prostatic hypertrophy	0
K200.	Prostatic hyperplasia unspecified	0
K201.	Prostatic hyperplasia of the lateral lobe	0
K202.	Prostatic hyperplasia of the medial lobe	0
K20z.	Prostatic hyperplasia NOS	0
K21..	Prostate inflammatory diseases	0
K210.	Acute prostatitis	0
K211.	Chronic prostatitis	0
K212.	Abscess of prostate	0
K213.	Prostatocystitis	0
K214.	Prostatitis in diseases EC	0
K2140	Prostatitis in actinomycosis	0
K2141	Prostatitis in blastomycosis	0
K2142	Prostatitis in syphilis	1
K2143	Prostatitis in tuberculosis	0
K2144	Prostatitis in gonorrhoea	1
K2145	Prostatitis in moniliasis	0
K2146	Prostatitis in trichomoniasis	1
K214z	Prostatitis in diseases EC NOS	0
K21y.	Other prostatic inflammatory diseases	0
K21z.	Prostatitis NOS	0
K22..	Other disorders of prostate	0
K220.	Calculus of prostate	0
K221.	Prostatic congestion or haemorrhage	0
K2210	Prostatic congestion	0
K2211	Prostatic haemorrhage	0
K221z	Prostatic congestion or haemorrhage NOS	0
K222.	Atrophy of prostate	0
K22y.	Other disorders of prostate OS	0
K22y0	Prostatic fistula	0
K22y1	Infarction of prostate	0
K22y2	Stricture of prostate	0
K22y3	Periprostatic adhesions	0
K22yz	Other prostate disorders NOS	0
K22z.	Prostatic disorders NOS	0
Kyu60	[X]Other inflammatory diseases of prostate	0
Kyu61	[X]Other specified disorders of prostate	0
Kyu68	[X]Disorders of prostate in diseases classified elsewhere	0
PCy01	Congenital absence of prostate	0
PCyx.	Other congenital anomaly of vas deferens or prostate	0
Pyu69	[X]Other congenital malformations of vas deferens, epididymis, seminal vesicles and prostate	0

CONCEPT: Psoriasis

READ_CODE	DESCRIPTION_SAIL	SENSITIVE_CODE
14F2.	H/O: psoriasis	0
38Gg.	Psoriasis area and severity index	0
M16..	Psoriasis and similar disorders	0
M161.	Other psoriasis	0
M1610	Psoriasis unspecified	0
M1611	Psoriasis annularis	0
M1612	Psoriasis circinata	0
M1613	Psoriasis diffusa	0
M1614	Psoriasis discoidea	0
M1615	Psoriasis geographica	0
M1616	Guttate psoriasis	0
M1617	Psoriasis gyrata	0
M1618	Psoriasis inveterata	0
M1619	Psoriasis ostracea	0
M161A	Psoriasis palmaris	0
M161B	Psoriasis plantaris	0
M161C	Psoriasis punctata	0
M161D	Pustular psoriasis	0
M161E	Psoriasis universalis	0
M161F	Psoriasis vulgaris	0
M161H	Erythrodermic psoriasis	0
M161J	Flexural psoriasis	0
M161z	Psoriasis NOS	0
M166.	Palmoplantar pustular psoriasis	0
M16y.	Other psoriasis and similar disorders	0
M16y0	Scalp psoriasis	0
M16z.	Psoriasis and similar disorders NOS	0
Myu30	[X]Other psoriasis	0

CONCEPT: Psoriasis Arthritis

READ_CODE	DESCRIPTION_SAIL	SENSITIVE_CODE
M160.	Psoriatic arthropathy	0
M1600	Psoriasis spondylitica	0
M1601	Distal interphalangeal psoriatic arthropathy	0
M1602	Arthritis mutilans	0
M160z	Psoriatic arthropathy NOS	0
N0452	Juvenile arthritis in psoriasis	0
Nyu13	[X]Other psoriatic arthropathies	0

CONCEPT: PTSD (Post-traumatic stress disorders)

READ_CODE	DESCRIPTION_SAIL	SENSITIVE_CODE
1S1..	Flashbacks	0
1S10.	Short duration flashbacks	0
1S11.	Long duration flashbacks	0
1S12.	Episodic flashbacks	0
1S13.	Duplicative flashbacks	0
E2831	Acute posttrauma stress state	0
E29y1	Other post-traumatic stress disorder	0
Eu431	[X]Post - traumatic stress disorder	0

CONCEPT: Pheripheral vascular disease

READ_CODE	DESCRIPTION_SAIL	SENSITIVE_CODE
14NB.	H/O: Peripheral vascular disease procedure	0
1M110	Ischaemic foot pain at rest	0
1M111	Ischaemic foot pain when walking	0
2G63.	Ischaemic toe	0
5593.	Femoral arteriogram abnormal	0
55A2.	Lower limb arteriogram abnorm.	0
727A4	Scanning laser ophthalmoscopy evaluation of retina	0
7A10.	Extraanatomic bypass of aorta	0
7A100	Emergency bypass of aorta by anastomosis of axillary artery to femoral artery	0
7A101	Bypass of aorta by anastomosis of axillary artery to femoral artery NEC	0
7A102	Axillo-bifemoral bypass graft	0
7A103	Axillo-unifemoral PTFE bypass graft	0
7A104	Bypass of aorta by anastomosis of axillary artery to bilateral femoral arteries	0
7A10y	Other specified extraanatomic bypass of aorta	0
7A10z	Extraanatomic bypass of aorta NOS	0
7A192	Open embolectomy of bifurcation of aorta	0
7A4..	Iliac and femoral artery operations	0
7A40.	Replacement of aneurysmal iliac artery	0
7A400	Emergency replacement of aneurysmal iliac artery by anastomosis of iliac artery to femoral artery	0
7A401	Replacement of aneurysmal iliac artery by anastomosis of iliac artery to femoral artery NEC	0
7A402	Emergency replacement of aneurysmal iliac artery by anastomosis of femoral artery to femoral artery	0
7A403	Replacement of aneurysmal iliac artery by anastomosis of femoral artery to femoral artery NEC	0
7A404	Emergency replacement of aneurysmal common iliac artery by anastomosis of aorta to common iliac artery	0
7A405	Emergency replacement of aneurysmal iliac artery by anastomosis of aorta to external iliac artery	0
7A406	Emergency replacement of aneurysmal artery of leg by anastomosis of aorta to common femoral artery	0
7A407	Emergency replacement of aneurysmal artery of leg by anastomosis of aorta to superficial femoral artery	0
7A408	Emergency replacement of aneurysmal iliac artery by anastomosis of iliac artery to iliac artery	0
7A409	Replacement of aneurysmal common iliac artery by anastomosis of aorta to common iliac artery NEC	0
7A40A	Replacement of aneurysmal iliac artery by anastomosis of aorta to external iliac artery NEC	0
7A40B	Replacement of aneurysmal artery of leg by anastomosis of aorta to common femoral artery NEC	0
7A40C	Replacement of aneurysmal artery of leg by anastomosis of aorta to superficial femoral artery NEC	0
7A40D	Replacement of aneurysmal iliac artery by anastomosis of iliac artery to iliac artery NEC	0
7A40y	Other specified replacement of aneurysmal iliac artery	0
7A40z	Replacement of aneurysmal iliac artery NOS	0
7A41.	Other bypass of iliac artery	0
7A410	Emergency bypass of iliac artery by anastomosis of iliac artery to femoral artery NEC	0
7A411	Bypass of iliac artery by anastomosis of iliac artery to femoral artery NEC	0
7A412	Emergency bypass of iliac artery by anastomosis of femoral artery to femoral artery NEC	0
7A413	Bypass of iliac artery by anastomosis of femoral artery to femoral artery NEC	0
7A414	Emergency bypass of common iliac artery by anastomosis of aorta to common iliac artery NEC	0
7A415	Emergency bypass of iliac artery by anastomosis of aorta to external iliac artery NEC	0
7A416	Emergency bypass of artery of leg by anastomosis of aorta to common femoral artery NEC	0
7A417	Emergency bypass of artery of leg by anastomosis of aorta to deep femoral artery NEC	0
7A418	Emergency bypass of iliac artery by anastomosis of iliac artery to iliac artery NEC	0
7A419	Bypass of common iliac artery by anastomosis of aorta to common iliac artery NEC	0
7A41A	Bypass of iliac artery by anastomosis of aorta to external iliac NEC	0
7A41B	Bypass of artery of leg by anastomosis of aorta to common femoral artery NEC	0
7A41C	Bypass of artery of leg by anastomosis of aorta to deep femoral artery NEC	0
7A41D	Bypass of iliac artery by anastomosis of iliac artery to iliac artery NEC	0
7A41E	Emergency bypass of iliac artery by unspecified anastomosis	0
7A41F	Ilio-femoral prosthetic cross over graft	0
7A41y	Other specified other bypass of iliac artery	0
7A41z	Other bypass of iliac artery NOS	0
7A42.	Reconstruction of iliac artery	0
7A420	Endarterectomy of iliac artery and patch repair of iliac artery	0
7A421	Endarterectomy of iliac artery NEC	0
7A42y	Other specified reconstruction of iliac artery	0
7A42z	Reconstruction of iliac artery NOS	0
7A43.	Other open operations on iliac artery	0
7A430	Repair of iliac artery NEC	0
7A431	Open embolectomy of iliac artery	0
7A432	Operation on aneurysm of iliac artery NEC	0
7A433	Open insertion of iliac artery stent	0
7A43y	Other specified other open operation on iliac artery	0
7A43z	Other open operation on iliac artery NOS	0
7A44.	Transluminal operations on iliac artery	0
7A440	Percutaneous transluminal angioplasty of iliac artery	0
7A441	Percutaneous transluminal embolectomy of iliac artery	0
7A442	Arteriography of iliac artery	0
7A443	Insertion of iliac artery stent	0
7A444	Percutaneous transluminal insertion of iliac artery stent	0
7A44y	Other specified transluminal operation on iliac artery	0
7A44z	Transluminal operation on iliac artery NOS	0
7A45.	Emergency replacement of aneurysmal femoral or popliteal artery	0
7A450	Emergency replacement of aneurysmal femoral artery by anastomosis of femoral artery to popliteal artery using prosthesis	0
7A451	Emergency replacement of aneurysmal popliteal artery by anastomosis of popliteal artery to popliteal artery using prosthesis	0
7A452	Emergency replacement of aneurysmal femoral artery by anastomosis of femoral artery to popliteal artery using vein graft	0
7A453	Emergency replacement of aneurysmal popliteal artery by anastomosis of popliteal artery to popliteal artery using vein graft	0
7A454	Emergency replacement of aneurysmal femoral artery by anastomosis of femoral artery to tibial artery using prosthesis	0

7A455	Emergency replacement of aneurysmal popliteal artery by anastomosis of popliteal artery to tibial artery using prosthesis	0
7A456	Emergency replacement of aneurysmal femoral artery by anastomosis of femoral artery to tibial artery using vein graft	0
7A457	Emergency replacement of aneurysmal popliteal artery by anastomosis of popliteal artery to tibial artery using vein graft	0
7A458	Emergency replacement of aneurysmal femoral artery by anastomosis of femoral artery to peroneal artery using prosthesis	0
7A459	Emergency replacement of aneurysmal popliteal artery by anastomosis of popliteal artery to peroneal artery using prosthesis	0
7A45A	Emergency replacement of aneurysmal femoral artery by anastomosis of femoral artery to peroneal artery using vein graft	0
7A45B	Emergency replacement of aneurysmal popliteal artery by anastomosis of popliteal artery to peroneal artery using vein graft	0
7A45C	Emergency replacement of aneurysmal femoral artery by anastomosis of femoral artery to femoral artery	0
7A45D	Emergency replacement of aneurysmal popliteal artery by anastomosis of popliteal artery to femoral artery	0
7A45y	Other specified emergency replacement of aneurysmal femoral artery or popliteal artery	0
7A45z	Emergency replacement of aneurysmal femoral artery or popliteal artery NOS	0
7A46.	Other replacement of aneurysmal femoral artery	0
7A460	Replacement of aneurysmal femoral artery by anastomosis of femoral artery to popliteal artery using prosthesis NEC	0
7A461	Replacement of aneurysmal popliteal artery by anastomosis of popliteal artery to popliteal artery using prosthesis NEC	0
7A462	Replacement of aneurysmal femoral artery by anastomosis of femoral artery to popliteal artery using vein graft NEC	0
7A463	Replacement of aneurysmal popliteal artery by anastomosis of popliteal artery to popliteal artery using vein graft NEC	0
7A464	Replacement of aneurysmal femoral artery by anastomosis of femoral artery to tibial artery using prosthesis NEC	0
7A465	Replacement of aneurysmal popliteal artery by anastomosis of popliteal artery to tibial artery using prosthesis NEC	0
7A466	Replacement of aneurysmal femoral artery by anastomosis of femoral artery to tibial artery using vein graft NEC	0
7A467	Replacement of aneurysmal popliteal artery by anastomosis of popliteal artery to tibial artery using vein graft NEC	0
7A468	Replacement of aneurysmal femoral artery by anastomosis of femoral artery to peroneal artery using prosthesis NEC	0
7A469	Replacement of aneurysmal popliteal artery by anastomosis of popliteal artery to peroneal artery using prosthesis NEC	0
7A46A	Replacement of aneurysmal femoral artery by anastomosis of femoral artery to peroneal artery using vein graft NEC	0
7A46B	Replacement of aneurysmal popliteal artery by anastomosis of popliteal artery to peroneal artery using vein graft NEC	0
7A46C	Replacement of aneurysmal femoral artery by anastomosis of femoral artery to femoral artery NEC	0
7A46D	Replacement of aneurysmal popliteal artery by anastomosis of popliteal artery to femoral artery NEC	0
7A46y	Other specified other replacement of aneurysmal femoral artery or popliteal artery	0
7A46z	Other replacement of aneurysmal femoral artery or popliteal artery NOS	0
7A47.	Other emergency bypass of femoral artery or popliteal artery	0
7A470	Emergency bypass of femoral artery by anastomosis of femoral artery to popliteal artery using prosthesis NEC	0
7A471	Emergency bypass of popliteal artery by anastomosis of popliteal artery to popliteal artery using prosthesis NEC	0
7A472	Emergency bypass of femoral artery by anastomosis of femoral artery to popliteal artery using vein graft NEC	0
7A473	Emergency bypass of popliteal artery by anastomosis of popliteal artery to popliteal artery using vein graft NEC	0
7A474	Emergency bypass of femoral artery by anastomosis of femoral artery to tibial artery using prosthesis NEC	0
7A475	Emergency bypass of popliteal artery by anastomosis of popliteal artery to tibial artery using prosthesis NEC	0
7A476	Emergency bypass of femoral artery by anastomosis of femoral artery to tibial artery using vein graft NEC	0
7A477	Emergency bypass of popliteal artery by anastomosis of popliteal artery to tibial artery using vein graft NEC	0
7A478	Emergency bypass of femoral artery by anastomosis of femoral artery to peroneal artery using prosthesis NEC	0
7A479	Emergency bypass of popliteal artery by anastomosis of popliteal artery to peroneal artery using prosthesis NEC	0
7A47A	Emergency bypass of femoral artery by anastomosis of femoral artery to peroneal artery using vein graft NEC	0
7A47B	Emergency bypass of popliteal artery by anastomosis of popliteal artery to peroneal artery using vein graft NEC	0
7A47C	Emergency bypass of femoral artery by anastomosis of femoral artery to femoral artery NEC	0
7A47D	Emergency bypass of popliteal artery by anastomosis of popliteal artery to femoral artery NEC	0
7A47y	Other specified other emergency bypass of femoral artery or popliteal artery	0
7A47z	Other emergency bypass of femoral artery or popliteal artery NOS	0
7A48.	Other bypass of femoral artery or popliteal artery	0
7A480	Bypass of femoral artery by anastomosis of femoral artery to popliteal artery using prosthesis NEC	0
7A481	Bypass of popliteal artery by anastomosis of popliteal artery to popliteal artery using prosthesis NEC	0
7A482	Bypass of femoral artery by anastomosis of femoral artery to popliteal artery using vein graft NEC	0
7A483	Bypass of popliteal artery by anastomosis of popliteal artery to popliteal artery using vein graft NEC	0
7A484	Bypass of femoral artery by anastomosis of femoral artery to tibial artery using prosthesis NEC	0
7A485	Bypass of popliteal artery by anastomosis of popliteal artery to tibial artery using prosthesis NEC	0
7A486	Bypass of femoral artery by anastomosis of femoral artery to tibial artery using vein graft NEC	0
7A487	Bypass of popliteal artery by anastomosis of popliteal artery to tibial artery using vein graft NEC	0
7A488	Bypass of femoral artery by anastomosis of femoral artery to peroneal artery using prosthesis NEC	0
7A489	Bypass of popliteal artery by anastomosis of popliteal artery to peroneal artery using prosthesis NEC	0
7A48A	Bypass of femoral artery by anastomosis of femoral artery to peroneal artery using vein graft NEC	0
7A48B	Bypass of popliteal artery by anastomosis of popliteal artery to peroneal artery using vein graft NEC	0
7A48C	Bypass of femoral artery by anastomosis of femoral artery to femoral artery NEC	0
7A48D	Bypass of popliteal artery by anastomosis of popliteal artery to femoral artery NEC	0
7A48E	Femoro-femoral prosthetic cross over graft	0
7A48y	Other specified other bypass of femoral artery or popliteal artery	0
7A48z	Other bypass of femoral artery or popliteal artery NOS	0
7A49.	Reconstruction of femoral artery or popliteal artery	0
7A490	Endarterectomy of femoral artery and patch repair of femoral artery	0
7A491	Endarterectomy of popliteal artery and patch repair of popliteal artery	0
7A492	Endarterectomy of femoral artery NEC	0
7A493	Endarterectomy of popliteal artery NEC	0
7A494	Profundoplasty of femoral artery and patch repair of deep femoral artery	0
7A495	Profundoplasty of popliteal artery and patch repair of popliteal artery	0
7A496	Profundoplasty of femoral artery NEC	0
7A497	Profundoplasty of popliteal artery NEC	0
7A498	Reconstruction of femoral artery with vein graft	0
7A499	Reconstruction of popliteal artery with vein graft	0
7A49y	Other specified reconstruction of femoral artery or popliteal artery	0
7A49z	Reconstruction of femoral or popliteal artery NOS	0
7A4A.	Other open operations on femoral artery or popliteal artery	0
7A4A0	Repair of femoral artery NEC	0
7A4A1	Repair of popliteal artery NEC	0
7A4A2	Open embolectomy of femoral artery	0
7A4A3	Open embolectomy popliteal artery	0
7A4A4	Ligation of aneurysm of popliteal artery	0
7A4A5	Operation on aneurysm of femoral artery NEC	0
7A4A6	Operation on popliteal artery NEC	0
7A4A7	Repair of femoral artery with temporary silastic shunt	0
7A4A8	Repair of popliteal artery with temporary silastic shunt	0
7A4Ay	Other specified other open operation on femoral artery or popliteal artery	0
7A4Az	Other open operation on femoral artery or popliteal artery NOS	0
7A4B.	Transluminal operations on femoral artery or popliteal artery	0
7A4B0	Percutaneous transluminal angioplasty of femoral artery	0
7A4B1	Percutaneous transluminal angioplasty of popliteal artery	0
7A4B2	Percutaneous transluminal embolectomy of femoral artery	0
7A4B3	Percutaneous transluminal embolectomy of popliteal artery	0
7A4B4	Percutaneous transluminal embolisation of femoral artery	0
7A4B5	Percutaneous transluminal embolisation of popliteal artery	0
7A4B6	Arteriography of femoral artery	0
7A4B7	Arteriography of popliteal artery	0
7A4B8	Percutaneous transluminal thrombolysis of femoral graft using streptokinase	0
7A4B9	Percutaneous transluminal insertion of stent into femoral artery	0
7A4By	Other specified transluminal operation on femoral artery or popliteal artery	0
7A4Bz	Transluminal operation on femoral artery or popliteal artery NOS	0
7A4y.	Other specified operations on iliac and femoral artery	0
7A4z.	Iliac and femoral artery operations NOS	0

7A50.	Revision of reconstruction of artery	0
7A500	Revision of reconstruction involving aorta	0
7A501	Revision of reconstruction involving iliac artery	0
7A502	Revision of reconstruction involving femoral artery	0
7A503	Revision of reconstruction of popliteal artery	0
7A50y	Other specified revision of reconstruction of artery	0
7A50z	Revision of reconstruction of artery NOS	0
C107.	Diabetes mellitus with peripheral circulatory disorder	0
C1070	Diabetes mellitus, juvenile type, with peripheral circulatory disorder	0
C1071	Diabetes mellitus, adult onset, with peripheral circulatory disorder	0
C1072	Diabetes mellitus, adult with gangrene	0
C1073	IDDM with peripheral circulatory disorder	0
C1074	NIDDM with peripheral circulatory disorder	0
C107z	Diabetes mellitus NOS with peripheral circulatory disorder	0
C1086	Insulin dependent diabetes mellitus with gangrene	0
C108G	Insulin dependent diabetes mellitus with peripheral angiopathy	0
C1095	Non-insulin dependent diabetes mellitus with gangrene	0
C109F	Non-insulin-dependent diabetes mellitus with peripheral angiopathy	0
C10E6	Type 1 diabetes mellitus with gangrene	0
C10EG	Type 1 diabetes mellitus with peripheral angiopathy	0
C10F5	Type 2 diabetes mellitus with gangrene	0
C10FF	Type 2 diabetes mellitus with peripheral angiopathy	0
G702.	Extremity artery atheroma	0
G702z	Extremity artery atheroma NOS	0
G73..	Other peripheral vascular disease	0
G730.	Raynaud's syndrome	0
G7300	Raynaud's disease	0
G7301	Raynaud's phenomenon	0
G730z	Raynaud's syndrome NOS	0
G731.	Thromboangiitis obliterans	0
G7310	Buerger's disease	0
G7311	Presenile gangrene	0
G731z	Thromboangiitis obliterans NOS	0
G732.	Peripheral gangrene	0
G7320	Gangrene of toe	0
G7321	Gangrene of foot	0
G7322	Gangrene of finger	0
G7323	Gangrene of thumb	0
G7324	Gangrene of hand	0
G733.	Ischaemic foot	0
G734.	Peripheral arterial disease	0
G73y.	Other specified peripheral vascular disease	0
G73y0	Diabetic peripheral angiopathy	0
G73y1	Peripheral angiopathic disease EC NOS	0
G73y2	Acrocyanosis	0
G73y4	Acroparaesthesia - Schultze's type	0
G73y5	Acroparaesthesia - Nothnagel's type	0
G73y6	Acroparaesthesia - unspecified	0
G73y7	Erythrocyanosis	0
G73y8	Erythromelalgia	0
G73yz	Other specified peripheral vascular disease NOS	0
G73z.	Peripheral vascular disease NOS	0
G73z0	Intermittent claudication	0
G73zz	Peripheral vascular disease NOS	0
G742z	Peripheral arterial embolism and thrombosis NOS	0
G830.	Varicose veins of the leg with ulcer	0
G831.	Varicose veins of the leg with eczema	0
G832.	Varicose veins of the leg with ulcer and eczema	0
G833.	Varicose veins of the leg with rupture	0
G835.	Infected varicose ulcer	0
G836.	Varicose vein of leg with phlebitis	0
G837.	Venous ulcer of leg	0
G8y1.	Postphlebitic syndrome	0
G8y3.	Chronic peripheral venous hypertension	0
G8yy0	Chronic venous insufficiency NOS	0
Gyu74	[X]Other specified peripheral vascular diseases	0
Gyu7A	[X]Peripheral angiopathy in diseases classified elsewhere	0
M271.	Non-pressure ulcer lower limb	0
M2710	Ischaemic ulcer diabetic foot	0
P76..	Other peripheral vascular system anomalies	0
R0542	[D]Gangrene of toe in diabetic	0
R0543	[D]Widespread diabetic foot gangrene	0

CONCEPT: Renal transplant

READ_CODE	DESCRIPTION_SAIL	SENSITIVE_CODE
7B00.	Transplantation of kidney	0
7B000	Autotransplant of kidney	0
7B001	Transplantation of kidney from live donor	0
7B002	Transplantation of kidney from cadaver	0
7B003	Allotransplantation of kidney from cadaver, heart-beating	0
7B004	Allotransplantation of kidney from cadaver, heart non-beating	0
7B005	Allotransplantation of kidney from cadaver NEC	0
7B006	Xenograft renal transplant	0
7B00y	Other specified transplantation of kidney	0
7B00z	Transplantation of kidney NOS	0
7B015	Transplant nephrectomy	0
7B019	Excision of rejected transplanted kidney	0
7B063	Exploration of renal transplant	0
7B0F.	Interventions associated with transplantation of kidney	0
7B0F3	Post-transplantation of kidney examination, recipient	0
7B0F4	Post-transplantation of kidney examination, live donor	0
7B0Fy	Other specified interventions associated with transplantation of kidney	0
7B0Fz	Interventions associated with transplantation of kidney NOS	0
7L020	Autotransplantation of adrenal medulla to caudate nucleus of brain	0
K0B5.	Renal tubulo-interstitial disorders in transplant rejection	0
Kyu1C	[X]Renal tubulo-interstitial disorders in transplant rejection	0
SP081	Transplanted organ rejection	0
SP083	Kidney transplant failure and rejection	0
SP08C	Accelerated rejection of renal transplant	0
SP08D	Acute-on-chronic rejection of renal transplant	0
SP08E	Acute rejection of renal transplant - grade I	0
SP08F	Acute rejection of renal transplant - grade II	0

SP08G	Acute rejection of renal transplant - grade III	0
SP08H	Acute rejection of renal transplant	0
SP08J	Chronic rejection of renal transplant	0
SP08K	Chronic rejection of renal transplant - grade 1	0
SP08L	Chronic rejection of renal transplant - grade II	0
SP08M	Chronic rejection of renal transplant - grade III	0
SP08N	Unexplained episode of renal transplant dysfunction	0
SP08P	Stenosis of vein of transplanted kidney	0
SP08Q	Aneurysm of artery of transplanted kidney	0
SP08R	Renal transplant rejection	0
SP08S	Aneurysm of vein of transplanted kidney	0
SP08T	Urological complication of renal transplant	0
SP08V	Very mild acute rejection of renal transplant	0
SP08W	Vascular complication of renal transplant	0
SP08X	Rupture of artery of transplanted kidney	0
SP08Y	Rupture of vein of transplanted kidney	0
SP08Z	Thrombosis of artery of transplanted kidney	0
SP08a	Thrombosis of vein of transplanted kidney	0
TB001	Transplantation of kidney as the cause of abnormal reaction of patient, or of later complication, without mention of misadventure at the time of operation	0
ZV420	[V]Kidney transplanted	0

CONCEPT: Rheumatoid Arthritis

READ_CODE	DESCRIPTION_SAIL	SENSITIVE_CODE
14G1.	H/O: rheumatoid arthritis	0
38DZ.	Disease activity score in rheumatoid arthritis	0
38DZ0	Disease activity score 28 joint in rheumatoid arthritis	0
66HB0	Rheumatoid arthritis annual review	0
7P203	Delivery of rehabilitation for rheumatoid arthritis	0
9hR..	Exception reporting: rheumatoid arthritis quality indicators	0
9hR0.	Excepted from rheumatoid arthritis quality indicators: patient unsuitable	0
9hR1.	Excepted from rheumatoid arthritis quality indicators: informed dissent	0
9mM..	Rheumatoid arthritis monitoring invitation	0
9mM0.	Rheumatoid arthritis monitoring invitation first letter	0
9mM1.	Rheumatoid arthritis monitoring invitation second letter	0
9mM2.	Rheumatoid arthritis monitoring invitation third letter	0
9mM3.	Rheumatoid arthritis monitoring verbal invitation	0
9mM4.	Rheumatoid arthritis monitoring telephone invitation	0
F3712	Polyneuropathy in rheumatoid arthritis	0
F3964	Myopathy due to rheumatoid arthritis	0
H570.	Rheumatoid lung	0
N04..	Rheumatoid arthritis and other inflammatory polyarthropathies	0
N040.	Rheumatoid arthritis	0
N0400	Rheumatoid arthritis of cervical spine	0
N0401	Other rheumatoid arthritis of spine	0
N0402	Rheumatoid arthritis of shoulder	0
N0403	Rheumatoid arthritis of sternoclavicular joint	0
N0404	Rheumatoid arthritis of acromioclavicular joint	0
N0405	Rheumatoid arthritis of elbow	0
N0406	Rheumatoid arthritis of distal radio-ulnar joint	0
N0407	Rheumatoid arthritis of wrist	0
N0408	Rheumatoid arthritis of metacarpophalangeal joint	0
N0409	Rheumatoid arthritis of proximal interphalangeal joint of finger	0
N040A	Rheumatoid arthritis of distal interphalangeal joint of finger	0
N040B	Rheumatoid arthritis of hip	0
N040C	Rheumatoid arthritis of sacro-iliac joint	0
N040D	Rheumatoid arthritis of knee	0
N040E	Rheumatoid arthritis of tibio-fibular joint	0
N040F	Rheumatoid arthritis of ankle	0
N040G	Rheumatoid arthritis of subtalar joint	0
N040H	Rheumatoid arthritis of talonavicular joint	0
N040J	Rheumatoid arthritis of other tarsal joint	0
N040K	Rheumatoid arthritis of 1st metatarsophalangeal joint	0
N040L	Rheumatoid arthritis of lesser metatarsophalangeal joint	0
N040M	Rheumatoid arthritis of interphalangeal joint of toe	0
N040N	Rheumatoid vasculitis	0
N040P	Seronegative rheumatoid arthritis	0
N040Q	Rheumatoid bursitis	0
N040R	Rheumatoid nodule	0
N040S	Rheumatoid arthritis - multiple joint	0
N040T	Flare of rheumatoid arthritis	0
N041.	Felty's syndrome	0
N0421	Rheumatoid lung disease	0
N0422	Rheumatoid nodule	0
N047.	Seropositive errosive rheumatoid arthritis	0
N04X.	Seropositive rheumatoid arthritis, unspecified	0
N04y0	Rheumatoid lung	0
Nyu10	[X]Rheumatoid arthritis with involvement of other organs or systems	0
Nyu11	[X]Other seropositive rheumatoid arthritis	0
Nyu12	[X]Other specified rheumatoid arthritis	0
Nyu1G	[X]Seropositive rheumatoid arthritis, unspecified	0

CONCEPT: Schizophrenia

READ_CODE	DESCRIPTION_SAIL	SENSITIVE_CODE
1284.	FH: Schizophrenia	0
13L3.	Relative - psychotic illness	0
13Y2.	Schizophrenia association member	0
1464.	H/O: schizophrenia	0
212T.	Psychosis, schizophrenia and bipolar affective disorder resolved	0
212W.	Schizophrenia resolved	0
E10..	Schizophrenic disorders	0
E100.	Simple schizophrenia	0
E1000	Unspecified schizophrenia	0
E1001	Subchronic schizophrenia	0
E1002	Chronic schizophrenic	0
E1003	Acute exacerbation of subchronic schizophrenia	0
E1004	Acute exacerbation of chronic schizophrenia	0
E1005	Schizophrenia in remission	0
E100z	Simple schizophrenia NOS	0
E101.	Hebephrenic schizophrenia	0
E1010	Unspecified hebephrenic schizophrenia	0
E1011	Subchronic hebephrenic schizophrenia	0

E1012	Chronic hebephrenic schizophrenia	0
E1013	Acute exacerbation of subchronic hebephrenic schizophrenia	0
E1014	Acute exacerbation of chronic hebephrenic schizophrenia	0
E1015	Hebephrenic schizophrenia in remission	0
E101z	Hebephrenic schizophrenia NOS	0
E102.	Catatonic schizophrenia	0
E1020	Unspecified catatonic schizophrenia	0
E1021	Subchronic catatonic schizophrenia	0
E1022	Chronic catatonic schizophrenia	0
E1023	Acute exacerbation of subchronic catatonic schizophrenia	0
E1024	Acute exacerbation of chronic catatonic schizophrenia	0
E1025	Catatonic schizophrenia in remission	0
E102z	Catatonic schizophrenia NOS	0
E103.	Paranoid schizophrenia	0
E1030	Unspecified paranoid schizophrenia	0
E1031	Subchronic paranoid schizophrenia	0
E1032	Chronic paranoid schizophrenia	0
E1033	Acute exacerbation of subchronic paranoid schizophrenia	0
E1034	Acute exacerbation of chronic paranoid schizophrenia	0
E1035	Paranoid schizophrenia in remission	0
E103z	Paranoid schizophrenia NOS	0
E104.	Acute schizophrenic episode	0
E105.	Latent schizophrenia	0
E1050	Unspecified latent schizophrenia	0
E1051	Subchronic latent schizophrenia	0
E1052	Chronic latent schizophrenia	0
E1053	Acute exacerbation of subchronic latent schizophrenia	0
E1054	Acute exacerbation of chronic latent schizophrenia	0
E1055	Latent schizophrenia in remission	0
E105z	Latent schizophrenia NOS	0
E106.	Residual schizophrenia	0
E107.	Schizo-affective schizophrenia	0
E1070	Unspecified schizo-affective schizophrenia	0
E1071	Subchronic schizo-affective schizophrenia	0
E1072	Chronic schizo-affective schizophrenia	0
E1073	Acute exacerbation of subchronic schizo-affective schizophrenia	0
E1074	Acute exacerbation of chronic schizo-affective schizophrenia	0
E1075	Schizo-affective schizophrenia in remission	0
E107z	Schizo-affective schizophrenia NOS	0
E10y.	Other schizophrenia	0
E10y0	Atypical schizophrenia	0
E10y1	Coenesthopathic schizophrenia	0
E10yz	Other schizophrenia NOS	0
E10z.	Schizophrenia NOS	0
E14z.	Child psychosis NOS	0
Eu052	[X]Organic delusional [schizophrenia-like] disorder	0
Eu2..	[X]Schizophrenia, schizotypal and delusional disorders	0
Eu20.	[X]Schizophrenia	0
Eu200	[X]Paranoid schizophrenia	0
Eu201	[X]Hebephrenic schizophrenia	0
Eu202	[X]Catatonic schizophrenia	0
Eu203	[X]Undifferentiated schizophrenia	0
Eu204	[X]Post-schizophrenic depression	0
Eu205	[X]Residual schizophrenia	0
Eu206	[X]Simple schizophrenia	0
Eu20y	[X]Other schizophrenia	0
Eu20z	[X]Schizophrenia, unspecified	0
Eu21.	[X]Schizotypal disorder	0
Eu231	[X]Acute polymorphic psychotic disorder with symptoms of schizophrenia	0
Eu232	[X]Acute schizophrenia-like psychotic disorder	0
Eu252	[X]Schizoaffective disorder, mixed type	0
ZV110	[V]Personal history of schizophrenia	0

CONCEPT: Skin Cancer

READ_CODE	DESCRIPTION_SAIL	SENSITIVE_CODE
14250	H/O Malignant melanoma	0
4M70.	Clark melanoma level 1	0
4M71.	Clark melanoma level 2	0
4M72.	Clark melanoma level 3	0
4M73.	Clark melanoma level 4	0
4M74.	Clark melanoma level 5	0
7G03J	Excision of melanoma	0
7G03K	Excision malignant skin tumour	0
7G056	Excision biopsy of rodent ulcer	0
B3...	Malignant neoplasm of bone, connective tissue, skin and breast	0
B3104	Malignant neoplasm of tarsus of eyelid	0
B32..	Malignant melanoma of skin	0
B320.	Malignant melanoma of lip	0
B321.	Malignant melanoma of eyelid including canthus	0
B322.	Malignant melanoma of ear and external auricular canal	0
B3220	Malignant melanoma of auricle (ear)	0
B3221	Malignant melanoma of external auditory meatus	0
B322z	Malignant melanoma of ear and external auricular canal NOS	0
B323.	Malignant melanoma of other and unspecified parts of face	0
B3230	Malignant melanoma of external surface of cheek	0
B3231	Malignant melanoma of chin	0
B3232	Malignant melanoma of eyebrow	0
B3233	Malignant melanoma of forehead	0
B3234	Malignant melanoma of external surface of nose	0
B3235	Malignant melanoma of temple	0
B323z	Malignant melanoma of face NOS	0
B324.	Malignant melanoma of scalp and neck	0
B3240	Malignant melanoma of scalp	0
B3241	Malignant melanoma of neck	0
B324z	Malignant melanoma of scalp and neck NOS	0
B325.	Malignant melanoma of trunk (excluding scrotum)	0
B3250	Malignant melanoma of axilla	0
B3251	Malignant melanoma of breast	0
B3252	Malignant melanoma of buttock	0
B3253	Malignant melanoma of groin	0
B3254	Malignant melanoma of perianal skin	0
B3255	Malignant melanoma of perineum	0

B3256	Malignant melanoma of umbilicus	0
B3257	Malignant melanoma of back	0
B3258	Malignant melanoma of chest wall	0
B325z	Malignant melanoma of trunk, excluding scrotum, NOS	0
B326.	Malignant melanoma of upper limb and shoulder	0
B3260	Malignant melanoma of shoulder	0
B3261	Malignant melanoma of upper arm	0
B3262	Malignant melanoma of fore-arm	0
B3263	Malignant melanoma of hand	0
B3264	Malignant melanoma of finger	0
B3265	Malignant melanoma of thumb	0
B326z	Malignant melanoma of upper limb or shoulder NOS	0
B327.	Malignant melanoma of lower limb and hip	0
B3270	Malignant melanoma of hip	0
B3271	Malignant melanoma of thigh	0
B3272	Malignant melanoma of knee	0
B3273	Malignant melanoma of popliteal fossa area	0
B3274	Malignant melanoma of lower leg	0
B3275	Malignant melanoma of ankle	0
B3276	Malignant melanoma of heel	0
B3277	Malignant melanoma of foot	0
B3278	Malignant melanoma of toe	0
B3279	Malignant melanoma of great toe	0
B327z	Malignant melanoma of lower limb or hip NOS	0
B328.	Malignant melanoma stage IA	0
B329.	Malignant melanoma stage IB	0
B32A.	Malignant melanoma stage IIA	0
B32B.	Malignant melanoma stage IIB	0
B32C.	Malignant melanoma stage IIC	0
B32D.	Malignant melanoma stage IIIA	0
B32E.	Malignant melanoma stage IIIB	0
B32F.	Malignant melanoma stage IIIC	0
B32G.	Malignant melanoma stage IV M1a	0
B32H.	Malignant melanoma stage IV M1b	0
B32J.	Malignant melanoma stage IV M1c	0
B32y.	Malignant melanoma of other specified skin site	0
B32y0	Overlapping malignant melanoma of skin	0
B32z.	Malignant melanoma of skin NOS	0
B33..	Other malignant neoplasm of skin	0
B330.	Malignant neoplasm of skin of lip	0
B331.	Malignant neoplasm of eyelid including canthus	0
B3311	Malignant neoplasm of upper eyelid	0
B3312	Malignant neoplasm of lower eyelid	0
B332.	Malignant neoplasm of skin of ear and external auricular canal	0
B3320	Malignant neoplasm of skin of auricle (ear)	0
B3321	Malignant neoplasm of skin of external auditory meatus	0
B3322	Malignant neoplasm of pinna NEC	0
B332z	Malignant neoplasm of skin of ear and external auricular canal NOS	0
B333.	Malignant neoplasm of skin of other and unspecified parts of face	0
B3330	Malignant neoplasm of skin of cheek, external	0
B3331	Malignant neoplasm of skin of chin	0
B3332	Malignant neoplasm of skin of eyebrow	0
B3333	Malignant neoplasm of skin of forehead	0
B3334	Malignant neoplasm of skin of nose (external)	0
B3335	Malignant neoplasm of skin of temple	0
B333z	Malignant neoplasm of skin of other and unspecified part of face NOS	0
B334.	Malignant neoplasm of scalp and skin of neck	0
B3340	Malignant neoplasm of scalp	0
B3341	Malignant neoplasm of skin of neck	0
B334z	Malignant neoplasm of scalp or skin of neck NOS	0
B335.	Malignant neoplasm of skin of trunk, excluding scrotum	0
B3350	Malignant neoplasm of skin of axillary fold	0
B3351	Malignant neoplasm of skin of chest, excluding breast	0
B3352	Malignant neoplasm of skin of breast	0
B3353	Malignant neoplasm of skin of abdominal wall	0
B3354	Malignant neoplasm of skin of umbilicus	0
B3355	Malignant neoplasm of skin of groin	0
B3356	Malignant neoplasm of skin of perineum	0
B3357	Malignant neoplasm of skin of back	0
B3358	Malignant neoplasm of skin of buttock	0
B3359	Malignant neoplasm of perianal skin	0
B335A	Malignant neoplasm of skin of scapular region	0
B335z	Malignant neoplasm of skin of trunk, excluding scrotum, NOS	0
B336.	Malignant neoplasm of skin of upper limb and shoulder	0
B3360	Malignant neoplasm of skin of shoulder	0
B3361	Malignant neoplasm of skin of upper arm	0
B3362	Malignant neoplasm of skin of fore-arm	0
B3363	Malignant neoplasm of skin of hand	0
B3364	Malignant neoplasm of skin of finger	0
B3365	Malignant neoplasm of skin of thumb	0
B336z	Malignant neoplasm of skin of upper limb or shoulder NOS	0
B337.	Malignant neoplasm of skin of lower limb and hip	0
B3370	Malignant neoplasm of skin of hip	0
B3371	Malignant neoplasm of skin of thigh	0
B3372	Malignant neoplasm of skin of knee	0
B3373	Malignant neoplasm of skin of popliteal fossa area	0
B3374	Malignant neoplasm of skin of lower leg	0
B3375	Malignant neoplasm of skin of ankle	0
B3376	Malignant neoplasm of skin of heel	0
B3377	Malignant neoplasm of skin of foot	0
B3378	Malignant neoplasm of skin of toe	0
B3379	Malignant neoplasm of skin of great toe	0
B337z	Malignant neoplasm of skin of lower limb or hip NOS	0
B338.	Squamous cell carcinoma of skin	0
B339.	Dermatofibrosarcoma protuberans	0
B33X.	Malignant neoplasm overlapping lesion of skin	0
B33y.	Malignant neoplasm of other specified skin sites	0
B33z.	Malignant neoplasm of skin NOS	0
B33z0	Kaposi's sarcoma of skin	0
B3y..	Malignant neoplasm of bone, connective tissue, skin and breast otherwise specified	0
B3z..	Malignant neoplasm of bone, connective tissue, skin and breast NOS	0
B480.	Malignant neoplasm of prepuce (foreskin)	0
B509.	Malignant melanoma of eye	0

B582.	Secondary malignant neoplasm of skin	0
B5820	Secondary malignant neoplasm of skin of head	0
B5821	Secondary malignant neoplasm of skin of face	0
B5822	Secondary malignant neoplasm of skin of neck	0
B5823	Secondary malignant neoplasm of skin of trunk	0
B5824	Secondary malignant neoplasm of skin of shoulder and arm	0
B5825	Secondary malignant neoplasm of skin of hip and leg	0
B5826	Secondary malignant neoplasm of skin of breast	0
B582z	Secondary malignant neoplasm of skin NOS	0
B6286	Cutaneous follicle centre lymphoma	0
B62E3	Cutaneous T-cell lymphoma	0
B62xX	Other and unspecified peripheral and cutaneous T-cell lymphomas	0
B932.	Neoplasm of uncertain behaviour of skin	0
BA02.	Neoplasm of unspecified nature of bone, skin and soft tissue	0
BA022	Neoplasm of unspecified nature of skin	0
BA02z	Neoplasm of unspecified nature of bone, skin or soft tissue NOS	0
BB2..	[M]Papillary and squamous cell neoplasms	0
BB2A.	[M]Squamous cell carcinoma NOS	0
BB36.	[M]Metatypical carcinoma	0
BB5RA	[M]Merkel cell carcinoma	0
BB6..	[M]Adnexal and skin appendage neoplasms	0
BB60.	[M]Skin appendage adenoma and carcinoma	0
BB601	[M]Skin appendage carcinoma	0
BB60z	[M]Skin appendage adenoma or carcinoma NOS	0
BB611	[M]Sweat gland tumour NOS	0
BB62.	[M]Apocrine adenoma and adenocarcinomas	0
BB621	[M]Apocrine adenocarcinoma	0
BB62z	[M]Apocrine adenoma or adenocarcinoma NOS	0
BB69.	[M]Sebaceous adenoma and adenocarcinoma	0
BB691	[M]Sebaceous adenocarcinoma	0
BB69z	[M]Sebaceous adenoma or adenocarcinoma NOS	0
BB6z.	[M]Adnexal and skin appendage neoplasm NOS	0
BBB5.	[M]Adenocarcinoma with apocrine metaplasia	0
BBE..	[M]Naevi and melanomas	0
BBE1.	[M]Malignant melanoma NOS	0
BBE10	[M]Malignant melanoma, regressing	0
BBE11	[M]Desmoplastic melanoma, malignant	0
BBE2.	[M]Nodular melanoma	0
BBE4.	[M]Balloon cell melanoma	0
BBEA.	[M]Amelanotic melanoma	0
BBEC.	[M]Malignant melanoma in junctional naevus	0
BBEE.	[M]Malignant melanoma in precancerous melanosis	0
BBEG.	[M]Malignant melanoma in Hutchinson's melanotic freckle	0
BBEG0	[M]Acral lentiginous melanoma, malignant	0
BBEH.	[M]Superficial spreading melanoma	0
BBEM.	[M]Malignant melanoma in giant pigmented naevus	0
BBEN.	[M]Epithelioid and spindle cell naevus	0
BBEP.	[M]Epithelioid cell melanoma	0
BBEQ.	[M]Spindle cell melanoma NOS	0
BBER.	[M]Spindle cell melanoma, type A	0
BBES.	[M]Spindle cell melanoma, type B	0
BBET.	[M]Mixed epithelioid and spindle melanoma	0
BBEz.	[M]Naevi or melanoma NOS	0
BBGM.	[M]Dermatofibrosarcoma NOS	0
BBGP.	[M]Pigmented dermatofibrosarcoma protuberans	0
BBmD.	[M] Cutaneous lymphoma	0
Byu4.	[X]Melanoma and other malignant neoplasms of skin	0
Byu40	[X]Malignant melanoma of other and unspecified parts of face	0
Byu41	[X]Malignant melanoma of skin, unspecified	0
Byu42	[X]other malignant neoplasm of skin of other and unspecified parts of face	0
Byu43	[X]Malignant neoplasm of skin, unspecified	0
Byu5A	[X]Malignant neoplasm overlapping lesion of skin	0
ByuDD	[X]Other and unspecified peripheral and cutaneous T-cell lymphomas	0
ZV10y	[V]Personal history of other specified malignant neoplasm	0

CONCEPT: Stroke

READ_CODE	DESCRIPTION_SAIL	SENSITIVE_CODE
14A7.	H/O: CVA/stroke	0
14AB.	H/O: TIA	0
14AK.	H/O: Stroke in last year	0
1M4..	Central post-stroke pain	0
662M.	Stroke monitoring	0
662M1	Stroke 6 month review	0
662e.	Stroke/CVA annual review	0
662o.	Haemorrhagic stroke monitoring	0
70041	Evacuation of haematoma from temporal lobe of brain	0
70042	Evacuation of haematoma from cerebellum	0
70043	Evacuation of intracerebral haematoma NEC	0
70082	Aspiration of haematoma of brain tissue	0
7P242	Delivery of rehabilitation for stroke	0
8HBJ.	Stroke / transient ischaemic attack referral	0
8Hd6.	Admission to stroke unit	0
9N0p.	Seen in stroke clinic	0
Fyu55	[X]Other transient cerebral ischaemic attacks and related syndromes	0
G6...	Cerebrovascular disease	0
G60..	Subarachnoid haemorrhage	0
G600.	Ruptured berry aneurysm	0
G601.	Subarachnoid haemorrhage from carotid siphon and bifurcation	0
G602.	Subarachnoid haemorrhage from middle cerebral artery	0
G603.	Subarachnoid haemorrhage from anterior communicating artery	0
G604.	Subarachnoid haemorrhage from posterior communicating artery	0
G605.	Subarachnoid haemorrhage from basilar artery	0
G606.	Subarachnoid haemorrhage from vertebral artery	0
G60X.	Subarachnoid haemorrhage from intracranial artery, unspecified	0
G60z.	Subarachnoid haemorrhage NOS	0
G61..	Intracerebral haemorrhage	0
G610.	Cortical haemorrhage	0
G611.	Internal capsule haemorrhage	0
G612.	Basal nucleus haemorrhage	0
G613.	Cerebellar haemorrhage	0
G614.	Pontine haemorrhage	0
G615.	Bulbar haemorrhage	0

G616.	External capsule haemorrhage	0
G617.	Intracerebral haemorrhage, intraventricular	0
G618.	Intracerebral haemorrhage, multiple localized	0
G619.	Lobar cerebral haemorrhage	0
G61X.	Intracerebral haemorrhage in hemisphere, unspecified	0
G61X0	Left sided intracerebral haemorrhage, unspecified	0
G61X1	Right sided intracerebral haemorrhage, unspecified	0
G61z.	Intracerebral haemorrhage NOS	0
G62..	Other and unspecified intracranial haemorrhage	0
G620.	Extradural haemorrhage - nontraumatic	0
G621.	Subdural haemorrhage - nontraumatic	0
G622.	Subdural haematoma - nontraumatic	0
G623.	Subdural haemorrhage NOS	0
G62z.	Intracranial haemorrhage NOS	0
G63y0	Cerebral infarct due to thrombosis of precerebral arteries	0
G63y1	Cerebral infarction due to embolism of precerebral arteries	0
G64..	Cerebral arterial occlusion	0
G6400	Cerebral infarction due to thrombosis of cerebral arteries	0
G6410	Cerebral infarction due to embolism of cerebral arteries	0
G64z.	Cerebral infarction NOS	0
G64z0	Brainstem infarction	0
G64z2	Left sided cerebral infarction	0
G64z3	Right sided cerebral infarction	0
G64z4	Infarction of basal ganglia	0
G65..	Transient cerebral ischaemia	0
G650.	Basilar artery syndrome	0
G651.	Vertebral artery syndrome	0
G6510	Vertebro-basilar artery syndrome	0
G652.	Subclavian steal syndrome	0
G653.	Carotid artery syndrome hemispheric	0
G654.	Multiple and bilateral precerebral artery syndromes	0
G656.	Vertebrobasilar insufficiency	0
G65y.	Other transient cerebral ischaemia	0
G65z.	Transient cerebral ischaemia NOS	0
G65z0	Impending cerebral ischaemia	0
G65z1	Intermittent cerebral ischaemia	0
G65zz	Transient cerebral ischaemia NOS	0
G66..	Stroke and cerebrovascular accident unspecified	0
G663.	Brain stem stroke syndrome	0
G664.	Cerebellar stroke syndrome	0
G667.	Left sided CVA	0
G668.	Right sided CVA	0
G669.	Cerebral palsy, not congenital or infantile, acute	0
G6760	Cerebral infarction due to cerebral venous thrombosis, nonpyogenic	0
G680.	Sequelae of subarachnoid haemorrhage	0
G681.	Sequelae of intracerebral haemorrhage	0
G6W..	Cerebral infarction due to unspecified occlusion or stenosis of precerebral arteries	0
G6X..	Cerebral infarction due to unspecified occlusion or stenosis of cerebral arteries	0
Gyu60	[X]Subarachnoid haemorrhage from other intracranial arteries	0
Gyu61	[X]Other subarachnoid haemorrhage	0
Gyu62	[X]Other intracerebral haemorrhage	0
Gyu63	[X]Cerebral infarction due to unspecified occlusion or stenosis of cerebral arteries	0
Gyu64	[X]Other cerebral infarction	0
Gyu65	[X]Occlusion and stenosis of other precerebral arteries	0
Gyu66	[X]Occlusion and stenosis of other cerebral arteries	0
Gyu6E	[X]Subarachnoid haemorrhage from intracranial artery, unspecified	0
Gyu6F	[X]Intracerebral haemorrhage in hemisphere, unspecified	0
Gyu6G	[X]Cerebral infarction due to unspecified occlusion or stenosis of precerebral arteries	0
Q200.	Subdural and cerebral haemorrhage due to birth trauma	0
Q2000	Cerebral haemorrhage unspecified, due to birth trauma	0
ZV125	[V]Personal history of circulatory system disease	0
ZV12D	[V]Personal history of transient ischaemic attack	0

CONCEPT: Systemic Lupus Erythematosus

READ_CODE	DESCRIPTION_SAIL	SENSITIVE_CODE
F3710	Polyneuropathy in disseminated lupus erythematosus	0
F3961	Myopathy due to disseminated lupus erythematosus	0
H57y4	Lung disease with systemic lupus erythematosus	0
K01x4	Nephrotic syndrome in systemic lupus erythematosus	0
K0B40	Renal tubulo-interstitial disorder in systemic lupus erythematosus	0
M154.	Lupus erythematosus	0
M1540	Lupus erythematosus chronicus	0
M1542	Lupus erythematosus migrans	0
M1543	Lupus erythematosus nodularis	0
M1544	Lupus erythematosus profundus	0
M1545	Lupus erythematosus tumidus	0
M1546	Lupus erythematosus unguium mutilans	0
M1547	Subacute cutaneous lupus erythematosus	0
M154z	Lupus erythematosus NOS	0
Myu78	[X]Other local lupus erythematosus	0
N000.	Systemic lupus erythematosus	0
N0000	Disseminated lupus erythematosus	0
N0001	Libman-Sacks disease	0
N0002	Drug-induced systemic lupus erythematosus	0
N0003	Systemic lupus erythematosus with organ or system involvement	0
N0004	Systemic lupus erythematosus with pericarditis	0
N0006	Cerebral lupus	0
N000z	Systemic lupus erythematosus NOS	0
Nyu43	[X]Other forms of systemic lupus erythematosus	0

CONCEPT: Systemic sclerosis

READ_CODE	DESCRIPTION_SAIL	SENSITIVE_CODE
F3966	Myopathy due to scleroderma	0
H572.	Lung disease with systemic sclerosis	0
K0H..	Acute scleroderma renal crisis	0
K0J0.	Renal involvement in scleroderma	0
M210.	Circumscribed scleroderma	0
M2100	Unspecified circumscribed scleroderma	0
M2104	Linear scleroderma	0
M210z	Circumscribed scleroderma NOS	0
N001.	Scleroderma	0

N0010	Progressive systemic sclerosis	0
N0011	CREST syndrome	0
N0012	Systemic sclerosis induced by drugs and chemicals	0
Nyu45	[X]Other forms of systemic sclerosis	0
Q4711	Scleroderma in newborn	0

CONCEPT: Thalassaemia

READ_CODE	DESCRIPTION_SAIL	SENSITIVE_CODE
D104.	Thalassaemia	0
D1040	Thalassaemia major NEC	0
D1042	Thalassaemia with haemoglobin S disease	0
D1043	Alpha thalassaemia	0
D1046	Beta intermedia thalassaemia	0
D1047	Beta major thalassaemia	0
D1048	Beta minor thalassaemia	0
D1049	Delta-beta thalassaemia	0
D104z	Thalassaemia NOS	0
Dyu11	[X]Other thalassaemias	0

CONCEPT: TIA (Transient ischaemic attack)

READ_CODE	DESCRIPTION_SAIL	SENSITIVE_CODE
14AB.	H/O: TIA	0
14AB0	H/O amaurosis fugax	0
8CRB.	Transient ischaemic attack clinical management plan	0
F4236	Amaurosis fugax	0
Fyu55	[X]Other transient cerebral ischaemic attacks and related syndromes	0
G65..	Transient cerebral ischaemia	0
G657.	Carotid territory transient ischaemic attack	0
G65y.	Other transient cerebral ischaemia	0
G65z.	Transient cerebral ischaemia NOS	0
G65z0	Impending cerebral ischaemia	0
G65z1	Intermittent cerebral ischaemia	0
G65zz	Transient cerebral ischaemia NOS	0
R0027	[D]Drop attack	0
ZV12D	[V]Personal history of transient ischaemic attack	0

CONCEPT: Viral Hepatitis

READ_CODE	DESCRIPTION_SAIL	SENSITIVE_CODE
141E.	History of hepatitis B	1
14i..	H/O hepatitis C antiviral drug therapy	1
21267	Hepatitis C resolved	1
2J12.	Hepatitis C non immune	1
43B4.	Hepatitis B surface antig +ve	1
43B6.	Hepatitis B non immune	1
43B7.	Hepatitis C non-immune	1
43X3.	Hepatitis C antibody test positive	1
4JQ3.	Hepatitis C virus genotype	1
4JQD.	Hepatitis C viral ribonucleic acid polymerase chain reaction positive	1
4JQF.	Hepatitis C antigen positive	1
65Q7.	Viral hepatitis carrier	1
7Q052	Hepatitis B treatment drugs Band 1	1
7Q053	Respiratory syncytial virus treatment and Hepatitis C treatment drugs Band 1	1
8BB5.	12 week virologic response to hepatitis C treatment	1
9NgR.	On hepatitis C treatment plan	1
9kV..	Hepatitis C screening positive - enhanced services administration	1
9kZ..	Hepatitis B screening positive - enhanced services administration	1
A702.	Viral hepatitis B with coma	1
A7020	Acute hepatitis B with delta agent (coinfection) with hepatic coma	1
A703.	Viral (serum) hepatitis B	1
A7030	Acute hepatitis B with delta-agent (coinfection) without hepatic coma	1
A7040	Viral hepatitis C with coma	1
A7050	Viral hepatitis C without mention of hepatic coma	1
A7051	Acute delta-(super)infection of hepatitis B carrier	1
A7054	Hepatitis non A non B	1
A707.	Chronic viral hepatitis	1
A7070	Chronic viral hepatitis B with delta-agent	1
A7071	Chronic viral hepatitis B without delta-agent	1
A7072	Chronic viral hepatitis C	1
A7073	Chronic viral hepatitis B	1
A707X	Chronic viral hepatitis, unspecified	1
A70A.	Hepatitis C genotype 1	1
A70B.	Hepatitis C genotype 2	1
A70C.	Hepatitis C genotype 3	1
A70D.	Hepatitis C genotype 4	1
A70E.	Hepatitis C genotype 5	1
A70F.	Hepatitis C genotype 6	1
A70G.	Acute hepatitis C	1
A70z0	Hepatitis C	1
AE23.	Sequelae of viral hepatitis	1
AyuB1	[X]Other chronic viral hepatitis	1
AyuB2	[X]Chronic viral hepatitis, unspecified	1
AyuJ9	[X]Sequelae of viral hepatitis	1
Q409.	Congenital viral hepatitis	1
Q4091	Congenital hepatitis B infection	1
Q409y	Other specified congenital viral hepatitis	1
Q409z	Congenital viral hepatitis NOS	1
ZV026	[V]Viral hepatitis carrier	1
ZV02B	[V]Hepatitis B carrier	1
ZV02C	[V]Hepatitis C carrier	1

CONCEPT: VTD (Venous thrombotic disease)

READ_CODE	DESCRIPTION_SAIL	SENSITIVE_CODE
14A81	H/O: Deep Vein Thrombosis	0
F050.	Embolism of central nervous system venous sinus	0
F0500	Embolism cavernous sinus	0
F0501	Embolism superior longitudinal sinus	0
F0502	Embolism lateral sinus	0
F0503	Embolism transverse sinus	0
F050z	Embolism central nervous system venous sinus NOS	0
F051.	Thrombosis of central nervous system venous sinuses	0

F0510	Thrombosis cavernous sinus	0
F0511	Thrombosis of superior longitudinal sinus	0
F0512	Thrombosis lateral sinus	0
F0513	Thrombosis transverse sinus	0
F051z	Thrombosis of central nervous system venous sinus NOS	0
G676.	Nonpyogenic venous sinus thrombosis	0
G6760	Cerebral infarction due to cerebral venous thrombosis, nonpyogenic	0
G67A.	Cerebral vein thrombosis	0
G800.	Superficial vessel phlebitis and/or thrombophlebitis of the leg	0
G801.	Deep vein phlebitis and thrombophlebitis of the leg	0
G801C	Deep vein thrombosis of leg related to air travel	0
G801D	Deep vein thrombosis of lower limb	0
G801E	Deep vein thrombosis of leg related to intravenous drug use	0
G801F	Deep vein thrombosis of peroneal vein	0
G801G	Recurrent deep vein thrombosis	0
G801H	Unprovoked deep vein thrombosis	0
G801J	Provoked deep vein thrombosis	0
G8020	Thrombosis of vein of leg	0
G81..	Portal vein thrombosis	0
G82..	Other venous embolism and thrombosis	0
G820.	Budd - Chiari syndrome (hepatic vein thrombosis)	0
G822.	Embolism and thrombosis of the vena cava	0
G8220	Thrombosis of inferior vena cava	0
G823.	Embolism and thrombosis of the renal vein	0
G824.	Axillary vein thrombosis	0
G825.	Thrombosis of subclavian vein	0
G826.	Thrombosis of internal jugular vein	0
G827.	Thrombosis of external jugular vein	0
G82z0	Embolus of vein NOS	0
G82z1	Thrombosis of vein NOS	0
Gyu82	[X]Embolism and thrombosis of other specified veins	0
L413.	Antenatal deep vein thrombosis	0
L4130	Antenatal deep vein thrombosis unspecified	0
L4131	Antenatal deep vein thrombosis - delivered	0
L4132	Antenatal deep vein thrombosis with antenatal complication	0
L413z	Antenatal deep vein thrombosis NOS	0
L414.	Postnatal deep vein thrombosis	0
L4140	Postnatal deep vein thrombosis unspecified	0
L4141	Postnatal deep vein thrombosis - delivered with postnatal complication	0
L4142	Postnatal deep vein thrombosis with postnatal complication	0
L414z	Postnatal deep vein thrombosis NOS	0
L417.	Obstetric cerebral venous thrombosis	0
L4170	Cerebral venous thrombosis in pregnancy	0
L4171	Cerebral venous thrombosis in the puerperium	0
SP08a	Thrombosis of vein of transplanted kidney	0
SP122	Post operative deep vein thrombosis	0
ZV128	[V] Personal history deep vein thrombosis	0

ICD10 codes

CONCEPT: Addison's disease

ICD10_CODE	DESCRIPTION_SAIL	SENSITIVE_CODE
A187	Tuberculosis of adrenal glands	0
E271	Primary adrenocortical insufficiency	0
E272	Addisonian crisis	0
E351	Disorders of adrenal glands in diseases classified elsewhere	0
E896	Postprocedural adrenocortical(-medullary) hypofunction	0
G735	Myopathy in endocrine diseases	0

CONCEPT: Anaemia

ICD10_CODE	DESCRIPTION_SAIL	SENSITIVE_CODE
D51	Vitamin B12 deficiency anaemia	0
D510	Vitamin B12 deficiency anaemia due to intrinsic factor deficiency	0
D511	Vitamin B12 deficiency anaemia due to selective vitamin B12 malabsorption with proteinuria	0
D512	Transcobalamin II deficiency	0
D513	Other dietary vitamin B12 deficiency anaemia	0
D518	Other vitamin B12 deficiency anaemias	0
D519	Vitamin B12 deficiency anaemia, unspecified	0

CONCEPT: Aneurysm

ICD10_CODE	DESCRIPTION_SAIL	SENSITIVE_CODE
I71	Aortic aneurysm and dissection	0
I710	Dissection of aorta [any part]	0
I711	Thoracic aortic aneurysm, ruptured	0
I712	Thoracic aortic aneurysm, without mention of rupture	0
I713	Abdominal aortic aneurysm, ruptured	0
I714	Abdominal aortic aneurysm, without mention of rupture	0
I715	Thoracoabdominal aortic aneurysm, ruptured	0
I716	Thoracoabdominal aortic aneurysm, without mention of rupture	0
I718	Aortic aneurysm of unspecified site, ruptured	0
I719	Aortic aneurysm of unspecified site, without mention of rupture	0

CONCEPT: Ankylosing Spondylitis

ICD10_CODE	DESCRIPTION_SAIL	SENSITIVE_CODE
M081	Juvenile ankylosing spondylitis	0
M0810	Juvenile ankylosing spondylitis	0
M0811	Juvenile ankylosing spondylitis	0
M0812	Juvenile ankylosing spondylitis	0
M0813	Juvenile ankylosing spondylitis	0
M0814	Juvenile ankylosing spondylitis	0
M0815	Juvenile ankylosing spondylitis	0
M0816	Juvenile ankylosing spondylitis	0
M0817	Juvenile ankylosing spondylitis	0
M0818	Juvenile ankylosing spondylitis	0
M0819	Juvenile ankylosing spondylitis	0
M45	Ankylosing spondylitis	0
M45X0	Ankylosing spondylitis	0
M45X1	Ankylosing spondylitis	0
M45X2	Ankylosing spondylitis	0

M45X3	Ankylosing spondylitis	0
M45X4	Ankylosing spondylitis	0
M45X5	Ankylosing spondylitis	0
M45X6	Ankylosing spondylitis	0
M45X7	Ankylosing spondylitis	0
M45X8	Ankylosing spondylitis	0
M45X9	Ankylosing spondylitis	0

CONCEPT: Anxiety

ICD10_CODE	DESCRIPTION_SAIL	SENSITIVE_CODE
F064	Organic anxiety disorder	0
F40	Phobic anxiety disorders	0
F400	Agoraphobia	0
F401	Social phobias	0
F402	Specific (isolated) phobias	0
F408	Other phobic anxiety disorders	0
F409	Phobic anxiety disorder, unspecified	0
F41	Other anxiety disorders	0
F410	Panic disorder [episodic paroxysmal anxiety]	0
F411	Generalized anxiety disorder	0
F412	Mixed anxiety and depressive disorder	0
F413	Other mixed anxiety disorders	0
F418	Other specified anxiety disorders	0
F419	Anxiety disorder, unspecified	0
F606	Anxious [avoidant] personality disorder	0
F930	Separation anxiety disorder of childhood	0
F931	Phobic anxiety disorder of childhood	0
F932	Social anxiety disorder of childhood	0

CONCEPT: Arrhythmia

ICD10_CODE	DESCRIPTION_SAIL	SENSITIVE_CODE
I44	Atrioventricular and left bundle-branch block	0
I440	Atrioventricular block, first degree	0
I441	Atrioventricular block, second degree	0
I442	Atrioventricular block, complete	0
I443	Other and unspecified atrioventricular block	0
I447	Left bundle-branch block, unspecified	0
I451	Other and unspecified right bundle-branch block	0
I453	Trifascicular block	0
I455	Other specified heart block	0
I46	Cardiac arrest	0
I460	Cardiac arrest with successful resuscitation	0
I461	Sudden cardiac death, so described	0
I469	Cardiac arrest, unspecified	0
I47	Paroxysmal tachycardia	0
I470	Re-entry ventricular arrhythmia	0
I471	Supraventricular tachycardia	0
I472	Ventricular tachycardia	0
I479	Paroxysmal tachycardia, unspecified	0
I48	Atrial fibrillation and flutter	0
I480	Paroxysmal atrial fibrillation	0
I481	Persistent atrial fibrillation	0
I482	Chronic atrial fibrillation	0
I483	Typical atrial flutter	0
I484	Atypical atrial flutter	0
I489	Atrial fibrillation and atrial flutter, unspecified	0
I49	Other cardiac arrhythmias	0
I490	Ventricular fibrillation and flutter	0
I491	Atrial premature depolarization	0
I492	Junctional premature depolarization	0
I493	Ventricular premature depolarization	0
I494	Other and unspecified premature depolarization	0
I495	Sick sinus syndrome	0
I498	Other specified cardiac arrhythmias	0
I499	Cardiac arrhythmia, unspecified	0
P291	Neonatal cardiac dysrhythmia	0

CONCEPT: Asthma

ICD10_CODE	DESCRIPTION_SAIL	SENSITIVE_CODE
J45	Asthma	0
J450	Predominantly allergic asthma	0
J451	Nonallergic asthma	0
J458	Mixed asthma	0
J459	Asthma, unspecified	0
J46	Status asthmaticus	0

CONCEPT: Atopic Eczema

ICD10_CODE	DESCRIPTION_SAIL	SENSITIVE_CODE
L20	Atopic dermatitis	0
L200	Besnier prurigo	0
L208	Other atopic dermatitis	0
L209	Atopic dermatitis, unspecified	0

CONCEPT: Autism and ADHD

ICD10_CODE	DESCRIPTION_SAIL	SENSITIVE_CODE
F840	Childhood autism	0
F841	Atypical autism	0
F845	Asperger syndrome	0
F900	Disturbance of activity and attention	0

CONCEPT: Bipolar Disorder

ICD10_CODE	DESCRIPTION_SAIL	SENSITIVE_CODE
F30	Manic episode	0
F301	Mania without psychotic symptoms	0
F302	Mania with psychotic symptoms	0
F308	Other manic episodes	0
F309	Manic episode, unspecified	0
F31	Bipolar affective disorder	0
F310	Bipolar affective disorder, current episode hypomanic	0

F311	Bipolar affective disorder, current episode manic without psychotic symptoms	0
F312	Bipolar affective disorder, current episode manic with psychotic symptoms	0
F313	Bipolar affective disorder, current episode mild or moderate depression	0
F314	Bipolar affective disorder, current episode severe depression without psychotic symptoms	0
F315	Bipolar affective disorder, current episode severe depression with psychotic symptoms	0
F316	Bipolar affective disorder, current episode mixed	0
F317	Bipolar affective disorder, currently in remission	0
F318	Other bipolar affective disorders	0
F319	Bipolar affective disorder, unspecified	0
F340	Cyclothymia	0

CONCEPT: Blindness and low vision

ICD10_CODE	DESCRIPTION_SAIL	SENSITIVE_CODE
H540	Blindness, binocular	0
H541	Severe visual impairment, binocular	0

CONCEPT: Breast Cancer

ICD10_CODE	DESCRIPTION_SAIL	SENSITIVE_CODE
C50	Malignant neoplasm of breast	0
C501	Malignant neoplasm: Central portion of breast	0
C502	Malignant neoplasm: Upper-inner quadrant of breast	0
C503	Malignant neoplasm: Lower-inner quadrant of breast	0
C504	Malignant neoplasm: Upper-outer quadrant of breast	0
C505	Malignant neoplasm: Lower-outer quadrant of breast	0
C506	Malignant neoplasm: Axillary tail of breast	0
C508	Malignant neoplasm: Overlapping lesion of breast	0
C509	Malignant neoplasm: Breast, unspecified	0
D05	Carcinoma in situ of breast	0
D057	Other carcinoma in situ of breast	0
D059	Carcinoma in situ of breast, unspecified	0
D24	Benign neoplasm of breast	0
Z853	Personal history of malignant neoplasm of breast	0

CONCEPT: Bronchiectasis

ICD10_CODE	DESCRIPTION_SAIL	SENSITIVE_CODE
J47	Bronchiectasis	0
Q334	Congenital bronchiectasis	0

CONCEPT: Chronic Liver Disease

ICD10_CODE	DESCRIPTION_SAIL	SENSITIVE_CODE
B16	Acute hepatitis B	0
B160	Acute hepatitis B with delta-agent (coinfection) with hepatic coma	0
B161	Acute hepatitis B with delta-agent (coinfection) without hepatic coma	0
B162	Acute hepatitis B without delta-agent with hepatic coma	0
B169	Acute hepatitis B without delta-agent and without hepatic coma	0
B170	Acute delta-(super)infection of hepatitis B carrier	0
B171	Acute hepatitis C	1
B18	Chronic viral hepatitis	0
B180	Chronic viral hepatitis B with delta-agent	0
B181	Chronic viral hepatitis B without delta-agent	0
B182	Chronic viral hepatitis C	0
B188	Other chronic viral hepatitis	0
B189	Chronic viral hepatitis, unspecified	0
I85	Oesophageal varices	0
I850	Oesophageal varices with bleeding	0
I859	Oesophageal varices without bleeding	0
I982	Oesophageal varices without bleeding in diseases classified elsewhere	0
I983	Oesophageal varices with bleeding in diseases classified elsewhere	0
K70	Alcoholic liver disease	0
K700	Alcoholic fatty liver	0
K701	Alcoholic hepatitis	0
K702	Alcoholic fibrosis and sclerosis of liver	0
K703	Alcoholic cirrhosis of liver	0
K704	Alcoholic hepatic failure	0
K709	Alcoholic liver disease, unspecified	0
K713	Toxic liver disease with chronic persistent hepatitis	0
K714	Toxic liver disease with chronic lobular hepatitis	0
K715	Toxic liver disease with chronic active hepatitis	0
K717	Toxic liver disease with fibrosis and cirrhosis of liver	0
K72	Hepatic failure, not elsewhere classified	0
K721	Chronic hepatic failure	0
K729	Hepatic failure, unspecified	0
K73	Chronic hepatitis, not elsewhere classified	0
K730	Chronic persistent hepatitis, not elsewhere classified	0
K731	Chronic lobular hepatitis, not elsewhere classified	0
K732	Chronic active hepatitis, not elsewhere classified	0
K738	Other chronic hepatitis, not elsewhere classified	0
K739	Chronic hepatitis, unspecified	0
K74	Fibrosis and cirrhosis of liver	0
K740	Hepatic fibrosis	0
K742	Hepatic fibrosis with hepatic sclerosis	0
K743	Primary biliary cirrhosis	0
K744	Secondary biliary cirrhosis	0
K745	Biliary cirrhosis, unspecified	0
K746	Other and unspecified cirrhosis of liver	0
K754	Autoimmune hepatitis	0
K766	Portal hypertension	0
K767	Hepatorenal syndrome	0

CONCEPT: Chronic Pain

ICD10_CODE	DESCRIPTION_SAIL	SENSITIVE_CODE
R521	Chronic intractable pain	0
R522	Other chronic pain	0

CONCEPT: Chronic Kidney Disease (CKD) Stage 3_5

ICD10_CODE	DESCRIPTION_SAIL	SENSITIVE_CODE
N183	Chronic kidney disease, stage 3	0
N184	Chronic kidney disease, stage 4	0
N185	Chronic kidney disease, stage 5	0

CONCEPT: Coeliac Disease

ICD10_CODE	DESCRIPTION_SAIL	SENSITIVE_CODE
K900	Coeliac disease	0

CONCEPT: Colon Cancer

ICD10_CODE	DESCRIPTION_SAIL	SENSITIVE_CODE
C18	Malignant neoplasm of colon	0
C180	Malignant neoplasm: Caecum	0
C181	Malignant neoplasm: Appendix	0
C182	Malignant neoplasm: Ascending colon	0
C183	Malignant neoplasm: Hepatic flexure	0
C184	Malignant neoplasm: Transverse colon	0
C185	Malignant neoplasm: Splenic flexure	0
C186	Malignant neoplasm: Descending colon	0
C187	Malignant neoplasm: Sigmoid colon	0
C188	Malignant neoplasm: Overlapping lesion of colon	0
C189	Malignant neoplasm: Colon, unspecified	0
C19	Malignant neoplasm of rectosigmoid junction	0
C20	Malignant neoplasm of rectum	0
C218	Malignant neoplasm: Overlapping lesion of rectum, anus and anal canal	0
C785	Secondary malignant neoplasm of large intestine and rectum	0
D010	Carcinoma in situ: Colon	0
D011	Carcinoma in situ: Rectosigmoid junction	0
D012	Carcinoma in situ: Rectum	0
D013	Carcinoma in situ: Anus and anal canal	0
D017	Carcinoma in situ: Other specified digestive organs	0
D37	Neoplasm of uncertain or unknown behaviour of oral cavity and digestive organs	0
D374	Neoplasm of uncertain or unknown behaviour: Colon	0
D375	Neoplasm of uncertain or unknown behaviour: Rectum	0

CONCEPT: Congenital disease and chromosomal abnormalities

ICD10_CODE	DESCRIPTION_SAIL	SENSITIVE_CODE
I070	Tricuspid stenosis	0
I072	Tricuspid stenosis with insufficiency	0
P70	Transitory disorders of carbohydrate metabolism specific to fetus and newborn	0
P700	Syndrome of infant of mother with gestational diabetes	0
P701	Syndrome of infant of a diabetic mother	0
P702	Neonatal diabetes mellitus	0
P703	Iatrogenic neonatal hypoglycaemia	0
P704	Other neonatal hypoglycaemia	0
P708	Other transitory disorders of carbohydrate metabolism of fetus and newborn	0
P709	Transitory disorder of carbohydrate metabolism of fetus and newborn, unspecified	0
P71	Transitory neonatal disorders of calcium and magnesium metabolism	0
P72	Other transitory neonatal endocrine disorders	0
P720	Neonatal goitre, not elsewhere classified	0
P721	Transitory neonatal hyperthyroidism	0
P722	Other transitory neonatal disorders of thyroid function, not elsewhere classified	0
P728	Other specified transitory neonatal endocrine disorders	0
P729	Transitory neonatal endocrine disorder, unspecified	0
Q20	Congenital malformations of cardiac chambers and connections	0
Q200	Common arterial trunk	0
Q201	Double outlet right ventricle	0
Q202	Double outlet left ventricle	0
Q203	Discordant ventriculoarterial connection	0
Q204	Double inlet ventricle	0
Q205	Discordant atrioventricular connection	0
Q206	Isomerism of atrial appendages	0
Q208	Other congenital malformations of cardiac chambers and connections	0
Q209	Congenital malformation of cardiac chambers and connections, unspecified	0
Q21	Congenital malformations of cardiac septa	0
Q210	Ventricular septal defect	0
Q211	Atrial septal defect	1
Q212	Atrioventricular septal defect	0
Q213	Tetralogy of Fallot	0
Q214	Aortopulmonary septal defect	0
Q218	Other congenital malformations of cardiac septa	1
Q219	Congenital malformation of cardiac septum, unspecified	1
Q22	Congenital malformations of pulmonary and tricuspid valves	0
Q220	Pulmonary valve atresia	0
Q221	Congenital pulmonary valve stenosis	0
Q222	Congenital pulmonary valve insufficiency	0
Q223	Other congenital malformations of pulmonary valve	0
Q224	Congenital tricuspid stenosis	0
Q225	Ebstein anomaly	0
Q226	Hypoplastic right heart syndrome	0
Q228	Other congenital malformations of tricuspid valve	0
Q229	Congenital malformation of tricuspid valve, unspecified	0
Q23	Congenital malformations of aortic and mitral valves	0
Q230	Congenital stenosis of aortic valve	0
Q231	Congenital insufficiency of aortic valve	0
Q232	Congenital mitral stenosis	0
Q233	Congenital mitral insufficiency	0
Q234	Hypoplastic left heart syndrome	0
Q238	Other congenital malformations of aortic and mitral valves	0
Q239	Congenital malformation of aortic and mitral valves, unspecified	0
Q24	Other congenital malformations of heart	0
Q240	Dextrocardia	0
Q241	Laevocardia	0
Q242	Cor triatriatum	0
Q243	Pulmonary infundibular stenosis	0
Q244	Congenital subaortic stenosis	0
Q245	Malformation of coronary vessels	0
Q246	Congenital heart block	0
Q248	Other specified congenital malformations of heart	0
Q249	Congenital malformation of heart, unspecified	0
Q25	Congenital malformations of great arteries	0
Q250	Patent ductus arteriosus	0
Q251	Coarctation of aorta	0
Q252	Atresia of aorta	0
Q253	Stenosis of aorta	0

Q254	Other congenital malformations of aorta	0
Q255	Atresia of pulmonary artery	0
Q256	Stenosis of pulmonary artery	0
Q257	Other congenital malformations of pulmonary artery	0
Q258	Other congenital malformations of great arteries	0
Q259	Congenital malformation of great arteries, unspecified	0
Q26	Congenital malformations of great veins	0
Q260	Congenital stenosis of vena cava	0
Q261	Persistent left superior vena cava	0
Q262	Total anomalous pulmonary venous connection	0
Q263	Partial anomalous pulmonary venous connection	0
Q264	Anomalous pulmonary venous connection, unspecified	0
Q268	Other congenital malformations of great veins	0

CONCEPT: COPD (Chronic obstructive pulmonary disease)

ICD10_CODE	DESCRIPTION_SAIL	SENSITIVE_CODE
J41	Simple and mucopurulent chronic bronchitis	0
J410	Simple chronic bronchitis	0
J411	Mucopurulent chronic bronchitis	0
J418	Mixed simple and mucopurulent chronic bronchitis	0
J42	Unspecified chronic bronchitis	0
J43	Emphysema	0
J430	MacLeod syndrome	0
J431	Panlobular emphysema	0
J432	Centrilobular emphysema	0
J438	Other emphysema	0
J439	Emphysema, unspecified	0
J44	Other chronic obstructive pulmonary disease	0
J440	Chronic obstructive pulmonary disease with acute lower respiratory infection	0
J441	Chronic obstructive pulmonary disease with acute exacerbation, unspecified	0
J448	Other specified chronic obstructive pulmonary disease	0
J449	Chronic obstructive pulmonary disease, unspecified	0
J982	Interstitial emphysema	0

CONCEPT: Coronary heart disease

ICD10_CODE	DESCRIPTION_SAIL	SENSITIVE_CODE
I20	Angina pectoris	0
I200	Unstable angina	0
I201	Angina pectoris with documented spasm	0
I208	Other forms of angina pectoris	0
I209	Angina pectoris, unspecified	0
I24	Other acute ischaemic heart diseases	0
I240	Coronary thrombosis not resulting in myocardial infarction	0
I248	Other forms of acute ischaemic heart disease	0
I249	Acute ischaemic heart disease, unspecified	0
I25	Chronic ischaemic heart disease	0
I250	Atherosclerotic cardiovascular disease, so described	0
I251	Atherosclerotic heart disease	0
I253	Aneurysm of heart	0
I254	Coronary artery aneurysm and dissection	0
I255	Ischaemic cardiomyopathy	0
I256	Silent myocardial ischaemia	0
I258	Other forms of chronic ischaemic heart disease	0
I259	Chronic ischaemic heart disease, unspecified	0
Z500	Cardiac rehabilitation	0
Z955	Presence of coronary angioplasty implant and graft	0

CONCEPT: Cystic fibrosis

ICD10_CODE	DESCRIPTION_SAIL	SENSITIVE_CODE
E84	Cystic fibrosis	0
E840	Cystic fibrosis with pulmonary manifestations	0
E841	Cystic fibrosis with intestinal manifestations	0
E848	Cystic fibrosis with other manifestations	0
E849	Cystic fibrosis, unspecified	0
P75	Meconium ileus in cystic fibrosis	0

CONCEPT: Dementia alzheimer

ICD10_CODE	DESCRIPTION_SAIL	SENSITIVE_CODE
A810	Creutzfeldt-Jakob disease	0
F00	Dementia in Alzheimer disease	0
F000	Dementia in Alzheimer disease with early onset	0
F001	Dementia in Alzheimer disease with late onset	0
F002	Dementia in Alzheimer disease, atypical or mixed type	0
F009	Dementia in Alzheimer disease, unspecified	0
F02	Dementia in other diseases classified elsewhere	0
F020	Dementia in Pick disease	0
F021	Dementia in Creutzfeldt-Jakob disease	0
F022	Dementia in Huntington disease	0
F023	Dementia in Parkinson disease	0
F024	Dementia in human immunodeficiency virus [HIV] disease	1
F028	Dementia in other specified diseases classified elsewhere	0
F03	Unspecified dementia	0
F051	Delirium superimposed on dementia	0
G30	Alzheimer disease	0
G300	Alzheimer disease with early onset	0
G301	Alzheimer disease with late onset	0
G308	Other Alzheimer disease	0
G309	Alzheimer disease, unspecified	0
G912	Normal-pressure hydrocephalus	0

CONCEPT: Depression

ICD10_CODE	DESCRIPTION_SAIL	SENSITIVE_CODE
F204	Post-schizophrenic depression	0
F251	Schizoaffective disorder, depressive type	0
F32	Depressive episode	0
F320	Mild depressive episode	0
F321	Moderate depressive episode	0
F322	Severe depressive episode without psychotic symptoms	0
F323	Severe depressive episode with psychotic symptoms	0

F328	Other depressive episodes	0
F329	Depressive episode, unspecified	0
F33	Recurrent depressive disorder	0
F330	Recurrent depressive disorder, current episode mild	0
F331	Recurrent depressive disorder, current episode moderate	0
F332	Recurrent depressive disorder, current episode severe without psychotic symptoms	0
F333	Recurrent depressive disorder, current episode severe with psychotic symptoms	0
F338	Other recurrent depressive disorders	0
F339	Recurrent depressive disorder, unspecified	0
F341	Dysthymia	0
F412	Mixed anxiety and depressive disorder	0
F920	Depressive conduct disorder	0

CONCEPT: Diabetes Type 1

ICD10_CODE	DESCRIPTION_SAIL	SENSITIVE_CODE
E10	Type 1 diabetes mellitus	0
E100	Type 1 diabetes mellitus	0
E101	Type 1 diabetes mellitus	0
E102	Type 1 diabetes mellitus	0
E103	Type 1 diabetes mellitus	0
E104	Type 1 diabetes mellitus	0
E105	Type 1 diabetes mellitus	0
E106	Type 1 diabetes mellitus	0
E107	Type 1 diabetes mellitus	0
E108	Type 1 diabetes mellitus	0
E109	Type 1 diabetes mellitus	0
O240	Diabetes mellitus in pregnancy: Pre-existing type 1 diabetes mellitus	0

CONCEPT: Diabetes Type 2

ICD10_CODE	DESCRIPTION_SAIL	SENSITIVE_CODE
E11	Type 2 diabetes mellitus	0
E110	Type 2 diabetes mellitus	0
E111	Type 2 diabetes mellitus	0
E112	Type 2 diabetes mellitus	0
E113	Type 2 diabetes mellitus	0
E114	Type 2 diabetes mellitus	0
E115	Type 2 diabetes mellitus	0
E116	Type 2 diabetes mellitus	0
E117	Type 2 diabetes mellitus	0
E118	Type 2 diabetes mellitus	0
E119	Type 2 diabetes mellitus	0
O241	Diabetes mellitus in pregnancy: Pre-existing type 2 diabetes mellitus	0

CONCEPT: Diabetic retinopathy

ICD10_CODE	DESCRIPTION_SAIL	SENSITIVE_CODE
E103	Type 1 diabetes mellitus	0
E113	Type 2 diabetes mellitus	0
E133	Other specified diabetes mellitus	0
E143	Unspecified diabetes mellitus	0
H360	Diabetic retinopathy	0

CONCEPT: Dialysis

ICD10_CODE	DESCRIPTION_SAIL	SENSITIVE_CODE
Y602	During kidney dialysis or other perfusion	0
Y612	During kidney dialysis or other perfusion	0
Y622	During kidney dialysis or other perfusion	0
Y841	Kidney dialysis	0
Z49	Care involving dialysis	0
Z490	Preparatory care for dialysis	0
Z491	Extracorporeal dialysis	0
Z492	Other dialysis	0
Z992	Dependence on renal dialysis	0

CONCEPT: Diverticular Disease

ICD10_CODE	DESCRIPTION_SAIL	SENSITIVE_CODE
K57	Diverticular disease of intestine	0
K570	Diverticular disease of small intestine with perforation and abscess	0
K571	Diverticular disease of small intestine without perforation or abscess	0
K572	Diverticular disease of large intestine with perforation and abscess	0
K573	Diverticular disease of large intestine without perforation or abscess	0
K574	Diverticular disease of both small and large intestine with perforation and abscess	0
K575	Diverticular disease of both small and large intestine without perforation or abscess	0
K578	Diverticular disease of intestine, part unspecified, with perforation and abscess	0
K579	Diverticular disease of intestine, part unspecified, without perforation or abscess	0

CONCEPT: Drug or alcohol misuse

ICD10_CODE	DESCRIPTION_SAIL	SENSITIVE_CODE
E244	Alcohol-induced pseudo-Cushing syndrome	0
F04	Organic amnesic syndrome, not induced by alcohol and other psychoactive substances	0
F05	Delirium, not induced by alcohol and other psychoactive substances	0
F10	Mental and behavioural disorders due to use of alcohol	0
F100	Mental and behavioural disorders due to use of alcohol	0
F101	Mental and behavioural disorders due to use of alcohol	0
F102	Mental and behavioural disorders due to use of alcohol	0
F103	Mental and behavioural disorders due to use of alcohol	0
F104	Mental and behavioural disorders due to use of alcohol	0
F105	Mental and behavioural disorders due to use of alcohol	0
F106	Mental and behavioural disorders due to use of alcohol	0
F107	Mental and behavioural disorders due to use of alcohol	0
F108	Mental and behavioural disorders due to use of alcohol	0
F109	Mental and behavioural disorders due to use of alcohol	0
F19	Mental and behavioural disorders due to multiple drug use and use of other psychoactive substances	0
F190	Mental and behavioural disorders due to multiple drug use and use of other psychoactive substances	0
F191	Mental and behavioural disorders due to multiple drug use and use of other psychoactive substances	0
F192	Mental and behavioural disorders due to multiple drug use and use of other psychoactive substances	0
F193	Mental and behavioural disorders due to multiple drug use and use of other psychoactive substances	0
F194	Mental and behavioural disorders due to multiple drug use and use of other psychoactive substances	0
F195	Mental and behavioural disorders due to multiple drug use and use of other psychoactive substances	0
F196	Mental and behavioural disorders due to multiple drug use and use of other psychoactive substances	0

F197	Mental and behavioural disorders due to multiple drug use and use of other psychoactive substances	0
F198	Mental and behavioural disorders due to multiple drug use and use of other psychoactive substances	0
F199	Mental and behavioural disorders due to multiple drug use and use of other psychoactive substances	0
F55	Abuse of non-dependence-producing substances	0
G312	Degeneration of nervous system due to alcohol	0
G621	Alcoholic polyneuropathy	0
G721	Alcoholic myopathy	0
I426	Alcoholic cardiomyopathy	0
K292	Alcoholic gastritis	0
K70	Alcoholic liver disease	0
K700	Alcoholic fatty liver	0
K701	Alcoholic hepatitis	0
K702	Alcoholic fibrosis and sclerosis of liver	0
K703	Alcoholic cirrhosis of liver	0
K704	Alcoholic hepatic failure	0
K709	Alcoholic liver disease, unspecified	0
K852	Alcohol-induced acute pancreatitis	0
K860	Alcohol-induced chronic pancreatitis	0
T436	Poisoning: Psychostimulants with abuse potential	0
Y497	Psychostimulants with abuse potential	0
Z722	Drug use	0
Z864	Personal history of psychoactive substance abuse	0

CONCEPT: Eating disorders

ICD10_CODE	DESCRIPTION_SAIL	SENSITIVE_CODE
F50	Eating disorders	0
F500	Anorexia nervosa	0
F501	Atypical anorexia nervosa	0
F502	Bulimia nervosa	0
F503	Atypical bulimia nervosa	0
F504	Overeating associated with other psychological disturbances	0
F505	Vomiting associated with other psychological disturbances	0
F508	Other eating disorders	0
F509	Eating disorder, unspecified	0
F983	Pica of infancy and childhood	0

CONCEPT: Epilepsy

ICD10_CODE	DESCRIPTION_SAIL	SENSITIVE_CODE
F803	Acquired aphasia with epilepsy [Landau-Kleffner]	0
G40	Epilepsy	0
G400	Localization-related (focal)(partial) idiopathic epilepsy and epileptic syndromes with seizures of localized onset	0
G401	Localization-related (focal)(partial) symptomatic epilepsy and epileptic syndromes with simple partial seizures	0
G402	Localization-related (focal)(partial) symptomatic epilepsy and epileptic syndromes with complex partial seizures	0
G403	Generalized idiopathic epilepsy and epileptic syndromes	0
G404	Other generalized epilepsy and epileptic syndromes	0
G405	Special epileptic syndromes	0
G408	Other epilepsy	0
G409	Epilepsy, unspecified	0
G41	Status epilepticus	0
G410	Grand mal status epilepticus	0
G411	Petit mal status epilepticus	0
G412	Complex partial status epilepticus	0
G418	Other status epilepticus	0
G419	Status epilepticus, unspecified	0

CONCEPT: Fibromyalgia

ICD10_CODE	DESCRIPTION_SAIL	SENSITIVE_CODE
M797	Fibromyalgia	0
M7970	Fibromyalgia	0
M7971	Fibromyalgia	0
M7972	Fibromyalgia	0
M7973	Fibromyalgia	0
M7974	Fibromyalgia	0
M7975	Fibromyalgia	0
M7976	Fibromyalgia	0
M7977	Fibromyalgia	0
M7978	Fibromyalgia	0
M7979	Fibromyalgia	0

CONCEPT: Glaucoma

ICD10_CODE	DESCRIPTION_SAIL	SENSITIVE_CODE
H40	Glaucoma	0
H401	Primary open-angle glaucoma	0
H402	Primary angle-closure glaucoma	0
H408	Other glaucoma	0
H409	Glaucoma, unspecified	0
H42	Glaucoma in diseases classified elsewhere	0
H420	Glaucoma in endocrine, nutritional and metabolic diseases	0
H428	Glaucoma in other diseases classified elsewhere	0

CONCEPT: Gout

ICD10_CODE	DESCRIPTION_SAIL	SENSITIVE_CODE
M10	Gout	0
M100	Idiopathic gout	0
M1000	Idiopathic gout	0
M1001	Idiopathic gout	0
M1002	Idiopathic gout	0
M1003	Idiopathic gout	0
M1004	Idiopathic gout	0
M1005	Idiopathic gout	0
M1006	Idiopathic gout	0
M1007	Idiopathic gout	0
M1008	Idiopathic gout	0
M1009	Idiopathic gout	0
M101	Lead-induced gout	0
M1010	Lead-induced gout	0
M1011	Lead-induced gout	0
M1012	Lead-induced gout	0
M1013	Lead-induced gout	0

M1014	Lead-induced gout	0
M1015	Lead-induced gout	0
M1016	Lead-induced gout	0
M1017	Lead-induced gout	0
M1018	Lead-induced gout	0
M1019	Lead-induced gout	0
M102	Drug-induced gout	0
M1020	Drug-induced gout	0
M1021	Drug-induced gout	0
M1022	Drug-induced gout	0
M1023	Drug-induced gout	0
M1024	Drug-induced gout	0
M1025	Drug-induced gout	0
M1026	Drug-induced gout	0
M1027	Drug-induced gout	0
M1028	Drug-induced gout	0
M1029	Drug-induced gout	0
M103	Gout due to impairment of renal function	0
M1030	Gout due to impairment of renal function	0
M1031	Gout due to impairment of renal function	0
M1032	Gout due to impairment of renal function	0
M1033	Gout due to impairment of renal function	0
M1034	Gout due to impairment of renal function	0
M1035	Gout due to impairment of renal function	0
M1036	Gout due to impairment of renal function	0
M1037	Gout due to impairment of renal function	0
M1038	Gout due to impairment of renal function	0
M1039	Gout due to impairment of renal function	0
M104	Other secondary gout	0
M1040	Other secondary gout	0
M1041	Other secondary gout	0
M1042	Other secondary gout	0
M1043	Other secondary gout	0
M1044	Other secondary gout	0
M1045	Other secondary gout	0
M1046	Other secondary gout	0
M1047	Other secondary gout	0
M1048	Other secondary gout	0
M1049	Other secondary gout	0
M109	Gout, unspecified	0
M1090	Gout, unspecified	0
M1091	Gout, unspecified	0
M1092	Gout, unspecified	0
M1093	Gout, unspecified	0
M1094	Gout, unspecified	0
M1095	Gout, unspecified	0
M1096	Gout, unspecified	0
M1097	Gout, unspecified	0
M1098	Gout, unspecified	0
M1099	Gout, unspecified	0
M140	Gouty arthropathy due to enzyme defects and other inherited disorders	0

CONCEPT: Heart Valve Disorders

ICD10_CODE	DESCRIPTION_SAIL	SENSITIVE_CODE
G110	Congenital nonprogressive ataxia	0
G111	Early-onset cerebellar ataxia	0
G112	Late-onset cerebellar ataxia	0
G113	Cerebellar ataxia with defective DNA repair	0
G114	Hereditary spastic paraplegia	0
G118	Other hereditary ataxias	0
G119	Hereditary ataxia, unspecified	0
I05	Rheumatic mitral valve diseases	0
I050	Mitral stenosis	0
I051	Rheumatic mitral insufficiency	0
I052	Mitral stenosis with insufficiency	0
I058	Other mitral valve diseases	0
I059	Mitral valve disease, unspecified	0
I06	Rheumatic aortic valve diseases	0
I060	Rheumatic aortic stenosis	0
I062	Rheumatic aortic stenosis with insufficiency	0
I068	Other rheumatic aortic valve diseases	0
I069	Rheumatic aortic valve disease, unspecified	0
I07	Rheumatic tricuspid valve diseases	0
I070	Tricuspid stenosis	0
I072	Tricuspid stenosis with insufficiency	0
I078	Other tricuspid valve diseases	0
I079	Tricuspid valve disease, unspecified	0
I08	Multiple valve diseases	0
I080	Disorders of both mitral and aortic valves	0
I081	Disorders of both mitral and tricuspid valves	0
I082	Disorders of both aortic and tricuspid valves	0
I083	Combined disorders of mitral, aortic and tricuspid valves	0
I088	Other multiple valve diseases	0
I089	Multiple valve disease, unspecified	0
I091	Rheumatic diseases of endocardium, valve unspecified	0
I34	Nonrheumatic mitral valve disorders	0
I340	Mitral (valve) insufficiency	0
I341	Mitral (valve) prolapse	0
I342	Nonrheumatic mitral (valve) stenosis	0
I348	Other nonrheumatic mitral valve disorders	0
I349	Nonrheumatic mitral valve disorder, unspecified	0
I35	Nonrheumatic aortic valve disorders	0
I350	Aortic (valve) stenosis	0
I351	Aortic (valve) insufficiency	0
I352	Aortic (valve) stenosis with insufficiency	0
I358	Other aortic valve disorders	0
I359	Aortic valve disorder, unspecified	0
I36	Nonrheumatic tricuspid valve disorders	0
I360	Nonrheumatic tricuspid (valve) stenosis	0
I361	Nonrheumatic tricuspid (valve) insufficiency	0
I362	Nonrheumatic tricuspid (valve) stenosis with insufficiency	0
I368	Other nonrheumatic tricuspid valve disorders	0

I369	Nonrheumatic tricuspid valve disorder, unspecified	0
I37	Pulmonary valve disorders	0
I370	Pulmonary valve stenosis	0
I371	Pulmonary valve insufficiency	0
I372	Pulmonary valve stenosis with insufficiency	0
I378	Other pulmonary valve disorders	0
I379	Pulmonary valve disorder, unspecified	0
I38	Endocarditis, valve unspecified	0
I39	Endocarditis and heart valve disorders in diseases classified elsewhere	0
I390	Mitral valve disorders in diseases classified elsewhere	0
I391	Aortic valve disorders in diseases classified elsewhere	0
I392	Tricuspid valve disorders in diseases classified elsewhere	0
I393	Pulmonary valve disorders in diseases classified elsewhere	0
I394	Multiple valve disorders in diseases classified elsewhere	0
I398	Endocarditis, valve unspecified, in diseases classified elsewhere	0
Q22	Congenital malformations of pulmonary and tricuspid valves	0
Q220	Pulmonary valve atresia	0
Q221	Congenital pulmonary valve stenosis	0
Q222	Congenital pulmonary valve insufficiency	0
Q223	Other congenital malformations of pulmonary valve	0
Q224	Congenital tricuspid stenosis	0
Q225	Ebstein anomaly	0
Q228	Other congenital malformations of tricuspid valve	0
Q229	Congenital malformation of tricuspid valve, unspecified	0
Q23	Congenital malformations of aortic and mitral valves	0
Q230	Congenital stenosis of aortic valve	0
Q231	Congenital insufficiency of aortic valve	0
Q232	Congenital mitral stenosis	0
Q233	Congenital mitral insufficiency	0
Q238	Other congenital malformations of aortic and mitral valves	0
Q239	Congenital malformation of aortic and mitral valves, unspecified	0
Q243	Pulmonary infundibular stenosis	0
Q244	Congenital subaortic stenosis	0
Q642	Congenital posterior urethral valves	0
T820	Mechanical complication of heart valve prosthesis	0
T822	Mechanical complication of coronary artery bypass and valve grafts	0
T826	Infection and inflammatory reaction due to cardiac valve prosthesis	0
Z952	Presence of prosthetic heart valve	0
Z953	Presence of xenogenic heart valve	0
Z954	Presence of other heart-valve replacement	0

CONCEPT: HF (Heart Failure)

ICD10_CODE	DESCRIPTION_SAIL	SENSITIVE_CODE
I11	Hypertensive heart disease	0
I110	Hypertensive heart disease with (congestive) heart failure	0
I119	Hypertensive heart disease without (congestive) heart failure	0
I13	Hypertensive heart and renal disease	0
I130	Hypertensive heart and renal disease with (congestive) heart failure	0
I131	Hypertensive heart and renal disease with renal failure	0
I132	Hypertensive heart and renal disease with both (congestive) heart failure and renal failure	0
I139	Hypertensive heart and renal disease, unspecified	0
I255	Ischaemic cardiomyopathy	0
I260	Pulmonary embolism with mention of acute cor pulmonale	0
I420	Dilated cardiomyopathy	0
I421	Obstructive hypertrophic cardiomyopathy	0
I422	Other hypertrophic cardiomyopathy	0
I429	Cardiomyopathy, unspecified	0
I50	Heart failure	0
I500	Congestive heart failure	0
I501	Left ventricular failure	0
I509	Heart failure, unspecified	0
P290	Neonatal cardiac failure	0
Z994	Dependence on artificial heart	0

CONCEPT: HIV/AIDS

ICD10_CODE	DESCRIPTION_SAIL	SENSITIVE_CODE
B20	Human immunodeficiency virus [HIV] disease resulting in infectious and parasitic diseases	1
B200	HIV disease resulting in mycobacterial infection	1
B201	HIV disease resulting in other bacterial infections	1
B202	HIV disease resulting in cytomegaloviral disease	1
B203	HIV disease resulting in other viral infections	1
B204	HIV disease resulting in candidiasis	1
B205	HIV disease resulting in other mycoses	1
B206	HIV disease resulting in Pneumocystis jirovecii pneumonia	1
B207	HIV disease resulting in multiple infections	1
B208	HIV disease resulting in other infectious and parasitic diseases	1
B209	HIV disease resulting in unspecified infectious or parasitic disease	1
B21	Human immunodeficiency virus [HIV] disease resulting in malignant neoplasms	1
B210	HIV disease resulting in Kaposi sarcoma	1
B211	HIV disease resulting in Burkitt lymphoma	1
B212	HIV disease resulting in other types of non-Hodgkin lymphoma	1
B213	HIV disease resulting in other malignant neoplasms of lymphoid, haematopoietic and related tissue	1
B217	HIV disease resulting in multiple malignant neoplasms	1
B218	HIV disease resulting in other malignant neoplasms	1
B219	HIV disease resulting in unspecified malignant neoplasm	1
B22	Human immunodeficiency virus [HIV] disease resulting in other specified diseases	1
B220	HIV disease resulting in encephalopathy	1
B221	HIV disease resulting in lymphoid interstitial pneumonitis	1
B222	HIV disease resulting in wasting syndrome	1
B227	HIV disease resulting in multiple diseases classified elsewhere	1
B23	Human immunodeficiency virus [HIV] disease resulting in other conditions	1
B230	Acute HIV infection syndrome	1
B231	HIV disease resulting in (persistent) generalized lymphadenopathy	1
B232	HIV disease resulting in haematological and immunological abnormalities, not elsewhere classified	1
B238	HIV disease resulting in other specified conditions	1
B24	Unspecified human immunodeficiency virus [HIV] disease	1
F024	Dementia in human immunodeficiency virus [HIV] disease	1
O987	Human immunodeficiency virus [HIV] disease complicating pregnancy, childbirth and the puerperium	1
R75	Laboratory evidence of human immunodeficiency virus [HIV]	1
Z21	Asymptomatic human immunodeficiency virus [HIV] infection status	1

CONCEPT: Hypertension

ICD10_CODE	DESCRIPTION_SAIL	SENSITIVE_CODE
I10	Essential (primary) hypertension	0
I11	Hypertensive heart disease	0
I110	Hypertensive heart disease with (congestive) heart failure	0
I119	Hypertensive heart disease without (congestive) heart failure	0
I12	Hypertensive renal disease	0
I120	Hypertensive renal disease with renal failure	0
I129	Hypertensive renal disease without renal failure	0
I13	Hypertensive heart and renal disease	0
I130	Hypertensive heart and renal disease with (congestive) heart failure	0
I131	Hypertensive heart and renal disease with renal failure	0
I132	Hypertensive heart and renal disease with both (congestive) heart failure and renal failure	0
I139	Hypertensive heart and renal disease, unspecified	0
I15	Secondary hypertension	0
I150	Renovascular hypertension	0
I151	Hypertension secondary to other renal disorders	0
I152	Hypertension secondary to endocrine disorders	0
I158	Other secondary hypertension	0
I159	Secondary hypertension, unspecified	0
I674	Hypertensive encephalopathy	0
O10	Pre-existing hypertension complicating pregnancy, childbirth and the puerperium	0
O100	Pre-existing essential hypertension complicating pregnancy, childbirth and the puerperium	0
O101	Pre-existing hypertensive heart disease complicating pregnancy, childbirth and the puerperium	0
O102	Pre-existing hypertensive renal disease complicating pregnancy, childbirth and the puerperium	0
O103	Pre-existing hypertensive heart and renal disease complicating pregnancy, childbirth and the puerperium	0
O104	Pre-existing secondary hypertension complicating pregnancy, childbirth and the puerperium	0
Y525	Other antihypertensive drugs, not elsewhere classified	0

CONCEPT: Hyperthyroidism

ICD10_CODE	DESCRIPTION_SAIL	SENSITIVE_CODE
E05	Thyrotoxicosis [hyperthyroidism]	0
E050	Thyrotoxicosis with diffuse goitre	0
E051	Thyrotoxicosis with toxic single thyroid nodule	0
E052	Thyrotoxicosis with toxic multinodular goitre	0
E053	Thyrotoxicosis from ectopic thyroid tissue	0
E059	Thyrotoxicosis, unspecified	0

CONCEPT: Hypothyroidism

ICD10_CODE	DESCRIPTION_SAIL	SENSITIVE_CODE
E001	Congenital iodine-deficiency syndrome, myxoedematous type	0
E03	Other hypothyroidism	0
E030	Congenital hypothyroidism with diffuse goitre	0
E031	Congenital hypothyroidism without goitre	0
E032	Hypothyroidism due to medicaments and other exogenous substances	0
E033	Postinfectious hypothyroidism	0
E034	Atrophy of thyroid (acquired)	0
E035	Myxoedema coma	0
E038	Other specified hypothyroidism	0
E039	Hypothyroidism, unspecified	0
E890	Postprocedural hypothyroidism	0
G132	Systemic atrophy primarily affecting central nervous system in myxoedema	0

CONCEPT: IBD (Inflammatory bowel disease)

ICD10_CODE	DESCRIPTION_SAIL	SENSITIVE_CODE
K50	Crohn disease [regional enteritis]	0
K500	Crohn disease of small intestine	0
K501	Crohn disease of large intestine	0
K508	Other Crohn disease	0
K509	Crohn disease, unspecified	0
K51	Ulcerative colitis	0
K510	Ulcerative (chronic) pancolitis	0
K512	Ulcerative (chronic) proctitis	0
K513	Ulcerative (chronic) rectosigmoiditis	0
K518	Other ulcerative colitis	0
K519	Ulcerative colitis, unspecified	0
M074	Arthropathy in Crohn disease [regional enteritis]	0
M0740	Arthropathy in Crohn disease [regional enteritis]	0
M0741	Arthropathy in Crohn disease [regional enteritis]	0
M0742	Arthropathy in Crohn disease [regional enteritis]	0
M0743	Arthropathy in Crohn disease [regional enteritis]	0
M0744	Arthropathy in Crohn disease [regional enteritis]	0
M0745	Arthropathy in Crohn disease [regional enteritis]	0
M0746	Arthropathy in Crohn disease [regional enteritis]	0
M0747	Arthropathy in Crohn disease [regional enteritis]	0
M0748	Arthropathy in Crohn disease [regional enteritis]	0
M0749	Arthropathy in Crohn disease [regional enteritis]	0
M075	Arthropathy in ulcerative colitis	0
M0750	Arthropathy in ulcerative colitis	0
M0751	Arthropathy in ulcerative colitis	0
M0752	Arthropathy in ulcerative colitis	0
M0753	Arthropathy in ulcerative colitis	0
M0754	Arthropathy in ulcerative colitis	0
M0755	Arthropathy in ulcerative colitis	0
M0756	Arthropathy in ulcerative colitis	0
M0757	Arthropathy in ulcerative colitis	0
M0758	Arthropathy in ulcerative colitis	0
M0759	Arthropathy in ulcerative colitis	0
M091	Juvenile arthritis in Crohn disease [regional enteritis]	0
M0910	Juvenile arthritis in Crohn disease [regional enteritis]	0
M0911	Juvenile arthritis in Crohn disease [regional enteritis]	0
M0912	Juvenile arthritis in Crohn disease [regional enteritis]	0
M0913	Juvenile arthritis in Crohn disease [regional enteritis]	0
M0914	Juvenile arthritis in Crohn disease [regional enteritis]	0
M0915	Juvenile arthritis in Crohn disease [regional enteritis]	0
M0916	Juvenile arthritis in Crohn disease [regional enteritis]	0
M0917	Juvenile arthritis in Crohn disease [regional enteritis]	0
M0918	Juvenile arthritis in Crohn disease [regional enteritis]	0
M0919	Juvenile arthritis in Crohn disease [regional enteritis]	0
M092	Juvenile arthritis in ulcerative colitis	0

M0920	Juvenile arthritis in ulcerative colitis	0
M0921	Juvenile arthritis in ulcerative colitis	0
M0922	Juvenile arthritis in ulcerative colitis	0
M0923	Juvenile arthritis in ulcerative colitis	0
M0924	Juvenile arthritis in ulcerative colitis	0
M0925	Juvenile arthritis in ulcerative colitis	0
M0926	Juvenile arthritis in ulcerative colitis	0
M0927	Juvenile arthritis in ulcerative colitis	0
M0928	Juvenile arthritis in ulcerative colitis	0
M0929	Juvenile arthritis in ulcerative colitis	0

CONCEPT: ILD (Inflammatory Lung Disease)

ICD10_CODE	DESCRIPTION_SAIL	SENSITIVE_CODE
J703	Chronic drug-induced interstitial lung disorders	0
J704	Drug-induced interstitial lung disorders, unspecified	0
J841	Other interstitial pulmonary diseases with fibrosis	0

CONCEPT: Leukaemia

ICD10_CODE	DESCRIPTION_SAIL	SENSITIVE_CODE
B213	HIV disease resulting in other malignant neoplasms of lymphoid, haematopoietic and related tissue	1
C91	Lymphoid leukaemia	0
C910	Acute lymphoblastic leukaemia [ALL]	0
C911	Chronic lymphocytic leukaemia of B-cell type	0
C913	Prolymphocytic leukaemia of B-cell type	0
C914	Hairy-cell leukaemia	0
C915	Adult T-cell lymphoma/leukaemia [HTLV-1-associated]	0
C916	Prolymphocytic leukaemia of T-cell type	0
C917	Other lymphoid leukaemia	0
C918	Mature B-cell leukaemia Burkitt-type	0
C919	Lymphoid leukaemia, unspecified	0
C92	Myeloid leukaemia	0
C920	Acute myeloblastic leukaemia [AML]	0
C921	Chronic myeloid leukaemia [CML], BCR/ABL-positive	0
C922	Atypical chronic myeloid leukaemia, BCR/ABL-negative	0
C923	Myeloid sarcoma	0
C924	Acute promyelocytic leukaemia [PML]	0
C925	Acute myelomonocytic leukaemia	0
C926	Acute myeloid leukaemia with 11q23-abnormality	0
C927	Other myeloid leukaemia	0
C928	Acute myeloid leukaemia with multilineage dysplasia	0
C929	Myeloid leukaemia, unspecified	0
C93	Monocytic leukaemia	0
C930	Acute monoblastic/monocytic leukaemia	0
C931	Chronic myelomonocytic leukaemia	0
C933	Juvenile myelomonocytic leukaemia	0
C937	Other monocytic leukaemia	0
C939	Monocytic leukaemia, unspecified	0
C94	Other leukaemias of specified cell type	0
C940	Acute erythroid leukaemia	0
C942	Acute megakaryoblastic leukaemia	0
C943	Mast cell leukaemia	0
C944	Acute panmyelosis with myelofibrosis	0
C946	Myelodysplastic and myeloproliferative disease, not elsewhere classified	0
C947	Other specified leukaemias	0
C95	Leukaemia of unspecified cell type	0
C950	Acute leukaemia of unspecified cell type	0
C951	Chronic leukaemia of unspecified cell type	0
C957	Other leukaemia of unspecified cell type	0
C959	Leukaemia, unspecified	0
C96	Other and unspecified malignant neoplasms of lymphoid, haematopoietic and related tissue	0
C967	Other specified malignant neoplasms of lymphoid, haematopoietic and related tissue	0
C969	Malignant neoplasm of lymphoid, haematopoietic and related tissue, unspecified	0
D474	Osteomyelofibrosis	0
D475	Chronic eosinophilic leukaemia [hypereosinophilic syndrome]	0
Z856	Personal history of leukaemia	0
Z857	Personal history of other malignant neoplasms of lymphoid, haematopoietic and related tissues	0

CONCEPT: Metastatic cancers

ICD10_CODE	DESCRIPTION_SAIL	SENSITIVE_CODE
C77	Secondary and unspecified malignant neoplasm of lymph nodes	0
C770	Secondary and unspecified malignant neoplasm: Lymph nodes of head, face and neck	0
C771	Secondary and unspecified malignant neoplasm: Intrathoracic lymph nodes	0
C772	Secondary and unspecified malignant neoplasm: Intra-abdominal lymph nodes	0
C773	Secondary and unspecified malignant neoplasm: Axillary and upper limb lymph nodes	0
C774	Secondary and unspecified malignant neoplasm: Inguinal and lower limb lymph nodes	0
C775	Secondary and unspecified malignant neoplasm: Intrapelvic lymph nodes	0
C778	Secondary and unspecified malignant neoplasm: Lymph nodes of multiple regions	0
C779	Secondary and unspecified malignant neoplasm: Lymph node, unspecified	0
C78	Secondary malignant neoplasm of respiratory and digestive organs	0
C780	Secondary malignant neoplasm of lung	0
C781	Secondary malignant neoplasm of mediastinum	0
C782	Secondary malignant neoplasm of pleura	0
C783	Secondary malignant neoplasm of other and unspecified respiratory organs	0
C784	Secondary malignant neoplasm of small intestine	0
C785	Secondary malignant neoplasm of large intestine and rectum	0
C786	Secondary malignant neoplasm of retroperitoneum and peritoneum	0
C787	Secondary malignant neoplasm of liver and intrahepatic bile duct	0
C788	Secondary malignant neoplasm of other and unspecified digestive organs	0
C79	Secondary malignant neoplasm of other and unspecified sites	0
C790	Secondary malignant neoplasm of kidney and renal pelvis	0
C791	Secondary malignant neoplasm of bladder and other and unspecified urinary organs	0
C792	Secondary malignant neoplasm of skin	0
C793	Secondary malignant neoplasm of brain and cerebral meninges	0
C794	Secondary malignant neoplasm of other and unspecified parts of nervous system	0
C795	Secondary malignant neoplasm of bone and bone marrow	0
C796	Secondary malignant neoplasm of ovary	0
C797	Secondary malignant neoplasm of adrenal gland	0
C798	Secondary malignant neoplasm of other specified sites	0
C799	Secondary malignant neoplasm, unspecified site	0

CONCEPT: Multiple sclerosis

ICD10_CODE	DESCRIPTION_SAIL	SENSITIVE_CODE
G35	Multiple sclerosis	0

CONCEPT: Osteoarthritis

ICD10_CODE	DESCRIPTION_SAIL	SENSITIVE_CODE
G552	Nerve root and plexus compressions in spondylosis	0
M15	Polyarthrosis	0
M150	Primary generalized (osteo)arthrosis	0
M153	Secondary multiple arthrosis	0
M154	Erosive (osteo)arthrosis	0
M158	Other polyarthrosis	0
M159	Polyarthrosis, unspecified	0
M16	Coxarthrosis [arthrosis of hip]	0
M160	Primary coxarthrosis, bilateral	0
M161	Other primary coxarthrosis	0
M164	Post-traumatic coxarthrosis, bilateral	0
M165	Other post-traumatic coxarthrosis	0
M166	Other secondary coxarthrosis, bilateral	0
M167	Other secondary coxarthrosis	0
M169	Coxarthrosis, unspecified	0
M17	Gonarthrosis [arthrosis of knee]	0
M170	Primary gonarthrosis, bilateral	0
M171	Other primary gonarthrosis	0
M172	Post-traumatic gonarthrosis, bilateral	0
M173	Other post-traumatic gonarthrosis	0
M174	Other secondary gonarthrosis, bilateral	0
M175	Other secondary gonarthrosis	0
M179	Gonarthrosis, unspecified	0
M18	Arthrosis of first carpometacarpal joint	0
M180	Primary arthrosis of first carpometacarpal joints, bilateral	0
M181	Other primary arthrosis of first carpometacarpal joint	0
M182	Post-traumatic arthrosis of first carpometacarpal joints, bilateral	0
M183	Other post-traumatic arthrosis of first carpometacarpal joint	0
M184	Other secondary arthrosis of first carpometacarpal joints, bilateral	0
M185	Other secondary arthrosis of first carpometacarpal joint	0
M189	Arthrosis of first carpometacarpal joint, unspecified	0
M19	Other arthrosis	0
M190	Primary arthrosis of other joints	0
M1900	Primary arthrosis of other joints	0
M1901	Primary arthrosis of other joints	0
M1902	Primary arthrosis of other joints	0
M1903	Primary arthrosis of other joints	0
M1904	Primary arthrosis of other joints	0
M1905	Primary arthrosis of other joints	0
M1907	Primary arthrosis of other joints	0
M1908	Primary arthrosis of other joints	0
M1909	Primary arthrosis of other joints	0
M191	Post-traumatic arthrosis of other joints	0
M1910	Post-traumatic arthrosis of other joints	0
M1911	Post-traumatic arthrosis of other joints	0
M1912	Post-traumatic arthrosis of other joints	0
M1913	Post-traumatic arthrosis of other joints	0
M1914	Post-traumatic arthrosis of other joints	0
M1915	Post-traumatic arthrosis of other joints	0
M1917	Post-traumatic arthrosis of other joints	0
M1918	Post-traumatic arthrosis of other joints	0
M1919	Post-traumatic arthrosis of other joints	0
M192	Other secondary arthrosis	0
M1920	Other secondary arthrosis	0
M1921	Other secondary arthrosis	0
M1922	Other secondary arthrosis	0
M1923	Other secondary arthrosis	0
M1924	Other secondary arthrosis	0
M1925	Other secondary arthrosis	0
M1927	Other secondary arthrosis	0
M1928	Other secondary arthrosis	0
M1929	Other secondary arthrosis	0
M198	Other specified arthrosis	0
M1980	Other specified arthrosis	0
M1981	Other specified arthrosis	0
M1982	Other specified arthrosis	0
M1983	Other specified arthrosis	0
M1984	Other specified arthrosis	0
M1985	Other specified arthrosis	0
M1986	Other specified arthrosis	0
M1987	Other specified arthrosis	0
M1988	Other specified arthrosis	0
M1989	Other specified arthrosis	0
M199	Arthrosis, unspecified	0
M1990	Arthrosis, unspecified	0
M1991	Arthrosis, unspecified	0
M1992	Arthrosis, unspecified	0
M1993	Arthrosis, unspecified	0
M1994	Arthrosis, unspecified	0
M1995	Arthrosis, unspecified	0
M1997	Arthrosis, unspecified	0
M1998	Arthrosis, unspecified	0
M1999	Arthrosis, unspecified	0
M47	Spondylosis	0
M471	Other spondylosis with myelopathy	0
M4710	Other spondylosis with myelopathy	0
M4711	Other spondylosis with myelopathy	0
M4712	Other spondylosis with myelopathy	0
M4713	Other spondylosis with myelopathy	0
M4714	Other spondylosis with myelopathy	0
M4715	Other spondylosis with myelopathy	0
M4716	Other spondylosis with myelopathy	0
M4717	Other spondylosis with myelopathy	0
M4718	Other spondylosis with myelopathy	0
M4719	Other spondylosis with myelopathy	0
M472	Other spondylosis with radiculopathy	0

M4720	Other spondylosis with radiculopathy	0
M4721	Other spondylosis with radiculopathy	0
M4722	Other spondylosis with radiculopathy	0
M4723	Other spondylosis with radiculopathy	0
M4724	Other spondylosis with radiculopathy	0
M4725	Other spondylosis with radiculopathy	0
M4726	Other spondylosis with radiculopathy	0
M4727	Other spondylosis with radiculopathy	0
M4728	Other spondylosis with radiculopathy	0
M4729	Other spondylosis with radiculopathy	0
M478	Other spondylosis	0
M4780	Other spondylosis	0
M4781	Other spondylosis	0
M4782	Other spondylosis	0
M4783	Other spondylosis	0
M4784	Other spondylosis	0
M4785	Other spondylosis	0
M4786	Other spondylosis	0
M4787	Other spondylosis	0
M4788	Other spondylosis	0
M4789	Other spondylosis	0
M479	Spondylosis, unspecified	0
M4790	Spondylosis, unspecified	0
M4791	Spondylosis, unspecified	0
M4792	Spondylosis, unspecified	0
M4793	Spondylosis, unspecified	0
M4794	Spondylosis, unspecified	0
M4795	Spondylosis, unspecified	0
M4796	Spondylosis, unspecified	0
M4797	Spondylosis, unspecified	0
M4798	Spondylosis, unspecified	0
M4799	Spondylosis, unspecified	0

CONCEPT: Osteoporosis

ICD10_CODE	DESCRIPTION_SAIL	SENSITIVE_CODE
M80	Osteoporosis with pathological fracture	0
M800	Postmenopausal osteoporosis with pathological fracture	0
M8000	Postmenopausal osteoporosis with pathological fracture	0
M8001	Postmenopausal osteoporosis with pathological fracture	0
M8002	Postmenopausal osteoporosis with pathological fracture	0
M8003	Postmenopausal osteoporosis with pathological fracture	0
M8004	Postmenopausal osteoporosis with pathological fracture	0
M8005	Postmenopausal osteoporosis with pathological fracture	0
M8006	Postmenopausal osteoporosis with pathological fracture	0
M8007	Postmenopausal osteoporosis with pathological fracture	0
M8008	Postmenopausal osteoporosis with pathological fracture	0
M8009	Postmenopausal osteoporosis with pathological fracture	0
M801	Postoophorectomy osteoporosis with pathological fracture	0
M8010	Postoophorectomy osteoporosis with pathological fracture	0
M8011	Postoophorectomy osteoporosis with pathological fracture	0
M8012	Postoophorectomy osteoporosis with pathological fracture	0
M8013	Postoophorectomy osteoporosis with pathological fracture	0
M8014	Postoophorectomy osteoporosis with pathological fracture	0
M8015	Postoophorectomy osteoporosis with pathological fracture	0
M8016	Postoophorectomy osteoporosis with pathological fracture	0
M8017	Postoophorectomy osteoporosis with pathological fracture	0
M8018	Postoophorectomy osteoporosis with pathological fracture	0
M8019	Postoophorectomy osteoporosis with pathological fracture	0
M802	Osteoporosis of disuse with pathological fracture	0
M8020	Osteoporosis of disuse with pathological fracture	0
M8021	Osteoporosis of disuse with pathological fracture	0
M8022	Osteoporosis of disuse with pathological fracture	0
M8023	Osteoporosis of disuse with pathological fracture	0
M8024	Osteoporosis of disuse with pathological fracture	0
M8025	Osteoporosis of disuse with pathological fracture	0
M8026	Osteoporosis of disuse with pathological fracture	0
M8027	Osteoporosis of disuse with pathological fracture	0
M8028	Osteoporosis of disuse with pathological fracture	0
M8029	Osteoporosis of disuse with pathological fracture	0
M803	Postsurgical malabsorption osteoporosis with pathological fracture	0
M8030	Postsurgical malabsorption osteoporosis with pathological fracture	0
M8031	Postsurgical malabsorption osteoporosis with pathological fracture	0
M8032	Postsurgical malabsorption osteoporosis with pathological fracture	0
M8033	Postsurgical malabsorption osteoporosis with pathological fracture	0
M8034	Postsurgical malabsorption osteoporosis with pathological fracture	0
M8035	Postsurgical malabsorption osteoporosis with pathological fracture	0
M8036	Postsurgical malabsorption osteoporosis with pathological fracture	0
M8037	Postsurgical malabsorption osteoporosis with pathological fracture	0
M8038	Postsurgical malabsorption osteoporosis with pathological fracture	0
M8039	Postsurgical malabsorption osteoporosis with pathological fracture	0
M804	Drug-induced osteoporosis with pathological fracture	0
M8040	Drug-induced osteoporosis with pathological fracture	0
M8041	Drug-induced osteoporosis with pathological fracture	0
M8042	Drug-induced osteoporosis with pathological fracture	0
M8043	Drug-induced osteoporosis with pathological fracture	0
M8044	Drug-induced osteoporosis with pathological fracture	0
M8045	Drug-induced osteoporosis with pathological fracture	0
M8046	Drug-induced osteoporosis with pathological fracture	0
M8047	Drug-induced osteoporosis with pathological fracture	0
M8048	Drug-induced osteoporosis with pathological fracture	0
M8049	Drug-induced osteoporosis with pathological fracture	0
M805	Idiopathic osteoporosis with pathological fracture	0
M8050	Idiopathic osteoporosis with pathological fracture	0
M8051	Idiopathic osteoporosis with pathological fracture	0
M8052	Idiopathic osteoporosis with pathological fracture	0
M8053	Idiopathic osteoporosis with pathological fracture	0
M8054	Idiopathic osteoporosis with pathological fracture	0
M8055	Idiopathic osteoporosis with pathological fracture	0
M8056	Idiopathic osteoporosis with pathological fracture	0
M8057	Idiopathic osteoporosis with pathological fracture	0
M8058	Idiopathic osteoporosis with pathological fracture	0
M8059	Idiopathic osteoporosis with pathological fracture	0

M808	Other osteoporosis with pathological fracture	0
M8080	Other osteoporosis with pathological fracture	0
M8081	Other osteoporosis with pathological fracture	0
M8082	Other osteoporosis with pathological fracture	0
M8083	Other osteoporosis with pathological fracture	0
M8084	Other osteoporosis with pathological fracture	0
M8085	Other osteoporosis with pathological fracture	0
M8086	Other osteoporosis with pathological fracture	0
M8087	Other osteoporosis with pathological fracture	0
M8088	Other osteoporosis with pathological fracture	0
M8089	Other osteoporosis with pathological fracture	0
M809	Unspecified osteoporosis with pathological fracture	0
M8090	Unspecified osteoporosis with pathological fracture	0
M8091	Unspecified osteoporosis with pathological fracture	0
M8092	Unspecified osteoporosis with pathological fracture	0
M8093	Unspecified osteoporosis with pathological fracture	0
M8094	Unspecified osteoporosis with pathological fracture	0
M8095	Unspecified osteoporosis with pathological fracture	0
M8096	Unspecified osteoporosis with pathological fracture	0
M8097	Unspecified osteoporosis with pathological fracture	0
M8098	Unspecified osteoporosis with pathological fracture	0
M8099	Unspecified osteoporosis with pathological fracture	0
M81	Osteoporosis without pathological fracture	0
M810	Postmenopausal osteoporosis	0
M8100	Postmenopausal osteoporosis	0
M8101	Postmenopausal osteoporosis	0
M8102	Postmenopausal osteoporosis	0
M8103	Postmenopausal osteoporosis	0
M8104	Postmenopausal osteoporosis	0
M8105	Postmenopausal osteoporosis	0
M8106	Postmenopausal osteoporosis	0
M8107	Postmenopausal osteoporosis	0
M8108	Postmenopausal osteoporosis	0
M8109	Postmenopausal osteoporosis	0
M811	Postoophorectomy osteoporosis	0
M8110	Postoophorectomy osteoporosis	0
M8111	Postoophorectomy osteoporosis	0
M8112	Postoophorectomy osteoporosis	0
M8113	Postoophorectomy osteoporosis	0
M8114	Postoophorectomy osteoporosis	0
M8115	Postoophorectomy osteoporosis	0
M8116	Postoophorectomy osteoporosis	0
M8117	Postoophorectomy osteoporosis	0
M8118	Postoophorectomy osteoporosis	0
M8119	Postoophorectomy osteoporosis	0
M812	Osteoporosis of disuse	0
M8120	Osteoporosis of disuse	0
M8121	Osteoporosis of disuse	0
M8122	Osteoporosis of disuse	0
M8123	Osteoporosis of disuse	0
M8124	Osteoporosis of disuse	0
M8125	Osteoporosis of disuse	0
M8126	Osteoporosis of disuse	0
M8127	Osteoporosis of disuse	0
M8128	Osteoporosis of disuse	0
M8129	Osteoporosis of disuse	0
M813	Postsurgical malabsorption osteoporosis	0
M8130	Postsurgical malabsorption osteoporosis	0
M8131	Postsurgical malabsorption osteoporosis	0
M8132	Postsurgical malabsorption osteoporosis	0
M8133	Postsurgical malabsorption osteoporosis	0
M8134	Postsurgical malabsorption osteoporosis	0
M8135	Postsurgical malabsorption osteoporosis	0
M8136	Postsurgical malabsorption osteoporosis	0
M8137	Postsurgical malabsorption osteoporosis	0
M8138	Postsurgical malabsorption osteoporosis	0
M8139	Postsurgical malabsorption osteoporosis	0
M814	Drug-induced osteoporosis	0
M8140	Drug-induced osteoporosis	0
M8141	Drug-induced osteoporosis	0
M8142	Drug-induced osteoporosis	0
M8143	Drug-induced osteoporosis	0
M8144	Drug-induced osteoporosis	0
M8145	Drug-induced osteoporosis	0
M8146	Drug-induced osteoporosis	0
M8147	Drug-induced osteoporosis	0
M8148	Drug-induced osteoporosis	0
M8149	Drug-induced osteoporosis	0
M815	Idiopathic osteoporosis	0
M8150	Idiopathic osteoporosis	0
M8151	Idiopathic osteoporosis	0
M8152	Idiopathic osteoporosis	0
M8153	Idiopathic osteoporosis	0
M8154	Idiopathic osteoporosis	0
M8155	Idiopathic osteoporosis	0
M8156	Idiopathic osteoporosis	0
M8157	Idiopathic osteoporosis	0
M8158	Idiopathic osteoporosis	0
M8159	Idiopathic osteoporosis	0
M816	Localized osteoporosis [Lequesne]	0
M8160	Localized osteoporosis [Lequesne]	0
M8161	Localized osteoporosis [Lequesne]	0
M8162	Localized osteoporosis [Lequesne]	0
M8163	Localized osteoporosis [Lequesne]	0
M8164	Localized osteoporosis [Lequesne]	0
M8165	Localized osteoporosis [Lequesne]	0
M8166	Localized osteoporosis [Lequesne]	0
M8167	Localized osteoporosis [Lequesne]	0
M8168	Localized osteoporosis [Lequesne]	0
M8169	Localized osteoporosis [Lequesne]	0
M818	Other osteoporosis	0
M8180	Other osteoporosis	0
M8181	Other osteoporosis	0

M8182	Other osteoporosis	0
M8183	Other osteoporosis	0
M8184	Other osteoporosis	0
M8185	Other osteoporosis	0
M8186	Other osteoporosis	0
M8187	Other osteoporosis	0
M8188	Other osteoporosis	0
M8189	Other osteoporosis	0
M819	Osteoporosis, unspecified	0
M8190	Osteoporosis, unspecified	0
M8191	Osteoporosis, unspecified	0
M8192	Osteoporosis, unspecified	0
M8193	Osteoporosis, unspecified	0
M8194	Osteoporosis, unspecified	0
M8195	Osteoporosis, unspecified	0
M8196	Osteoporosis, unspecified	0
M8197	Osteoporosis, unspecified	0
M8198	Osteoporosis, unspecified	0
M8199	Osteoporosis, unspecified	0
M82	Osteoporosis in diseases classified elsewhere	0
M820	Osteoporosis in multiple myelomatosis	0
M8200	Osteoporosis in multiple myelomatosis	0
M8201	Osteoporosis in multiple myelomatosis	0
M8202	Osteoporosis in multiple myelomatosis	0
M8203	Osteoporosis in multiple myelomatosis	0
M8204	Osteoporosis in multiple myelomatosis	0
M8205	Osteoporosis in multiple myelomatosis	0
M8206	Osteoporosis in multiple myelomatosis	0
M8207	Osteoporosis in multiple myelomatosis	0
M8208	Osteoporosis in multiple myelomatosis	0
M8209	Osteoporosis in multiple myelomatosis	0
M821	Osteoporosis in endocrine disorders	0
M8210	Osteoporosis in endocrine disorders	0
M8211	Osteoporosis in endocrine disorders	0
M8212	Osteoporosis in endocrine disorders	0
M8213	Osteoporosis in endocrine disorders	0
M8214	Osteoporosis in endocrine disorders	0
M8215	Osteoporosis in endocrine disorders	0
M8216	Osteoporosis in endocrine disorders	0
M8217	Osteoporosis in endocrine disorders	0
M8218	Osteoporosis in endocrine disorders	0
M8219	Osteoporosis in endocrine disorders	0
M828	Osteoporosis in other diseases classified elsewhere	0
M8280	Osteoporosis in other diseases classified elsewhere	0
M8281	Osteoporosis in other diseases classified elsewhere	0
M8282	Osteoporosis in other diseases classified elsewhere	0
M8283	Osteoporosis in other diseases classified elsewhere	0
M8284	Osteoporosis in other diseases classified elsewhere	0
M8285	Osteoporosis in other diseases classified elsewhere	0
M8286	Osteoporosis in other diseases classified elsewhere	0
M8287	Osteoporosis in other diseases classified elsewhere	0
M8288	Osteoporosis in other diseases classified elsewhere	0
M8289	Osteoporosis in other diseases classified elsewhere	0

CONCEPT: Pancreatic disease

ICD10_CODE	DESCRIPTION_SAIL	SENSITIVE_CODE
K860	Alcohol-induced chronic pancreatitis	0
K861	Other chronic pancreatitis	0

CONCEPT: Paralysis

ICD10_CODE	DESCRIPTION_SAIL	SENSITIVE_CODE
G041	Tropical spastic paraplegia	0
G114	Hereditary spastic paraplegia	0
G800	Spastic quadriplegic cerebral palsy	0
G802	Spastic hemiplegic cerebral palsy	0
G81	Hemiplegia	0
G810	Flaccid hemiplegia	0
G811	Spastic hemiplegia	0
G819	Hemiplegia, unspecified	0
G82	Paraplegia and tetraplegia	0
G820	Flaccid paraplegia	0
G821	Spastic paraplegia	0
G822	Paraplegia, unspecified	0
G823	Flaccid tetraplegia	0
G824	Spastic tetraplegia	0
G825	Tetraplegia, unspecified	0

CONCEPT: Parkinson's disease

ICD10_CODE	DESCRIPTION_SAIL	SENSITIVE_CODE
F023	Dementia in Parkinson disease	0
G20	Parkinson disease	0

CONCEPT: Plasmacell

ICD10_CODE	DESCRIPTION_SAIL	SENSITIVE_CODE
C882	Other heavy chain disease	0
C90	Multiple myeloma and malignant plasma cell neoplasms	0
C900	Multiple myeloma	0
C901	Plasma cell leukaemia	0
C902	Extramedullary plasmacytoma	0
C903	Solitary plasmacytoma	0
M820	Osteoporosis in multiple myelomatosis	0
M8200	Osteoporosis in multiple myelomatosis	0
M8201	Osteoporosis in multiple myelomatosis	0
M8202	Osteoporosis in multiple myelomatosis	0
M8203	Osteoporosis in multiple myelomatosis	0
M8204	Osteoporosis in multiple myelomatosis	0
M8205	Osteoporosis in multiple myelomatosis	0
M8206	Osteoporosis in multiple myelomatosis	0
M8207	Osteoporosis in multiple myelomatosis	0
M8208	Osteoporosis in multiple myelomatosis	0

M8209	Osteoporosis in multiple myelomatosis	0

CONCEPT: PMR (Polymyalgia reheumatica)

ICD10_CODE	DESCRIPTION_SAIL	SENSITIVE_CODE
M315	Giant cell arteritis with polymyalgia rheumatica	0
M353	Polymyalgia rheumatica	0

CONCEPT: Primary Lung Cancer

ICD10_CODE	DESCRIPTION_SAIL	SENSITIVE_CODE
C33	Malignant neoplasm of trachea	0
C34	Malignant neoplasm of bronchus and lung	0
C39	Malignant neoplasm of other and ill-defined sites in the respiratory system and intrathoracic organs	0

CONCEPT: Prostate Cancer

ICD10_CODE	DESCRIPTION_SAIL	SENSITIVE_CODE
C61	Malignant neoplasm of prostate	0

CONCEPT: Psoriasis

ICD10_CODE	DESCRIPTION_SAIL	SENSITIVE_CODE
L40	Psoriasis	0
L400	Psoriasis vulgaris	0
L401	Generalized pustular psoriasis	0
L404	Guttate psoriasis	0
L405	Arthropathic psoriasis	0
L408	Other psoriasis	0
L409	Psoriasis, unspecified	0

CONCEPT: Psoriasis Arthritis

ICD10_CODE	DESCRIPTION_SAIL	SENSITIVE_CODE
L405	Arthropathic psoriasis	0
M07	Psoriatic and enteropathic arthropathies	0
M070	Distal interphalangeal psoriatic arthropathy	0
M0700	Distal interphalangeal psoriatic arthropathy	0
M0704	Distal interphalangeal psoriatic arthropathy	0
M0707	Distal interphalangeal psoriatic arthropathy	0
M0709	Distal interphalangeal psoriatic arthropathy	0
M071	Arthritis mutilans	0
M0710	Arthritis mutilans	0
M0711	Arthritis mutilans	0
M0712	Arthritis mutilans	0
M0713	Arthritis mutilans	0
M0714	Arthritis mutilans	0
M0715	Arthritis mutilans	0
M0716	Arthritis mutilans	0
M0717	Arthritis mutilans	0
M0718	Arthritis mutilans	0
M0719	Arthritis mutilans	0
M073	Other psoriatic arthropathies	0
M0730	Other psoriatic arthropathies	0
M0731	Other psoriatic arthropathies	0
M0732	Other psoriatic arthropathies	0
M0733	Other psoriatic arthropathies	0
M0734	Other psoriatic arthropathies	0
M0735	Other psoriatic arthropathies	0
M0736	Other psoriatic arthropathies	0
M0737	Other psoriatic arthropathies	0
M0738	Other psoriatic arthropathies	0
M0739	Other psoriatic arthropathies	0
M090	Juvenile arthritis in psoriasis	0
M0900	Juvenile arthritis in psoriasis	0
M0901	Juvenile arthritis in psoriasis	0
M0902	Juvenile arthritis in psoriasis	0
M0903	Juvenile arthritis in psoriasis	0
M0904	Juvenile arthritis in psoriasis	0
M0905	Juvenile arthritis in psoriasis	0
M0906	Juvenile arthritis in psoriasis	0
M0907	Juvenile arthritis in psoriasis	0
M0908	Juvenile arthritis in psoriasis	0
M0909	Juvenile arthritis in psoriasis	0

CONCEPT: PTSD (Post-traumatic stress disorders)

ICD10_CODE	DESCRIPTION_SAIL	SENSITIVE_CODE
F431	Post-traumatic stress disorder	0

CONCEPT: Pheripheral vascular disease

ICD10_CODE	DESCRIPTION_SAIL	SENSITIVE_CODE
I70	Atherosclerosis	0
I700	Atherosclerosis of aorta	0
I7000	Atherosclerosis of aorta	0
I7001	Atherosclerosis of aorta	0
I701	Atherosclerosis of renal artery	0
I7010	Atherosclerosis of renal artery	0
I7011	Atherosclerosis of renal artery	0
I702	Atherosclerosis of arteries of extremities	0
I7020	Atherosclerosis of arteries of extremities	0
I7021	Atherosclerosis of arteries of extremities	0
I708	Atherosclerosis of other arteries	0
I7080	Atherosclerosis of other arteries	0
I7081	Atherosclerosis of other arteries	0
I709	Generalized and unspecified atherosclerosis	0
I7090	Generalized and unspecified atherosclerosis	0
I7091	Generalized and unspecified atherosclerosis	0
I73	Other peripheral vascular diseases	0
I730	Raynaud syndrome	0
I731	Thromboangiitis obliterans [Buerger]	0
I738	Other specified peripheral vascular diseases	0
I739	Peripheral vascular disease, unspecified	0
I792	Peripheral angiopathy in diseases classified elsewhere	0

CONCEPT: Renal transplant

ICD10_CODE	DESCRIPTION_SAIL	SENSITIVE_CODE
T861	Kidney transplant failure and rejection	0
Z940	Kidney transplant status	0

CONCEPT: Rheumatoid Arthritis

ICD10_CODE	DESCRIPTION_SAIL	SENSITIVE_CODE
J990	Rheumatoid lung disease	0
M05	Seropositive rheumatoid arthritis	0
M050	Felty syndrome	0
M0500	Felty syndrome	0
M0501	Felty syndrome	0
M0502	Felty syndrome	0
M0503	Felty syndrome	0
M0504	Felty syndrome	0
M0505	Felty syndrome	0
M0506	Felty syndrome	0
M0507	Felty syndrome	0
M0508	Felty syndrome	0
M0509	Felty syndrome	0
M051	Rheumatoid lung disease	0
M0510	Rheumatoid lung disease	0
M0511	Rheumatoid lung disease	0
M0512	Rheumatoid lung disease	0
M0513	Rheumatoid lung disease	0
M0514	Rheumatoid lung disease	0
M0515	Rheumatoid lung disease	0
M0516	Rheumatoid lung disease	0
M0517	Rheumatoid lung disease	0
M0518	Rheumatoid lung disease	0
M0519	Rheumatoid lung disease	0
M052	Rheumatoid vasculitis	0
M0520	Rheumatoid vasculitis	0
M0521	Rheumatoid vasculitis	0
M0522	Rheumatoid vasculitis	0
M0523	Rheumatoid vasculitis	0
M0524	Rheumatoid vasculitis	0
M0525	Rheumatoid vasculitis	0
M0526	Rheumatoid vasculitis	0
M0527	Rheumatoid vasculitis	0
M0528	Rheumatoid vasculitis	0
M0529	Rheumatoid vasculitis	0
M053	Rheumatoid arthritis with involvement of other organs and systems	0
M0530	Rheumatoid arthritis with involvement of other organs and systems	0
M0531	Rheumatoid arthritis with involvement of other organs and systems	0
M0532	Rheumatoid arthritis with involvement of other organs and systems	0
M0533	Rheumatoid arthritis with involvement of other organs and systems	0
M0534	Rheumatoid arthritis with involvement of other organs and systems	0
M0535	Rheumatoid arthritis with involvement of other organs and systems	0
M0536	Rheumatoid arthritis with involvement of other organs and systems	0
M0537	Rheumatoid arthritis with involvement of other organs and systems	0
M0538	Rheumatoid arthritis with involvement of other organs and systems	0
M0539	Rheumatoid arthritis with involvement of other organs and systems	0
M058	Other seropositive rheumatoid arthritis	0
M0580	Other seropositive rheumatoid arthritis	0
M0581	Other seropositive rheumatoid arthritis	0
M0582	Other seropositive rheumatoid arthritis	0
M0583	Other seropositive rheumatoid arthritis	0
M0584	Other seropositive rheumatoid arthritis	0
M0585	Other seropositive rheumatoid arthritis	0
M0586	Other seropositive rheumatoid arthritis	0
M0587	Other seropositive rheumatoid arthritis	0
M0588	Other seropositive rheumatoid arthritis	0
M0589	Other seropositive rheumatoid arthritis	0
M059	Seropositive rheumatoid arthritis, unspecified	0
M0590	Seropositive rheumatoid arthritis, unspecified	0
M0591	Seropositive rheumatoid arthritis, unspecified	0
M0592	Seropositive rheumatoid arthritis, unspecified	0
M0593	Seropositive rheumatoid arthritis, unspecified	0
M0594	Seropositive rheumatoid arthritis, unspecified	0
M0595	Seropositive rheumatoid arthritis, unspecified	0
M0596	Seropositive rheumatoid arthritis, unspecified	0
M0597	Seropositive rheumatoid arthritis, unspecified	0
M0598	Seropositive rheumatoid arthritis, unspecified	0
M0599	Seropositive rheumatoid arthritis, unspecified	0
M06	Other rheumatoid arthritis	0
M060	Seronegative rheumatoid arthritis	0
M0600	Seronegative rheumatoid arthritis	0
M0601	Seronegative rheumatoid arthritis	0
M0602	Seronegative rheumatoid arthritis	0
M0603	Seronegative rheumatoid arthritis	0
M0604	Seronegative rheumatoid arthritis	0
M0605	Seronegative rheumatoid arthritis	0
M0606	Seronegative rheumatoid arthritis	0
M0607	Seronegative rheumatoid arthritis	0
M0608	Seronegative rheumatoid arthritis	0
M0609	Seronegative rheumatoid arthritis	0
M062	Rheumatoid bursitis	0
M0620	Rheumatoid bursitis	0
M0621	Rheumatoid bursitis	0
M0622	Rheumatoid bursitis	0
M0623	Rheumatoid bursitis	0
M0624	Rheumatoid bursitis	0
M0625	Rheumatoid bursitis	0
M0626	Rheumatoid bursitis	0
M0627	Rheumatoid bursitis	0
M0628	Rheumatoid bursitis	0
M0629	Rheumatoid bursitis	0
M063	Rheumatoid nodule	0
M0630	Rheumatoid nodule	0
M0631	Rheumatoid nodule	0

M0632	Rheumatoid nodule	0
M0633	Rheumatoid nodule	0
M0634	Rheumatoid nodule	0
M0635	Rheumatoid nodule	0
M0636	Rheumatoid nodule	0
M0637	Rheumatoid nodule	0
M0638	Rheumatoid nodule	0
M0639	Rheumatoid nodule	0
M068	Other specified rheumatoid arthritis	0
M0680	Other specified rheumatoid arthritis	0
M0681	Other specified rheumatoid arthritis	0
M0682	Other specified rheumatoid arthritis	0
M0683	Other specified rheumatoid arthritis	0
M0684	Other specified rheumatoid arthritis	0
M0685	Other specified rheumatoid arthritis	0
M0686	Other specified rheumatoid arthritis	0
M0687	Other specified rheumatoid arthritis	0
M0688	Other specified rheumatoid arthritis	0
M0689	Other specified rheumatoid arthritis	0
M069	Rheumatoid arthritis, unspecified	0
M0690	Rheumatoid arthritis, unspecified	0
M0691	Rheumatoid arthritis, unspecified	0
M0692	Rheumatoid arthritis, unspecified	0
M0693	Rheumatoid arthritis, unspecified	0
M0694	Rheumatoid arthritis, unspecified	0
M0695	Rheumatoid arthritis, unspecified	0
M0696	Rheumatoid arthritis, unspecified	0
M0697	Rheumatoid arthritis, unspecified	0
M0698	Rheumatoid arthritis, unspecified	0
M0699	Rheumatoid arthritis, unspecified	0

CONCEPT: Schizophrenia

ICD10_CODE	DESCRIPTION_SAIL	SENSITIVE_CODE
F062	Organic delusional [schizophrenia-like] disorder	0
F20	Schizophrenia	0
F200	Paranoid schizophrenia	0
F201	Hebephrenic schizophrenia	0
F202	Catatonic schizophrenia	0
F203	Undifferentiated schizophrenia	0
F204	Post-schizophrenic depression	0
F205	Residual schizophrenia	0
F206	Simple schizophrenia	0
F208	Other schizophrenia	0
F209	Schizophrenia, unspecified	0
F230	Acute polymorphic psychotic disorder without symptoms of schizophrenia	0
F231	Acute polymorphic psychotic disorder with symptoms of schizophrenia	0
F232	Acute schizophrenia-like psychotic disorder	0

CONCEPT: Skin Cancer

ICD10_CODE	DESCRIPTION_SAIL	SENSITIVE_CODE
C43	Malignant melanoma of skin	0
C430	Malignant neoplasm: Malignant melanoma of lip	0
C431	Malignant neoplasm: Malignant melanoma of eyelid, including canthus	0
C432	Malignant neoplasm: Malignant melanoma of ear and external auricular canal	0
C433	Malignant neoplasm: Malignant melanoma of other and unspecified parts of face	0
C434	Malignant neoplasm: Malignant melanoma of scalp and neck	0
C435	Malignant neoplasm: Malignant melanoma of trunk	0
C436	Malignant neoplasm: Malignant melanoma of upper limb, including shoulder	0
C437	Malignant neoplasm: Malignant melanoma of lower limb, including hip	0
C438	Malignant neoplasm: Overlapping malignant melanoma of skin	0
C439	Malignant neoplasm: Malignant melanoma of skin, unspecified	0
C44	Other malignant neoplasms of skin	0
C440	Malignant neoplasm: Skin of lip	0
C441	Malignant neoplasm: Skin of eyelid, including canthus	0
C442	Malignant neoplasm: Skin of ear and external auricular canal	0
C443	Malignant neoplasm: Skin of other and unspecified parts of face	0
C444	Malignant neoplasm: Skin of scalp and neck	0
C445	Malignant neoplasm: Skin of trunk	0
C446	Malignant neoplasm: Skin of upper limb, including shoulder	0
C447	Malignant neoplasm: Skin of lower limb, including hip	0
C448	Malignant neoplasm: Overlapping lesion of skin	0
C449	Malignant neoplasm: Malignant neoplasm of skin, unspecified	0
C460	Kaposi sarcoma of skin	0
C792	Secondary malignant neoplasm of skin	0
C826	Cutaneous follicle centre lymphoma	0
C848	Cutaneous T-cell lymphoma, unspecified	0
D485	Neoplasm of uncertain or unknown behaviour: Skin	0

CONCEPT: Stroke

ICD10_CODE	DESCRIPTION_SAIL	SENSITIVE_CODE
G463	Brain stem stroke syndrome	0
G464	Cerebellar stroke syndrome	0
I60	Subarachnoid haemorrhage	0
I600	Subarachnoid haemorrhage from carotid siphon and bifurcation	0
I601	Subarachnoid haemorrhage from middle cerebral artery	0
I602	Subarachnoid haemorrhage from anterior communicating artery	0
I603	Subarachnoid haemorrhage from posterior communicating artery	0
I604	Subarachnoid haemorrhage from basilar artery	0
I605	Subarachnoid haemorrhage from vertebral artery	0
I606	Subarachnoid haemorrhage from other intracranial arteries	0
I607	Subarachnoid haemorrhage from intracranial artery, unspecified	0
I608	Other subarachnoid haemorrhage	0
I609	Subarachnoid haemorrhage, unspecified	0
I61	Intracerebral haemorrhage	0
I610	Intracerebral haemorrhage in hemisphere, subcortical	0
I611	Intracerebral haemorrhage in hemisphere, cortical	0
I612	Intracerebral haemorrhage in hemisphere, unspecified	0
I613	Intracerebral haemorrhage in brain stem	0
I614	Intracerebral haemorrhage in cerebellum	0
I615	Intracerebral haemorrhage, intraventricular	0
I616	Intracerebral haemorrhage, multiple localized	0

I618	Other intracerebral haemorrhage	0
I619	Intracerebral haemorrhage, unspecified	0
I64	Stroke, not specified as haemorrhage or infarction	0
I672	Cerebral atherosclerosis	0
I679	Cerebrovascular disease, unspecified	0
I691	Sequelae of intracerebral haemorrhage	0

CONCEPT: Systemic Lupus Erythematosus

ICD10_CODE	DESCRIPTION_SAIL	SENSITIVE_CODE
L93	Lupus erythematosus	0
L931	Subacute cutaneous lupus erythematosus	0
L932	Other local lupus erythematosus	0
M32	Systemic lupus erythematosus	0
M320	Drug-induced systemic lupus erythematosus	0
M321	Systemic lupus erythematosus with organ or system involvement	0
M328	Other forms of systemic lupus erythematosus	0
M329	Systemic lupus erythematosus, unspecified	0

CONCEPT: Systemic sclerosis

ICD10_CODE	DESCRIPTION_SAIL	SENSITIVE_CODE
L940	Localized scleroderma [morphea]	0
L941	Linear scleroderma	0
M34	Systemic sclerosis	0
M340	Progressive systemic sclerosis	0
M342	Systemic sclerosis induced by drugs and chemicals	0
M348	Other forms of systemic sclerosis	0
M349	Systemic sclerosis, unspecified	0

CONCEPT: Thalassaemia

ICD10_CODE	DESCRIPTION_SAIL	SENSITIVE_CODE
D56	Thalassaemia	0
D560	Alpha thalassaemia	0
D561	Beta thalassaemia	0
D562	Delta-beta thalassaemia	0
D563	Thalassaemia trait	0
D564	Hereditary persistence of fetal haemoglobin [HPFH]	0
D568	Other thalassaemias	0
D569	Thalassaemia, unspecified	0

CONCEPT: TIA (Transient ischaemic attack)

ICD10_CODE	DESCRIPTION_SAIL	SENSITIVE_CODE
G45	Transient cerebral ischaemic attacks and related syndromes	0
G453	Amaurosis fugax	0
G454	Transient global amnesia	0
G458	Other transient cerebral ischaemic attacks and related syndromes	0
G459	Transient cerebral ischaemic attack, unspecified	0

CONCEPT: Viral Hepatitis

ICD10_CODE	DESCRIPTION_SAIL	SENSITIVE_CODE
B16	Acute hepatitis B	1
B160	Acute hepatitis B with delta-agent (coinfection) with hepatic coma	1
B161	Acute hepatitis B with delta-agent (coinfection) without hepatic coma	1
B162	Acute hepatitis B without delta-agent with hepatic coma	1
B169	Acute hepatitis B without delta-agent and without hepatic coma	1
B170	Acute delta-(super)infection of hepatitis B carrier	1
B171	Acute hepatitis C	1
B180	Chronic viral hepatitis B with delta-agent	1
B181	Chronic viral hepatitis B without delta-agent	1
B182	Chronic viral hepatitis C	1

CONCEPT: VTD (Venous thrombotic disease)

ICD10_CODE	DESCRIPTION_SAIL	SENSITIVE_CODE
I802	Phlebitis and thrombophlebitis of other deep vessels of lower extremities	0
I822	Embolism and thrombosis of vena cava	0
I828	Embolism and thrombosis of other specified veins	0
I870	Postthrombotic syndrome	0
O223	Deep phlebothrombosis in pregnancy	0
O225	Cerebral venous thrombosis in pregnancy	0
O871	Deep phlebothrombosis in the puerperium	0
O873	Cerebral venous thrombosis in the puerperium	0
