# Supplementary material for: Cohort profile: creation of the SAIL MELD-B e-cohort (SMC) and SAIL MELD-B children and young adult e-cohort (SMYC) to investigate the lived experience of the ‘burdensomeness’ of multimorbidity
Source: BMJ Open. 2025 Jan 7;15(1):e087946. doi: 10.1136/bmjopen-2024-087946 (PMC11792564; doi:10.1136/bmjopen-2024-087946)
Supplement: Supplementary file 3 [file bmjopen-2024-087946.reviewer_comments.pdf]

PEER REVIEW HISTORY

BMJ Open publishes all reviews undertaken for accepted manuscripts. Reviewers are asked to complete a checklist review form and are provided with free text boxes to elaborate on their assessment. These free text comments are reproduced below.

ARTICLE DETAILS

Title (Provisional)

Cohort profile: the creation of the SAIL MELD-B e-cohort (SMC) and SAIL MELD-B children and young adult e-cohort (SMYC) to investigate the lived experience of the 'burdensomeness' of multimorbidity

Authors

Chiovoloni, Roberta; Dylag, Jakub J; Alwan, Nisreen A; Berrington, Ann; Boniface, Michael; Fair, Nic; Holland, Emilia; Hoyle, Rebecca; Shiranirad, Mozhdeh; Stannard, Sebastian; Zlatev, Zlatko; Owen, Rhiannon K; Fraser, Simon; Akbari, Ashley

---

VERSION 1 - REVIEW

**Reviewer**	**1**
**Name**	**Heikkila, Katriina**
**Affiliation**	**Finnish Institute for Health and Welfare**
**Date**	**17-May-2024**
**COI**	**None.**

General comment

Thank you for the opportunity to review this interesting manuscript. The work setting up the e-cohorts is impressive and the cohorts represent a much-needed data resources in an important research area. With this in mind, the cohort profile would benefit from revisions to make the paper more easily readable and focused. Please see my specific comments below.

Major comments

1. It would be helpful to incorporate a figure/figures to describe the cohorts design features (section 2.2.): these would help the reader to understand the cohort timelines, inclusion and exclusion criteria and the data sources used to build up the cohorts.

2. In a similar vein, the authors could consider including a summary figure to complement the description of the cohort evolution (section 3.1.1). (I appreciate the details are shown in the supplement.)

3. Section 2.3, on concept curation pipeline, should be revised to clarify the extent to which this is work in progress. In its current form, this section is not very clear on which concepts have already been developed and whether these can be, or already have been, used in research studies.

4. It would be worth revising the text throughout the paper so it does not repeat information that is presented in tables or figures. For example, section 3.2 could be shortened by omitting the information shown in figure 4.

5. The authors should reorganise the text to correspond to the sub-headings.

a. For example, paragraph 2, p.6, on challenges of utilising routinely collected health records data would be better placed in the “Strengths and limitations”-section.

b. The second and third paragraphs under “2. Cohort description” should be presented in the Discussion-section: these paragraphs describe the rationale for setting up e-cohorts to study multimorbidity, not SMC and SMYC cohorts per se.

Minor comments

1. It would be best to avoid using abbreviations as much as possible: e.g. section2.2 on cohort design is heavy on abbreviations, which make the information difficult to take in. It would be clearer if the names of the multiple data sources were written out in full throughout the text. Not all readers will be familiar with WDSD, ADDE, and NCC, for instance.

2. The authors should ensure that all terminology and spellings are used concisely throughout the manuscript. At the moment, for example, “upset plots” and “Upset plots” are used interchangeably.

3. Ethics considerations are described in a fairly general terms and mainly in relation to the SAIL data bank. It would be worth revising this to focus more on specific ethics issues relating to the e-cohorts (e.g. relevant legal requirements and other processes relating to individual consent, ethics committee approval, data linkage, data storage and analysis).

**Reviewer**	**2**
**Name**	**Berntsen, Gro**
**Affiliation**	**Norwegian centre for E-health Research, Integrated care**
**Date**	**26-Jun-2024**
**COI**	**None**

Review:

Cohort profile for the creation of the SAIL MELD-B e-cohort (SMC) and SAIL MELD-B children and Young adult e-cohort (SMYC)

This is a paper submitted as a "cohort profile" which details the establishment of the cohorts and key variables for the *"Multidisciplinary Ecosystem to study Lifecourse Determinants and Prevention of Early-onset Burdensome Multimorbidity*" (MELD-B) collaboration, within the Secure Anonymised Information Linkage (SAIL) Databank.
The authors describe which datasources that are merged within SAIL, which concepts they wish to explore, and in extensive appendix they publish exactly which variable values that contribute to some central disease concepts.

It is of great importance that trusted research environments like SAIL are created, and that cohorts can be defined within such e-repositories. These have a tremendous effect on lowering the barriers to important research projects. Like wise, it is commendable that the MELD-B project publish openly their operationalization of the conditions they include.

The paper provides a description of : the title of the collaboration, what data sources are included, the subsequent cohort identification and the operationalization of 83 morbidity concepts.

Major comments:

What research questions can this cohort answer?

There is no doubt that the questions of causes of multimorbidity are important, and that databanks such as this are necessary. While the importance of such datasets can not be overstated, the paper explain some of the general themes that are interesting in multimorbidity studies, such as patterns of morbidity occurrence, treatment patterns, social inequities in broad overarching themes etc.

While it is important to describe accurately what was done, so that it can be referenced in the methods section of a future research paper, it is difficult to understand whether the choices made here are appropriate. Choices of method should carry forward from the research question. With such a general description of potential RQs, it is also difficult to assess the utility of the cohort. That said, the databank seems to be extraordinarily comprehensive, and as such a rich and important tool for researchers in this field.

Some choices are described, but there is little information as to why certain choices were made, and what considerations guided these decisions, and if any, what impact such choices might have on the potential research areas. I challenge the authors to describe in a stepwise manner the exact nature of their imagined future research, which concepts needed defining, whether their concepts are supported by other published operationalizations, and why certain choices were made.

Background – other similar cohorts, in similar infrastructure?

The paper does not give an overview of any other similar or overlapping cohorts that might be relevant for the field, and how this cohort is different from any other population cohorts.

Most older cohorts are not couched in a Trusted research Environment, but just how unique this TER-initiative is at this point in time is not discussed.

Concepts

The authors refer to the most simple definition of multimorbidity: two long-term simultaneous conditions. However, there is also common agreement that complex multimorbidity, is the most important to understand, i.e. multimorbidity that has some kind of impact the patient's life in terms of burden of symptoms, treatments, follow-up etc. Given that this project seeks to look at burdensomeness, I would expect them to be interested in complex multimorbidity. However, the authors do not reflect upon the difference between simple and complex multimorbidity, or explain if complexity is something they are trying to capture and define.

Also, the following concepts are key to the research question, but are not described at all, only mentioned in passing:

What are the burdensomeness concepts? The cohort is established to study "burdensomeness", and the authors describe that this term has been operationalized. This could be a potential original contribution, but the term remains undefined and un-operationalized.

What is the concept of "sensitive" codes? There is an identification of certain diagnosis codes as "sensitive" in the appendix. Most of them are contagious diseases that are linked to stigma. However, I am wondering why we should not learn more about multimorbidity which includes such socially challenging conditions? Is it not even more important to understand the patterns in marginalized and vulnerable populations? Is this the result of a privacy concern? Was this a requirement from the ethics board?

Establishing the e-cohort:

Certain choices were made but not argued: "*Note that if a person has an available maternal record but the mother is not included in SMC, we considered this individual as not having a maternal record (n = 1248, 0.1%) thus, all the MAT_ALF linked to the SMYC form a subset of the SMC*".
If I understand this correctly, this excludes a set of persons whose mother was not included in the larger cohort. This would probably be persons who lived with their father, were orphaned, migrants without parents? Many of these would represent vulnerable populations. While the n is small, it might be a very important group to study. Thus, such an exclusion should be underpinned by arguments. My apologies if I misunderstood, in which case you might want to clarify the text.

Cohort censoring:

In 2.2 the authors write: *"As a longitudinal e-cohort, the number of individuals will change throughout the study as they can leave or join the e-cohort at any time during the study period."* Which leads me o believe that individuals can re-enter the cohort if they leave Wales and then come back.
Later the authors write: *"Note that once an individual meets one of the censorship criteria, they are not allowed to re-enter the e-cohort, see Supplementary".* Which indicates that a person can not reenter the cohort. There may be good reasons for such a choice, but the authors do not provide any insights into their reasoning. Again, persons who move around may differ in important ways, from those who do not, thus the choice should be underpinned by some logic.

Anonymity.

The authors make clear that no one organization can unlock the identity information linked to the individualized study-id. However, to be fully anonymous, it must not be possible to "backwards identify" a person: I.e. if you combine a number of characteristics with information found elsewhere (eg social media), you may still identify the person. Also if there is a key between the study-id- and the identifying information, which allows unlocking the identity of a person under given circumstances (i.e. – need for data quality checks), the data can not be called anonymous. Individualized data, linked by the same study id is pseudonymous. Could the authors describe whether these two forms of re-identification could be theoretically possible?

References

Some of the references could not be accessed by this reviewer, as the URLs were either "404 -page not found", or they were behind a log-in barrier. References should only include souces that can be accessed by the general academic reader.

Ex:

- 53] J. Dylag, R. Chiovoloni, A. Akbari, S. Fraser, M. Boniface, A Tool for Automating the Curation of Medical Concepts derived from Coding Lists, 2024 (n.d.). https://git.soton.ac.uk/meld/meldb/concepts-processing (accessed April 22, 2024).

- All codes and scripts used in this study are available for others to access here: https://github.com/SwanseaUniversityDataScience/1377-MELD_B-CohortCuration

Minor comments:

A few acronyms that are not explained: Example: WIND, MAT_ALF,

The Flow-chart of the development of the two cohorts: In the SMYC chart, the 1248 individuals excluded because the maternal record is missing, are not represented.

The WIMD -quintiles – e.g. 20%-tiles, in table one, is marked as approx. 11%, which is illogical. This probably relates to the fact that table 1 shows characteristics at baseline, at the cohort start of follow-up date, i.e. 2,99 mill. To make the table consistent – either provide baseline characteristics for everyone, upon entry into the cohort, no matter when they entered,(N=5,2mill), or at baseline (N=2,9 mill). Do not mix the two.

Study end-point – is a confusing term. I first thought this was an outcome which would cause censoring. Then I understood study end point was synonymous to “end of follow-up”. Consider changing to “end of follow-up”.

VERSION 1 - AUTHOR RESPONSE

Reviewer1: Dr Katriina Heikkila, Finnish Institute for Health and Welfare

Major comments

1. *It would be helpful to incorporate a figure/figures to describe the cohorts design features (section 2.2.): these would help the reader to understand the cohort timelines, inclusion and exclusion criteria and the data sources used to build up the cohorts.*
   We have added a reference to Figure 2 in Section 2.2 to enhance clarity regarding cohort design features.
2. *In a similar vein, the authors could consider including a summary figure to complement the description of the cohort evolution (section 3.1.1). (I appreciate the details are shown in the supplement.)*
   Thanks for this suggestion. We moved Figures from the supplementary files to the main text to address this, see Fig.4 and Fig.4 in the paper. 2:
3. *Section 2.3, on concept curation pipeline, should be revised to clarify the extent to which this is work in progress. In its current form, this section is not very clear on which concepts have already been developed and whether these can be, or already have been, used in research studies.*

   We have added clarifying sentences in Sections 2.3 and 2.3.1 to indicate which burdensomeness concepts have been developed so far and explain how they will be used in future research studies.
4. *It would be worth revising the text throughout the paper so it does not repeat information that is presented in tables or figures. For example, section 3.2 could be shortened by omitting the information shown in figure 4.*

   We have addressed this point by removing text that repeated information already presented in tables and figures. Please see the updates in Section 3.2 as an example.
5. *The authors should reorganise the text to correspond to the sub-headings.*
   a. *For example, paragraph 2, p.6, on challenges of utilising routinely collected health records data would be better placed in the “Strengths and limitations”-section.*
   b. *The second and third paragraphs under “2. Cohort description” should be presented in the Discussion-section: these paragraphs describe the rationale for setting up e-cohorts to study multimorbidity, not SMC and SMYC cohorts per se.*

   Thank you for the suggestion, we moved the paragraph on the challenges of using routinely collected data to the “strength and limitations” section, and we moved the paragraphs mentioned in point b. in the Introduction.

Minor comments

6. *It would be best to avoid using abbreviations as much as possible: e.g. section2.2 on cohort design is heavy on abbreviations, which make the information difficult to take in. It would be clearer if the names of the multiple data sources were written out in full throughout the text. Not all readers will be familiar with WDSD, ADDE, and NCC, for instance.*

   We reduced the number of times abbreviations are used throughout the paper, for example in Sec. 2.2.

7. *The authors should ensure that all terminology and spellings are used concisely throughout the manuscript. At the moment, for example, "upset plots" and "Upset plots" are used interchangeably.*

   We checked and corrected all the different spellings we found.

8. Ethics considerations are described in a fairly general terms and mainly in relation to the SAIL data bank. It would be worth revising this to focus more on specific ethics issues relating to the e-cohorts (e.g. relevant legal requirements and other processes relating to individual consent, ethics committee approval, data linkage, data storage and analysis).

   Thanks for this, we updated this section. Just to be aware, consent is not the legal basis by which SAIL data are accessed. All data are linked, stored, accessed and analysed within the SAIL Databank Trusted Research Environment; see "Data availability statement" section for further details as confirmed with the SAIL TRE team.

Reviewer2: Prof. Gro Berntsen, Norwegian centre for E-health Research

Major comments:

1. *What research questions can this cohort answer? There is no doubt that the questions of causes of multimorbidity are important, and that databanks such as this are necessary. While the importance of such datasets can not be overstated, the paper explain some of the general themes that are interesting in multimorbidity studies, such as patterns of morbidity occurrence, treatment patterns, social inequities in broad overarching themes etc. While it is important to describe accurately what was done, so that it can be referenced in the methods section of a future research paper, it is difficult to understand whether the choices made here are appropriate. Choices of method should carry forward from the research question. With such a general description of potential RQs, it is also difficult to assess the utility of the cohort. That said, the databank seems to be extraordinarily comprehensive, and as such a rich and important tool for researchers in this field. Some choices are described, but there is little information as to why certain choices were made, and what considerations guided these decisions, and if any, what impact such choices might have on the potential research areas. I challenge the authors to describe in a stepwise manner the exact nature of their imagined future research, which concepts needed defining, whether their concepts are supported by other published operationalisations, and why certain choices were made.*

   Thank you for these insightful comments. In Section 1, we outlined the primary aim of the MELD-B project, which is to "develop a deeper understanding of the lived experience of 'burdensomeness' in multimorbidity, identify new clusters of burdensome MLTC-M, explore their key early-life risk factors, map trajectories across the life course towards these burdensome clusters (with a focus on individuals under 65), and model prevention scenarios to inform policy." and we reference [35], where the overarching aims of the MELD-B consortium are discussed in greater detail.

   In the same section, we added a couple of sentences to expand on the research questions that the MELD-B consortia aims to answer. These research questions are built around several key areas of inquiry:

1. **Clustering individuals based on burdensomeness indicators – What burdensomeness indicators tend to cluster together in individuals? Analysing the determinants of these burdensomeness clusters – Once clusters are identified, can we determine common factors (e.g., socioeconomic, biological, environmental, or health inequalities) that explain their formation?**
2. **Examining the sequence of acquisition of burdensomeness features - Is there a recognisable pattern in how burdensome features accumulate? Which burdensomeness indicators tend to appear before others?**
3. **Identifying early-life (predictors of health outcomes and mediators across the life course such as childhood obesity – how much can early-life risk factors (e.g. childhood socioeconomic conditions, parents' health, add others), in children/young people predict certain health outcomes later in life? Analysing the sequence of sentinel conditions and subsequent accrual of burden – How does the first long-term condition in an individual's life affect their risk of developing more burdensome MLTC-M over time?**

   To ensure these questions are addressed comprehensively, the SMC cohort was designed with broad generalizability, making it adaptable for other researchers within the MELD-B

project to explore these and other related research questions. The SMYC cohort is focused on addressing questions about early-life risk factors, as it is specific to younger individuals.

The concept curation pipeline we developed ensures flexibility and adaptability allowing for the reproducible implementation of concepts to be curated across the different research questions.

The burdensomeness concepts (as detailed in Sections 2.3.1 and 3.2) were selected by the MELD-B clinical domain experts based on both their relevance to multimorbidity research and feasibility within the available datasets. These concepts align with frameworks from the NIHR AIM Research Consortium and the existing literature [55–59], ensuring that they are supported by prior operationalisations.

2. *Background – other similar cohorts, in similar infrastructure? The paper does not give an overview of any other similar or overlapping cohorts that might be relevant for the field, and how this cohort is different from any other population cohorts. Most older cohorts are not couched in a Trusted research Environment, but just how unique this TERinitiative is at this point in time is not discussed.*

We acknowledge that similar cohorts have been created for multimorbidity studies (e.g., the cohort used in [61]), but there is no directly overlapping cohort. One of the key motivations for publishing this paper is that, in the field of multimorbidity research, cohorts are often created uniquely for each study, following specific rules and criteria. However, studies rarely share the exact codes or methodologies used to define their cohorts, leading to challenges in replicating or building upon prior work. In contrast, our approach is designed to be fully transparent and reproducible. We provide not only a detailed explanation of our methods but also all the code and the pipeline used to create and analyse the cohort, including the phenotypes. Additionally, the cohort is hosted in a Trusted Research Environment (TRE), which adds a layer of security and governance that sets it apart from most older cohorts.

We added a paragraph in the "strengths and limitations section" to address this.

3. *Concepts:*

3.1 *The authors refer to the most simple definition of multimorbidity: two long-term simultaneous conditions. However, there is also common agreement that complex multimorbidity, is the most important to understand, i.e. multimorbidity that has some kind of impact the patient's life in terms of burden of symptoms, treatments, follow-up etc. Given that this project seeks to look at burdensomeness, I would expect them to be interested in complex multimorbidity. However, the authors do not reflect upon the difference between simple and complex multimorbidity, or explain if complexity is something they are trying to capture and define.*

Thank you for this comment, we agree that two long-term simultaneous conditions is the most commonly used definition of multimorbidity, and we take on board the comment

about complex multimorbidity. However - complex multimorbidity has been defined in different ways in the literature. For example, this well-conducted study by Head et al it is defined as 'comprising at least three chronic conditions affecting at least three body systems.', see [1]. Others have suggested combined physical and mental health multimorbidity, such as this paper [2] . Your definition suggests that it refers to impact on a person's life, which is a very reasonable suggestion, but there is lack of consensus. This review is a helpful overview: [3]. The MELD-B project has taken a patient-centred approach to explore the burden or 'work' of living with multimorbidity, enhancing previous concepts of treatment burden and symptom burden as described in our qualitative evidence synthesis [4]. This clearly overlaps with 'complexity' but, involving patient and public contributors, we have critically discussed the meaning of complexity, which is often considered from a health service perspective. We have not tried to define complexity but have developed eight themes of 'work' which describe the lived experience of multimorbidity and illustrate the complexity for many patients.

3.2 *Also, the following concepts are key to the research question, but are not described at all, only mentioned in passing: What are the burdensomeness concepts? The cohort is established to study "burdensomeness", and the authors describe that this term has been operationalised. This could be a potential original contribution, but the term remains undefined and un-operationalised.*

To address this concern, we have moved the following paragraphs from Sec.2 to the Introduction "These concepts include indicators of the 'work' or 'burden' associated with living with multiple long-term conditions (MLTCs) such as symptoms, emotions, indicators of financial stress, and observable and measurable information relevant to health or healthcare, such as medical diagnoses, blood tests, appointments, hospital admissions and number of medications".
These work concepts are derived from a substantial qualitative evidence synthesis of 46 studies describing the experience of living with MLTCs. This evidence synthesis identified eight 'themes of work' including 'learning and adapting' (learning about new and existing conditions and their management, including the physical and psychological adjustments required), 'accumulation and complexity' (the additional and cumulative burden of living with multiple, rather than just one, long-term condition), 'investigation and monitoring' (the work of tests related to MLTCs), 'health service and administration' (work related to navigating health services) 'medication work' (work associated with taking and managing medications), 'financial work' (the financial impact of living with MLTCs), 'symptom work' and 'emotional work'. We have derived a series of indicators based on these themes using clinical code lists and engineered concepts, see [4] **(a separate paper has been submitted on this work). We hope that moving this to the introduction, our additional wording, and the reference to the qualitative evidence synthesis clarifies this question.**

3.3 *What is the concept of "sensitive" codes? There is an identification of certain diagnosis codes as "sensitive" in the appendix. Most of them are contagious diseases that are linked to stigma. However, I am wondering why we should not learn more about multimorbidity which includes such socially challenging conditions? Is it not even more important to understand the patterns*

in marginalised and vulnerable populations? Is this the result of a privacy concern? Was this a requirement from the ethics board?

Sensitive codes are codes that relates to certain protected treatments or diagnoses. We updated the text in Sec2.3 to make it clearer. "The current lists of all known sensitive ICD-10 and ReadV2 codes, which are based on a combination of sensitive codes provided by Digital Health and Care Wales (DHCW), a published list of sensitive codes for England and any other code flagged as sensitive by the SAIL team[1], are downloaded from the Concept Library [5] and processed as part of the pipeline." The use of data related to sensitive codes is restricted by NHS Wales. We updated the text to clarify this point better.

4. *Establishing the e-cohort: Certain choices were made but not argued: "Note that if a person has an available maternal record but the mother is not included in SMC, we considered this individual as not having a maternal record (n = 1248, 0.1%) thus, all the MAT_ALF linked to the SMYC form a subset of the SMC". If I understand this correctly, this excludes a set of persons whose mother was not included in the larger cohort. This would probably be persons who lived with their father, were orphaned, migrants without parents? Many of these would represent vulnerable populations. While the n is small, it might be a very important group to study. Thus, such an exclusion should be underpinned by arguments. My apologies if I misunderstood, in which case you might want to clarify the text.*

   We updated the text to make this passage clearer, see end of Sec2.2.1. If a child (ALF) can be linked to a mother (MAT_ALF) in either NCCH or MIDS, but the mother *is not* in SMC, then we include the child in SMYC but we consider this individual as not having a maternal record.

5. *Cohort censoring: In 2.2 the authors write: "As a longitudinal e-cohort, the number of individuals will change throughout the study as they can leave or join the e-cohort at any time during the study period." Which leads me o believe that individuals can re-enter the cohort if they leave Wales and then come back. Later the authors write: "Note that once an individual meets one of the censorship criteria, they are not allowed to re-enter the e-cohort, see Supplementary". Which indicates that a person can not re-enter the cohort. There may be good reasons for such a choice, but the authors do not provide any insights into their reasoning. Again, persons who move around may differ in important ways, from those who do not, thus the choice should be underpinned by some logic.*

   Before deciding not to allow individuals to re-enter the cohort, we analysed the full cohort to determine how many individuals would have re-entered (i.e., left Wales and then returned during the cohort period). The results of this analysis are provided in Appendix SA. Our findings showed that over 90% of individuals joined the cohort only once and did not return after moving out of Wales. Given this and the challenges associated with allowing re-entry, we decided to prevent individuals from re-entering the cohort.

   The primary challenges involve recovering accurate residency and GP registration data, as there is often a delay in updating these records when someone moves. This delay complicates the accurate tracking of individuals' movements and introduces inconsistencies in the data

[1] The current set of sensitive codes covers: miscarriage, HIV/AIDS, pregnancy termination and sexually transmitted diseases. The sensitive code lists are processed as part of the pipeline.

6. *Anonymity: The authors make clear that no one organisation can unlock the identity information linked to the individualised study-id. However, to be fully anonymous, it must not be possible to "backwards identify" a person: I.e. if you combine a number of characteristics with information found elsewhere (eg social media), you may still identify the person. Also if there is a key between the study-id- and the identifying information, which allows unlocking the identity of a person under given circumstances (i.e. – need for data quality checks), the data can not be called anonymous. Individualised data, linked by the same study id is pseudonymous. Could the authors describe whether these two forms of re-identification could be theoretically possible?*

   Thank you for your comment. We used the term 'anonymised' in reference to SAIL, as it stands for Secure Anonymised Information Linkage and is referred to as an anonymised Trusted Research Environment (TRE). However, we acknowledge that, according to the Information Commissioner's Office (ICO), SAIL data could currently be described as 'pseudonymised' since each individual is assigned a unique personal identifier that allows linkage across data sources and we added this note at the end of the first paragraph in Sec 2.1 "Note that while SAIL is referred to as an anonymised TRE, the ICO might also describe it as pseudo-anonymised, see [6]".
   Moreover, SAIL only permits results to be taken out of the environment once the risk of re-identification has been minimised via a standardised dual review and standardised disclosure control policy to ensure privacy protection is upheld. In response to your comment, we have clarified in Section 2.1 that the data are pseudonymised rather than fully anonymised.

7. *References : Some of the references could not be accessed by this reviewer, as the URLs were either "404 -page not found", or they were behind a log-in barrier. References should only include souces that can be accessed by the general academic reader. Ex: • 53] J. Dylag, R. Chiovoloni, A. Akbari, S. Fraser, M. Boniface, A Tool for Automating the Curation of Medical Concepts derived from Coding Lists, 2024 (n.d.). https://git.soton.ac.uk/meld/meldb/concepts-processing (accessed April 22, 2024). • All codes and scripts used in this study are available for others to access here: https://github.com/SwanseaUniversityDataScience/1377-MELD_B-CohortCuration*

   Thanks for pointing this out. I corrected the links and made the Github repo public.

Minor comments

8. *A few acronyms that are not explained: Example: WIND, MAT_ALF.*

   WIMD is explained in Sec 2.1 ("Welsh Index of Multiple Deprivation, 2019 version (WIMD 2019)", while MAT_ALF is explained at the beginning of Sec 2.2.1

9. *The Flow-chart of the development of the two cohorts: In the SMYC chart, the 1248 individuals excluded because the maternal record is missing, are not represented.*

   These individuals are not excluded from SMYC, that's why they are not represented in Figure 2. . We just don't include their MAT_ALF because this is not part of the ALF for SMC. I rephrased this in the paper to make it clearer.

10. *The WIMD -quintiles – e.g. 20%-tiles, in table one, is marked as approx. 11%, which is illogical. This probably relates to the fact that table 1 shows characteristics at baseline, at the cohort start of follow-up date, i.e. 2,99 mill. To make the table consistent – either provide baseline characteristics for everyone, upon entry into the cohort, no matter when they entered,(N=5,2mill), or at baseline (N=2,9 mill). Do not mix the two.*

    We removed the number of individuals within each WIMD quintile at cohort start date in Table 1 to avoid confusion.

11. *Study end-point – is a confusing term. I first thought this was an outcome which would cause censoring. Then I understood study end point was synonymous to "end of follow-up". Consider changing to "end of follow-up"*

    Thanks, we agreed to change study end-point to end of follow-up for a better clarity.

[1] A. Head, K. Fleming, C. Kypridemos, P. Schofield, J. Pearson-Stuttard, and M. O'Flaherty, "Inequalities in incident and prevalent multimorbidity in England, 2004-19: a population-based, descriptive study," *Lancet Healthy Longev*, vol. 2, no. 8, pp. e489–e497, Aug. 2021, doi: 10.1016/S2666-7568(21)00146-X.

[2] P. A. Coventry, N. Small, M. Panagioti, I. Adeyemi, and P. Bee, "Living with complexity; Marshalling resources: A systematic review and qualitative meta-synthesis of lived experience of mental and physical multimorbidity Multimorbidity in Primary Care Knowledge, attitudes, behaviors, education, and communication," *BMC Fam Pract*, vol. 16, no. 1, pp. 1–12, Nov. 2015, doi: 10.1186/S12875-015-0345-3/FIGURES/1.

[3] S. Pati, C. MacRae, D. Henderson, D. Weller, B. Guthrie, and S. Mercer, "Defining and measuring complex multimorbidity: a critical analysis," *Br J Gen Pract*, vol. 73, no. 733, pp. 373–376, Aug. 2023, doi: 10.3399/BJGP23X734661.

[4] E. Holland *et al.*, "The impact of living with multiple long-term conditions (multimorbidity) on everyday life – a qualitative evidence synthesis," Jun. 2024, doi: 10.21203/RS.3.RS-4419503/V1.

[5] "Concept Library." Accessed: Sep. 21, 2023. [Online]. Available: https://conceptlibrary.saildatabank.com/

[6] ICO Information Commissioner's Office, "Chapter 3: pseudonymisation Draft anonymisation, pseudonymisation and privacy enhancing technologies guidance."
